# CLIC3 interacts with NAT10 to inhibit N4-acetylcytidine modification of p21 mRNA and promote bladder cancer progression

**DOI:** 10.1038/s41419-023-06373-z

**Published:** 2024-01-05

**Authors:** Yujun Shuai, Hui Zhang, Changhao Liu, Junting Wang, Yangkai Jiang, Jiayin Sun, Xincheng Gao, Xiaochen Bo, Xingyuan Xiao, Xin Liao, Chao Huang, Hebing Chen, Guosong Jiang

**Affiliations:** 1 Department of Urology, Union Hospital, Tongji Medical College, Huazhong University of Science and Technology, Wuhan, 430022 China; 2 Institute of Health Service and Transfusion Medicine, Beijing, 100850 China; 3 Department of Urology, Zhongnan Hospital of Wuhan University, Wuhan, 430071 China; 4 Department of General Medicine, Tongji Hospital, Tongji Medical College, Huazhong University of Science and Technology, Wuhan, 430030 China

**Keywords:** Oncogenes, Acetylation, Expression systems

## Abstract

Chromatin accessibility plays important roles in revealing the regulatory networks of gene expression, while its application in bladder cancer is yet to be fully elucidated. Chloride intracellular channel 3 (CLIC3) protein has been reported to be associated with the progression of some tumors, whereas the specific mechanism of CLIC3 in tumor remains unclear. Here, we screened for key genes in bladder cancer through the identification of transcription factor binding site clustered region (TFCR) on the basis of chromatin accessibility and TF motif. CLIC3 was identified by joint profiling of chromatin accessibility data with TCGA database. Clinically, CLIC3 expression was significantly elevated in bladder cancer and was negatively correlated with patient survival. CLIC3 promoted the proliferation of bladder cancer cells by reducing p21 expression in vitro and in vivo. Mechanistically, CLIC3 interacted with NAT10 and inhibited the function of NAT10, resulting in the downregulation of ac4C modification and stability of p21 mRNA. Overall, these findings uncover an novel mechanism of mRNA ac4C modification and CLIC3 may act as a potential therapeutic target for bladder cancer.

## Introduction

Bladder cancer, classified as non-muscle-invasive bladder cancer (NMIBC) and muscle-invasive bladder cancer (MIBC), is one of the most common malignancies in the urinary system with an estimated 81,180 new cases and 17,100 deaths in the United States in 2022 [1, 2]. Approximately 30% of bladder cancers are MIBC, of which 50% of patients will still die from tumor progression within five years [3]. Therefore, it is essential to identify the key pathogenic genes of bladder cancer and elucidate its molecular mechanism to improve the treatment strategies for bladder cancer patients.

Eukaryotic genome is tightly packed into chromatin, and only open chromatin can be targeted by regulatory factors such as transcription factors (TFs) [4]. The organization of accessible chromatin across the genome reflects a network of permissible physical interactions that cooperatively regulate gene expression [5, 6]. Moreover, the assay of transposase accessible chromatin (ATAC-seq) and DNase I-hypersensitive site sequencing (DNase-seq) are the measurements of chromatin accessibility that capture similar regulatory information [7, 8]. Previously, we developed a method to identify transcription factor binding site clustered regions (TFCRs) on the basis of chromatin accessibility and TF motif [9]. However, TFCR-based joint profiling of chromatin accessibility data from ATAC-seq and DNase-seq to screen for key genes in bladder cancer is yet to be revealed.

The chloride intracellular channel (CLIC) protein family is an important negative ion channel in the human body [10, 11] that has also been implicated in tumor regulation [12, 13]. CLIC3, which belongs to a member of CLIC protein family, can act as a glutathione (GSH)-dependent oxidoreductase to promote tumor invasion via inhibiting the ability of TGM2 [14]. Meanwhile, CLIC3 and Rab25 collaborate to promote aggressiveness of pancreatic ductal adenocarcinoma through recycling integrin [15]. More recently, high expression of CLIC3 is associated with the poor clinicopathological factors and poor prognosis of bladder cancer patients [16]. However, the role and mechanism of CLIC3 in regulation of bladder cancer still remain unclear.

N4-acetylcytidine (ac4C) is identified as a novel mRNA modification that acetylation of Cytidine in mRNA can promote stability and translation [17]. Furthermore, ac4C is associated with the occurrence and metastasis of a variety of cancers, including gastric cancer [18], esophageal cancer [19] and colon cancer [20]. As the only known ac4C writer, the nucleolar protein N-acetyltransferase 10 (NAT10) has both acetyltransferase and RNA-binding functions that regulates ac4C modification of RNA [21, 22]. Notably, recent studies suggest that NAT10 promotes the progression of gastric cancer via ac4C modification of COL5A1 [18]. On the other hand, NAT10 acts as a tumor suppressor that acetylated p53 at K120 to inhibit cell proliferation in colorectal carcinomas [23]. Therefore, elucidating the regulatory factors of NAT10 and specific biological circumstances that determines the divergent functionality of NAT10 are of paramount importance.

In this study, we identified the TFCRs by integrating chromatin accessibility data of ATAC-seq and DNase-seq with TF motif, and discovered that CLIC3 was upregulated in bladder cancer tissues. Functionally, CLIC3 could promote the proliferation of bladder cancer cells in vitro and in vivo. Mechanistically, CLIC3 interacted with NAT10 and inhibited its function, resulting in the downregulation of ac4C modification and stability of p21 mRNA. Collectively, we proposed a TFCR-based framework to screen for key genes in bladder cancer, and revealed that CLIC3 could be a regulatory factor of NAT10-catalyzed ac4C modification, elucidating a novel mechanism for mRNA ac4C modification. Therefore, CLIC3 might act as a promising therapeutic target in bladder cancer.

## Materials and methods

### Identification of TFCRs and candidate genes

The identification of TFCR was described in detail according to previously published paper [9]. Briefly, we regarded each transcription factor binding site (TFBS) on the genome wide as a Gaussian distribution with a bandwidth of 300 bp centered on this point. Each peak in the density profile was considered as a TFCR. To characterize the features of each TFCR, we ruled out the TFBS with Gaussian signal intensity greater than 0.1 in each TFCR. The window for each TFCR was determined by finding the maximum distance (in bp) from the TFCR to a contributing TF and then adding 150 bp (one-half of the bandwidth). From these, we defined the average score of all ATAC-seq peaks or DNase-seq peaks with overlapping interval as the chromatin accessibility score (SC).

Based on the TFCR identification method, we identified 95 genes (Supplementary Table 1). To filter out false positives, we screened for genes with FPKM ≥ 5, ultimately obtaining 53 candidate genes (Supplementary Table 1).

### Patient tissue specimens

Eight-six pairs of bladder cancer tissues and paired adjacent normal bladder tissues were obtained from patients suffering radical cystectomy at Department of Urology of the Union Hospital affiliated of Tong Medical College (Wuhan, PR China) from 2014 to 2019. All the specimens were classified by at least two experienced clinical pathologists independently according to the criteria of the sixth edition TNM classification of the International Union Against Cancer. This study was approved by the ethics review committee of Tongji Medical College of Huazhong University of Science and Technology (Wuhan, P.R. China) and all patients received written informed consent before the research started. These specimens were immediately snap-frozen in liquid nitrogen, and then stored at −80 °C. Detailed information is presented in Supplementary Table 2. All of the patients were followed up on a regular basis, overall survival (OS) time was determined from the date of surgery to the date of death or the date of the last follow-up visit for survivors.

### Cell lines

Human bladder cancer cell lines J82, TCCSUP, EJ, UMUC3, T24, 5637 and RT4, human immortalized uroepithelium cell line SV-HUC-1, were purchased from American Type Culture Collection (ATCC, USA). The human bladder cancer cell line T24T was provided by Dr. Dan Theodorescu (Departments of Urology, University of Virginia, Charlottesville, VA, USA) as described in our previous studies [24]. J82 and TCCSUP cells were cultured in MEM (Gibco, USA) supplemented with 10% FBS (Gibco, USA), 1% penicillin/streptomycin (Gibco, USA). T24T and UMUC3 cells were cultured in DMEM (Gibco, USA) supplemented with 10% FBS (Gibco, USA), 1% penicillin/streptomycin (Gibco, USA). 5637, EJ, T24 and RT4 cells were cultured in RPMI-1640 medium (Gibco, USA) supplemented with 10% FBS (Gibco, USA), 1% penicillin/streptomycin (Gibco, USA). SV-HUC-1 were cultured in F-12K medium (Gibco, USA) supplemented with 10% FBS (Gibco, USA), 1% penicillin/streptomycin (Gibco, USA). Cells were cultured in an incubator at 37 °C with humidifified atmosphere of 5% CO_2_. All bladder cancer cell lines were confirmed within 6 months before use by using a short tandem repeat profiling and were confirmed negative for Mycoplasma contamination.

### RNA preparation and qRT-PCR

Total RNA of tissue samples and cell lines were extracted by TRIzol reagent (Invitrogen, USA) according to the manufacturer’s instructions. cDNA was synthesized by HiScript III RT SuperMix for quantitative PCR (Vazyme, China). The real-time PCR analyses were performed using SYBR Green Master Mix (Vazyme, China). The primers are listed in Supplementary Table 3. The results were analyzed with the StepOne Plus Real-Time PCR System (Applied Biosystems, USA) and 2^−∆∆Ct^ method was used to analyzed the results of transcript levels.

### Plasmids construction and stable transfection

The short hairpin RNAs targeting CLIC3, NAT10 and p21 (Supplementary Table 3) were synthesized by TSINGKE (Wuhan, China), and were cloned into pLKO.1 vector (Sigma-Aldrich). Truncations of CLIC3 and NAT10 were amplified with primers (Supplementary Table 3), and were cloned into pcDNA3.1-3×Flag-C vector (Sigma-Aldrich). Point mutations of p21 3′-UTR were amplified with primers (Supplementary Table 3), and were cloned into psiCHECK-2^TM^ vector. To construct CLIC3 overexpression plasmids, human CLIC3 cDNAs were synthesized by TSINGKE (Wuhan, China) and cloned into pcDNA3.1-3×Flag-C vector. Lipofectamine 2000 (Life Technologies, USA) was used for plasmid transfection according to the manufacturer’s instructions. Stable cell lines were screened by administration of puromycin (Invitrogen).

### Luciferase reporter assay

The p21 promoter reporter vector (pGL3-Basic vector) and p21 3′-UTR reporter vector (psiCHECK^TM^−2 vector) were designed and synthesized by TSINGKE (Wuhan, China). The p21 promoter reporter was transiently transfected with Renilla control plasmid, concomitantly with CLIC3 shRNA Vectors. The p21 3′-UTR reporter was transiently transfected with CLIC3 shRNA Vectors. Dual luciferase reporter assay detection kit (Promega, USA) was used to measure the luciferase activities according to the manufacturer’s protocol.

### Western blotting

Tissues and cell lines were collected and lysed in RIPA buffer (Thermo Scientific) supplemented with protease inhibitor cocktail (MCE, China). The concentration of total protein was measured by BCA protein assay kit (HYcezmbio, China). Total protein was subjected to 10% SDS-PAGE gels and transferred to nitrocellulose membranes (Millipore). After blocking with 5% non-fat milk for 1 h at room temperature, membranes were incubated with primary antibodies overnight at 4 °C. Then, membranes were incubated in the specific horseradish peroxidase (HRP)-conjugated secondary antibodies for an hour at room temperature. All members were visualized by ECL substrate kit (Millipore) and the images were obtained by Bio Spectrum 600 Imaging System (UVP). Antibodies used included primary antibodies against ACTB (81115-1-RR, Proteintech), Tubulin (11224-1-AP, Proteintech), H3 (17168-1-AP, Proteintech), CLIC3 (15971-1-AP, Proteintech), NAT10 (13365-1-AP, Proteintech), p21 (10355-1-AP, Proteintech) and Flag (ab205606, abcam); HRP-conjugated secondary goat anti-mouse (SA00001-1, Proteintech), or goat anti-rabbit (SA00001-2, Proteintech) antibodies.

### Immunofluorescence assay

Bladder cancer cells grown on confocal dishes were fixed with 4% paraformaldehyde for 30 min at temperature and then permeabilized with 0.1% TritonX-100 for 10 min. After blocking with 1% BSA for an hour at room temperature, the dishes were incubated with antibodies specific for CLIC3 (15971-1-AP, Proteintech) or NAT10 (13365-1-AP, Proteintech) overnight at 4 °C. The next day, the dishes were washed with PBS and then incubated with corresponding secondary antibody for 1 h at room temperature, followed by sealing with parafilm containing DAPI. The images were photographed under a Nikon A1Si Laser Scanning Confocal Microscope (Nikon Instruments Inc., Japan).

### Nuclear and cytoplasmic extraction

Nuclear and Cytoplasmic fractions were isolated by Nuclear and Cytoplasmic Protein Extraction Kit (Beyotime, China) according to the manufacturer’s instructions. Briefly, cells were lysed in cytoplasmic buffer on ice for 15 min. After centrifugation at 16,000 × *g* for 5 min at 4 °C, the supernatant was collected as cytoplasmic protein. Then, the pellet was lysed in nuclear buffer on ice for 30 min. After centrifugation at 16,000 × *g* for 10 min at 4 °C, the supernatant was collected as nuclear protein.

### Cell counting kit-8 assay

Cell viability was detected by CCK-8 assay (HYcezmbio, China) following the manufacturer’s instructions. Cells stably transfected with plasmids were cultured in 96-well plates of 3 × 10^3^ per well and cultured for different time periods, respectively (0, 24, 48, and 72 h). Then, CCK-8 solution (10 μL) was added into each well and incubated at 37 °C for 2 h. The absorbance at 450 nm was measured using an automatic microplate reader (Synergy4; BioTek, Winooski, VT, USA).

### EdU assay

Bladder cancer cells were seeded into 96-well plates at a density of 3 × 10^4^ cells per well. The EdU assay was performed by using the 5-ethynyl-20-deoxyuridine assay (EdU; Cell Light EdU DNA imaging Kit, Ribo Bio) as the protocol described. EdU (100 μmol/L) was added to the medium and incubated with the cells for 2 h. The images were photographed under Olympus FSX100 microscope (Olympus).

### Colony formation assay

Bladder cancer cells were seeded into 6-well plates at a density of 800 cells per well and cultured for 2 weeks. The cell colonies were fixed with 4% poly-formaldehyde for 30 min and stained with 0.1% crystal violet (Sigma-Aldrich) for another 30 min at room temperature. Cell colonies with more than 50 cells were counted.

### Flow cytometry assay for the cell cycle and apoptosis

For the cell cycle assay, bladder cancer cells were seeded into a six-well plate. Cells were harvested to analyze the proportion of cell apoptosis by flow cytometry (Becton Dickinson) after stained with propidium iodide buffer (BD PharMingen). The results were analyzed by the ModFit LT software.

For the apoptosis assay, bladder cancer cells were harvested to analyze cell apoptosis by flow cytometry (Becton Dickinson) after stained with FITC Annexin V and propidium iodide (PI) staining (BD Pharmingen). FlowJo software was used to analyze the results.

### Tumor xenograft assay

All procedures for the animal experiments were approved by the Animal Care Committee of Tongji Medical College (Wuhan, PR China). Four-week-old female BALB/c nude mice were chosen for tumor xenografts experiments. All animals were randomized assigned to experimental or control group (at least five mice per group), while no blinding was used in the experiments. Bladder cancer cells (5 × 10^6^) were subcutaneously injected into the right axilla of the nude mice. Tumor growth rates were monitored every other week. Tumor volume was calculated according to the formula (Tumor volume = π/6 × length × width^2^). At the end of the experiment, mice were sacrificed and tumors were excised and weighed. All animal experiments were allowed in the light of NIH Guidelines for the Care and Use of Laboratory Animals and approved by the Animal Care Committee of Tongji Medical College.

### RNA sequencing

Total RNA was isolated from CLIC3-knockdown bladder cancer cells and the corresponding control cells using TRIzol reagent (Invitrogen) following the manufacturer’s instructions. Transcriptome sequencing were accomplished by Novogene (Tianjin, China). Differentially expressed predicted target genes were screened according to the criteria of |log_2_(FoldChange)| ≥ 4 and *p* value top 500. RNA Sequencing results were deposited in the Gene Expression Omnibus database The following secure token has been created to allow review of record GSE232965 while it remains in private status: mfmxikyuzzqzxol.

### RNA degradation assay

Bladder cancer cells were seeded into 6-well plates to get 60% confluency after 24 h. Cells were treated with 5 μg/mL actinomycin D for 0, 2, 4, 6, 8 and 10 h and collected at the same time. The total RNA was extracted by TRIzol reagent (Invitrogen) according to the manufacturer’s instruction and analyzed by qRT-PCR. The turnover rate and half-life of mRNA was estimated according to previously published paper [25].

### Coimmunoprecipitation (Co-IP)

Briefly, 2 × 10^7^ cells were lysed in 2 ml Co-IP buffer (20 mM Tris-HCL, pH 7.5, 150 mM NaCl, 1 mM EDTA and 0.5% NP-40) supplemented with protease inhibitor cocktail (MCE, China) for an hour on ice. Five percent of cell lysate was used as input, remaining were divided equally into two parts and incubated with 4 μg target or IgG antibody overnight at 4 °C, respectively. Total protein was immunoprecipitated with antibodies against CLIC3 (WH0009022M2, Sigma-Aldrich), NAT10 (13365-1-AP, Proteintech), Flag (ab205606, abcam), control rabbit IgG (ab172730, abcam) or control mouse IgG (ab190475, abcam). Then Protein A/G Magnetic Beads (MCE, China) were incubated with cell lysate for 2 h at 4 °C. After washing three times in Co-IP buffer and boiling for 10 min at 95 °C, purified proteins were detected by Western blotting. The following secondary antibodies were used for immunoblotting: HRP-goat anti-mouse IgG heavy chain specific (AS064, ABclonal), anti-mouse IgG light chain specific (AS062, ABclonal) or HRP-mouse anti-rabbit IgG conformation specific (5127, Cell Signaling Technology).

### Silver staining and mass spectrometry analysis

For the silver staining, 10% SDS-PAGE gels were treated by the PAGE Gel Silver Staining Kit (Solarbio) after electrophoresis was completed. Mass spectrometry analysis was performed by APTBIO (Shanghai, China). The identification and quantification of differential proteins were accomplished by Proteome Discoverer software (version 1.4; Thermo Fisher Scientific).

### RNA immunoprecipitation assay (RIP)

RIP assay was performed according to the instructions of Magna RIPTM RNA-Binding Protein Immunoprecipitation Kit (Millipore). In brief, approximately 1 × 10^7^ cells were harvested and lysed to extract total protein. Total protein was immunoprecipitated with antibodies against CLIC3 (WH0009022M2, Sigma-Aldrich), NAT10 (13365-1-AP, Proteintech), rabbit IgG (ab172730, abcam), mouse IgG (ab190475, abcam) or Protein A/G magnetic beads (Life Technologies). After treating with proteinase K, input and co-immunoprecipitated RNAs were extracted with a RNeasy Mini kit (QIAGEN, Germany) according to the manufacturer’s instructions and analyzed by qRT-PCR.

### ac4C-RIP assay

Total RNA was isolated from cells using TRIzol reagent (Invitrogen) according to the manufacturer’s instruction. ac4C-RIP was performed using a GenSeq ac4C-RIP kit (Cloudseq biotech) according to the manufacturer’s instructions. Briefly, a total of 180 μg RNA was fragmented in Fragmentation Buffer for 6 min at 70 °C. We used 3 μg of RNA fragments as input, remaining were divided equally into two parts and incubated with 4 μg anti-ac4C (Cloudseq biotech) or IgG (Cloudseq biotech) antibody for an hour at room temperature, respectively. After incubating with corresponding magnetic beads and purification, relative ac4C modification of target genes was detected by qRT-PCR.

### Statistics analysis

All in vitro experiments were repeated independently at least three times to ensue reproducibility. All data were indicated as Mean ± SD. Statistical analysis was performed using GraphPad Prism 8.0 software. Student t test and ANOVA were used to assess the group difference. The Fisher’s exact test or Chi-square test was used to analyze the relationship between CLIC3 expression and clinicopathologic characteristics. Kaplan–Meier survival curve and log-rank test were employed to evaluate survival difference. Pearson’s correlation test was used to analyzed the correlation between the transcript levels of CLIC3 and those of NAT10 and p21 in human bladder cancer samples. *P* < 0.05 was considered statistically significant.

## Results

### Identification of TFCRs to screen for key genes in bladder cancer

**Fig. 1:**
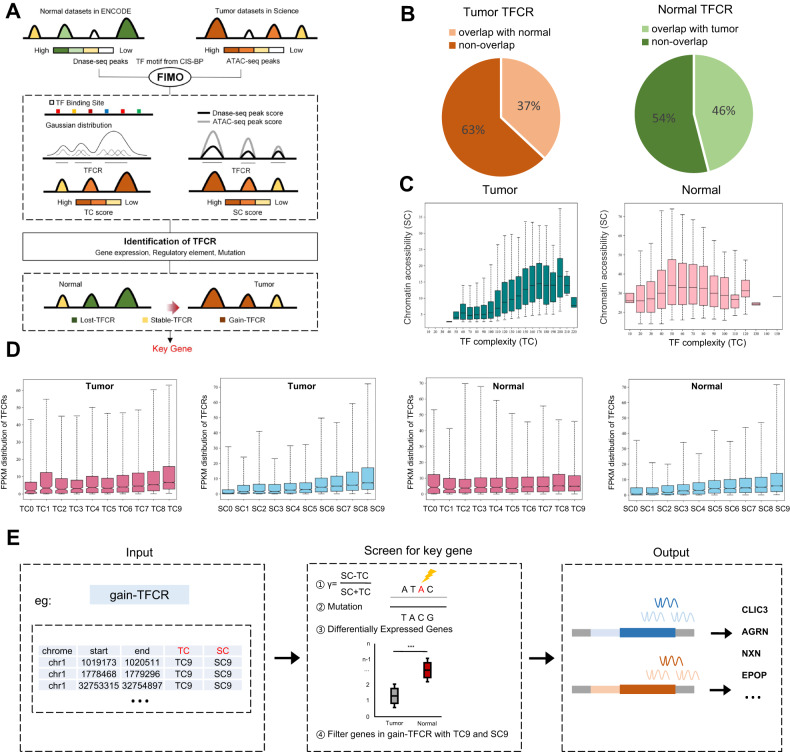
Identification of TFCRs to screen for key genes in bladder cancer. **A** Schematic diagram of our TFCR-based framework showed the screening for key genes in bladder cancer. **B** Pie chart showed the differences between tumor TFCRs and normal TFCRs. **C** Barplot showed the relationship between TC and SC in bladder cancer tissues. **D** Barplot showed the distribution of highly expressed genes in tumor TFCRs and normal TFCRs. **E** The pipeline showed the identification for key genes in bladder cancer.

We performed an improved analysis to identify TFCRs and screened for key genes in bladder cancer (Fig. 1A). Firstly, we collected ATAC-seq data of bladder cancer tissues from previous study in Science [26], DNase-seq data of normal bladder tissues in ENCODE database [27] and TF binding information in CIS-BP database [28], and identified TFCRs by FIMO [9] (Fig. 1A). As shown in Fig. 1B, 63% of 67,844 tumor TFCRs and 54% of 81,501 normal TFCRs did not overlap of each other, indicating that TFCRs in bladder cancer tissues were significantly different from those in normal bladder tissues. As previously reported that TFCRs could be characterized by TF complexity (TC) and chromatin accessibility score (SC) [29], we further investigated the relationship between TC and SC in bladder cancer and normal bladder tissues. Correlation analysis indicated that TC was positively correlated with SC when TC was less than 170 in bladder cancer tissues and TC was less than 50 in normal bladder tissues (Fig. 1C). However, the correlation disappeared when TC was greater than 170 in bladder cancer tissues and TC was greater than 50 in normal bladder tissues (Fig. 1C). We speculated that transcription factor binding site (TFBSs) might reach saturation with the increase of TC, which led TC and SC to an unbalanced state. To further determine the relationship between TFCRs and gene expression, we divided TC and SC into ten groups, TC0-TC9 and SC0-SC9. Notably, highly expressed genes were more likely to be enriched in TC9/SC9 TFCRs, especially in bladder cancer tissues (Fig. 1D). Furthermore, we investigated the proportion of TFCRs in regulatory elements, including promoters, enhancers, and CpG islands. The proportion of TFCRs was observed to increase steadily with the increase of TC and SC value (Fig. S1A). It is well accepted that the accumulated gene mutations play important roles in tumorigenesis [30], we next explored the mutation rates in different TC/SC value of TFCR. The mutation rates gradually increased in tumor and normal TFCRs with the increase of TC/SC value (Fig. S1B). Taken together, these findings implied that highly expressed genes, regulatory elements and accumulated mutations preferred to be enriched near TFCRs with high TC or high SC.

To further subdivide the differences between tumor TFCRs and normal TFCRs, we defined three concepts of TFCRs, gain-TFCR, lost-TFCR and stable-TFCR (Fig. 1A). Gain-TFCR and lost-TFCR reflects the unique TFCRs of tumor tissues and normal tissues, respectively. Stable-TFCRs reflects the overlapping TFCRs of tumor TFCRs and normal TFCRs. As we have suggested that TFCRs with high TC or SC may be the driver of tumorigenesis [29], we constructed a screening formula to calculate the carcinogenic potential γ of each gain-TFCR, and screened for genes covered by gain-TFCRs that belong to both TC9 and SC9 (Fig. 1E). Finally, we identified 53 candidate genes (Supplementary Table 1), which might be involved in the development of bladder cancer and serve as potential diagnostic markers or therapeutic targets for bladder cancer.

### CLIC3 is upregulated in bladder cancer, and mainly located in the nucleus

**Fig. 2:**
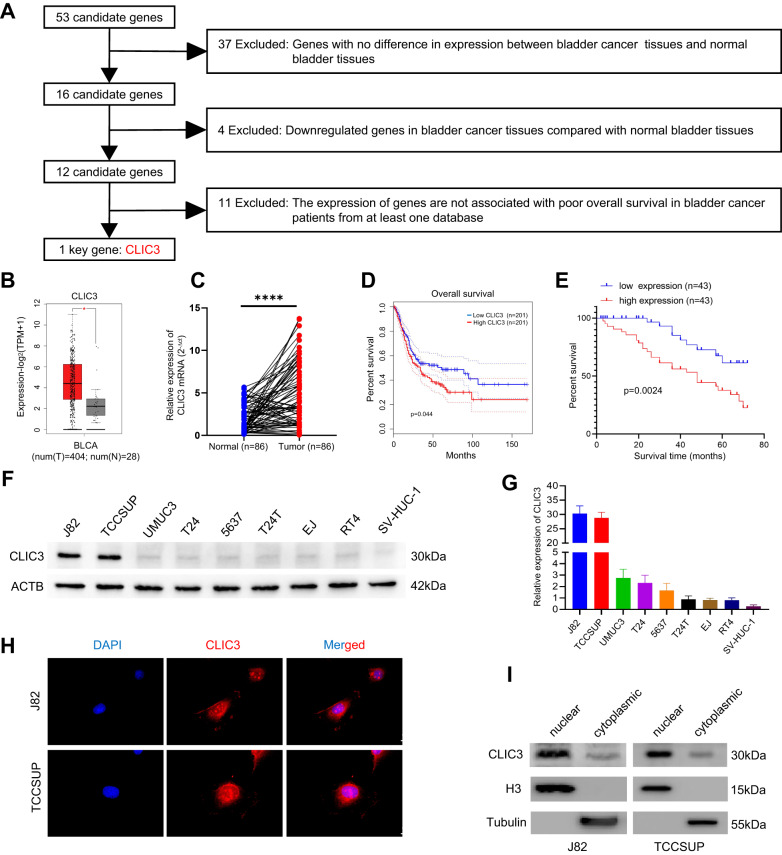
Identification and distribution of CLIC3. **A** Flow chart was performed to identified key genes in bladder cancer. Two databases (http://gepia.cancer-pku.cn/ and http://ualcan.path.uab.edu/) were used to identify the expression levels and overall survival of candidate genes. **B** The expression of CLIC3 in bladder cancer tissues compared with normal bladder tissues obtained from TCGA database. **C** qRT-PCR assay showed the relative levels of CLIC3 in human bladder cancer tissues compared with their adjacent normal tissues (*n* = 86). **D** Kaplan–Meier curves of overall survival (OS) in bladder cancer patients with low versus high expression of CLIC3 from TCGA database. **E** Kaplan–Meier curves of OS in baldder cancer patients (*n* = 86) with high or low expression of CLIC3. Patients were grouped by the median CLIC3 expression. (*P* = 0.0024, log-rank test). The expression of CLIC3 was detected by western blotting (**F**) and qRT-PCR (**G**) in bladder cancer cells. ACTB was used as internal control. **H** Immunofluorescence staining assay showed the distribution of CLIC3 (red) in J82 and TCCSUP cells; nuclei (blue) were stained with DAPI. Scale bar, 10 μm. **I** Western blotting indicated the distribution of CLIC3 in J82 and TCCSUP cells; H3 and Tubulin were applied as positive controls in the nucleus and cytoplasm, respectively; Data are presented as the means ± SD from three independent experiments. ****P < 0.0001 (Student *t* test).

Following the analysis pipeline (Fig. 2A and Fig. S2A–D), CLIC3 was identified as a key gene among 53 candidate genes. To further confirm the clinical role of CLIC3 in bladder cancer, we analyzed 86 pairs of bladder cancer and paired normal bladder tissues. Consistent with those identified in TCGA database, CLIC3 was significantly upregulated in bladder cancer tissues (Fig. 2B, C). Kaplan–Meier survival analysis revealed that high expression of CLIC3 was remarkably associated with poor prognosis in bladder cancer patients (Fig. 2D, E). Furthermore, CLIC3 was expressed at a high level in bladder cancer cells compared to normal urothelial cell SV-HUC-1, especially in J82 and TCCSUP (Fig. 2F, G). Immunofluorescence assay and nuclear/cytoplasmic protein extraction assay indicated that CLIC3 was mainly located in the nucleus of J82 and TCCSUP cells (Fig. 2H, I). In summary, these results demonstrated that CLIC3 was a key gene in bladder cancer, which was mainly localized in the nucleus and was significantly upregulated in bladder cancer.

### CLIC3 exerts pro-carcinogenic roles in bladder cancer

**Fig. 3:**
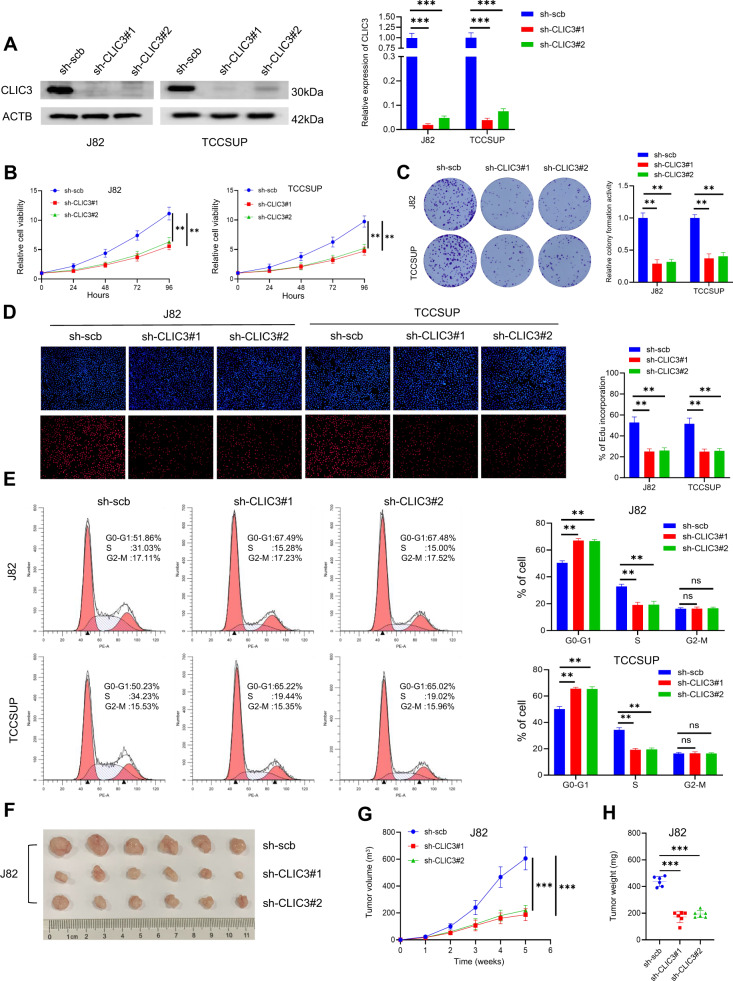
Knockdown of CLIC3 inhibits the proliferation of bladder cancer cells in vitro and in vivo. **A** The efficiency of CLIC3 knockdown in J82 and TCCSUP cells was detected by western blotting (left) and qRT-PCR (right). **B** CCK-8 assay revealed the cell viability of J82 and TCCSUP cells stably transfected with scramble, sh-CLIC3#1, or sh-CLIC3#2. **C** Colony formation assay was performed in J82 and TCCSUP cells stably transfected with scramble, sh-CLIC3#1, or sh-CLIC3#2. **D** EdU assay showed the proliferation of J82 and TCCSUP cells stably transfected with scramble, sh-CLIC3#1, or sh-CLIC3#2. **E** Flow cytometry assay revealed the cell cycle distributions in J82 and TCCSUP cells stably transfected with scramble, sh-CLIC3#1, or sh-CLIC3#2. Representative (**F**), in vivo growth curve (**G**), and weight at the endpoints (**H**) of xenograft tumors formed by subcutaneous injection of J82 cells stably transfected with scramble, sh-CLIC3#1 or sh-CLIC3#2 into the right flanks of nude mice (5 × 10^6^ cells per mouse; *n* = 6 for each group). Data are presented as the means ± SD from three independent experiments. ns, nonsignificant; **P < 0.01; ***P < 0.001 (Student *t* test).

To investigate the effects of CLIC3 in bladder cancer cells, two shRNAs targeting the coding region of CLIC3 (sh-CLIC3) were designed and stably transfected into J82 and TCCSUP cells. The efficiency of two shRNAs in these stable transfectants was detected by western blot and qRT-PCR (Fig. 3A). Subsequently, we observed that knockdown of CLIC3 attenuated cell viability, colony formation ability and cell proliferation of bladder cancer cells (Fig. 3B–D). As determined by cell cycle assays, inhibition of CLIC3 expression induced cell cycle arrest at G0/G1 phase (Fig. 3E). Moreover, silence of CLIC3 had no effect on apoptosis of bladder cancer cells (Fig. S3A). Subsequently, multiple bladder cancer cell lines with stable overexpression of CLIC3 were constructed (Fig. S3B). As we expected, ectopic expression of CLIC3 promoted the proliferation of bladder cancer cells (Fig. S3C, D).

To gain further insights into the function of CLIC3 in vivo, bladder cancer cells with stably transfected with scramble or sh-CLIC3 were subcutaneously injected into nude mice. Consistent with the biological roles of CLIC3 in vitro, CLIC3 knockdown led to a significant decrease in growth and tumor weight of xenograft tumors (Fig. 3F–H and Fig. S3E). Meanwhile, overexpression of CLIC3 also promoted the proliferation of bladder cancer cells in vivo (Fig. S3F). Collectively, these results impiled that CLIC3 exerted pro-carcinogenic roles in bladder cancer.

### Knockdown of CLIC3 increases the stability of p21 mRNA

**Fig. 4:**
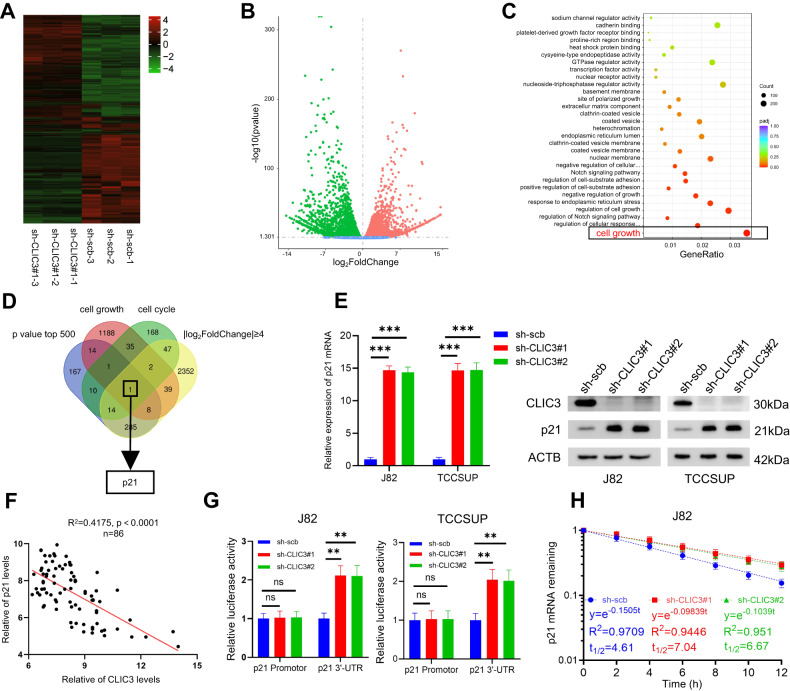
Knockdown of CLIC3 increases the stability of p21 mRNA. Heatmap (**A**) and Volcano map (**B**) depicted the differentially expressed mRNAs upon CLIC3 knockdown in J82 cells. Scramble group and sh-CLIC3#1 group contained three independent replicates, respectively. **C** GO enrichment analysis revealed the enriched pathways upon CLIC3 knockdown in J82 cells. **D** Venn diagram showed the differential genes detected by RNA-seq upon CLIC3 knockdown in J82 cells and overlapping analysis with cell growth- and cell cycle-related genes from AmiGO2 database. **E** The expression of p21 was detected by qRT-PCR (left) and Western blotting (right) in J82 and TCCSUP cells stably transfected with scramble, sh-CLIC3#1, or sh-CLIC3#2. **F** Analysis of the correlation between the transcript levels of CLIC3 and p21 in the tumor tissues of the 86 bladder cancer patients. The relative expression of CLIC3 and p21 was calculated by ∆Ct value. *P*-values were calculated by Pearson correlation analysis. **G** The relative luciferase activity of p21 promoter and 3’-UTR in J82 and TCCSUP cells stably transfected with scramble, sh-CLIC3#1, or sh-CLIC3#2. **H** The relative remaining levels of p21 mRNA was analyzed by qRT-PCR after treatment with actinomycin D (5 μg/μl) at the indicated time points in J82 cells transfected with scramble, sh-CLIC3#1, or sh-CLIC3#2. Data are presented as the means ± SD from three independent experiments. ns, nonsignificant; **P < 0.01; ***P < 0.001 (Student t test).

To determine the target genes and downstream signaling pathways of CLIC3 in bladder cancer cells, transcriptome analysis was performed in bladder cancer cells stably transfected with scramble or sh-CLIC3 (Fig. 4A, B). The Gene Ontology (GO) enrichment analysis showed that CLIC3 was highly associated with cell growth (Fig. 4C). Meanwhile, comprehensive analysis indicated that p21 might be the major target gene regulated by CLIC3 (Fig. 4D). Consistent with our RNA-seq results, knockdown of CLIC3 markedly increased p21 expression (Fig. 4E) and CLIC3 expression was inversely correlated with p21 expression in bladder cancer tissues (Fig. 4F). Furthermore, CLIC3 knockdown could enhance the luciferase activity of p21 3′-UTR, whereas the luciferase activity of p21 promoter was not affected (Fig. 4G). Therefore, we speculated that CLIC3 might regulate p21 expression by promoting mRNA stability, but not the activity of promoter. As expected, RNA degradation assay demonstrated that inhibition of CLIC3 expression could increase the stability of p21 mRNA (Fig. 4H).

**Fig. 5:**
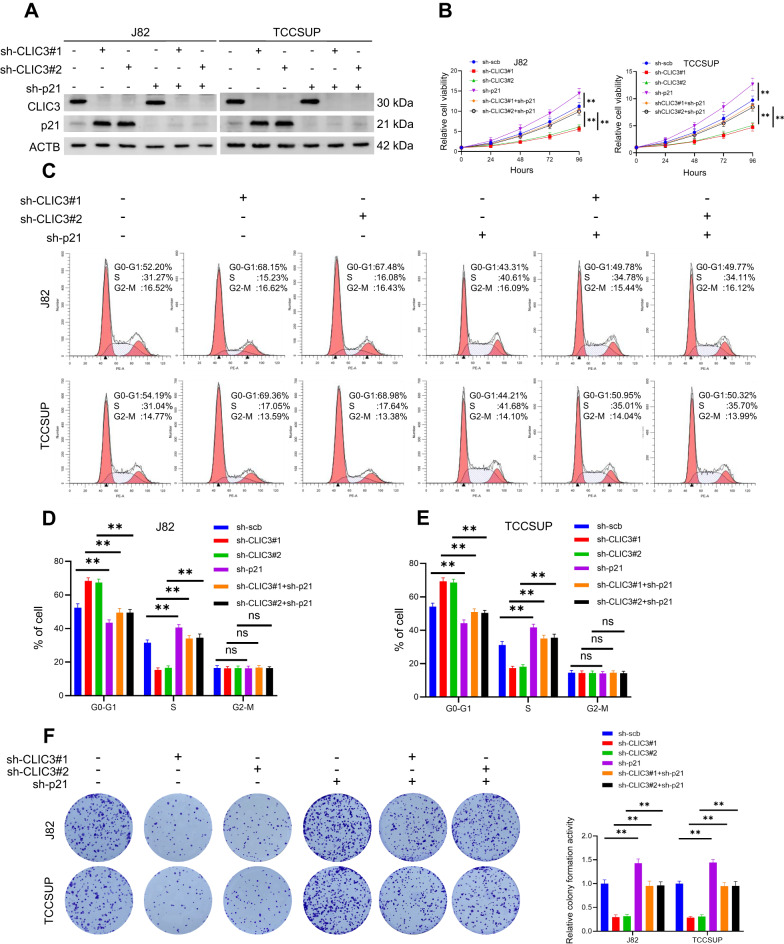
Knockdown of p21 reverses the effect of CLIC3 knockdown. **A** Western blotting with the indicated antibodies in J82 and TCCSUP cells stably transfected with scramble, sh-CLIC3#1 or sh-CLIC3#2, and those cotransfected with scramble or sh-p21. **B** CCK-8 assay revealed the cell viability of J82 and TCCSUP cells stably transfected with scramble, sh-CLIC3#1, or sh-CLIC3#2, and those cotransfected with scramble or sh-p21. **C**–**E** Flow cytometry assay revealed the cell cycle distributions in J82 and TCCSUP cells stably transfected with scramble, sh-CLIC3#1, or sh-CLIC3#2, and those cotransfected with scramble or sh-p21. **F** Colony formation assay was performed in J82 and TCCSUP cells stably transfected with scramble, sh-CLIC3#1, or sh-CLIC3#2, and those cotransfected with scramble or sh-p21. Data are presented as the means ± SD from three independent experiments. ns, nonsignificant; **P < 0.01 (Student *t* test).

To confirm whether the effects of CLIC3 on cell proliferation were mediated via p21, we knocked down p21 in CLIC3-knockdown bladder cancer cells (Fig. 5A). As shown in Fig. 5B, the reduction of cell viability upon CLIC3 knockdown could be reversed by knockdown of p21. Meanwhile, inhibition of p21 expression rescued the G0/G1 cell cycle arrest (Fig. 5C–E), and attenuated the inhibition of colony formation ability upon knockdown of CLIC3 in bladder cancer cells (Fig. 5F). These findings suggested that knockdown of CLIC3 could promote p21 expression though increasing its stability, and the function of CLIC3 on cell proliferation was dependent on p21 expression.

### CLIC3 interacts with NAT10 protein

**Fig. 6:**
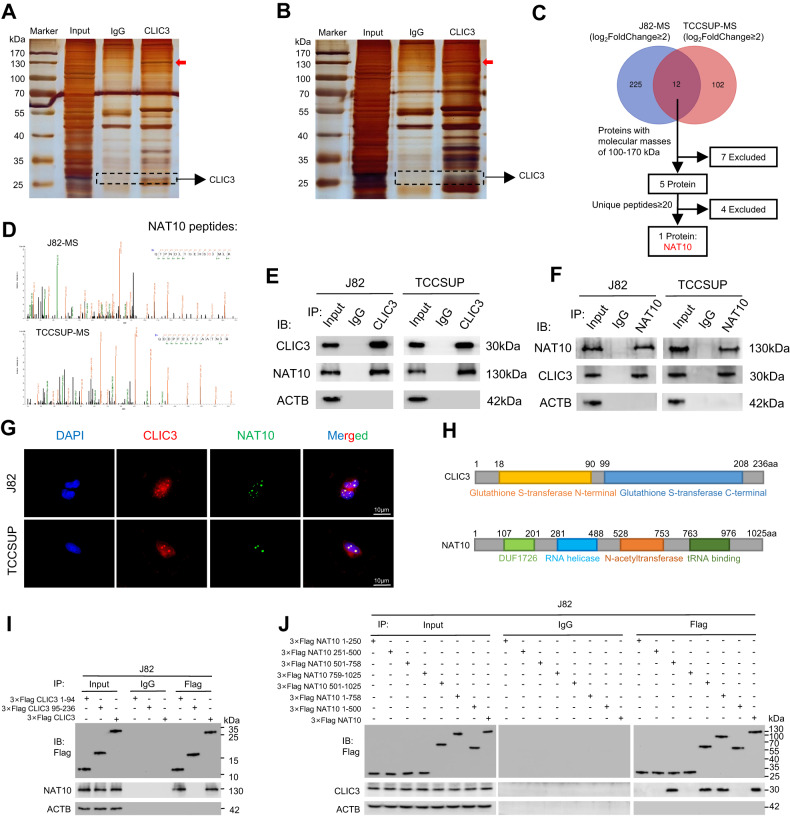
CLIC3 interacts with NAT10 protein. Silver staining showed the proteins pulled down by CLIC3 from the lysates of J82 (**A**) and TCCSUP (**B**) cells. Red arrow indicates the major differential band precipitated in J82 and TCCSUP lysates. **C** Venn diagram showed the overlapping of the differential proteins collection in J82 and TCCSUP cells of MS assay. Analysis pipeline was performed to identify proteins that interact with CLIC3: (1) The proteins with log2FC ≥ 2 in J82-MS or TCCSUP-MS were selected; (2) The 12 proteins were identified after overlapping; (3) The 5 proteins with molecular masses of 100–170 kDa were then selected as the candidates according to the positive band found in silver staining; (4) NAT10 was selected as it was the only protein with high abundance (no less than 20 unique peptides). **D** J82-MS and TCCSUP-MS each showed one of the NAT10 peptides pulled down by CLIC3, respectively. **E** Co-IP assay using antibody specific for CLIC3 showed the interaction between CLIC3 and NAT10 in J82 and TCCSUP cells. The precipitate was subjected to Western blotting with the antibodies against CLIC3, NAT10 and ACTB. **F** Co-IP assay using antibody specific for NAT10 showed the interaction between CLIC3 and NAT10 in J82 and TCCSUP cells. The precipitate was subjected to Western blotting with the antibodies against NAT10, CLIC3 and ACTB. **G** Immunofluorescence staining assay indicated the co-localization of CLIC3 (red) and NAT10 (green), with nuclei staining with DAPI (blue). Scale bar, 10 μm. **H** Schematic illustration of the functional domains of CLIC3 and NAT10 from SMART database (https://smart.embl.de/). **I** Co-IP assay using antibody specific for Flag showed the interaction between NAT10 and full-length or truncations of Flag-tagged recombinant CLIC3 in J82 cells. The precipitate was subjected to Western blotting with the antibodies against Flag, NAT10 and ACTB. **J** Co-IP assay using antibody specific for Flag showed the interaction between CLIC3 and full-length or truncations of Flag-tagged recombinant NAT10 in J82 cells. The precipitate was subjected to Western blotting with the antibodies against Flag, CLIC3 and ACTB.

We had shown that CLIC3 could reduce the stability of p21 mRNA. However, CLIC3 is not considered to be a common RNA-binding protein (RBP) [31]. Therefore, we speculated that CLIC3 could influence the stability of p21 mRNA through interacting with partners. To further verify our hypothesis, we performed Co-IP assay and identified potential interacting proteins (Fig. 6A, B). Following the analysis pipeline (Fig. 6C and Supplementary Table 4), NAT10 was identified as the major differential protein through mass spectrometry (Fig. 6D). The interaction between CLIC3 and NAT10 was confirmed by Co-IP assays (Fig. 6E, F), whereas CLIC3 and NAT10 did not affect the expression of each other (Fig. S4A, C). Meanwhile, there was no correlation between CLIC3 and NAT10 in bladder cancer tissues (Fig. S4D). Furthermore, CLIC3 and NAT10 were colocalized in the nuclear of bladder cancer cells (Fig. 6G). Moreover, knockdown of NAT10 promoted the proliferation of bladder cancer cells J82 and TCCSUP (Fig. S4E–H). However, previous studies report that NAT10 knockdown inhibits the viability of bladder cancer cells T24 and UMUC3 [32]. Consistently, we also verified that silence of NAT10 could inhibit cell growth of T24 and UMUC3 (Fig. S4I–M).

To delineate the structural determinants of the interaction between CLIC3 and NAT10, we subdivided the functional domains of CLIC3 and NAT10 based on the SMART database (Fig. 6H). Anti-Flag Co-IP assays showed that removal of 1–94 amino acids region of CLIC3 or 501–758 amino acids region of NAT10 abolished the interaction between CLIC3 and NAT10 (Fig. 6I, J), indicating that CLIC3 and NAT10 formed a protein-protein complex through the interaction between Gluta- thione S-transferase (GST) N-terminal domain of CLIC3 and N-acetyltransferase domain of NAT10 in bladder cancer cells.

### CLIC3/NAT10 complex regulates p21 mRNA stability by ac4C modification in bladder cancer cells

**Fig. 7:**
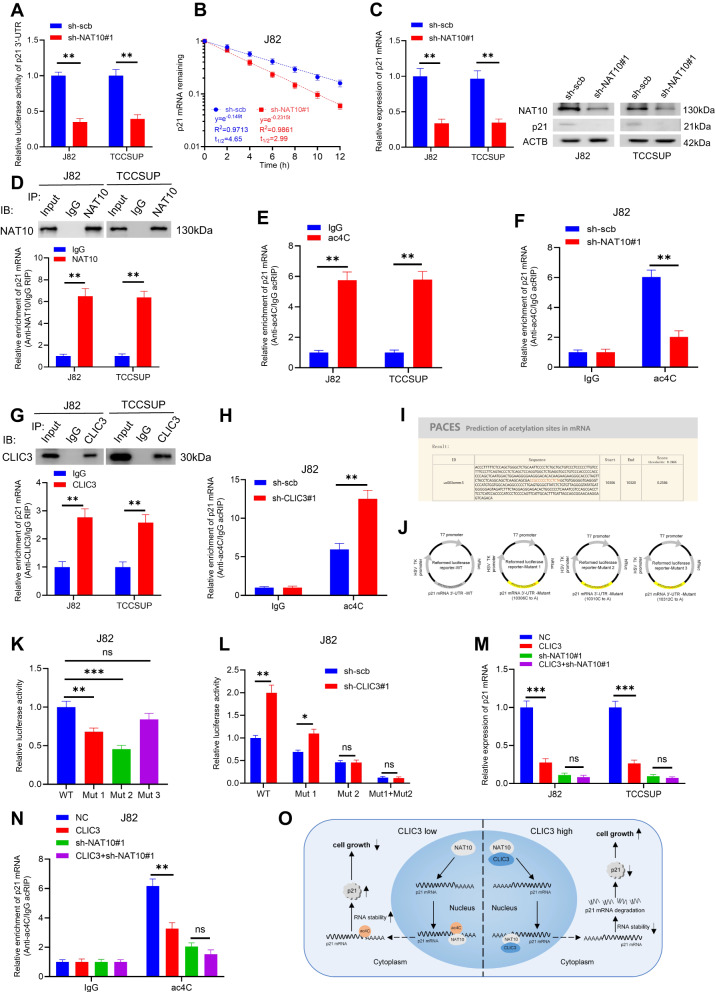
CLIC3/NAT10 complex mediates ac4C modification of p21 mRNA in bladder cancer cells. **A** The relative luciferase activity of p21 3′-UTR in J82 and TCCSUP cells stably transfected with scramble or sh-NAT10#1. **B** The relative remaining levels of p21 mRNA was analyzed by qRT-PCR after treatment with actinomycin D (5 μg/μl) at the indicated time points in J82 cells transfected with scramble or sh-NAT10#1. **C** The expression of p21 was detected by qRT-PCR (left) and Western blotting (right) in J82 and TCCSUP cells stably transfected with scramble or sh-NAT10#1. **D** RIP assays in J82 and TCCSUP cells using NAT10 and IgG antibody. The precipitate was subjected to Western blotting with the antibody against NAT10. The NAT10-enriched p21 mRNA relative to the IgG-enriched value was calculated by qRT-PCR. **E** ac4C-RIP assays in J82 and TCCSUP cells using ac4C and IgG antibody. The ac4C-enriched p21 mRNA relative to the IgG-enriched value was calculated by qRT-PCR. **F** ac4C-RIP assays in J82 cells stably transfected with scramble or sh-NAT10#1 using ac4C and IgG antibody. The ac4C-enriched p21 mRNA relative to the IgG-enriched value was calculated by qRT-PCR. **G** RIP assays in J82 and TCCSUP cells using CLIC3 and IgG antibody. The precipitate was subjected to western blotting with the antibodies against CLIC3. The CLIC3-enriched p21 mRNA relative to the IgG-enriched value was calculated by qRT-PCR. **H** ac4C-RIP assays in J82 cells stably transfected with scramble or sh-CLIC3#1 using ac4C and IgG antibody. The ac4C-enriched p21 mRNA relative to the IgG-enriched value was calculated by qRT-PCR. **I** PACES tools (http://rnanut.net/paces/) were used to predict the conserved acetylation sites in p21 3′-UTR. **J** Schematic illustration of the establishment of the four reformed luciferase reporter plasmids with wild-type, 10306 mutation (C to A), 10310 mutation (C to A) or 10312 mutation (C to A). **K** The relative luciferase activity of p21 3′-UTR in J82 cells transfected with reformed luciferase reporter plasmids WT, Mut1, Mut2, or Mut3. **L** The relative luciferase activity of p21 3′-UTR in J82 cells stably transfected with scramble or sh-CLIC3#1, and those cotransfected with reformed luciferase reporter plasmids WT, Mut1, Mut2, or Mut1+Mut2. **M** The expression of p21 was detected by qRT-PCR in J82 and TCCSUP cells transfected with vector or CLIC3, and those cotransfected with sh-scb or sh-NAT10#1. **N** ac4C-RIP assays in J82 cells stably transfected with vector or CLIC3, and those cotransfected with scramble or sh-NAT10#1, using ac4C and IgG antibody. The ac4C-enriched p21 mRNA relative to the IgG-enriched value was calculated by qRT-PCR. **O** Schematic of our hypothesis showing the effect of the CLIC3/NAT10 complex on bladder cancer proliferation. CLIC3 could bind to NAT10 in the nucleus, where it inhibited NAT10-mediated ac4C modification of p21 mRNA and decreased the stability of p21 mRNA, thereby promoting cell growth in bladder cancer. Data are presented as the means ± SD from three independent experiments. ns, nonsignificant; *P < 0.05; **P < 0.01; ***P < 0.001 (Student *t* test).

It is previously reported that NAT10-mediated ac4C modification of mRNA can promote its stability [22]. We found that knockdown of NAT10 could reduce the luciferase activity of p21 3′-UTR (Fig. 7A) and decreased p21 expression by reducing the stability of p21 mRNA (Fig. 7B, C). The interaction between NAT10 and p21 mRNA was further validated by RIP assays (Fig. 7D). Moreover, ac4C-RIP assays indicated that p21 mRNA could undergo ac4C modification (Fig. 7E), and NAT10 knockdown decreased the ac4C modification of p21 mRNA in bladder cancer cells (Fig. 7F and Fig. S5A). Given that CLIC3 could interact with NAT10, we wondered whether CLIC3 could bind to p21 mRNA. As expected, we confirmed that CLIC3 could bind to p21 mRNA (Fig. 7G). Of note, although knockdown of CLIC3 had no effect on the interaction between NAT10 and p21 mRNA (Fig. S5B), CLIC3 knockdown significantly promoted ac4C modification of p21 mRNA (Fig. 7H and Fig. S5C).

Subsequently, we predicted the putative ac4C sites in p21 mRNA by PACES with a specificity of 95% (Fig. 7I), and constructed three recombinant luciferase reporter plasmids with mutation sites (Cytosine to Adenine) (Fig. 7J) referring to the dbPTM database [33]. As shown in Fig. 7K and Fig. S5D, the mutation at 10306 or 10310 resulted in decreased luciferase activity of p21 3′-UTR but not the mutation at 10312, suggesting that Cytosines at 10306 and 10310 were the ac4C sites of p21 mRNA. Furthermore, the luciferase activity of p21 3′-UTR was still influenced by CLIC3 when only the Cytosine at 10306 was mutated, whereas CLIC3 knockdown had no effect on the luciferase activity of p21 3′-UTR with a mutated Cytosine at 10310 (Fig. 7L and Fig. S5E). These results indicated that Cytosine at 10310 of p21 mRNA was the potential site where CLIC3 regulated ac4C modification of p21 mRNA.

To investigate whether the effects of CLIC3 on ac4C modification of p21 mRNA were mediated via NAT10, we constructed a stable model of CLIC3 over-expressed in NAT10-knockdown bladder cancer cells. Overexpression of CLIC3 had a minor effect on p21 mRNA upon NAT10 knockdown (Fig. 7M). Furthermore, CLIC3-mediated inhibitory effect on ac4C modification of p21 mRNA also became minimal upon knockdown of NAT10 (Fig. 7N). In general, CLIC3 could interact with NAT10 in the nucleus, where it inhibited NAT10-mediated ac4C modification of p21 mRNA, and promote cell growth in bladder cancer (Fig. 7O).

## Discussion

With the progress of sequencing technology and bioinformatics, the gene regulatory landscape can be measured more accurately and intuitively, which provides strong evidence to screen for key genes in tumor progression [5]. Quantitative analysis of chromatin accessibility to gene expression has elucidated the gene regulatory networks of various cancers [34–36], further identifying previously unrecognized molecular predictors of treatment response and cell subpopulations with durable therapeutic potential. Previously, we integrated chromatin accessibility data of ATAC-seq and transcription factor binding information to assess TFCRs and identify candidate oncogenes, obtaining 13 candidate key genes in endometrial cancer [29]. Nevertheless, the application of TFCR identification method in bladder cancer remains to be elucidated. Herein, based on the reports that ATAC-seq for mapping chromatin accessibility genome wide is highly correlated with DNase-seq assays [37], we applied the improved TFCR identification method to ATAC-seq data and DNase-seq data, and obtained 42,742 gain-TFCRs in bladder cancer tissues, which is likely to be associated with the development of cancer. Notably, CLIC3 was identified as a key gene in bladder cancer based on the correlation between gene expression and overall survival. Our findings implies that improved TFCR identification can provide a novel insight into gene regulatory landscape and help us screen for key genes in bladder cancer.

CLIC3 is initially reported as a member of CLIC family, which has integral membrane forms to regulate intracellular membranes maintenance and tubulogenesis [11]. CLIC3 also acts as a secreted protein that drives angiogenesis and invasiveness of cancer [14]. Besides, previous studies report that CLIC3 colocalizes with Rab25 and integrin in late endosomes/lysosomes to promote the migration and invasion of pancreatic ductal adenocarcinoma [15]. However, we demonstrated that CLIC3, mainly located in the nucleus, could interact with NAT10, but not with Rab25, to promote cell proliferation in bladder cancer. Thus, we speculate that diverse features of CLIC3 may be attributed to its differential subcellular localization and interactions with divergent partners. Nevertheless, more in-depth research is still needed to investigate the role of CLIC3 in regulating cell metastasis and the sensitivity of therapy in bladder cancer.

There are presently conflicting reports as to whether NAT10 functions primarily as a promoter or suppressor of cancer. NAT10 promotes the progression of gastric cancer [18] and pancreatic cancer [38]. Conversely, NAT10 inhibits cell proliferation in colon cancer [23]. Consistent with the previous study [32], we verified that NAT10 knockdown inhibited the viability of bladder cancer cells UMUC3 and T24. However, we also observed that knockdown of NAT10 promoted the proliferation of bladder cancer cells J82 and TCCSUP. These two seemingly divergent biological results may be determined by specific biological circumstances. As previously reported that Rab25 acts as a tumor suppressor in the absence of CLIC3, whereas Rab25 increases tumor aggressiveness after driving CLIC3 expression [15]. Importantly, CLIC3 expressed at a high level in J82 and TCCSUP compared with other bladder cancer cells, including UMUC3 and T24. Therefore, we speculate that in tumors where CLIC3 is highly expressed, NAT10 will likely act as a tumor suppressor through interacting with CLIC3. Moreover, it is of great significance to consider CLIC3 expression in tumor when targeting NAT10 for therapeutic purposes. Of note, we demonstrated that the GST N-terminal domain of CLIC3 could bind the N-acetyltransferase domain of NAT10, which indicates that CLIC3 may act as a molecular chaperone and possibly alter the spatial conformation of NAT10, thereby changing the role of NAT10 in tumor progression. However, the topological structure of CLIC3/NAT10 complex still needs to be further characterized, which may reveal detailed features of this interaction and unveil whether CLIC3 plays an important role in the conformational change of NAT10.

The ac4C is a novel mRNA modification that catalyzed by the acetyltransferase NAT10. Transcriptome-wide mapping of ac4C indicates that acetylated regions are mainly enriched within coding sequences (CDS), followed by 5′-UTR and 3′-UTR [22]. Intriguingly, ac4C within CDS stimulates mRNA translation, while 5′-UTR acetylation inhibits annotated start codons and contributes to the reduction of protein synthesis [39]. Additionally, NAT10 could increase the expression of KIF23 by up-regulating ac4C modification of KIF23 3′-UTR in colorectal cancer [40]. Similarly, we validated that NAT10 could catalyze ac4C modification within 3′-UTR of p21 mRNA, thereby increasing its stability and expression. Furthermore, we demonstrated that CLIC3 could inhibit NAT10-catalyzed ac4C modification, but not the binding of NAT10 to p21 mRNA, which may be associated with the interaction between CLIC3 and the N-acetyltransferase domain of NAT10. Notably, CLIC3 could inhibit the acetylation of Cytosine at 10310, but not Cytosine at 10306 of p21 3′-UTR. Although it is unclear how CLIC3 specifically catalyzes the acetylation site of p21 mRNA, these results highlight that CLIC3 can act as a regulatory factor of NAT10, enriching the current mechanisms of mRNA ac4C modification.

In summary, our work first identified TFCRs in bladder cancer by integrating chromatin accessibility data of ATAC-seq and DNase-seq with TF binding information, and provided a proof of concept for CLIC3 as a regulatory factor of NAT10 protein and of key cellular functions relevant to mRNA ac4C modification. Remarkably, our study reveals that CLIC3 may act as a potential therapeutic target for curative management of bladder cancer.

### Supplementary information

Supplementary information file

Authorship Change Approval

Supplementary Figures

## Data Availability

All data needed to evaluate the conclusions in the paper are present in the paper and the Supplementary Materials. RNA Sequencing results were deposited in the Gene Expression Omnibus database The following secure token has been created to allow review of record GSE232965 while it remains in private status: mfmxikyuzzqzxol.

## References

[1] Siegel RL, Miller KD, Fuchs HE, Jemal A (2022). Cancer statistics, 2022. CA Cancer J Clin.

[2] Humphrey PA, Moch H, Cubilla AL, Ulbright TM, Reuter VE (2016). The 2016 WHO classification of tumours of the urinary system and male genital organs-Part B: prostate and bladder tumours. Eur Urol.

[3] Zhang Z, Dong Pei, Li Y, Liu Z, Yao K, Han H (2014). Radical cystectomy for bladder cancer: oncologic outcome in 271 Chinese patients. Chin J Cancer.

[4] Becker PB, Workman JL (2013). Nucleosome remodeling and epigenetics. Cold Spring Harb Perspect Biol.

[5] Klemm SL, Shipony Z, Greenleaf WJ (2019). Chromatin accessibility and the regulatory epigenome. Nat Rev Genet.

[6] Carter B, Zhao K (2021). The epigenetic basis of cellular heterogeneity. Nat Rev Genet.

[7] Buenrostro JD, Wu B, Chang HY, Greenleaf WJ (2015). ATAC-seq: a method for assaying chromatin accessibility genome-wide. Curr Protoc Mol Biol.

[8] Corces MR, Trevino AE, Hamilton EG, Greenside PG, Sinnott-Armstrong NA, Vesuna S (2017). An improved ATAC-seq protocol reduces background and enables interrogation of frozen tissues. Nat Methods.

[9] Chen H, Li H, Liu F, Zheng X, Wang S, Bo X (2015). An integrative analysis of TFBS-clustered regions reveals new transcriptional regulation models on the accessible chromatin landscape. Sci Rep.

[10] Lele Jiang JMP, Yu J, Harrop SJ, Sokolova AV, Duff AP (2014). CLIC proteins, ezrin, radixin, moesin and the coupling of membranes to the actin cytoskeleton: a smoking gun?. Biochim Biophys Acta.

[11] Littler DR, Harrop SJ, Goodchild SC, Phang JM, Mynott AV, Jiang L (2010). The enigma of the CLIC proteins: Ion channels, redox proteins, enzymes, scaffolding proteins?. FEBS Lett.

[12] Rao SG, Patel NJ, Singh H (2020). Intracellular chloride channels: novel biomarkers in diseases. Front Physiol.

[13] Ozaki S, Mikami K, Kunieda T, Tanaka J (2022). Chloride intracellular channel proteins (CLICs) and malignant tumor progression: a focus on the preventive role of CLIC2 in invasion and metastasis. Cancers.

[14] Hernandez-Fernaud JR, Ruengeler E, Casazza A, Neilson LJ, Pulleine E, Santi A (2017). Secreted CLIC3 drives cancer progression through its glutathione-dependent oxidoreductase activity. Nat Commun.

[15] Dozynkiewicz MA, Jamieson NB, Macpherson I, Grindlay J, Berghe PVEVD, Thun AV (2012). Rab25 and CLIC3 collaborate to promote integrin recycling from late endosomes/lysosomes and drive cancer progression. Dev Cell.

[16] Chen M, Zhang S, Wen X, Cao H, Gao Y (2020). Prognostic value of CLIC3 mRNA overexpression in bladder cancer. PeerJ.

[17] Arango D, Sturgill D, Alhusaini N, Dillman AA, Sweet TJ, Hanson G (2018). Acetylation of cytidine in mRNA promotes translation efficiency. Cell.

[18] Zhang Y, Jing Y, Wang Y, Tang J, Zhu X, Jin W (2021). NAT10 promotes gastric cancer metastasis via N4-acetylated COL5A1. Signal Transduct Target Ther.

[19] Yu X, Li S, Yao Z, Xu J, Zheng C, Liu Z (2023). N4-acetylcytidine modification of lncRNA CTC-490G23.2 promotes cancer metastasis through interacting with PTBP1 to increase CD44 alternative splicing. Oncogene.

[20] Zheng X, Wang Q, Zhou Y, Zhang D, Geng Y, Hu W (2022). N-acetyltransferase 10 promotes colon cancer progression by inhibiting ferroptosis through N4-acetylation and stabilization of ferroptosis suppressor protein 1 (FSP1) mRNA. Cancer Commun.

[21] Sharma S, Langhendries J-L, Watzinger P, Kötter P, Entian K-D, Lafontaine DLJ (2015). Yeast Kre33 and human NAT10 are conserved 18S rRNA cytosine acetyltransferases that modify tRNAs assisted by the adaptor Tan1/THUMPD1. Nucleic Acids Res.

[22] Dominissini D, Rechavi G (2018). N4-acetylation of cytidine in mRNA by NAT10 regulates stability and translation. Cell.

[23] Liu X, Tan Y, Zhang C, Zhang Y, Zhang L, Ren P (2016). NAT10 regulates p53 activation through acetylating p53 at K120 and ubiquitinating Mdm2. EMBO Rep.

[24] Wu X, Liu D, Tao D, Xiang W, Xiao X, Wang M (2016). BRD4 regulates EZH2 transcription through upregulation of C-MYC and represents a novel therapeutic target in bladder cancer. Mol Cancer Ther.

[25] Zhang H, Xiao X, Wei W, Huang C, Wang M, Wang L (2021). CircLIFR synergizes with MSH2 to attenuate chemoresistance via MutSα/ATM-p73 axis in bladder cancer. Mol Cancer.

[26] Corces MR, Granja JM, Shams S, Louie BH, Seoane JA, Zhou W (2018). The chromatin accessibility landscape of primary human cancers. Science.

[27] Consortium EP (2012). An integrated encyclopedia of DNA elements in the human genome. Nature.

[28] Weirauch MT, Yang A, Albu M, Cote AG, Montenegro-Montero A, Drewe P (2014). Determination and inference of eukaryotic transcription factor sequence specificity. Cell.

[29] Tang X, Wang J, Tao H, Yuan L, Du G, Ding Y (2022). Regulatory patterns analysis of transcription factor binding site clustered regions and identification of key genes in endometrial cancer. Comput Struct Biotechnol J.

[30] Turajlic S, Sottoriva A, Graham T, Swanton C (2019). Resolving genetic heterogeneity in cancer. Nat Rev Genet.

[31] Carbon S, Ireland A, Mungall CJ, Shu S, Marshall B, Lewis S (2009). AmiGO: online access to ontology and annotation data. Bioinformatics.

[32] Xie R, Cheng L, Huang M, Huang L, Chen Z, Zhang Q (2023). NAT10 drives cisplatin chemoresistance by enhancing ac4C-associated DNA repair in bladder cancer. Cancer Res.

[33] Li Z, Li S, Luo M, Jhong J-H, Li W, Yao L (2022). dbPTM in 2022: an updated database for exploring regulatory networks and functional associations of protein post-translational modifications. Nucleic Acids Res.

[34] Regner MJ, Wisniewska K, Garcia-Recio S, Thennavan A, Mendez-Giraldez R, Malladi VS (2021). A multi-omic single-cell landscape of human gynecologic malignancies. Mol Cell.

[35] Shi X, Li Y, Yuan Q, Tang S, Guo S, Zhang Y (2022). Integrated profiling of human pancreatic cancer organoids reveals chromatin accessibility features associated with drug sensitivity. Nat Commun.

[36] Taavitsainen S, Engedal N, Cao S, Handle F, Erickson A, Prekovic S (2021). Single-cell ATAC and RNA sequencing reveal pre-existing and persistent cells associated with prostate cancer relapse. Nat Commun.

[37] Buenrostro JD, Giresi PG, Zaba LC, Chang HY, Greenleaf WJ (2013). Transposition of native chromatin for fast and sensitive epigenomic profiling of open chromatin, DNA-binding proteins and nucleosome position. Nat Methods.

[38] Feng Z, Li K, Qin K, Liang J, Shi M, Ma Y (2022). The LINC00623/NAT10 signaling axis promotes pancreatic cancer progression by remodeling ac4C modification of mRNA. J Hematol Oncol.

[39] Arango D, Sturgill D, Yang R, Kanai T, Bauer P, Roy J (2022). Direct epitranscriptomic regulation of mammalian translation initiation through N4-acetylcytidine. Mol Cell.

[40] Jin C, Wang T, Zhang D, Yang P, Zhang C, Peng W (2023). Acetyltransferase NAT10 regulates the Wnt/β-catenin signaling pathway to promote colorectal cancer progression via ac4C acetylation of KIF23 mRNA. J Exp Clin Cancer Res.

