# Supplementary material for: CLIC3 interacts with NAT10 to inhibit N4-acetylcytidine modification of p21 mRNA and promote bladder cancer progression
Source: Cell Death Dis. 2024 Jan 5;15(1):9. doi: 10.1038/s41419-023-06373-z (PMC10770081; doi:10.1038/s41419-023-06373-z)
Supplement: Supplementary file 1 — Supplementary information file [file 41419_2023_6373_MOESM1_ESM.pdf]

Fig. S1

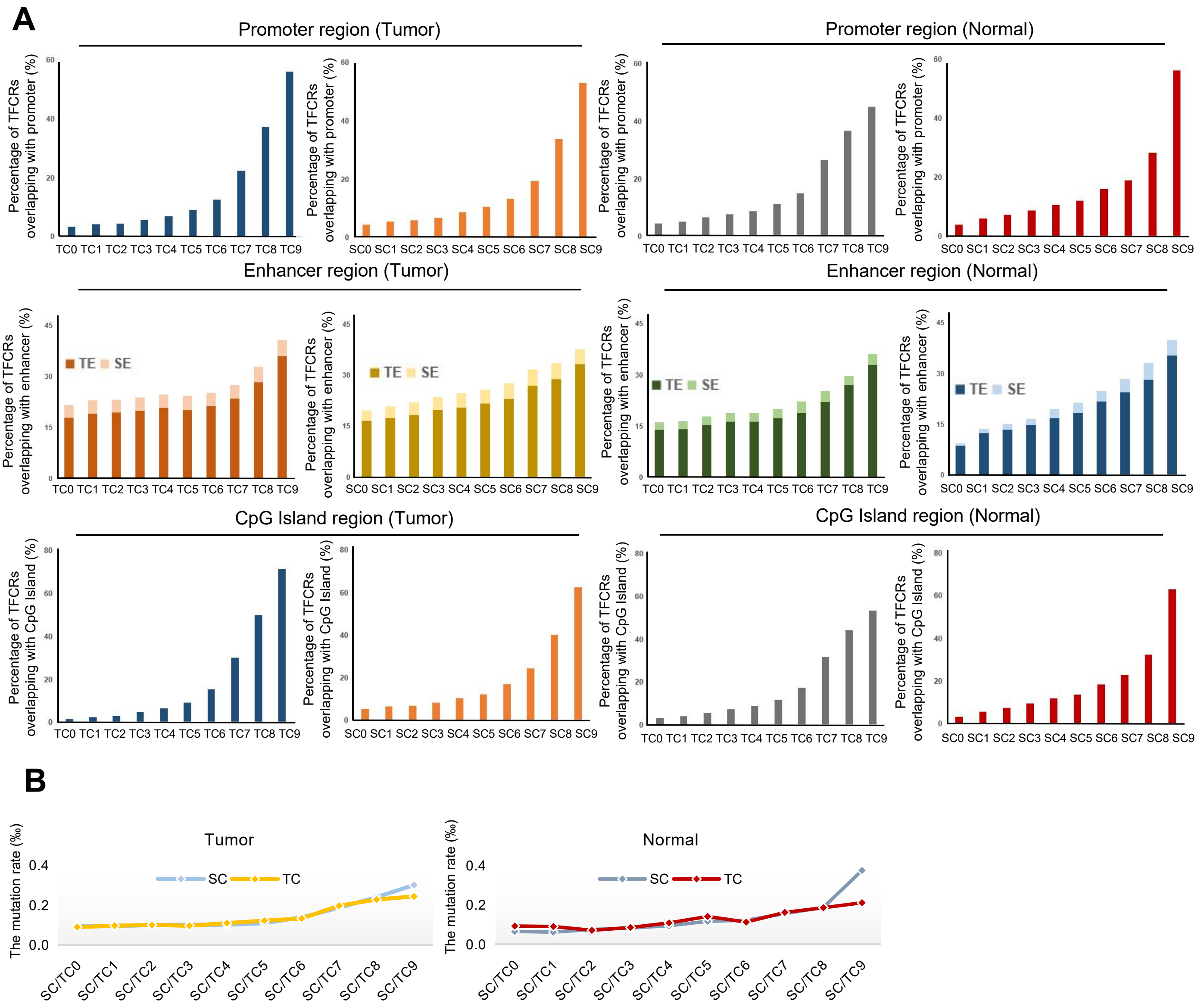

Supplementary Fig. 1 Identification of TFCRs to screen for key genes in bladder cancer progression.

(A) Barplot showed the proportion of TFCRs located in gene promoters, enhancers or CpG Islands (TE: typical enhancer; SE: super enhancer).

(B) Line graph showed the mutation rate in normal TFCRs and tumor TFCRs.

Fig. S2

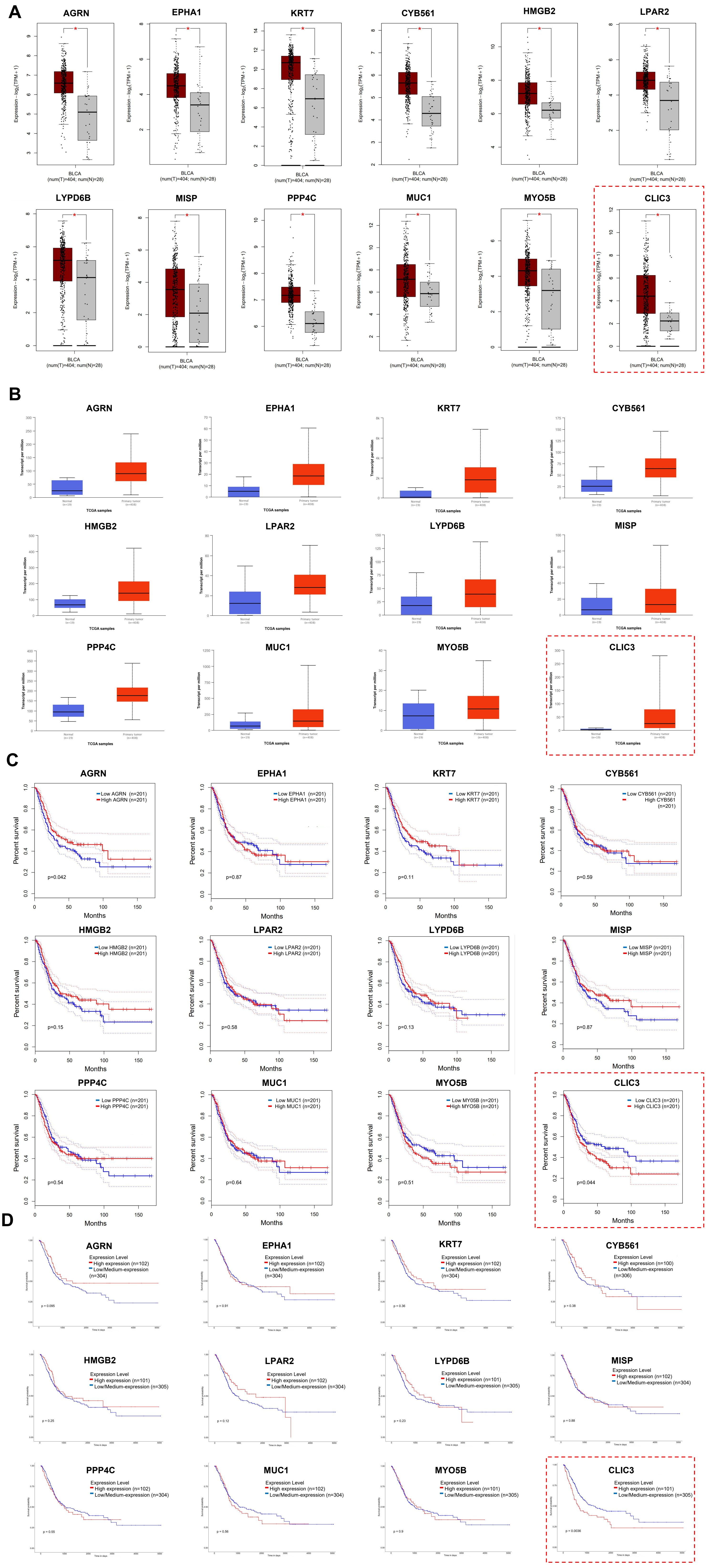

Supplementary Fig. 2 Identification and distribution of CLIC3.

(A) The expression levels of candidate genes in bladder cancer tissues compared with normal bladder tissues obtained from http://gepia.cancer-pku.cn/.

(B) The expression levels of candidate genes in bladder cancer tissues compared with normal bladder tissues obtained from https://ualcan.path.uab.edu/.

(C) Kaplan–Meier curves of overall survival in bladder cancer patients with low versus high expression of candidate genes from http://gepia.cancer-pku.cn/.

(D) Kaplan–Meier curves of overall survival in bladder cancer patients with low versus high expression of candidate genes http://ualcan.path.uab.edu/.

Fig. S3

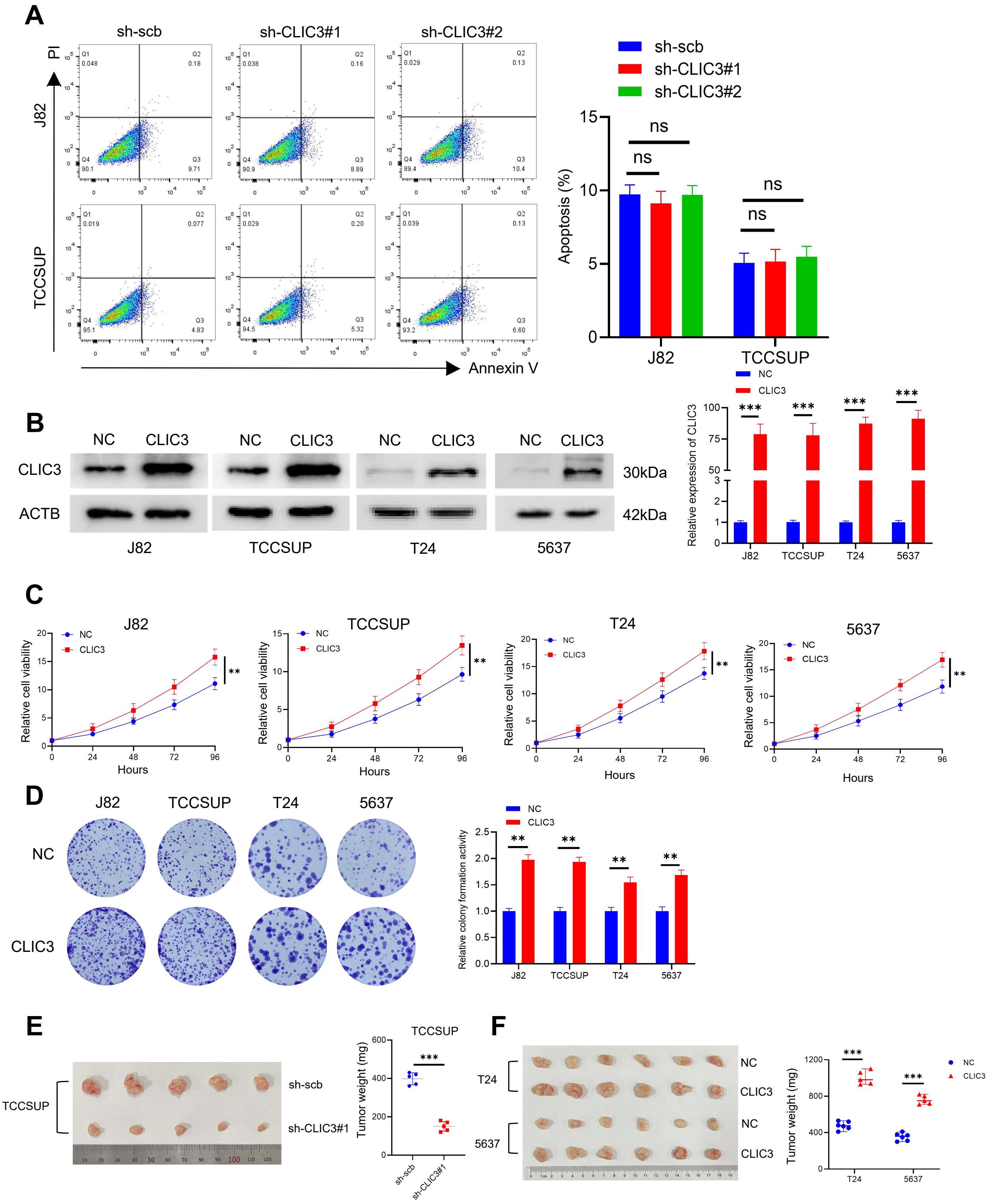

Supplementary Fig. 3 CLIC3 exerts pro-carcinogenic roles in bladder cancer.

(**A**) Flow cytometry assay revealed the rate of apoptosis in J82 and TCCSUP cells stably transfected with scramble, sh-CLIC3#1, or sh-CLIC3#2.

(B) The efficiency of CLIC3-overexpression in multiple bladder cancer cells was detected by Western blotting (left) and qRT-PCR (right).

(C) CCK-8 assay revealed the viability of multiple bladder cancer cells stably transfected with vector or CLIC3.

(D) Colony formation assay was performed in multiple bladder cancer cells stably transfected with vector or CLIC3.

(E) Representative and weight at the endpoints of xenograft tumors formed by subcutaneous injection of TCCSUP cells stably transfected with scramble or sh-CLIC3#1 into the right flanks of nude mice (5×10^6 cells per mouse; n = 5 for each group).

(F) Representative and weight at the endpoints of xenograft tumors formed by subcutaneous injection of T24 and 5637 cells stably transfected with vector or CLIC3 into the right flanks of nude mice (5×10^6 cells per mouse; n = 6 for each group).

Data are presented as the means ± SD from three independent experiments. ns, nonsignificant; **, $P < 0.01$; ***, $P < 0.001$ (Student t test).

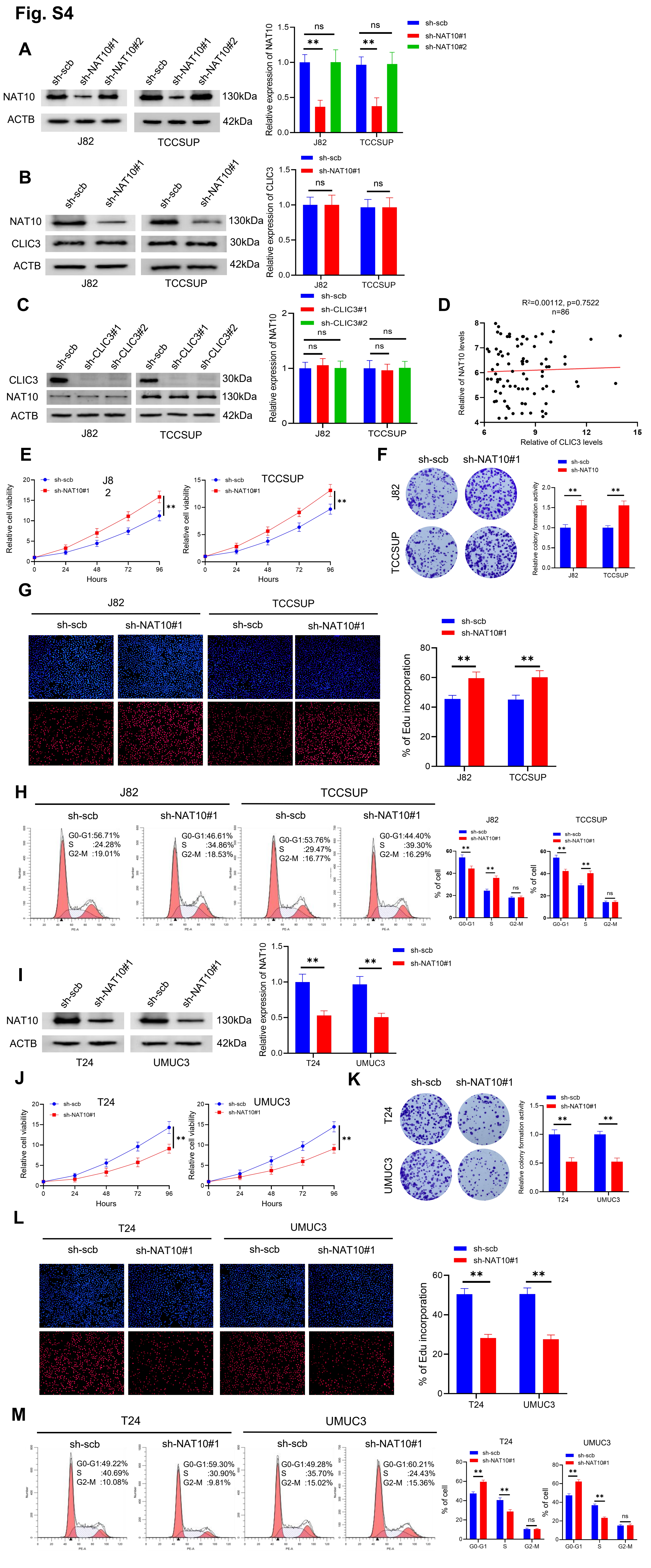

Supplementary Fig. 4 The effect of NAT10 knockdown on bladder cancer cells.

(A) The efficiency of NAT10 knockdown in J82 and TCCSUP cells was detected by Western blotting (left) and qRT-PCR (right).

(B) The expression of CLIC3 was detected by Western blotting (left) and qRT-PCR (right) in J82 and TCCSUP cells stably transfected with scramble or sh-NAT10#1.

(C) The expression of NAT10 was detected by Western blotting (left) and qRT-PCR (right) in J82 and TCCSUP cells stably transfected with scramble, sh-CLIC3#1, or sh-CLIC3#2.

(D) Analysis of the correlation between the transcript levels of CLIC3 and NAT10 in the tumor tissues of the 86 bladder cancer patients. The relative expression of CLIC3 and NAT10 was calculated by ΔCt value. P-values were calculated by Pearson correlation analysis.

(E) CCK-8 assay revealed the cell viability of J82 and TCCSUP cells stably transfected with scramble or sh-NAT10#1.

(F) Colony formation assay was performed in J82 and TCCSUP cells stably transfected with scramble or sh-NAT10#1.

(G) EdU assay showed the proliferation of J82 and TCCSUP cells stably transfected with scramble or sh-NAT10#1.

(H) Flow cytometry assay revealed the cell cycle distributions in J82 and TCCSUP cells stably transfected with scramble or sh-NAT10#1.

(I) The efficiency of NAT10 knockdown in T24 and UMUC3 cells was detected by Western blotting (left) and qRT-PCR (right).

(J) CCK-8 assay revealed the cell viability of T24 and UMUC3 cells stably transfected with scramble or sh-NAT10#1.

(K) Colony formation assay was performed in T24 and UMUC3 cells stably transfected with scramble or sh-NAT10#1.

(L) EdU assay showed the proliferation of T24 and UMUC3 cells stably transfected with scramble or sh-NAT10#1.

(M) Flow cytometry assay revealed the cell cycle distributions in T24 and UMUC3 cells stably transfected with scramble or sh-NAT10#1.

Data are presented as the means ± SD from three independent experiments. ns, nonsignificant; **, P < 0.01 (Student t test).

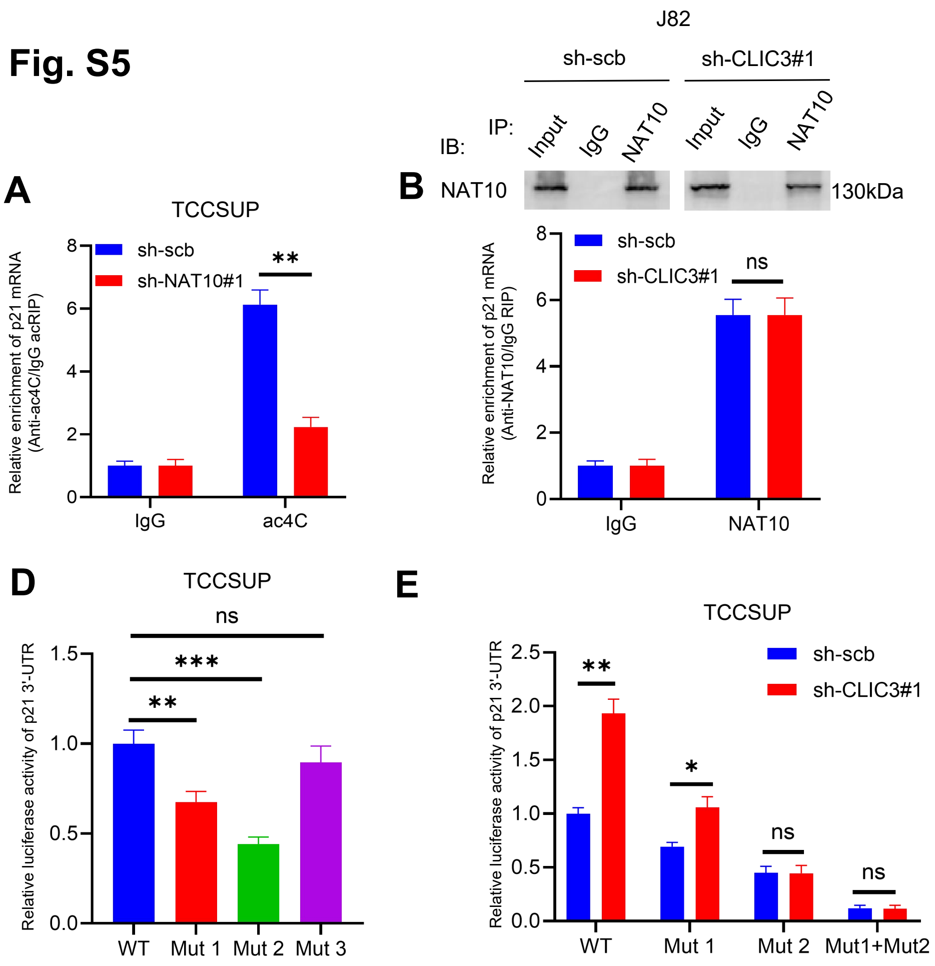

Supplementary Fig. 5 CLIC3/NAT10 complex mediates ac4C modification of p21 mRNA in bladder cancer cells.

(A) ac4C RIP assays in TCCSUP cells stably transfected with scramble or sh-NAT10#1 using ac4C and IgG antibody. The ac4C-enriched p21 mRNA relative to the IgG-enriched value was calculated by qRT-PCR.

(B) RIP assays in TCCSUP cells stably transfected with scramble or sh-CLIC3#1 using NAT10 and IgG antibody. The precipitate was subjected to western blotting with the antibody against NAT10. The NAT10-enriched p21 mRNA relative to the IgG-enriched value was calculated by qRT-PCR.

(C) ac4C RIP assays in TCCSUP cells stably transfected with scramble or sh-CLIC3#1 using ac4C and IgG antibody. The ac4C-enriched p21 mRNA relative to the IgG-enriched value was calculated by qRT-PCR.

(D) The relative luciferase activity of p21 3'-UTR in TCCSUP cells transfected with reformed luciferase reporter plasmids WT, Mut1, Mut2 or Mut3.

(E) The relative luciferase activity of p21 3'-UTR in TCCSUP cells stably transfected with scramble or sh-CLIC3#1, and those cotransfected with reformed luciferase reporter plasmids WT, Mut1, Mut2 or Mut1+Mut2.

Data are presented as the means ± SD from three independent experiments. ns, nonsignificant; *, $P < 0.05$; **, $P < 0.01$; ***, $P < 0.001$ (Student t test).

Supplementary table 1 Candidate genes screened based on the identification of TFCR.

TC9-SC9 Genes (95)		FPKM≥5 (53)
AC003688.1	LPAR2	ADAM15
AC118754.1	LRP5L	AGRN
ACTL10	LYPD6B	AKAP17A
ADAM15	MAFK	ANXA5
AGRN	MAP3K2	ARPC1B
AKAP17A	MBP	ASMTL
AL591806.3	MISP	BCL3
ANXA5	MOCOS	BOLA1
ARHGEF3	MPP5	CDC42BPG
ARPC1B	MROH1	CELSR1
ASAP2	MUC1	CLDN7
ASMTL	MYO5A	CLIC3
BAHCC1	MYO5B	CTDSP2
BCL3	NBPF11	CTDSPL
BICDL2	NR2C2	CYB561
BOLA1	NXN	DEAF1
CDC42BPG	ONECUT1	DENND2D
CELSR1	OTX1	DENND4B
CLDN7	PFKL	EPHA1
CLIC3	PKM	F11R
CPSF4L	PLCG1	FOS
CTDSP2	PLEKHF1	GDPD3
CTDSPL	PPP4C	HIST2H2AC
CYB561	PTPN18	HIST2H2BE
DEAF1	RARG	HMGB2
DENND2D	RFFL	IRF2BP2
DENND4B	RPS6KA4	KRT15
DOCK5	SHROOM1	KRT7
DUSP14	SLC16A6	LPAR2
ENTPD2	SLC25A6	LYPD6B
EPHA1	SLC6A11	MAFK
EPOP	SMAD6	MAP3K2
F11R	SSH3	MISP
FAM131A	TEAD3	MROH1
FHDC1	TNRC6A	MUC1
FOS	TPPP	MYO5B
GDAP1	TPTE	NXN
GDPD3	TRIM65	PFKL
GRAMD2A	TSPO	PKM
HGH1	TSTD1	PLCG1
HIST2H2AB	TWF2	PLEKHF1
HIST2H2AC	UGT2A3	PPP4C
HIST2H2BE		PTPN18
HMGB2		RARG
HOXA7		RPS6KA4
HRH1		SHROOM1
HS1BP3		SLC25A6
IRF2BP2		SSH3
JPH1		TEAD3
KIAA1671		TRIM65
KRT15		TSPO
KRT7		TSTD1
LEAP2		TWF2

Supplementary table 2 Clinicopathological features of 86 bladder cancer patients and the expressions of CLIC3.

Parameters	group	cases	CLIC3 mRNA expression		P-value
			High	Low	
Gender	Male	71	37	34	0.7014
	Female	15	7	8	
Age at surgery	＜60	23	10	13	0.4649
	≥60	63	33	30	
Pathological stage	Ta-T1	22	7	15	**0.0057**
	T2-T4	64	42	22	
Lymph node metastasis	Absent	75	40	35	0.3464
	Present	11	4	7	
Vascular invasion	Absent	79	41	38	0.4331
	Present	7	2	5	
Muscle invasion	NMIBC	27	6	15	**0.0122**
	MINC	59	39	26	
Total		86			

$p < 0.05$ represents statistical significance (Fisher’s exact test or Chi-square test)

Supplementary table 3 The sequences of primers and shRNA oligo sequences used in this study.

Primers for qPCR

Gene	Primer	Sequence (5′-3′)
CLIC3	Forward	GCTGCCCATCCTGCTCTATGA
	Reverse	TGCTGGTACAGGGCTTCGTC
ACTB	Forward	TGGCACCCAGCACAATGAA
	Reverse	CTAAGTCATAGTCCGCCTAGAAGCA
p21	Forward	GGCAGACCAGCATGACAGATTTC
	Reverse	AGATGTAGAGCGGGCCTTTGAG
NAT10	Forward	AGCTCAACATCCTGCCCATCTC
	Reverse	AAGACAGCTTTGGCCTGGTCT

Primers for plasmid construction

Vector	Primer	Sequence (5′-3′)
3xFlag CLIC3	Forward	CCGGAATTCGCCACCATGGCGGAGACCAAGCTCCA
	Reverse	CGGGGTACCGCGGGGGTGCACGGCGGGCCGGTA
3xFlag CLIC3 1-94	Forward	CCGGAATTCGCCACCATGGCGGAGACCAAGCTCCA
	Reverse	CGGGGTACCCAGGCTGGGGAAGTCGGGCGGCCCC
3xFlag CLIC3 95-236	Forward	CCGGAATTCGCCACCGCGCCTCGTTACAGGGAGTC
	Reverse	CGGGGTACCGCGGGGGTGCACGGCGGGCCGGTA
3xFlag NAT10	Forward	CCGGAATTCGCCACCATGCATCGGAAAAAGGTGGAT
	Reverse	CGGGGTACCTTTCTTCCGCTTCAGTTTCATATCT
3xFlag NAT10 1-250	Forward	CCGGAATTCGCCACCATGCATCGGAAAAAGGTGGAT
	Reverse	CGGGGTACCCACACCCACAGGCTGGGTGTCCT
3xFlag NAT10 251-500	Forward	CCGGAATTCGCCACCTTGGTGGACTGCTGTAAGAC
	Reverse	CGGGGTACCGGGGCAGCCTGAGACTATCCGAGTG
3xFlag NAT10 501-758	Forward	CCGGAATTCGCCACCTTGCCTGAAGCTTGTGAACT
	Reverse	CGGGGTACCAGCCTCATCCTCATCAGTGAGCGTC
3xFlag NAT10 759-1025	Forward	CCGGAATTCGCCACCGACCAGGGAGGCTGGCTTGC
	Reverse	CGGGGTACCTTTCTTCCGCTTCAGTTTCATATCT
3×Flag NAT10 501-1025	Forward	CCGGAATTCGCCACCTTGCCTGAAGCTTGTGAACT
	Reverse	CGGGGTACCTTTCTTCCGCTTCAGTTTCATATCT
3×Flag NAT10 1-758	Forward	CCGGAATTCGCCACCATGCATCGGAAAAAGGTGGAT
	Reverse	CGGGGTACCAGCCTCATCCTCATCAGTGAGCGTC
3×Flag NAT10 1-500	Forward	CCGGAATTCGCCACCATGCATCGGAAAAAGGTGGAT
	Reverse	CGGGGTACCGGGGCAGCCTGAGACTATCCGAGTG
p21 mutant (10306C to 10306A)	Forward	GCTCAAGCAGCGAACGCCCCCTCCTCTAGCTGTG
	Reverse	GAGGAGGGGGCGTTCGCTGCTTGAGCTGCCTGAG
p21 mutant (10310C to 10310A)	Forward	CAAGCAGCGACCGCACCCTCCTCTAGCTGTGGG
	Reverse	GCTAGAGGAGGGTGCGGTCGCTGCTTGAGCTGCC
p21 mutant (10312C to 10312A)	Forward	GCAGCGACCGCCCACTCCTCTAGCTGTGGG
	Reverse	CAGCTAGAGGAGTGGGCGGTCGCTGCTTGAG
p21 mutant (10310C/10312C to 10310A/10312A)	Forward	TCAAGCAGCGAACGCACCCTCCTCTAGCTGTGGGGG
	Reverse	GCTAGAGGAGGGTGCGTTCGCTGCTTGAGCTGCCTGA

ShRNA oligo sequences

Vector	Primer	Sequence (5′-3′)
scramble	Forward	CCGGCAACAAGATGAAGAGCACCAACTCGAGTTGGTGCTCTTCATCTTGTTGTTTTTGGTACC
	Reverse	AATTGGTACCAAAAACAACAAGATGAAGAGCACCAACTCGAGTTGGTGCTCTTCATCTTGTTG
sh-CLIC3#1	Forward	CCGGGCCTCGTTACAGGGAGTCCAACTCGAGTTGGACTCCCTGTAACGAGGCTTTTTG
	Reverse	AATTCAAAAAGCCTCGTTACAGGGAGTCCAACTCGAGTTGGACTCCCTGTAACGAGGC
sh-CLIC3#2	Forward	CCGGGCTCTATGACAGCGACGCCAACTCGAGTTGGCGTCGCTGTCATAGAGCTTTTTG
	Reverse	AATTCAAAAAGCTCTATGACAGCGACGCCAACTCGAGTTGGCGTCGCTGTCATAGAGC
sh-p21	Forward	CCGGGCTGATCTTCTCCAAGAGGAACTCGAGTTCCTCTTGGAGAAGATCAGCTTTTTG
	Reverse	AATTCAAAAAGCTGATCTTCTCCAAGAGGAACTCGAGTTCCTCTTGGAGAAGATCAGC
sh-NAT10#1	Forward	CCGGGCAATTGTACACAGTGACTATCTCGAGATAGTCACTGTGTACAATTGCTTTTTG
	Reverse	AATTCAAAAAGCAATTGTACACAGTGACTATCTCGAGATAGTCACTGTGTACAATTGC
sh-NAT10#2	Forward	CCGGCCAGTCTCTAAATCCTGAATTCTCGAGAATTCAGGATTTAGAGACTGGTTTTTG
	Reverse	AATTCAAAAACCAGTCTCTAAATCCTGAATTCTCGAGAATTCAGGATTTAGAGACTGG

Supplementary table 4 Cell growth- and cell cycle-related genes from AmiGO2 database and the differential genes detected by RNA-seq.

cell cycle (1288)			cell growth (278)	p value top 500 (500)	|log2FoldChange|≥4 (2749)					
ZNF324	NUP43	CFL1	L1CAM	FSTL1	MT1E	C2orf81	RCOR2	ANKRD20A5P	MAP2	AC008147.2
ZFP42	NUP37	CETN3	SPP1	PSG1	PADI2	CBS	AL132657.1	PSG5	WNT16	AC135352.1
TP53BP2	NUP214	CETN2	SH3GL2	LIMA1	PAX2	AC013467.1	KIAA1257	NUP62CL	FGF7P1	UNC5B
WRN	NUMA1	CETN1	SEMA4C	COL18A1	PAX8	AL356124.1	TTYH2	LAYN	KIF21B	PAPSS2
TRIM21	NUF2	CEP85	EI24	OXTR	WSCD1	HM13-AS1	EPB41L4B	ZNF439	TSPAN7	ELMOD1
SNX18	NUDT15	CEP72	FLRT3	OLR1	STMN3	RPL29P14	FAM47B	CMPK2	ANXA8	TNFRSF9
ZWILCH	NUDC	CEP68	WT1	RHOB	VCAN	AC005009.2	AC010618.2	PDGFD	PARM1	SBF2-AS1
TUBGCP6	NTMT1	CEP63	ARHGEF11	COL5A2	HAVCR1	AC118754.1	FAM27E3	THSD7B	MGAT3	CMYA5
USP29	NSUN2	CEP55	SEMA3F	F3	TM4SF18	TRABD2B	AL021707.8	THNSL2	PRKG1	FAM167A
SMC1B	NSMCE2	CEP44	ST7L	GBP4	CASC19	C3orf18	SLC3A1	ARMC4	LOXL1-AS1	ELAVL2
ZNF207	NSL1	CEP295	SPART	SLC12A8	COMT	LINC02328	AC096720.1	OVCH2	AL445649.1	DERL3
SYCE1L	NSFL1C	CEP250	CPNE5	DIO2	HNF1B	AC027607.1	FLJ12825	NRXN3	KBTBD12	APCDD1L-DT
USP44	NRDE2	CEP192	IGFBP4	DUBR	CD70	AL049838.1	RNU5A-1	AL137003.2	KALRN	ANKRD34B
ZMYND11	NR3C1	CEP164	TGFB1	TM4SF1	LRRN4	ONECUT1	CRABP2	BEX4	HRASLS5	EPPK1
XPC	NPR2	CEP152	CDKN1A	HEG1	GALNT14	AC008619.1	AL627230.4	AC016582.3	KCNK1	FTLP3
WNT4	NPPC	CEP135	NOV	PSG9	OAT	KRT83	PLEKHG1	ACCS	OVCH1	SLAMF7
USP39	NPM1	CEP131	LTBP4	L1CAM	ALDH7A1	SMIM15-AS1	OR1F1	LZTS1	AP005264.1	CFTR
USP22	NPAT	CEP126	IGFBP7	PLSCR4	PADI3	NCKAP5	AC108134.4	ARHGAP28	BNIP3P29	AC138356.2
USP26	NOX5	CEP120	IGFBP3	C1S	SHH	SPINT2	AL031595.2	WNT5A	ACADL	ARHGAP23
VRK1	NOP53	CENPX	ZEB2	IFITM3	AFAP1L2	AC005208.1	AL354928.1	AC005392.2	AC022809.1	IFITM2
WTAP	NOLC1	CENPW	INHBA	GATA2	DMKN	VEGFD	MIR4326	TRIM22	ERC2	ST3GAL1
ZNF830	NLRP5	CENPV	SDCBP	IGFBP4	CPNE8	AL022328.1	CARD10	KCNQ1OT1	GRIP2	SLC1A1
UVRAG	NLE1	CENPT	PTK2B	DOCK4	GPRC5C	SLITRK4	ACSBG2	TMC5	HEG1	SOWAHD
ZBTB17	NIPBL	CENPS	BST2	PSG5	ELFN2	IRS4	CCNI2	GALNT13	ZNF747	SLC44A2
WBP2NL	NIN	CENPQ	NRP2	GRAMD2B	FOXC2	SVEP1	CLDN6	AP005212.4	NDN	JAKMIP3
WDR6	NEUROG1	CENPN	PLXNA1	SPRY4	KCNJ16	AC068700.1	BX005266.2	SLC1A3	TNFRSF19	AC009119.2
WAC	NES	CENPK	NANOS1	DDX60L	TP73-AS1	QRICH2	AC073130.2	DTX4	GPR15	ADAMTSL4
WAPL	NEK9	CENPJ	SPHK1	RAPGEF3	CORO2B	AL139246.5	AIMP1P2	PCDH17	LINC00472	CD27-AS1
ZFYVE19	NEK8	CENPH	MAPT	TRIM22	SPP1	CPNE5	DDN-AS1	AC083967.1	IL1RL2	ABI3BP
WNT5A	NEK7	CENPF	SEMA4B	MX1	TSPAN18	AC006460.1	PKIA-AS1	SEMG1	LIMA1	TRPM3
ZCWPW1	NEK6	CENPE	SFN	TGFB1	RASSF2	LINC00511	NT5CP1	SERPINB7	IFI44	NFYB
VCP	NEK4	CENPC	BCL6	PFKFB3	COL8A1	PI4KAP1	POU5F1P3	AC063952.1	TBX3	CMAHP
ZWINT	NEK3	CENPA	HSPG2	OAS2	TFAP2C	P2RX2	KRT8P43	AC093278.2	STEAP4	METTL7A
ZW10	NEK2	CECR2	VEGFA	CDKN1A	TCEAL9	AC004982.1	AC007342.6	CCRL2	AL627309.6	ATAT1
ZSCAN21	NEK11	CDT1	OSGIN2	C3orf58	BCHE	PLEKHB1	ST3GAL1P1	GNG2	PTGFR	ALDH5A1
ZPR1	NEK1	CDKN3	SESN2	MYEOV	AC020912.1	ZNF730	RPL7AP2	LINC00165	MCTP2	AC104435.2
ZNRD2	NEDD9	CDKN2D	SEMA3C	LCP1	DKK3	AL390195.2	LINC01431	ZNF704	LUARIS	LGALS9
ZNF503	NEDD1	CDKN2C	JADE1	C1R	GABRG2	AC090673.1	FBXL22	PTPRQ	GTF2IP8	CNTNAP1
ZNF318	NDEL1	CDKN2B	APP	HACD2	FXYD2	AC004233.2	KLRC1	COL5A1	NANOS1	DYNC1I1
ZNF16	NDE1	CDKN2A	NPPB	PLSCR1	MALL	AC004623.1	NR4A2	CA8	FLRT2	PI4KAP2
ZFYVE26	NDC80	CDKN1C	EPB41L5	GLRX	NPNT	AC009301.1	NR2E3	OR5H3P	ABCA4	RPL23AP49
ZC3HC1	NDC1	CDKN1B	ULK2	GPNMB	TRIM55	FGF21	FLJ31356	UCHL1	NEDD9	MIR645
ZBTB49	NCOR1	CDKN1A	HSPA1A	STAT6	SIGLEC15	FABP6	AC104162.1	CDH6	LINC01203	QPRT
ZBED3	NCAPH2	CDK7	WNT5A	SLC44A2	EDIL3	BOC	AC012618.3	HS3ST3B1	AKR1C3	PTCHD4
YTHDF2	NCAPH	CDK6	TGFB2	ANGPTL4	UGT2B7	AC092118.1	RPS15AP36	AP005230.1	PBX1	PIWIL4
YTHDC2	NCAPG2	CDK5RAP3	CAMK2D	FSCN1	SPNS2	AL603839.4	AC004837.2	GALNT6	RUVBL1-AS1	SERTAD4
YEATS4	NCAPG	CDK5RAP2	TSG101	TIMP2	WDR72	CASTOR1	AP000523.1	MPP2	AC026310.2	GPR150
XRCC2	NCAPD3	CDK5R1	RIMS2	MYO10	CNTNAP3P2	AC009242.1	RNASEH2B-AS1	AC010615.2	FPR3	RNF157
WRAP73	NCAPD2	CDK5	MEGF8	ASS1	ITGB6	GDPD2	ATP8B3	SLC22A3	LONRF2P1	CASP1
WNT10B	NBN	CDK4	TGFBR2	LSR	NTNG1	TMC6	ZNF853	L1CAM	AC002074.1	LYPD5
WEE2	NASP	CDK3	SOCS2	AQP11	ZBED2	AC114316.2	RPL36AP15	AC004784.1	AL161719.1	CCDC9B
WEE1	NAE1	CDK2AP1	PAK4	ECM1	CCDC198	NPIPB2	ADGRA2	OR10A6	ELOVL2	PTGER4
WDR62	NABP2	CDK20	PLXNB1	GFRA1	N4BP3	FAM87B	ITGB2-AS1	COL1A2	MRPL23-AS1	TENT5A
WDHD1	NABP1	CDK2	LPAR3	RNF144B	FUOM	AC016769.2	VWCE	IRX2	AC007204.1	ANO9
WASL	NAA60	CDK18	ZFYVE27	TMTC1	CNTNAP3B	CYP24A1	AC084036.1	CYP4F35P	CREB3L1	ZNF365
WASHC5	NAA50	CDK17	FXYD2	SHISA2	KIF12	LINC01305	CRB3	MRPL23	TMEM154	AC019155.1
VPS4B	MZT1	CDK16	EIF4G2	PKP3	KLHDC7A	CLEC18A	CR381653.1	CAPG	ADA2	PARP9
VPS4A	MYOG	CDK15	POSTN	GATA2-AS1	ZDBF2	EGFL7	DLX2	ZNF287	AL021407.1	AC063944.1
VCPIP1	MYH9	CDK14	TGFBR1	SLC37A3	SLC17A3	LINC01687	CFAP58	WNT10A	AC080013.1	GJB5
VASH1	MYH10	CDK11B	KRT17	HLA-B	ZNF71	AC002128.1	STK19B	HMGB1P1	NBPF4	CEP164P1
UXT	MYC	CDK11A	SPOCK1	NOV	ARSE	AC013451.2	SNORA47	AC009275.1	AC098613.1	NELL2
UTP14C	MYBL2	CDK1	CDA	TP53I11	TINAG	PLGLB1	FAM95C	DIO2	SPOCD1	SYNE3
USP9X	MYBL1	CDCA8	SYT17	IFI44	PLCXD1	VWA5B2	FTLP12	ZNF502	SLC5A11	AC006262.1
USP8	MYB	CDCA5	PLXNA2	FOXL1	SHISAL1	STOM	RPL21P132	AC098864.1	OR2I1P	INHA
USP37	MUS81	CDCA3	RTN4R	IFI27	AC006452.1	LRRN4CL	TEX19	ANKRD36BP2	AC139713.2	AKR1C2
USP33	MUC1	CDCA2	SEMA3B	PAPSS2	SFN	MAPK15	AC092794.1	VTN	PALM2	SYNPO2
USP3	MTBP	CDC73	FBP1	TNFRSF21	CYS1	LINC01775	AC090409.1	CACNG6	ADGRB2	LRRC34
USP2	MTA3	CDC7	SEMA5A	PLPP3	BX571818.1	ITGB8	AC091153.1	LRP3	HADHAP1	HOXD8
USP17L2	MSH6	CDC6	ZNF639	SPINT2	COBL	AL157893.1	PRH1-PRR4	DPYSL3	CPEB1-AS1	FRG1EP
USP16	MSH5	CDC5L	RRAGC	NPNT	HOXC9	AP001271.1	AC106037.2	KCTD8	CYP46A1	AGMAT
USH1C	MSH4	CDC45	COBL	SBF2-AS1	NF2	CPNE8-AS1	FAM85B	GPAT2	AC009163.6	WT1
URGCP	MSH2	CDC34	EPHA4	DKK3	SOCS2	CCDC26	PSORS1C3	SULT1A1	CDK14	CTSH
UPF1	MRPL41	CDC27	ULK1	ADAM12	PLCH2	PAQR6	MIR4482	MYL9	HLA-B	TYMP
UNC119	MRNIP	CDC26	PRKCZ	PTK7	SLC17A1	AC010331.1	TNF	EWSAT1	AC099063.4	AC119674.2
UIMC1	MRE11	CDC25C	SOX9	CARD10	HGD	SH2D5	MATR3	HSPB3	SERPINB4	AL139383.1
UHRF2	MPLKIP	CDC25B	GSK3A	CYBRD1	SLC37A1	UBASH3B	CR392039.1	TBC1D3L	CNTFR	TIMP2
UHRF1	MOV10L1	CDC25A	CHPT1	PLEKHA7	TM4SF1	MYEOV	MYADML2	ABCB1	ACSL5	ARID5B
UBXN2B	MOS	CDC23	CYP27B1	RAI14	KCNF1	EFNA3	FES	PITX2	RDH10	MCAM
UBR2	MNS1	CDC20	CTGF	IFI44L	LINC02434	ASPRV1	GLDN	IGKC	PKP3	ROPN1B
UBE2S	MND1	CDC16	TNFRSF12A	DPP4	PHF21B	AC073195.1	AL133406.3	GLRB	CADM2	AP000919.2
UBE2L3	MNAT1	CDC14B	PLAA	CMTM3	RAB19	LINC01833	SNORD117	CPEB1	FOLR3	APOL3
UBE2I	MMS19	CDC14A	EIF2AK4	HAS3	SMO	FAM238C	SYNGR4	SLC7A8	AKR1C1	LGALS3BP
UBE2C	MLH3	CDC123	PPARG	KAZN	LRCH2	AC008079.1	PSAT1P3	CCDC194	SLC25A24P1	IRF7
UBE2B	MLH1	CD2AP	NET1	LIF	MMP17	KCNK10	RNA5SP187	AC079760.2	CPB1	KRT33B
UBB	MLF1	CCSAP	SFRP1	TNFRSF11B	SHROOM2	PADI1	CASC15	NLGN4Y	CD14	HACD1
UBA3	MKI67	CCPG1	MMP14	LTBP4	KCNJ2	WNT2B	AC005005.3	EDN2	MAB21L1	PSMB9
TXNL4B	MITD1	CCP110	SH3BP4	DAB2	FRG1CP	PCAT6	AL512422.1	KCNJ3	CCDC141	CTSS
TXNL4A	MISP	CCNY	FOXL2	DOCK11	EEF1A2	AC006518.1	DPF3	SELENBP1	HGF	AC099518.1
TXNIP	MIS18BP1	CCNT2	EGFR	ADAMTSL4	SCEL	SIGLEC16	RF00134	ECHDC3	HTR1B	ABCC6
TXLNG	MIS18A	CCNT1	PDLIM5	UBASH3B	FGF5	AL357055.3	HIST1H2APS4	LRRC2	AL357054.4	AL034374.1
TUSC2	MIS12	CCNP	PML	ARHGAP23	CHST9	AC105233.5	ZFPM2	ZNF366	AC074141.1	LINC01504
TUBGCP5	MICAL3	CCNO	DCBLD2	CALD1	CYP27C1	LINC02361	PRICKLE2	CCL5	HECW1	GSTM3

TUBGCP2	MEIOSIN	CCNJ	CSNK2A1	ATP2B4	VSIG1	LINC00271	AC012213.1	NRROS	RPS6KL1	GPX3
TUBG2	MEIOC	CCNI2	GOLGA4	LGALS3BP	SELENOM	COL18A1-AS1	LINC02204	TSPYL5	RASGRP3	AC008840.1
TUBG1	MEIOB	CCNI	RNF6	MATN2	SHANK2	AC011479.3	TMEM184A	ZNF583	HLA-F	KCNAB2
TUBE1	MEIKIN	CCNH	HSPA1B	PSG4	B4GALNT4	NRSN1	ANKRD20A1	AP005121.1	LINC00944	AC092070.2
TUBD1	MEI4	CCNG2	EDN1	TENT5A	CNTNAP3C	AL136452.1	LINC02560	EMILIN2	AC026801.2	KLF15
TUBB8B	MEI1	CCNG1	EFNA5	PTPRU	RPL39L	AC004706.3	ERVH48-1	GABRE	LINC01151	NR2F1
TUBB8	MED1	CCNF	SRF	IFI16	DUXAP9	IGFBP4	AL359397.1	DEPP1	NECAB1	GAD1
TUBB6	MECP2	CCNE2	PPT1	OGFRL1	BRSK1	KCCAT198	CLIP2	AC018647.1	HMGB2P1	LHX9
TUBB4B	MDM2	CCNE1	SERTAD2	CYTH1	VSTM1	TMEM74B	ARID3A	PRKY	PSG6	GATA6
TUBB4A	MDC1	CCNDBP1	RACK1	DTX3L	AC021218.1	ADAMTS9-AS1	AL451074.2	HNRNPA1P33	EGLN3	CCDC71L
TUBB3	MCTS1	CCND3	RAB21	DCDC2	FGFR4	AC015819.1	CD72	KCND3	ANKRD1	AC016590.3
TUBB2B	MCPH1	CCND2	DNAJB2	EIF5A2	AL035446.1	ADAM21	PRAME	S1PR1	AL512625.3	DKK1
TUBB2A	MCMDC2	CCND1	PAPPA2	DRAM1	SOBP	GJD3	CELF2	SLC4A4	SLC2A5	FBXO27
TUBB1	MCMBP	CCNC	NLGN3	MAP3K8	TEAD2	AL352955.1	MIF	ZNF649	ZFP3	WBP2NL
TUBB	MCM8	CCNB3	STK11	ANXA8	FXYD6	AC108471.2	SLC11A1	MECOM	LEP	CPED1
TUBAL3	MCM7	CCNB2	PRMT2	HLA-F	NDUFA4L2	AC006486.2	USP51	AC091544.4	MIR7976	AP3B2
TUBA8	MCM6	CCNB1IP1	FN1	ITGB8	ITGA10	FAM230C	LINC02381	C11orf45	SLC25A27	MPP7
TUBA4B	MCM5	CCNB1	CDK5	BICC1	GATD3B	HSPB6	TTC3P1	TRPC4	AC244230.2	AC078993.1
TUBA4A	MCM4	CCNA2	RERG	ANKRD1	DDIT4	AC006042.1	PKD1P5	TMSB4Y	PTPN20CP	RNY1P16
TUBA3E	MCM3	CCNA1	CISH	LGALS3	NUS1P2	TRPM8	TM6SF1	SNX19P3	AP002761.4	IFIT3
TUBA3D	MCM2	CCDC69	RIMS1	IFI6	AC004241.5	AL390066.1	COL13A1	STAT6	FLT1	KLF5
TUBA3C	MCIDAS	CCDC61	GJA1	SEC61A1	SLC6A17	HKDC1	LINC01424	PLEKHG4	TM4SF20	APBB1IP
TUBA1C	MAU2	CCDC57	CLSTN3	ST5	EFNB3	FAM83E	GRB7	OASL	LCA10	ZYG11A
TUBA1B	MASTL	CCDC124	NRP1	PKP2	GFPT2	ADORA2A	PDZK1IP1	DLX6-AS1	AC025588.1	MT1F
TUBA1A	MARVELD1	CCDC102B	NOP58	AP1M2	EGFLAM	TMEM121B	MYOM2	PCDH9	LINC00518	NR2F1-AS1
TTYH1	MARK4	CCAR2	LAMTOR1	SLC1A1	MFNG	TMEM191B	GJA3	OR10A3	ZNF662	DTX3L
TTN	MARF1	CCAR1	SMARCA2	SMTN	AL713998.1	ESPNL	HIST2H2BF	TIMP4	LRAT	GCOM1
TTK	MAPRE3	CATSPERZ	GNG4	CCDC80	BRSK2	AC011479.2	C17orf107	GPNMB	GRAMD4P7	AC093535.1
TTC28	MAPRE2	CASP8AP2	KAZALD1	HPS5	TGFA	F2RL1	ADRA2C	KCNN2	RAMP2-AS1	HACD2
TTC19	MAPRE1	CASP2	DNM2	TGFA	SNORC	SSPO	AC091132.2	AC098850.3	SERPINB9P1	TSPY26P
TSPYL2	MAPK7	CAPN3	NKX6-1	SLC2A3	CERNA2	AL049536.1	AC069335.1	PCDH10	AC005586.2	EFCAB6
TSG101	MAPK6	CAMK2A	PAK1	GPRIN1	NLRP3P1	AC004466.2	TPM2	HPGD	CRTAC1	LINC00665
TRNP1	MAPK4	CAMK1	TSPYL2	MYO1D	ZNF182	MAP4K1	FAM110C	DUSP23	LY75	ANKRD31
TRIP13	MAPK3	CALM3	MACF1	FKBP10	FAM49A	HERC2P10	AL157700.1	SFRP4	AL121992.1	SP6
TRIOBP	MAPK13	CALM2	SLIT2	IGFBP7	TIMD4	DNM1P51	AC020612.1	CALB2	AC124067.4	AP003064.2
TRIM75	MAPK12	CALM1	CRABP2	KLF9	HOXB2	FAM182B	AL136038.4	GRIP1	AC079298.3	
TRIM71	MAPK1	CACUL1	LLPH	TRNP1	TMEM106A	AL137145.2	AL450306.1	FAM218A	AC131532.1	
TRIM39	MAP9	CABLES2	SGK2	CRYBG1	AL157931.1	AC002128.2	AL049840.6	LINC02577	KCNG3	
TRIM36	MAP4	CABLES1	DNPH1	PHLDB2	KLHL14	TSPEAR-AS1	FSD1	FOXL2NB	ARHGAP23P1	
TRIAP1	MAP3K8	C2CD3	USP47	CCDC71L	DUXAP10	KRT86	ATP9A	FAM106A	TMC1	
TREX1	MAP3K20	BUB3	CDH4	SERTAD4	BX322562.1	AC005326.1	AP001931.1	BISPR	HAND1	
TRAPPC12	MAP3K11	BUB1B	EXOSC2	SLC48A1	SOCS2-AS1	SMTN	TLE3	TACSTD2	CYP4F26P	
TPX2	MAP1S	BUB1	HDGFL2	ARL4C	WNK4	AC138951.1	KLC3	BEND3P1	ICOSLG	
TPR	MAP10	BTN2A2	ITCH	IGFBP3	RAPGEF3	AL589743.4	LINC01145	ANOS1	BDH1	
TPPP	MAJIN	BTBD18	APBB2	PARP9	CLDN2	AL353807.3	DUX4L50	GSG1	OXTR	
TPD52L1	MAEL	BRSK2	TAOK2	HACD1	EFHD1	GJB2	GCNT3	CDA	APCDD1L	
TP73	MAEA	BRSK1	RHOA	SOX13	AREG	CAMSAP3	S100A1	CYB5R2	AC023158.1	
TP53	MAD2L2	BRME1	RARG	MGST1	ANK3	AC130371.1	NRG2	TXNIP	ENPP3	
TOPBP1	MAD2L1	BRIP1	MAP1B	SLCO4A1	SBK1	DHCR24-DT	UPK3B	EFEMP1	GATA2-AS1	
TOP6BL	MAD1L1	BRINP3	RAPH1	ADD3	LINC01320	MIR4713HG	OLFML2B	AL121929.2	AC007991.2	
TOP3B	M1AP	BRINP2	PLXNA3	CLMN	STARD4	MYH15	SYT11	SIM1	GPAT2P1	
TOP3A	LZTS2	BRINP1	CXCL16	NFYB	LINC01127	C2orf27AP1	RPH3AL	ZNF582	AC012625.1	
TOP2B	LZTS1	BRDT	PTPRJ	MICAL1	ZIK1	AC000078.1	TRNP1	DTX3	LINC01535	
TOP2A	LSM14A	BRD7	ITSN2	TMEM52B	TMEM37	LHFPL5	TLR6	SEMA5A-AS1	ZNF114-AS1	
TOP1	LRRCC1	BRD4	NCBP1	ANXA8L1	SLC47A2	BX088651.4	CHDH	SAA1	MYLK	
TOGARAM2	LPIN1	BRCC3	RPS6KA1	NELL2	PXDN	AC241377.4	S100A2	GXYLT2	LTF	
TOGARAM1	LMNA	BRCA2	CDKN2A	IFIT1	FKBP1B	LRRC71	PSD4	SLFN12L	AC124067.2	
TNKS	LMLN	BRCA1	SEMA4G	SIRPA	DOK7	EIF4HP2	HOXB-AS1	NRN1	BX842568.2	
TMPRSS11A	LLGL2	BORA	NRCAM	PLIN2	CRLF1	LINC01140	RASEF	COL5A2	SEMA3D	
TMOD3	LLGL1	BOLL	DGKD	ALDH5A1	TFEB	SLC5A9	PLPPR3	FSTL1	FOXN4	
TMEM250	LIN9	BOD1L2	PIN1	RHOBTB3	B4GALNT3	GABBR2	AC005332.5	OLFML1	RRAD	
TM4SF5	LIN54	BOD1	PPP1R9B	LPCAT1	VWA1	LINC02274	CORO6	CTTNBP2	AC020571.1	
TLK2	LIMK2	BLM	CYFIP1	PYCR1	INHBB	CT45A1	MAFG-DT	LYL1	CALHM3	
TLK1	LIG4	BLCAP	HDAC6	ABCA1	STARD8	TGM4	MYRFL	ZNF880	AC005920.1	
TLE6	LIG3	BIRC6	KIF14	ID2	ADGRG1	AL139424.2	AC139495.3	ADPRH	FGD3	
TIPIN	LIG1	BIRC5	APBB1	ALDH3B1	MYO10	ARPC4-TTLL3	AC116021.1	HMGCLL1	ERICH2	
TIMELESS	LIF	BIN3	CDC73	ANXA1	MYO5B	HAS3	EPHB2	ARHGAP22	CFI	
TICRR	LCMT1	BEX4	PPP3CB	STXBP6	LINC01876	KDM7A-DT	SLC27A5	AP001610.1	LINC02009	
THAP5	LATS2	BEX2	EGLN2	PDCD1LG2	HOXA10-AS	PCDHB7	XDH	PSG7	AC021733.1	
THAP1	LATS1	BECN1	DCUN1D3	TPM2	OSCAR	LTBP2	LRRC75B	AC010889.1	NHS	
TFDP3	KRT18	BCL2L11	ABL1	TACSTD2	LINC02575	MANCR	AC010536.2	ALPP	CCDC122	
TFDP2	KPNB1	BCL2	ADIPOR2	CUL4B	FIBCD1	AGGF1P2	NDUFB2-AS1	FPR1	ZNF597	
TFDP1	KNTC1	BCCIP	TMEM97	OAS1	TNC	AC090833.1	SPOCK2	MIR34AHG	AC008957.3	
TEX19	KNSTRN	BCAT1	LIMK1	LINC01085	CEBPA	DDX25	PLIN4	GAS1	SLC7A2	
TEX15	KNL1	BBS4	CLSTN1	HERC6	AC007846.1	C2orf66	SHISA3	CPM	LRRK2	
TEX14	KMT5A	BARD1	KMT2D	AC021218.1	TLR1	AC084759.3	LINC01111	ZNF334	ESRRG	
TEX12	KMT2E	BAP1	DPYSL2	CCL5	HORMAD1	MFAP4	CLDN16	KRT14	RERG	
TEX11	KLLN	BANP	CYBA	CD55	MCF2L	RN7SL663P	FLJ45513	PRND	IFIT1	
TET2	KLK10	BANF2	RBBP7	BMPER	CD37	AL162231.2	LINC01001	CLSTN2	GUCY1A1	
TESMIN	KLHL9	BANF1	SERTAD3	ATXN1	ZNF467	AL162431.1	C20orf144	S1PR3	UNC13D	
TERF2	KLHL42	BAG6	TWF2	NR4A1	PCDHGB7	HIST1H2APS2	FN1	UGT1A7	KCNV1	
TERF1	KLHL22	BACH1	TP53	PLEKHA6	ENDOU	CNN2P1	HIST1H2AE	ZNF521	AF241728.1	
TERB2	KLHL21	BABAM2	UCN	SLC15A3	FBXL16	AL365203.2	EPHA10	SLITRK5	AC022034.1	
TERB1	KLHL18	BABAM1	SHTN1	GGA1	LHX1	FOXL1	SH3TC1	PRRT4	PRRX2	
TENT4B	KLHL13	AZI2	CDK11B	GBP1	RPSAP52	AC126323.1	ABCG4	TIMP3	HOXA11	
TENT4A	KLHDC8B	AVPI1	IFRD1	SLC44A1	PAPPA2	VPS9D1-AS1	MTMR8	RBP7	TWIST1	
TDRKH	KLHDC3	AURKC	HTRA1	ZNF350	TBC1D30	AP000525.1	INO80B	LRRIQ1	ATP2B4	
TDRD9	KLF11	AURKB	IP6K2	PRNP	FSCN1	RASL10A	EFNA1	EHF	LINC01956	
TDRD12	KIZ	AURKA	ESR2	LYPD6B	CCR7	SLC16A13	SIRPB3P	COL14A1	CRYBG1	
TDRD1	KIFC2	AUNIP	DVL1	TNFAIP3	ST6GAL1	OR2AT4	AC068134.3	LINC02385	CTAGE11P	
TBRG1	KIFC1	ATRX	HTRA3	IFIT3	ENPEP	RGS17	IGSF10	ALDH2	AC012501.2	
TBCE	KIF4B	ATM	PRKN	FBXO17	AL162151.2	AL627389.1	AC010326.4	RNF180	OFCC1	
TBCD	KIF4A	ATF2	G6PD	KLF5	TUBB4A	DKKL1	MSH4	NSUN7	CXCR4	
TAOK3	KIF3B	ATAD5	SEMA4F	ITM2C	AC243562.2	LINC01844	ITGB1-DT	LY6E-DT	TNFRSF21	
TAOK2	KIF3A	ASZ1	DCLK1	STOM	GAL3ST1	AC112907.2	GPR161	DIO3OS	DUBR	
[illegible]	[illegible]	[illegible]	[illegible]	[illegible]	[illegible]	[illegible]	[illegible]	[illegible]	[illegible]	

TAF1L	KIF2A	ARL8B	FGF13	IRF2BPL	LIPG	PPFIA4	TOB1-AS1	AKR1E2	CACNA1C
TAF10	KIF25	ARL8A	SLC3A2	PRXL2A	CISH	MELTF	NPIPA3	CD34	OPN3
TAF1	KIF23	ARL3	CDKN2D	PARP14	LINC01012	AC136475.2	TRIM9	CASP10	PDE4B
TADA3	KIF22	ARL2	CDHR2	LTBP2	SPEF2	AL162231.1	SNORA60	KCNK2	AL158211.5
TACC3	KIF20B	ARHGEF2	EIF4G1	PI4KAP1	AC109361.2	ARHGAP9	MEF2B	GUSBP5	LINC02012
TACC2	KIF20A	ARHGEF10	LHX2	CD274	FAM27B	CACNA2D4	LFNG	PDGFRB	IL1A
TACC1	KIF18B	ARF6	CYR61	SLC1A5	LINC00649	ITIH2	ADRB2	ZMAT1	ELFN1-AS1
SYF2	KIF18A	ARF1	BLZF1	ZNF615	RHOB	EGFR-AS1	ERFE	CXCL11	SPARC
SYCP3	KIF15	ARAP1	LGI1	C9orf3	AC092153.1	TNRC6C-AS1	SLC14A1	KLHDC9	SLCO5A1
SYCP2L	KIF14	APPL2	SMAD3	VGLL3	SERPINA1	PM20D1	THSD4	SYT10	AC114878.1
SYCP2	KIF13A	APPL1	FHL1	LAMB3	STK31	SMTNL2	HIST1H3G	ADCYAP1	OR7E14P
SYCP1	KIF11	APP	NEDD4L	ZEB2	EVPL	RUNDC3A	MYO15B	C1S	RN7SL810P
SYCE3	KIAA1614	APEX2	NDN	EDIL3	UNC5CL	INHBA	CHKB-CPT1B	HSPB8	NRIR
SYCE2	KIAA0753	APC	RPS6KA3	SKP2	TLL2	BMS1P17	PPL	PNPLA1	PLXNA4
SYCE1	KHDRBS1	ANXA11	CDKN2AIP	MEST	MAPK10	AC107419.1	MT2P1	ZNF736P9Y	PRRX1
SUV39H2	KDM8	ANLN	H3F3A	ELAVL2	AL589743.5	EMBP1	AADAT	GNA15	AC016582.1
SUV39H1	KCTD11	ANKRD53	ADNP2	MAOA	AC005722.2	KAZN	GNB3	PLEKHS1	LGALS3
SUN2	KATNB1	ANKRD31	BRAT1	DEK	AF131215.6	AC004890.3	CXADR	ZNF879	LINC02015
SUN1	KATNA1	ANKLE2	AKT1	MFSD1	SFTA1P	AL513174.1	AC093909.1	ANKRD7	C9orf170
SUGT1	KAT5	ANKLE1	SMURF1	PAX8-AS1	SYT13	CCDC168	AL606534.4	LCP1	AC092919.1
STRADB	KAT2B	ANKFN1	SIRT6	PLCD3	UNC13C	KRT15	AL390198.2	CCDC144A	ACY3
STRADA	KASH5	ANK3	BTG1	KLHL4	SLC16A8	TMEM59L	C1orf229	ART4	CNN1
STRA8	JTB	ANAPC7	NDUFS3	MN1	CAPN13	ABCA12	OCLNP1	TNFRSF1B	PLEKHA6
STOX1	ITGB3BP	ANAPC5	MAP2K5	MCM2	DOCK4	ZNRF2P2	HCN2	GRIA1	MKRN3
STMN1	ITGB1	ANAPC4	CRYAB	EFNA1	SLCO2B1	C1orf146	LINC01748	ABCC6P2	AP001025.1
STK35	IST1	ANAPC2	CCAR2	DDX58	BMS1P22	IMPDH1P5	TBX15	SPOCK1	CFH
STK33	IQGAP3	ANAPC16	CDKL5	PADI1	NEGR1	C11orf94	AGPAT4-IT1	MAST4	COLCA2
STK11	INTS3	ANAPC15	ADIPOR1	IDH1	SEMA6B	AC234782.4	NKAIN4	C1R	NPY4R2
STK10	INTS13	ANAPC13	GDI1	PLAC8	ACTA2	LARGE1	ADAMTS9	NKX6-1	TRABD2A
STIL	INSM1	ANAPC11	IST1	TXNRD1	NLGN3	MST1R	SEMA3F	ARNT2	AC011447.7
STEAP3	INSC	ANAPC10	SGK1	CYB5A	BASP1P1	NOP53-AS1	HAP1	BAHCC1	AC073869.3
STAT5B	INO80	ANAPC1	ERBB2	MCAM	DSCAML1	AC022400.8	OR2A1	PDLIM3	ARHGEF6
STARD9	INIP	AMBRA1	ACVR1B	CAVIN2	NOVA2	IGFALS	AL078621.3	OR10Y1P	GAS1RR
STAMBP	ING4	ALKBH4	PPARD	ZNF432	TNFSF15	AC084864.1	ANG	ZSCAN23	AP000919.4
STAG3L4	ING2	AKAP8L	SPG11	C4orf19	GLI2	TESC	PYGM	GBP4	AC008592.3
STAG3L3	ING1	AKAP8	TAF9	LIMCH1	AQP4	GPR55	LRFN1	FMN1	LINC01106
STAG3L2	INCENP	AJUBA	PSRC1	HTATIP2	AL357033.1	AC005077.4	PTGER1	CETP	DACT1
STAG3L1	IKZF1	AHR	IGFBP5	FBLN1	COL24A1	H2AFY2	IGSF3	AL035467.1	KRT19P1
STAG3	IK	AHCTF1	SEMA3A	KRT8	MIR3189	SLC37A3	LINC01315	LINC01444	AC104237.1
STAG2	IHO1	AGO4	MUL1	ZNF649	GDF15	SNRK-AS1	WTIP	TMEM229B	PRSS8
STAG1	IFFO1	ACVR1B	WFDC1	SLC35F2	PCDHGA12	PRMT1P1	SLC48A1	LINC02315	AL133304.2
SSX2IP	IER3	ACTR8	KIAA1109	TES	1-Dec	AC008443.3	PDIA3P2	ZNF619	APOBEC3G
SSNA1	ID4	ACTR3	NUBP1	H2AFY2	MSR1	AC006538.1	GUCA1B	CCDC184	AL357140.4
SRSF2	ID2	ACTR2	SMAD4	GSTM3	PRR5	BEGAIN	GPRIN1	TYRP1	RASSF9
SRPK1	HUS1B	ABRAXAS2	FXN	SLC6A9	HRCT1	SIRPA	AL445435.1	MYRIP	DUTP1
SRC	HUS1	ABRAXAS1	ATAD3A	SLC39A8	DHRS2	AP1M2	ACMSD	HBD	SSTR2
SPTBN1	HTT	ABL1	SEMA3D	UNC13D	HMGA2	DGCR5	NOTUM	EVA1C	AC007743.1
SPRY2	HSPA2	ABCB1	C9orf72	ZNF738	GAMT	NBEAP3	HCG23	RPP25	IFI16
SPRY1	HSF2BP	AAAS	WISP2	TMEM173	CDH20	TBR1	PLAU	SPX	GPRIN2
SPOUT1	HOXA13		CEACAM1	PELI1	NRSN2	KCNJ2-AS1	HIST1H4K	SLC43A3	EDNRA
SPO16	HORMAD2		MSX1	PRDM11	KRT223P	AC253536.3	AC018653.3	IL20	ISG15
SPO11	HORMAD1		NDUFA13	LRP2	AC005070.1	AC090559.1	SMOX	FLNC	ZBP1
SPIRE2	HNRNPU		SEMA5B	MST1R	SIRPG-AS1	AL035448.1	AC016245.1	EEF1DP5	CTLA4
SPIRE1	HMGA2		NPR1	NEDD9	TNFSF9	TMEM204	LINC01182	HOXB13	LINC01117
SPIN2B	HMG20B		BCAR1	VEGFC	TBX2-AS1	SVIL2P	SLC9B1	PCSK5	FLG-AS1
SPIN2A	HMCN1		USP9X	MTHFD2	TNS1	AFAP1-AS1	MAP1LC3A	PLXDC2	GAPDHP32
SPIN1	HJURP		MTPN	HLA-C	CALHM5	MNX1-AS2	SLC37A2	PABPC4L	UBQLNL
SPICE1	HINFP		ENO1	PTPRM	ESAM	AC068946.1	LINC00632	HLA-DRA	LINC00348
SPECC1L	HGF		AKAP6	B2M	SHISA2	CAPN14	C10orf62	LINC00968	AC084262.2
SPDYC	HFM1		EXOSC4	ADARB1	LINC01224	LMO7DN	ITGA9-AS1	PSTPIP2	MYH14
SPDYA	HEPACAM2		SIPA1	GFM1	C8orf48	RNU1-103P	CCDC192	PLSCR4	AC087752.3
SPDL1	HEPACAM		PLXNB3	SLC12A3	PEAR1	ENHO	ZP3	C1GALT1C1L	ABAT
SPC25	HELLS		SGK3	TMEM158	HOXC10	SIGLEC10	TAS2R5	HAGLROS	CH25H
SPC24	HDAC8		NAIF1	SNX4	LINC00561	AC024575.1	AC023157.1	LINC01638	MIR30C2
SPATA22	HDAC3		ERBIN	PTPRK	TRIM74	AC026461.3	AC063965.1	FBLN7	AC079610.2
SPAST	HCFC1		NDEL1	ACSL5	RHEX	LINC01433	SAPCD2P3	ZNF501	LSAMP
SPAG8	HAUS8		CDK5R1	DUSP4	AC004846.1	AL589765.4	ADAMTS14	ECM1	AJ271736.1
SPAG5	HAUS7		F2	TBC1D4	EMC10	KCNE2	AC007952.2	LINC01391	ST18
SON	HAUS6		N6AMT1	PKD1P5	AL358334.2	AC079305.1	KDM7A	KLF14	AC016769.3
SNX9	HAUS5		CAPRIN2	INHBA	AL359715.2	AC008555.8	AGAP10P	CSAG3	RBPMSLP
SNX33	HAUS4		SIRT1	HLA-E	AL117329.1	AP006621.4	PRLR	FAM110B	LINC01018
SMPD3	HAUS3		PLCE1	TLR4	COL18A1	ASNS	MME	AC021678.2	RPS2P32
SMC5	HAUS2		CPNE1	LTBR	WFDC21P	AC026691.1	EPSTI1	CNTN5	PWP2
SMC4	HAUS1		SEMA3E	SULF1	EGR4	AP003501.3	HCP5	ZNF804A	AC011447.4
SMC3	HASPIN		NDRG4	ANK2	SOX2-OT	KHDRBS3	LRP2	IL1RAPL1	RF00096
SMC2	HACE1		CIB1	SDCBP	RAB17	KCNH8	PRUNE2	ZNF732	ZNF385B
SMC1A	H2AX		ARIH2	PLEKHA2	RPS6KA2	TAPT1-AS1	PPARGC1A	LINC00882	OR51B5
SMARCB1	H1-8		DDX39B	FAS	SLC6A16	RTL5	LUM	IL9R	OR5E1P
SMARCAD1	GTSE1		ADAM15	TXNRD3	NR4A1	AC020891.2	GMPR	FOLR1	AC023158.2
SLX4	GTF2B		PLXNA4	SHANK2	VMO1	AC244021.1	PSG2	SOX5	PHLDA3
SLC39A5	GSPT2		EXOSC9	GULP1	TMEM121	ADAM20P1	NCAM2	ANO2	AL359182.1
SLC2A8	GSPT1		IL17RB	PTPRE	AL137784.3	AC100847.1	SDR42E1	PSG9	COL5A1-AS1
SLC26A8	GPSM2		RASGRP2	SCIN	LY96	LAT2	HSD3B1	FREM2	WNT5A-AS1
SLC25A5	GPSM1		SYT1	IRF7	NFATC1	DMBT1	LYPD6	ZNF688	AC027335.1
SLC25A31	GPR132		CDKN1B	ZNF577	AL022322.1	AC009269.4	GYPC	ARMCX4	AC006299.1
SLC16A1	GPER1		ENOX2	TNFRSF19	LINC02377	AC010463.3	RFLNA	TMEM246	TNIP3
SKP2	GOLGA8T		NTN1	GPAT2	SPNS3	AC015977.2	CPVL	AC106791.1	JAKMIP2
SKA3	GOLGA8S			SAMD9	LINC00621	PMEPA1	KLHDC7B	IFITM3	AL663070.1
SKA2	GOLGA8R			ABCG2	GPC4	AC010913.1	PTGES	PDCD6IPP2	PCSK1N
SKA1	GOLGA8Q			PTK2B	ITGA11	AURKAP1	IFITM1	OR2T8	AC011416.3
SIX6OS1	GOLGA8O			ANKS6	TECTA	AC018413.1	ANO5	ZNF606	AC007879.3
SIRT7	GOLGA8N			KALRN	FOXC2-AS1	AC005009.1	CTSF	CCR2	TEX41
SIRT2	GOLGA8M			LINC00511	AC027559.1	C11orf86	ARMCX1	AC012470.1	AL158839.1
SIK1	GOLGA8K			DSC3	PPP2R2C	FLJ30679	CNKSR2	INA	B3GALT2
SIAH2	GOLGA8J			EDNRB	GOLGA6L10	CSRNP3	RPS24P17	SYT9	ZNF793
[illegible]	[illegible]			[illegible]	[illegible]	[illegible]	[illegible]	[illegible]	[illegible]

SHCBP1L	GOLGA8G	PPP1R13L	P2RX5	AC004556.1	UROC1	MLPH	MAGI2-AS3
SGSM3	GOLGA8F	BST2	FGFBP1	ASB12	TOX	ZNF630	SDK1
SGO2	GOLGA8DP	IFIH1	SRGAP3	GALNT12	SLC12A3	LINC01679	RNA5SP490
SGO1	GOLGA8CP	ST3GAL1	KCNIP1	KNDC1	ANXA6	CFAP300	LINC01725
SETMAR	GOLGA8B	PSD3	BEAN1	ICA1	MX2	AL445490.1	ABCC6P1
SETDB2	GOLGA8A	KCND3	MN1	MIR135A1	DAPK1	LINC01123	NALCN
SETD2	GOLGA6D	HOMER2	RND1	MIR3176	GJA5	ALDH1L1-AS2	LHX2
SEPTIN9	GOLGA6C	CYTH3	SPRY4-AS1	SLC14A2	RIN3	ZNF577	OAS1
SEPTIN8	GOLGA6B	NRP2	ZNF385A	HIST1H2AL	RGS7BP	MIR6863	SPCS2P3
SEPTIN7	GOLGA6A	CLMP	PTCH2	LRRC66	CAVIN3	MYHAS	RNU1-72P
SEPTIN6	GOLGA2P5	PLXNA1	CPSF1P1	AGAP1-IT1	CHRDL1	BMP4	ZNF578
SEPTIN5	GOLGA2	TPD52L1	DMD	PLSCR3	OLFML3	NRK	IQCA1
SEPTIN4	GNAI3	NCOA7	AL590094.1	LINC00638	CADM1	SLPI	LINC01085
SEPTIN3	GNAI2	TRIB1	ADGRL4	IGFL1	WNT6	AC091544.2	PDE4D
SEPTIN2	GNAI1	DDX60	AP000757.2	ROBO2	GCNT4	TTC23L	AC129502.1
SEPTIN14	GMNN	CBX6	CYP27B1	AC012313.6	FOXF2	BFSP2-AS1	TNFRSF4
SEPTIN12	GMNC	GPR161	AC003072.1	AC011043.1	TRIM58	RPL29P19	KCNE3
SEPTIN11	GML	EFEMP1	CST6	ZFP2	USP9Y	AL139405.1	AC092903.2
SEPTIN10	GJA1	NUP210	AL359834.1	VWA2	ANKRD30B	AL133467.2	ZNF649-AS1
SEPTIN1	GINS3	DUXAP10	LURAP1	AC093162.2	TBX18	LINC01116	CR1L
SENP5	GINS1	NLRC5	AL035446.2	OLAH	ALDH1L1	ZNF300	LAMC2
SEH1L	GIGYF2	NUAK1	PPP1R14C	LINC01393	IL7R	AC138649.1	AC026894.1
SDE2	GFI1	GRIP1	KRTAP2-4	ARVCF	TMEM52B	CCL28	CBLN2
SDCCAG8	GEM	SMOX	LINC01268	AC242842.1	GABRA2	MYOT	NME5
SBDS	GATA6	CPOX	BCO1	AC083964.2	GALC	PSG8	DOCK10
SASS6	GAS2	GCH1	AP003900.1	AATK	TTTY15	CLMP	TNFRSF11B
SAPCD2	GAS1	LAT2	PAX8-AS1	GTSE1-DT	ULK2	AL353743.4	BATF
SAC3D1	GAK	SHC1	AL136382.1	CYP4F29P	AL139002.1	IL2RG	AL359633.2
RUVBL1	GADD45GIP1	PADI2	AC009093.4	SLC28A1	PLIN2	SLC38A4	TMEM179
RTTN	GADD45A	CLDN1	MNX1	DENND2A	SNHG18	QRFPR	AC005089.1
RTKN	FZR1	PMP22	AC107308.1	RNU6-1053P	SCIN	NHLRC1	POTEC
RTF2	FSD1	SWAP70	LTBP4	AC016957.2	OLR1	MYO5LID	GJB3
RTEL1	FOXO4	TAP1	AC138969.1	DAND5	VGLL2	ZNF844	CES4A
RSPH1	FOXN3	KRT18	LINC02516	AC013403.2	FUT9	LINC01124	KLF9
RRS1	FOXM1	SRGAP3	TSPAN15	SEPT5-GP1BB	CRISPLD1	EPHA6	CYBB
RPS6KB1	FOXJ3	SCARB1	AC021066.2	AC078864.1	NEURL1	SLC19A3	EEF1B2P7
RPS6KA3	FOXJ2	DUXAP8	SGK2	AC027020.2	BST2	LUZP2	AC027796.5
RPS6KA1	FMN2	SLC40A1	EDA	Z94160.1	MPV17L	MKX	MYO3A
RPS6	FLNA	TFPI2	ELFN2	AGBL5-IT1	MT1M	LINC01906	FA2H
RPS3	FKBP6	BDH1	AC073349.2	POPDC2	HTATIP2	LINC01285	AC103706.1
RPS27L	FIGNL1	GTF2H1	CLEC11A	RASA4	OAS2	PLEKHA7	PRDM9
RPL26	FIGN	ADAMTS9	AC092683.1	SNHG27	PLAC8	COL15A1	MTRNR2L4
RPL24	FGF8	VAMP5	GEM	NKD1	GRB10	DDO	TLL1
RPL23	FGF10	FP565260.1	GNRH2	SEMA5B	ZFY	NDRG2	AC022080.3
RPL10L	FES	NANOS1	LHX1-DT	LINC00431	ANO4	SLC40A1	PID1
RPA4	FEN1	SERPINH1	PCDHGA11	AC240565.2	KDM5D	TSLP	TIMM8AP1
RPA2	FBXW7	PWP2	AC074135.1	AL034399.2	SYT17	SLC9A4	ZNF311
RPA1	FBXW11	UCP2	AGAP7P	HOXA9	C4BPB	SAMD12-AS1	PTPRM
ROPN1B	FBXO5	OBSL1	SEMA4G	BICDL2	PBDC1	FTLP14	PRAL
ROCK2	FBXO43	WT1	AC004241.2	UNC5A	ZNF542P	AL359764.1	RPS26P39
ROCK1	FBXO31	LACTB2	AC090825.1	GOLGA6L5P	LIMCH1	CCDC85A	LINC01842
RNF8	FBXL8	SAMD9L	LINC01234	AC004461.2	CAVIN2	AL137003.1	ZNF790-AS1
RNF212B	FBXL7	TPRA1	LRP4	AL162274.1	OR5H5P	LINC00923	SMKR1
RNF212	FBXL6	EMC10	MIR5587	LINC00973	MGARP	CHMP1B2P	BCL2L10
RNF2	FBXL3	RIN2	HIF1A-AS1	SRPK3	SMIM10	SESN3	ST5
RNF112	FBXL22	RMI1	TRIM7	AL157394.1	CDH1	C7orf69	AC116903.2
RMI1	FBXL21P	PPM1H	ZBED6	TAS2R6P	CTNND2	PRR29	AVPR2
RMDN1	FBXL17	ASB1	SLC45A1	AC009299.3	PI3	AL391427.1	STAT5A
RIOK3	FBXL15	TGM2	ADAMTS9-AS2	NOTCH3	AIM2	HEPHL1	RNA5SP137
RIOK2	FBXL12	CDKN2B	IL1R2	MAP3K15	SIX2	P2RY2	KCNQ5
RINT1	FANCM	SPHK1	LINC02167	NUTM2E	DHX58	ANXA8L1	KCNK15
RIF1	FANCI	NR2F1	FAM183A	FYB2	SULF1	H19	RGS7
RIDA	FANCD2	CTSH	RHD	AL683842.1	MAP2K6	EVI2B	GIPC2
RHOU	FANCA	CDK14	GPR143	DUSP8P3	FAM198B	GATA2	NEFL
RHOC	FAM9C	DENND2A	QRFP	RGL4	FBP1	ABCG2	C8orf34
RHOB	FAM9B	EEFSEC	GRB14	DNLZ	FAM189A1	UGT3A2	CARD16
RHOA	FAM9A	PDGFRB	AC009533.2	AC093423.2	FRMPD4	CYP11A1	GABRR3
RHNO1	FAM83D	WTIP	AL121845.3	AL136366.1	C1QTNF1-AS1	LINC00491	AC006157.1
RGS2	FAM32A	SHISAL1	BTNL9	LRRC17	EDNRB	TSPAN12	IGFBP3
RGS14	FAM110A	DAPK1	AL161804.1	AC116447.1	SYNC	ZNF572	WDR64
RGCC	FAM107A	TMEM246	MNX1-AS1	AL162727.1	BVES	IQGAP2	NKX2-1
RFWD3	EZR	LEPR	CPN2	POM121B	PRTFDC1	ETNPPL	CHST4
RFPL1	EZH2	MAPT	AC120057.3	PADI4	ZNF43	C8orf31	SUSD2
REEP4	EXOC8	DUSP5	AC026412.3	AL773545.3	WDR17	YPEL3	LINC01686
REEP3	EXOC7	SEMA4B	AC099552.3	RAB11FIP5	LYPD6B	SARDH	SYTL5
RECQL5	EXOC6B	MEGF6	CASKIN1	FOXP4-AS1	CPPED1	DLX6	ZMAT4
REC8	EXOC6	ZFP36	MYRF	TRPM2	IDO1	FLG	BVES-AS1
REC114	EXOC5	TMC3	AC011912.1	AC108062.1	MSLN	SLC44A3-AS1	TARID
RCC2	EXOC4	MRPL23	PALM	ARL4C	ZNF354C	PAGE5	PRR35
RCC1	EXOC3	PAQR6	AC007952.4	AC084024.4	SEMA6D	DNER	SRP68P3
RCBTB1	EXOC2	PTGER4	SLC47A1P2	OR7E7P	ZSCAN18	AL807742.1	STARD13-IT1
RBM7	EXOC1	MT1F	FSBP	AC132153.1	CACNA2D1	ANKRD18B	C6orf132
RBM38	EXO1	NR4A2	TNFSF8	ACTBP13	RNF144B	TMEM98	KRT17
RBL2	EXD1	COL7A1	AC007906.2	RF00012	KIAA1217	ZNF788P	TPD52L1
RBL1	EVI5	NEDD4	CNTNAP3	FOXP3	PPP2R2B	LINC01615	AL589986.2
RBBP8	ESRRB	AC004556.1	ACTBL2	MIR6075	IFI27	AC245041.1	GPR27
RBBP4	ESPL1	BTN3A3	TREX2	AC005865.2	XAF1	LINC01825	LINC01121
RB1CC1	ESCO2	BRIX1	SLC35F4	AC097639.1	TENM3	AC005392.1	ANKFN1
RB1	ESCO1	TRIB3	LINC01629	LMO7-AS1	VGLL3	TP53I11	AL050303.4
RASSF4	ERH	ALDH2	CCDC200	VASH1	SNAP91	AC093627.4	RPSAP71
RASSF2	EREG	OASL	SULT2B1	RORA-AS1	MYEF2	SLC15A3	MARK1
RASSF1	ERCC6L	SFN	MYO7A	HCG25	P2RX6	ZDHHC1	LINC01336
RASA1	ERCC4	CSF1	ANGPTL4	AC006254.1	ENOX1	AC015522.1	MMP11
RAN	ERCC2	MELTF	CHD5	AC104695.3	TMTC1	BNIP3P17	FAM133A
RALB	ERCC1	IFITM1	TEX48	PPP4R1L	ALX1	TCIM	LINC01633
[illegible]	[illegible]	[illegible]	[illegible]	[illegible]	[illegible]	[illegible]	[illegible]

RAD9B	EPB41	ZFAND5	NR1I3	SHANK1	AC090152.1	MT1A	AC008063.1
RAD9A	EP300	CD47	CLDND2	ANKS6	OR2W3	VAMP5	RF00019
RAD54L	ENTR1	PLAU	AC133919.2	AC023886.1	VIT	DDIT4L	AC007495.1
RAD54B	ENSA	TEX41	KCNMB1	AC009090.5	SCN8A	CDYL2	AC091885.1
RAD51D	ENKD1	CLDN11	ALPG	AC020915.2	L3MBTL4	C1orf140	LINC00884
RAD51C	EML4	CLIP2	ID4	ANGPT4	LDLR	ETV7	HOXA11-AS
RAD51B	EML3	SEMA4C	PCDHB2	MACROD2	ACOT4	KCNJ12	ZMYND12
RAD51AP1	EML1	YES1	TGFB1	PSORS1C2	C10orf67	IL34	MIR3164
RAD51	EME2	LINC00665	HTRA3	SSBP3-AS1	UTY	CDH3	CCR3
RAD50	EME1	GDF15	HOXC-AS2	AL137013.1	ANGPT1	REPS2	SLC9A7P1
RAD21L1	EIF4EBP1	PHLDA3	RDH5	AL451064.2	ICAM2	GDNF	LINC01099
RAD21	EIF4E	PDE4D	PRKN	LINC01589	SEMA5A	AL133467.4	AC093249.2
RAD17	EIF2AK4	ARHGAP29	AL592295.4	BLACAT1	C5orf38	TINCR	AP000897.2
RAD1	EID1	NEXN	PINLYP	ZNF559-ZNF177	NUP210	GREM2	AL606469.1
RACK1	EHMT2	KRT19	AC004112.1	IGLV1-51	FAM155A	LINC01750	LPL
RACGAP1	EFHC2	MID1IP1	LVRN	CA15P1	LINC00664	ROR2	LINC02574
RABGAP1	EFHC1	GSTM4	DUXAP8	TM4SF19	TCEAL8	BATF2	TNFRSF14-AS1
RAB6C	EDNRA	BCL6	CASC8	PLD4	LTBR	AC005840.2	CR381653.2
RAB35	EDN1	ITPKB	THY1	AC010530.1	TXLNGY	RBM24	ELOVL3
RAB11FIP3	ECT2	TTLL3	NOG	HAVCR2	KRT19	NPR3	AL157937.1
RAB11A	ECRG4	QPRT	EFHB	U47924.3	RPS4Y1	SERPINB5	NYNRIN
PYHIN1	E4F1	LACC1	RF02038	AC026412.2	MID1IP1	AL034346.1	AL450992.2
PTTG3P	E2F8	ABCB1	AK4P3	GGN	ARMCX2	XK	H2AFZP4
PTTG2	E2F7	SMKR1	ZACN	FAM95B1	EPHA4	HS3ST3A1	AC098617.1
PTTG1	E2F6	ADAM19	AF131215.5	AC243562.1	PICSAR	AP005212.2	OR2L5
PTPRC	E2F4	TBX18	LINC01202	AC022509.3	SP5	CHST13	ADHFE1
PTPN6	E2F3	HSPG2	GBX2	HES7	CDH10	CEACAMP10	OR5H14
PTPN11	E2F2	CAPG	CLMN	DLG4	EIF1AY	OR10V3P	DNAJA4
PTPA	E2F1	DPYD	AC146944.4	PIANP	ABCA1	MAOB	TLR4
PTP4A1	DYRK3	CHPF	AADACL2-AS1	ATP1A3	BAMBI	HOXD4	OGDHL
PSRC1	DYNLT3	SAPCD2	AC144450.1	AC113383.1	TRIM29	IFI6	B4GALT4-AS1
PSMG2	DYNLT1	NFIL3	AC022028.2	TMEM240	SLC46A3	ZNF781	AL356275.1
PSME3	DYNC1LI1	C1QTNF6	SYT8	LAGE3P1	DSC3	LNCTAM34A	XKR8
PSMD13	DYNC1H1	MEGF9	GNG3	C15orf53	ARAP3	ST13P2	CLDN11
PSMC3IP	DUSP13	PSD4	ESRRB	SYP	RAC2	NOV	AC011447.3
PSMA8	DUSP1	ZNF880	AC025259.3	TMEM151A	EN1	GIMAP2	DGKG
PRR5	DTL	PDE4DIP	KLHL13	SLCO4A1-AS1	COL3A1	ZNF229	SCG2
PRR19	DSN1	NEGR1	AC090409.2	FLJ46906	GPX7	DAB2	NEXN
PRPF40A	DSCC1	CPM	ATP8B2	AL596220.1	LRRC37A6P	SLC2A3	RPL7AP60
PRNP	DONSON	NEU1	SNORD3B-1	PDE11A	KISS1	ACTN2	RNU2-69P
PRKDC	DNMT3L	CD109	GRM3	CIB2	SNCA	RGL3	RNU6-88P
PRKCE	DNMT3A	LY6E	AP000346.2	C2CD4C	ASS1	FOXF1	PLSCR1
PRKCD	DNM2	KCTD15	LINC02253	DCDC2	GPR1	USP32P2	CD274
PRKCB	DNA2	CSF2RA	AC211476.6	AC005387.2	MX1	MLXIPL	AL138767.3
PRKCA	DMTF1	NLRP2	AC126696.2	AL357079.1	BEX2	AP006565.1	AC089984.2
PRICKLE1	DMRTC2	SCML1	AC092868.1	TMEFF1	IFI44L	CXCL5	TUBB2BP1
PRDM9	DMRT1	PLEKHH1	SLC17A7	AC004233.1	SAA2	TAS2R1	AC103974.1
PRDM5	DMC1	CCDC14	AC078850.1	MAPK8IP1P2	MYLIP	IL24	RARRES3
PRCC	DLGAP5	COL12A1	VAV3	BMP6	CDKL2	LINC02056	PRKCQ-AS1
PRC1	DLG1	ZNF114	HCAR1	Z98048.1	EPDR1	MARC1	AP001528.1
PRAP1	DIS3L2	CELSR1	PTGS1	CIB4	BMP5	MYO1D	RLN2
PPP6C	DEUP1	C14orf132	ZMYND15	SMIM17	DCP1B	PCBP3	AC110285.1
PPP5C	DDX4	MUC1	CHRNA3	AC126755.6	SAMD5	DCLK1	AP000808.1
PPP3CA	DDX3X	AC011043.1	AC011450.1	AC010624.1	ZNF114	C3	RRN3P1
PPP2R2D	DDX12P	SPARC	ALOX5AP	AC023421.1	SLC12A8	AL160254.1	ARHGAP42
PPP2R1A	DDX11L8	ZNF415	DYSF	AC004264.1	ZNF257	AC005736.1	CST7
PPP2CB	DDX11	EVI2B	HSD17B13	PKDREJ	NLRP2	F3	ZNF415
PPP2CA	DDIT3	CAV2	PTPRU	AL021392.1	STXBP6	FILIP1L	SOX6
PPP1R1C	DDIAS	PXMP4	SMIM1	AP000437.1	LINC02302	KLHL34	TMEM158
PPP1R12A	DCTN6	PSMB9	CDHR2	SAMD13	POSTN	ISL2	GRIK2
PPP1CC	DCTN3	CALB2	ACTA2-AS1	FAM160A1-DT	ZNF486	SNX18P7	MAATS1
PPP1CB	DCTN2	SEH1L	NDST2	FBXO2	RSAD2	AC103858.2	SLC2A10
PPP1CA	DCTN1	DNAJB4	GLYCTK-AS1	CES3	DDX3Y	HOXC13-AS	AC069281.1
PPME1	DCLRE1A	CHKA	IMPDH1P8	AC009065.2	GYG2	RAET1E	AC061961.1
PPM1D	DCDC1	DNAJC21	AC008875.1	GPR75-ASB3	GGT5	GDNF-AS1	PDE1C
POLE	DBF4B	TPD52	ADM2	LGALSL-DT	RAB27B	SNAI2	GRAMD2B
POLDIP2	DBF4	ARNT2	AC092118.2	ADM5	CARD6	ADAM12	HS3ST5
POLA1	DAB2IP	STAT1	SPDYE21P	LINC01191	FAM174B	NMRAL2P	ECM2
POGZ	CYP27B1	PHGDH	HOXA-AS3	AP001363.1	HSD17B2	GNAO1	PCDH7
POC5	CYP26B1	MAP3K15	CX3CL1	CECR7	ADAMTS12	G2E3-AS1	AC007848.1
POC1B	CYLD	TBL1X	AC106795.2	AC103564.1	AC044810.2	AC079140.5	AL353653.1
PML	CUZD1	MYLIP	AC073389.3	PCDHGA10	PGBD5	FGF13	AC097478.1
PMF1	CUL7	GABRE	EVA1A	CLECL1	FLI1	GCNT1	MAP3K8
PLK5	CUL5	OSBPL10	GOLGA7B	AC016526.3	CXCL10	IKZF2	AQP11
PLK4	CUL4B	ERFE	CKLF-CMTM1	AC012615.4	ZC3HAV1L	RIPOR2	PRDM1
PLK3	CUL4A	JUP	SIRPG	AC027271.1	FOXQ1	LGI1	IFITM4P
PLK2	CUL3	VEGFA	ALOXE3	BEAN1-AS1	NTF3	TMEM173	C1QTNF6
PLK1	CUL2	FBXO41	AC025171.5	ASS1P10	COPZ2	NPY4R	RAMP2
PLEC	CUL1	C1orf21	MT-TS1	AC138473.1	LAMA1	GLRX	PSG4
PLD6	CTNNB1	CMYA5	RF02039	CHAC1	DTNA	PCDHGB1	CALR4P
PLCB1	CTDP1	BCAR3	CCDC62	CDKN1A	ATXN1	AC002525.1	NKAIN3
PKN2	CTDNEP1	PTPRG	AL079301.1	SLCO4A1	CCDC144B	ANKRD30BP1	PDE4A
PKMYT1	CTCFL	MT1E	ZFPM2-AS1	PLEKHG2	HAGLR	HLA-DPA1	AL031667.2
PKHD1	CTCF	SVIP	PLEKHD1	MTHFD2P1	MEST	GADD45G	PDE7B
PKD2	CSNK2A2	PCDH7	INHBA-AS1	AC099548.2	ACSS3	FENDRR	SV2A
PIWIL4	CSNK2A1	STON2	GPAT3	EFCAB14-AS1	STON2	SLFN13	SOD3
PIWIL3	CSNK1D	PAX2	MT-TW	GGT6	PIEZO2	PDCD1LG2	NUAK1
PIWIL2	CSNK1A1	ZNF841	AL390719.2	FNDC8	CPA3	LAMA4	AC073848.1
PIWIL1	CROCCP3	OGDH	PTK7	DPP9-AS1	RARRES1	AP001094.2	C8orf88
PINX1	CROCCP2	HCN2	AL117335.1	GTF3C2-AS1	LIN28B	CAPSL	PLPPR4
PIN1	CROCC	CLEC2B	CICP16	F2	PCDHGB4	OR10V2P	AC011447.6
PIMREG	CREBL2	TFRC	GOLGA8K	DISP3	FBN2	PLXNA2	SPATA9
PIM3	CRADD	GPT2	ID2	AP006222.1	TMC3	EPHA5	DMTN
PIM2	CORT	RRM1	TPO	LINC01510	TUB	FAM198B-AS1	PPFIBP2
PIM1	CNTROB	PAX8	AC111000.2	TMEM63C	MXRA5	DDX18P3	AC007405.1
[illegible]	[illegible]	[illegible]	[illegible]	[illegible]	[illegible]	[illegible]	[illegible]

PIBF1	CNTD1	MMP2	LSR	HOXB8	MMP2	PLPPR5	AC110285.7
PHGDH	CLTCL1	MACROD2	AC139795.1	KCNB1	BMPER	MAMDC2	FAT3
PHF8	CLTC	CTSL	AP000526.1	PSD3	MAOA	LINC00842	SH3GL2
PHF13	CLTA	ZNF844	AL139220.2	RASGEF1C	HRASLS	CLEC7A	SNORA2B
PHB2	CLSPN	RRAGD	OTUD7A	SH2D3A	PSG11	RNLS	KCNJ8
PELO	CLIP1	NDRG2	AC097059.1	AC145098.1	L3MBTL1	SNAP25	AL031055.1
PDS5B	CLASP2	RGS17	SRRM3	SPHK1	PTPRZ1	WIPF1	GALM
PDS5A	CLASP1	HSPH1	AC127521.1	GUSBP3	NPAS3	KRT8P3	NWD2
PDIK1L	CKS2	AP000577.1	AP000692.2	MTDHP1	SLC4A8	VIPR1	PTPRR
PDE4DIP	CKS1B	SNHG4	AC008687.4	AC079781.5	CARD11	FRAS1	PLCB2
PDCD6IP	CKAP5	TSPAN5	RNF128	AC008610.1	USP32P1	PLEK2	RPL21P54
PDCD2L	CKAP2	FAR1	SEPT5	AL110115.1	PNMT	AL356274.2	CLIC3
PCNT	CIT	OAS3	FOXD4L1	AC005746.1	AC015909.1	TMED10P2	WFDC1
PCNP	CINP	AC245041.1	LINC00337	LINC01607	SERPINB9	CHGA	ZEB2
PCNA	CIB1	ZFPM2-AS1	CECR2	AC023790.2	ENPP5	AMPH	DDX60L
PCM1	CIAO2B	WSCD1	AC016573.1	AC091181.1	GPR173	LPAR3	LINC00278
PCLAF	CIAO2A	AL365203.2	FP325330.3	AC018521.6	RCAN2	LACC1	PRDM13
PBX1	CIAO1	OSGIN2	AC003984.1	AC233723.1	TSPAN8	CXXC4	C3orf58
PBRM1	CHTF8		SMIM10L2A	AC104461.1	RORB	AC020951.1	KLHL3
PBK	CHTF18		AC007686.4	SNED1	GABRA3	C4orf19	LINC01695
PAX6	CHORDC1		AC141930.1	AC092611.2	DUSP4	EYA4	VEGFC
PARD6G	CHMP7		TTC6	DGCR9	MYT1L	GPR85	PLPP3
PARD6B	CHMP6		CYP4F11	PROX2	ARHGAP40	PRSS3	LINC01443
PARD6A	CHMP5		COL28A1	PLEKHH1	LRMDA	GATA6-AS1	LINC01998
PARD3B	CHMP4C		BATF3	RPL23AP87	AC103770.1	ALDH3B1	FBLN2
PARD3	CHMP4BP1		HIST3H2BB	AL162258.2	SFRP1	TENT5B	TRPM6
PAK4	CHMP4B		AL158166.2	PFN1P2	PLCXD3	BEX5	AC007546.1
PAFAH1B1	CHMP4A		AC009090.4	ABALON	FOXL2	ADAMTSL1	RPL12P11
PABIR1	CHMP3		GRIN3B	AC122134.1	RTL8B	NMNAT3	AC116351.1
P3H4	CHMP2B		AC233280.19	AL627230.2	CASC9	AC089983.1	EHD4-AS1
OVOL1	CHMP2A		GLI1	AQP7P1	GPR37	AP000221.1	RARB
OSGIN2	CHMP1B		NXPH4	FCER1G	KRT8	FRK	CEACAM1
ORC4	CHMP1A		AC091563.1	C11orf91	HNMT	DPP4	EPHB6
ORC1	CHFR		IL11	IL36G	FOXE1	PCA3	GPLD1
OOEP	CHEK2		FP565260.1	PFKFB3	SLCO3A1	MAFB	DGAT2
OIP5	CHEK1		CYCSP10	AC004801.6	RTP4	POU4F1	LINC02076
OFD1	CHD3		CCL20	SNTB1	ZNF532	ZNF785	ZNF350-AS1
ODF2	CHAMP1		ANO7	ISM1	C14orf132	FGF12	LINC00899
NUSAP1	CHAF1B		MDGA1	AC046185.3	C17orf51	LINC01983	G0S2
NUPR2	CHAF1A		TMEM191C	NUPR1	GLMP	AC008969.1	IGFBP5
NUP88	CGRRF1		ADORA1	AC073111.1	GFRA1	AC009977.1	NOL4L
NUP62	CGREF1		MERTK	KCNJ11	SSTR1	FCMR	SMTNL1

Supplementary table 5 The proteins pulled down by CLIC3 in J82-MS and TCCSUP-MS.

J82-MS

Accession	Descripti	Coverage	# Peptide	# Unique	# AAs	MW [kDa]	calc. pI	Abundances (Grouped): CLIC3-IgG	Abundances (Grouped): CLIC3-IP
A0A8I5KWT	Myosin-9	62	140	118	1981	229	5.64	13614671458	35421890167
P35580	Myosin-1C	59	140	2	1976	228.9	5.54	9863593143	30925806119
A0A8I5KZ3	Myosin-1C	59	139	1	1986	230	5.57	811629.2941	3215501.75
Q01082	Spectrin	53	110	101	2364	274.4	5.57	453145913.6	2327328413
Q7Z406	Myosin-14	55	104	56	1995	227.7	5.6	2118114669	5783881210
Q92614	Unconvent	41	76	39	2054	233	6.3	511502344.6	2949009745
O43707	Alpha-act	64	52	38	911	104.8	5.44	1041340779	1872186064
A0A384P5S	Epididymi	43	95	95	2452	282.1	5.34	279452682.7	977972215.6
Q4VCS5	Angiomoti	52	50	47	1084	118	7.64	3138330442	3991677682
P35908	Keratin,	77	47	32	639	65.4	8	27175788840	9924938881
B9EGI2	Myosin ph	58	52	3	1037	118	6.39	64608147.39	60568742
P35527	Keratin,	78	36	35	623	62	5.24	11402479094	3778653475
A0A494BZV	Myosin ph	24	53	4	2425	274.2	5.6	822567383.3	974191768.5
A0A087X0K	Tight jun	51	63	5	1768	197.3	6.67	347454342.9	979253252.6
B2RMV2	Cytospin-	47	52	52	1117	124.5	5.76	1017609247	1076677960
Q9H0A0	RNA cytid	45	43	43	1025	115.7	8.27	587441203.7	8413065837
H6VRF8	Keratin,	60	45	38	644	66	8.12	33835463598	12799850435
A0A6I8PIX	Supervill	39	56	22	1904	213.5	7.28	282271661.2	441577939.5
Q16643	Drebrin (	52	35	7	649	71.4	4.45	6412387149	6813378317
O94832	Unconvent	54	55	55	1006	116.1	9.39	1301218792	1944829813
Q6MZU1	Uncharact	49	60	2	1692	189.5	6.8	11399119.21	20026387.75
P21333	Filamin-A	34	68	62	2647	280.6	6.06	95446039.04	344440234.3
M0QY43	Myosin-14	54	46	2	1002	113.7	7.59	24539412.98	94241760.13
P15924	Desmoplak	30	83	83	2871	331.6	6.81	656318256.4	311647282.2
Q9BZF9	Uveal aut	49	69	69	1416	162.4	7.03	608131941.1	636022889
A7BI36	p180/ribo	41	52	52	1540	165.6	8.97	310266292.8	549131885.3
A0A087WYQ	Spectrin	30	57	48	2397	271.5	6.23	65649372.49	349098831.6
A0A1B0GVI	Keratin,	56	37	29	624	63.3	5.26	30505683789	10893295970
O14974	Protein p	51	51	50	1030	115.2	5.4	799615556.6	909986511.8
A5YM63	Neurofila	50	41	39	877	98.3	4.86	919575582.7	919197823.6
Q9UHB6	LIM domai	60	41	41	759	85.2	6.84	1890447291	1968515506
A0A804HII	Alpha-act	49	42	28	913	105.4	5.39	169886161.1	247867425.7
B4E0T8	cDNA FLJ5	36	38	1	1181	133.5	7.09	2568416.038	11382006
A0A024R3T	Poly [ADP	42	40	40	1014	113	8.88	230589865.8	533565517.3
Q9P2M7	Cingulin	45	48	48	1203	137	5.52	357496235.6	381680457.8
Q9P0K7	Ankycorbi	49	43	43	980	110	6.21	448380823.9	363127574.2
D6R9W4	Drebrin (	63	29	1	317	36.4	5.14	9434367.096	15696416
H3BUF6	Ataxin-2-	39	32	1	1068	112.6	8.69	3417468.479	5248249.5
Q8WWM7	Ataxin-2-	38	33	2	1075	113.3	8.59	1212154826	770644579
Q9BQG0	Myb-bindi	32	41	1	1328	148.8	9.28	329971859.5	1309424201
I3L1L3	Myb-bindi	34	41	1	1252	140.2	9.26		1198596
B4E3H1	cDNA FLJ5	38	36	2	1128	127.3	6.73	4120259.351	6623540.75
P13647	Keratin,	56	40	12	590	62.3	7.74	3010736487	1151141212
P46940	Ras GTPas	33	46	43	1657	189.1	6.48	162832574.6	329350879
P02538	Keratin,	56	38	2	564	60	8	834321010.2	585131663.1
P02533	Keratin,	63	32	12	472	51.5	5.16	3041366369	1353502389
P04259	Keratin,	56	37	1	564	60	8	35722217.67	29991413
Q86V48	Leucine z	45	41	41	1076	120.2	8.5	222130640	280218174.9
A0A7I2V4F	ATP-depen	51	33	33	734	81.7	7.33	555021571.9	225503634.1
Q96SB3	Neurabin-	43	27	25	817	89.3	4.97	646461996.7	657619356.4
B2R853	cDNA, FLJ	54	37	1	564	60	7.72	34671429.35	32598907.5
E9PHV5	Protein I	35	35	35	1237	136.1	5.2	417918102.7	341806824.5
Q9BY89	Uncharact	32	40	40	1806	196.6	8.47	29966680.34	124690747.3
Q08211	ATP-depen	26	28	28	1270	140.9	6.84	249663751.5	386425383.6
F8WE88	Unconvent	27	47	42	1855	215.2	8.51	120870820.1	156719952.2
A0A804HK7	Filamin-B	21	44	38	2562	273.9	5.73	29786023.23	97208440.5
P08779	Keratin,	62	28	6	473	51.2	5.05	289587941.9	221851518.4
Q8NI35	InaD-like	24	33	7	1801	196.2	4.94	57698854.79	156746880.7
Q53G99	Beta acti	58	16	9	375	41.7	5.59	2252299671	2242272680
O14654	Insulin r	34	28	28	1257	133.7	8.44	175113213.9	342740198.8
Q86YZ3	Hornerin	21	21	21	2850	282.2	10.04	565980750.6	135743617.6
Q9NR30	Nucleolar	44	31	29	783	87.3	9.28	252386678.3	511735158.2
E9PMS6	LIM domai	35	36	36	1275	145.3	7.3	87871263.31	113106659.1
Q15149	Plectin (	12	51	51	4684	531.5	5.96	62538678.99	99895078.72
Q9Y2W1	Thyroid h	31	28	20	955	108.6	10.15	1392915527	557082970.4
O00159	Unconvent	31	31	5	1063	121.6	9.41	164936646.5	266026292.7
E7EX73	Eukaryoti	30	36	31	1436	158.5	5.21	66942241.86	272077448.3
B4DKV4	cDNA FLJ6	54	30	1	526	55.7	8.43	8745951.278	5090769.5
P14923	Junction	50	29	24	745	81.7	6.14	342637798.2	175122623.3
A0A024R5M	Nuclear m	20	36	36	2101	236.4	5.8	38285722.44	148779147.9
Q00839	Heterogen	31	26	11	825	90.5	6	1743604339	1051164443
F4ZW66	NF110b OS	33	25	19	898	95.7	8.81	207511382.3	483798008.9
Q7Z417	FMR1-inte	42	22	22	695	76.1	8.7	523705771.4	298752992.5
A0A8V8TQK	Clathrin	25	37	37	1651	188.8	5.67	111106588.5	124276284.1
B2RBS8	cDNA, FLJ	52	30	30	609	69.3	6.28	1764339881	692086981.8
P07814	Bifunctio	28	32	32	1512	170.5	7.33	76668554.71	156602499.8
H0YAR2	Polyadeny	43	21	15	599	66.6	9.28	199123899.1	217424760.1
A0A8C8NWT	Pleckstri	30	28	28	1340	147.5	5.49	95532340.15	160180416.1
D3DTH7	Myosin IC	33	27	1	862	98.9	9.42	6893635.006	14824432.25
J3KN01	Afadin OS	22	33	1	1816	205.5	6.29	59467284.78	117074889.8
E7ETY2	Treacle p	21	28	5	1488	152.2	8.85	317531252	355313733.2
A8MQ02	Afadin OS	23	33	1	1781	201.7	6.51	748557.9436	1309507.25
E9PDF6	Unconvent	33	33	33	1107	128.4	9.31	152501487.5	225632823.3
B4E2N5	cDNA FLJ5	29	27	1	1249	137.2	4.79	26007394.9	7199811
Q02413	Desmoglei	28	20	20	1049	113.7	5.03	185866067	76351297.53
P08670	Vimentin	59	28	26	466	53.6	5.12	463649168	709351631.4
A0A669KB8	SCRIB ove	29	36	29	1658	177.7	5.14	50790528.09	135456343.3
O43290	U4/U6.U5	43	26	26	800	90.2	6.13	79191231.2	125860457.5
A0A2X0SFB	ARHGEF11	27	30	13	1524	167.8	5.5	52567734.74	90625907.25
E9PHY5	Band 4.1-	35	25	16	935	104.3	5.39	114859704.1	158838417.9
Q14126	Desmoglei	32	25	25	1118	122.2	5.24	188647120.4	216204993
Q9NYF8	Bcl-2-ass	28	23	22	920	106.1	9.98	526733867.6	261950013.8
Q9UPN4	Centrosom	32	30	30	1083	122.1	8.69	85155422.24	124101826
A0A1Y0BRN	Unconvent	26	29	29	1272	146.9	8.56	70478373.7	128121174.4
P52272	Heterogen	40	28	13	730	77.5	8.7	150952112.4	400442313.9
P36578	60S ribos	43	17	17	427	47.7	11.06	468896465.2	953490901.4
Q14247	Src subst	42	20	20	550	61.5	5.4	232679435	305713293.1
O75643	U5 small	16	31	31	2136	244.4	6.06	22606887.57	90873577.75
O43166	Signal-in	18	25	5	1804	199.9	8.19	34630196.84	88450509.06
A0A6I8PRA	Treacle p	25	27	4	1240	125.2	7.93	6242880.79	10244288.75
Q14573	Inositol	14	31	27	2671	303.9	6.48	43762396.85	85264782.78
Q9ULV0	Unconvent	19	32	27	1848	213.5	7.2	42772150.43	49161673.88
P67809	Y-box-bin	50	11	7	324	35.9	9.88	248956832.9	268993454
Q7Z2W4	Zinc fing	30	20	20	902	101.4	8.4	19353394.75	73719753.75
A0A024RD8	Heat shoc	38	23	12	724	83.2	5.03	135538124.8	123399367.9
Q6FI03	G3BP prot	42	14	13	466	52.1	5.59	16101529.91	93117379.19
Q86UP2	Kinectin	26	31	31	1357	156.2	5.64	80440213.13	111430329.1
Q01804	OTU domai	28	23	23	1114	124	6.71	116756538.9	48580346.63
A0A024RDB	Ras-GTPas	44	15	4	449	50.8	5.38	175227128	304060394.8
A0A024QZW	Nucleopor	22	21	21	1475	153.9	8.73	26255124.38	65002282.63
Q92804	TATA-bind	33	13	11	592	61.8	8.02	1691209630	300098772.3
A0A1U9X98	PRRC2A OS	18	25	23	2157	228.7	9.45	63407532.39	103970800.8
B3KY60	cDNA FLJ1	31	19	19	802	92.2	8.18	53917812.25	146616275
A0A5H1ZRQ	RNA helic	40	25	18	731	80.4	8.37	189163952.2	336772556.4
A0A804HL4	Leucine-r	21	31	31	1393	157.7	6.18	72250798.83	109135767.1
P19338	Nucleolin	30	24	24	710	76.6	4.7	327786715	795772152.5
O00571	ATP-depen	40	24	23	662	73.2	7.18	311384111.1	241223108.3
A0A0A0MRA	Band 4.1-	32	21	2	918	102.2	5.31	43437507.37	66710599.31
P05787	Keratin,	41	24	15	483	53.7	5.59	120851494.1	134226149.1
A0A7I2V53	Non-POU d	35	17	16	501	57.6	9.33	280605627.1	220360462.3
O60292	Signal-in	19	27	27	1781	194.5	8.32	17310864.82	73007456.94
B3KY28	cDNA FLJ4	35	20	1	756	84.6	5.44	1020910.934	1625929
P23246	Splicing	31	21	20	707	76.1	9.44	750131196.4	578113980.3
A8K6U7	cDNA FLJ7	25	19	19	856	95.6	6.92	134892626.2	154828526.5
A0A7I2YQJ	Cytospin-	33	27	26	1029	114.1	6.87	918599334.4	788733765.4
Q5JTH9	RRP12-lik	24	25	25	1297	143.6	8.75	73251275.49	181302252.6
Q96PK6	RNA-bindi	33	24	24	669	69.4	9.67	350569917.7	294881532.3
P11387	DNA topoi	30	23	23	765	90.7	9.31	94364719.82	160961406.1
B4DYX7	cDNA FLJ5	24	21	1	1257	139.4	8.18	529648.6613	3579833.5
Q6PKG0	La-relate	28	21	21	1096	123.4	8.82	71008674.21	135023457.8
Q9NZB2	Constitut	21	19	19	1118	121.8	8.88	34126611.87	60978547.5
A0A024R2Z	Guanine n	36	20	20	537	60.5	8.79	75737571.05	368691234.5
P53621	Coatomer	23	25	25	1224	138.3	7.66	41090650.32	97997622.66
O43390	Heterogen	35	21	15	633	70.9	8.13	119167169.4	159629281.5
Q5SW79	Centrosom	18	23	23	1584	175.2	7.11	34922894.11	86307438.25
B2R8R5	cDNA, FLJ	27	18	18	835	88.5	5.77	85312002.32	118517580.5
Q8IY37	Probable	20	21	21	1157	129.5	8.1	25435934.05	96060202.88
A0A024R7L	UPF1 OS=H	26	23	23	1118	123	6.68	53907985.93	59197175.41
Q5T9A4	ATPase fa	33	19	8	648	72.5	9.2	309916691.4	462822588.3
P05023	Sodium/po	21	19	19	1023	112.8	5.49	57797656.31	75547247.88
H0YI76	Keratin,	77	18	1	201	23.1	4.97	2855963.152	1814984.688
A0A024R23	Tight jun	23	25	25	1190	133.9	7.4	68477210.48	86520019.59
Q32MZ4	Leucine-r	30	20	19	808	89.2	4.65	80337045.2	85025728.5
Q14444	Caprin-1	26	14	14	709	78.3	5.25	1495220403	866404291.9
J3KTA4	Probable	35	21	14	614	69	8.85	158035346.3	271205049.4
Q8IY63	Angiomoti	26	20	17	956	106.5	7.11	54727635.26	59170530.22
Q13045	Protein f	19	22	10	1269	144.7	6.05	67786009.22	84508112.13
B2R8Z8	cDNA, FLJ	33	18	12	623	69.6	8.76	45489622.9	73089015.69
Q9UKV3	Apoptotic	19	20	20	1341	151.8	6.43	97776374.75	72844602
Q8N1N4	Keratin,	38	19	16	520	56.8	6.02	171291535.2	46831288.38
P07900	Heat shoc	29	18	8	732	84.6	5.02	19973396.99	15074611.94
Q4KMQ1	Taperin (	32	15	15	711	75.5	7.25	57584056.63	75734408.5
A0A7I2V5Y	Kinesin-1	24	19	19	974	111.6	8.5	42001680.1	68142998.81
Q04695	Keratin,	42	20	6	432	48.1	5.02	84030749.59	65317719.25
Q6IQ30	Polyadeny	33	22	16	660	72.3	9.35	115477686	130357209.4
Q86UE4	Protein L	27	16	15	582	63.8	9.32	57003854.13	128176205.9
A0A8I5KVB	General t	21	19	7	1118	126.5	9.07	58279523.56	58542319.75
P62424	60S ribos	45	16	16	266	30	10.61	276355300.3	422907801.9
Q8TBR3	Fusion (I	20	9	7	526	53.4	9.38	742044146.2	327406771
Q96IR1	RPS4X pro	55	14	14	243	27.2	9.94	194028068	251371165.4
Q7Z794	Keratin,	31	19	15	578	61.9	5.99	194444419.5	78950774.09
P11142	Heat shoc	30	16	8	646	70.9	5.52	65728431.33	68639841.03
A0A0S2Z4G	Tropomyos	58	20	13	248	29	4.78	425482728.5	443626830.9
B4E2K2	cDNA FLJ5	29	18	2	942	103.5	5.07	46161441.79	54876178.19
Q9NVI7	ATPase fa	27	16	5	634	71.3	8.98	55938318.78	64191897.13
Q5D862	Filaggrin	13	16	16	2391	247.9	8.31	198247169	52079932.81
Q5T749	Keratinoc	32	10	10	579	64.1	8.27	193307658.8	84625466.66
Q9Y3I0	RNA-splic	42	18	18	505	55.2	7.23	134592477.8	131289972.6
B3KT06	Tubulin a	38	13	1	416	46.3	5.14	3593818.233	9503862

TCCSUP-MS

Accession	Descripti	Coverage	# Peptide	# PSMs	# Unique	# AAs	MW [kDa]	calc. pI	Abundances (Grouped)	Abundances (Grouped): CLIC3_Ip
P35527	Keratin,	79	38	110	38	623	62	5.24	287500327.8	185612516.4
Q9P2E9	Ribosome-	44	55	108	19	1410	152.4	8.6	37675084.86	150280132.8
H6VRF8	Keratin,	62	46	118	39	644	66	8.12	562738621.8	462299399.5
Q9H0A0	RNA cytid	48	41	89	20	1025	115.7	8.27	31847773.13	426496322
A1A5C5	RRBP1 pro	57	37	77	1	751	84.3	5.01	198366.0313	729966.273
B7Z597	60 kDa he	49	17	53	17	564	60	5.74	22992468.12	1137342.531
Q08211	ATP-depen	38	35	68	35	1270	140.9	6.84	29804611.31	33665539.33
Q00839	Heterogen	41	25	53	25	825	90.5	6	39222620.68	68013817.03
P35908	Keratin,	64	34	82	25	639	65.4	8	104111760.8	104675998.8
A0A1B0GVI	Keratin,	54	29	77	26	624	63.3	5.26	171506889.5	204717921.4
A0A8I5KWT	Myosin-9	27	43	50	33	1981	229	5.64	11006767.97	1098064.967
Q9BQG0	Myb-bindi	34	37	55	37	1328	148.8	9.28	8138475.418	28621179.3
P13639	Elongatio	36	15	24	15	858	95.3	6.83	15596770.41	298473.3088
A0A024R3T	Poly [ADP	40	29	43	29	1014	113	8.88	6781882.623	12057648.79
Q05CW7	NAT10 pro	45	22	49	1	554	62.3	9.26		55088.72472
P07900	Heat shoc	37	17	29	10	732	84.6	5.02	18704565.7	238133.2831
P23246	Splicing	39	22	56	21	707	76.1	9.44	181598270	283979975.2
Q86UP2	Kinectin	30	31	42	31	1357	156.2	5.64	9685976.431	26523348.09
Q86YZ3	Hornerin	20	18	39	18	2850	282.2	10.04	7974162.355	8517590.304
A0A8V8TQ1	Clathrin	17	15	20	15	1680	192.1	5.69	3252083.732	106224.6541
P78527	DNA-depen	8	12	16	12	4128	468.8	7.12	3177840.117	179857.587
A8JZY9	Tubulin a	55	14	20	3	451	50.1	5.06	10246409.59	537898.5089
A0A024RD8	Heat shoc	27	11	23	3	724	83.2	5.03	10473434.94	80787.61309
Q2TSD0	Glycerald	47	7	14	7	335	36	8.46	9615563.297	169136.6922
P13010	X-ray rep	33	10	18	10	732	82.7	5.81	7161128.553	169155.4122
A0A0G2JIW	Heat shoc	34	12	22	6	642	70.1	5.66	4942561.492	1149854.233
Q53GA7	Tubulin a	48	12	18	1	449	49.8	5.14	141187.9531	
P22314	Ubiquitin	25	11	16	11	1058	117.8	5.76	3743823.586	
Q14974	Importin	29	9	13	9	876	97.1	4.78	3780522.82	
P08779	Keratin,	53	22	44	11	473	51.2	5.05	24244572.88	12617789.77
P02538	Keratin,	47	28	50	4	564	60	8	21558382.36	13052509.98
A0A5F9ZHM	L-lactate	47	9	17	8	341	37.4	6.25	5237497.977	14918.16688
A0A7I2V53	Non-POU d	44	19	40	17	501	57.6	9.33	21880130.27	39912403.79
Q8N1C8	Stress-70	27	13	20	12	681	73.8	6.37	29241044.12	18760576.47
Q53HQ7	Elongatio	47	11	20	11	462	50.2	8.94	20068962.79	865546.8477
P11142	Heat shoc	33	12	21	9	646	70.9	5.52	11645686.38	706885.5013
A0A024R7L	UPF1 OS=H	24	21	38	21	1118	123	6.68	3121840.301	3493204.655
Q53G99	Beta acti	41	10	20	9	375	41.7	5.59	13409413.65	1725438.437
P04259	Keratin,	45	27	49	3	564	60	8	1657148.25	744719.2282
P07814	Bifunctio	22	22	31	21	1512	170.5	7.33	1855357.176	2825237.266
Q9Y2W1	Thyroid h	23	18	39	11	955	108.6	10.15	21107234.16	35393309.95
P63241	Eukaryoti	50	6	13	6	154	16.8	5.24	11603291.71	
A0A384NYT	Tubulin b	54	11	17	5	442	49.4	4.89	14729063.77	176771.0866
P13647	Keratin,	38	24	48	13	590	62.3	7.74	19039980.89	19814307.39
A0A2R8Y6G	phosphopy	38	11	19	11	434	47.3	6.99	13577999.63	
P15924	Desmoplak	11	26	38	26	2871	331.6	6.81	4290008.867	3632365.131
P78371	T-complex	32	9	12	9	535	57.5	6.46	2173915.066	119875.5087
O14654	Insulin r	23	17	28	17	1257	133.7	8.44	2216904.099	8421793.709
P02533	Keratin,	55	21	37	8	472	51.5	5.16	6684723.102	6050684.15
Q9NR30	Nucleolar	31	18	27	17	783	87.3	9.28	5796080.273	7410089.23
B2R9K8	cDNA, FLJ	40	9	10	9	531	57.9	6.8	1997685.5	
P12277	Creatine	61	11	14	11	381	42.6	5.59	3401578.205	84307.96112
P29401	Transketo	28	7	9	7	623	67.8	7.66	6231561.189	
O60841	Eukaryoti	21	17	23	17	1220	138.7	5.49	1155786.856	4939057.21
B3KM80	Nucleolin	29	14	18	14	536	58.5	4.67	8460097.426	8126967.066
Q9NYV4	Cyclin-de	15	16	23	15	1490	164.1	9.44	5646330.079	6310457.566
Q14444	Caprin-1	24	11	21	11	709	78.3	5.25	30822924.84	43576095.62
A0A7I2V32	Heterogen	33	10	15	9	333	35.2	8.87	10448987.16	1339568.486
P35580	Myosin-1C	11	14	17	7	1976	228.9	5.54	249572.7285	
Q3B7A7	Trifuncti	16	8	9	8	1010	107.7	6.77	634690.6992	
A0A7P0TAI	78 kDa gl	30	10	17	9	616	68.1	5.14	2221296.883	717035.94
Q8WWM7	Ataxin-2-	25	18	27	18	1075	113.3	8.59	10751469.36	19212875.38
P08670	Vimentin	26	9	12	9	466	53.6	5.12	8039421.133	133308.9797
P39023	60S ribos	27	8	15	8	403	46.1	10.18	5660117.348	3103829.848
A0A3B3ITJ	Heterogen	23	5	8	5	537	59.2	7.11	4703020.797	120530.195
B2RDN9	cDNA, FLJ	22	6	10	6	609	69.7	6.7	2097642.219	
P04350	Tubulin b	45	8	10	2	444	49.6	4.88	482967.5	
P36578	60S ribos	38	16	24	16	427	47.7	11.06	3590453.027	18371075.85
P27816	Microtubu	18	14	19	14	1152	120.9	5.43	1861230.746	3336916.62
P00338	L-lactate	33	7	9	6	332	36.7	8.27	2770756.906	
Q12797	Aspartyl/	25	15	20	15	758	85.8	5.01	2784811.133	14504939.91
Q58EY4	SWI/SNF r	22	15	19	11	1105	122.8	5.72	631627.5	4684561.793
P06576	ATP synth	23	5	7	5	529	56.5	5.4	7114609.063	
Q8TBR3	Fusion (I	21	8	17	6	526	53.4	9.38	7950952.393	14430303.22
J3KTA4	Probable	32	18	27	13	614	69	8.85	8026905.223	11790871.61
Q9NYF8	Bcl-2-ass	14	12	26	11	920	106.1	9.98	4992543.305	13898517.36
A0A8J9BZL	Golgin su	22	16	22	16	1029	116	5.05	1226569.699	5060238.964
E9PCA1	T-complex	30	6	9	6	520	57.1	5.55	804138.9141	19063.54256
E7EX73	Eukaryoti	12	12	18	12	1436	158.5	5.21	3177726.977	1012025.651
A0A0S2Z4G	Nucleopho	29	5	12	5	265	29.4	4.61	6101973.105	341431.345
A0A5H1ZRQ	RNA helic	26	14	22	9	731	80.4	8.37	1814510.676	3191580.555
Q96PK6	RNA-bindi	26	15	28	15	669	69.4	9.67	3259958.844	7235082.413
B3KY60	cDNA FLJ1	22	12	14	12	802	92.2	8.18	458481.3281	2546629.825
B2R5U1	Staphyloc	22	12	18	12	885	99.6	6.96	1199943.064	1320306.923
F8W6I7	Heterogen	41	11	14	10	307	33.1	9.13	10224743.39	7986115.467
Q01804	OTU domai	23	17	27	17	1114	124	6.71	1980452.441	4177315.319
A0A024RDB	Ras-GTPas	35	10	19	3	449	50.8	5.38	6295676.527	3998407.419
P04844	Dolichyl-	26	6	7	6	631	69.2	5.69	575219.457	
Q9NVI7	ATPase fa	29	15	25	9	634	71.3	8.98	3558913.633	18122388.11
Q99832	T-complex	29	7	8	7	543	59.3	7.65	2241127.406	
Q9NZB2	Constitut	23	19	31	1	1118	121.8	8.88	202935.1719	222500.6321
A0A8V8TNY	Constitut	23	19	31	1	1117	121.8	8.88	4104182.875	3422964.546
P26641	Elongatio	36	6	9	6	437	50.1	6.67	4595389.125	
A8K6U7	cDNA FLJ7	23	13	18	13	856	95.6	6.92	4529135.729	4598550.335
B4DJ30	cDNA FLJ6	13	5	6	5	995	112.9	6.06	3293166.406	
H3BPK7	Alanine--	15	6	8	6	999	110.1	5.67	1734928.074	
Q7Z2W4	Zinc fing	25	16	21	16	902	101.4	8.4	914850.4512	2485770.756
P68431	Histone H	29	2	7	1	136	15.4	11.12	124663160.8	342709.6468
A8MXP9	Matrin-3	20	12	20	12	895	99.9	6.04	2434402.447	2612383.689
A0A7G6J4N	Ubiquitin	19	11	19	11	802	87.5	5.27	2834800.828	3662600.072
A0A8I5KRC	Core hist	23	5	8	5	343	36.5	9.95	2279092.563	
Q92667	A-kinase	20	13	22	13	903	97.3	4.94	1104003.369	3659650.211
O00571	ATP-depen	28	16	27	15	662	73.2	7.18	2675012.73	4243793.574
A0A7I2V4F	ATP-depen	31	16	22	16	734	81.7	7.33	2545028.807	2683708.809
P49327	Fatty aci	5	5	6	5	2511	273.3	6.44	427530.543	
A8K6Y1	cDNA FLJ7	23	5	6	5	367	40.9	6.4	4143174.426	
B2R8R5	cDNA, FLJ	25	12	16	12	835	88.5	5.77	603108.4727	4660173.918
A0A7I2V64	Polyadeny	25	13	22	9	590	65.1	9.36	2678863.27	4075769.476
E5RH77	40S ribos	40	4	7	4	134	14.3	9.52	2638613.234	87034.57432
J9R021	Eukaryoti	12	12	19	12	1382	166.4	6.79	1172000.816	1659614.5
Q12906	Interleuk	19	12	20	12	894	95.3	8.76	1852959.512	4047170.589
Q8IY37	Probable	14	13	17	13	1157	129.5	8.1	818561.207	3500479.182
P25705	ATP synth	17	5	8	5	553	59.7	9.13	1982644.178	1479297.696
A0A8V8TNX	Pyruvate	20	8	11	8	605	65.8	7.99	6665488.063	68195.46523
P16104	Histone H	46	4	9	3	143	15.1	10.74	7096080.637	
B4DS57	Nuclear a	15	4	5	4	688	74.8	4.27	1597286.508	
Q68DI0	AP comple	14	9	12	5	951	105.6	5.38	431054.0762	923537.9636
A0A7P0TAE	Endoplasm	18	7	9	6	789	90.7	5.24	4191437.031	13539.81218
A8K651	Complemen	35	6	7	6	282	31.4	4.84	10692349.22	168036.4713
P06744	Glucose-6	13	3	6	3	558	63.1	8.32	2182821.453	
Q15393	Splicing	15	6	7	6	1217	135.5	5.26	829336.375	99015.69109
A0A024RA6	Cell divi	16	14	19	13	1452	158.3	9.7	568204.041	3022053.05
B4DP52	HCG200563	25	5	7	4	350	40.6	8.73	4007334.938	
Q8TDD1	ATP-depen	20	13	18	13	881	98.5	10.02	1223871.305	3396856.064
A0A7P0T9F	Cytoplasm	4	6	7	6	3758	431.5	6.35	849101.5234	
Q07065	Cytoskele	30	14	18	14	602	66	5.92	733968.6523	3732334.573
G9K389	YWHAE/FAM	14	3	7	3	384	41.3	4.97	2650967.633	
P17987	T-complex	20	4	5	4	556	60.3	6.11	2491848.555	
O75533	Splicing	14	12	14	12	1304	145.7	7.09	960613.3525	1615721.173
Q6FHF5	Prolifera	41	4	6	4	261	28.7	4.69	1827040.563	
E9PQD7	40S ribos	31	7	11	7	235	25.2	10.24	3316904.875	3620777.211
P54886	Delta-1-p	15	5	6	5	795	87.2	7.12	1261506.148	
P20700	Lamin-B1	25	7	8	7	586	66.4	5.16	1084665.844	
V9GZ78	C-1-tetra	13	5	6	5	854	92.5	6.95	1298787.203	
A0A024R2Z	Guanine n	28	11	11	11	537	60.5	8.79	509882.6162	3673982.515
Q53FG5	DNA repli	16	6	7	6	734	82.2	8.28	605445.4609	562563.1385
A0A1U9X98	PRRC2A OS	11	13	18	13	2157	228.7	9.45	1758848.242	1398443.39
Q13823	Nucleolar	20	12	15	12	731	83.6	9.25	1497768.906	4065000.112
B4DFL1	Dihydroli	28	4	4	4	459	48.9	7.49	1593876.781	
Q02413	Desmoglei	18	12	19	12	1049	113.7	5.03	855266.6289	1269544.857
A0A2R8Y5Y	60S ribos	29	3	4	3	220	25	9.74	596016.1875	
O75643	U5 small	8	7	7	7	2136	244.4	6.06	403091.75	
Q4VCS5	Angiomoti	16	12	16	12	1084	118	7.64	4886694.777	1995506.931
A0A087X0X	Heterogen	22	13	19	13	730	77.5	8.78	2986846.115	3670110.911
Q9P258	Protein R	26	11	16	11	522	56	8.78	1105243.047	2342715.98
B2RNR6	Zinc fing	15	10	15	10	1074	116.9	9.04	599174.4316	2669608.209
B4DUQ1	Heterogen	38	9	10	9	439	48.5	5.92	1615720.41	496633.5351
Q13200	26S prote	8	3	5	3	908	100.1	5.2	605156.9102	
A0A024QZW	Nucleopor	12	12	14	12	1475	153.9	8.73	363992.4941	1810541.33
Q10567	AP-1 comp	11	6	8	2	949	104.5	5.06	816989.7188	
Q71DI3	Histone H	29	2	7	1	136	15.4	11.27	65585039.49	
D6RGY2	Calnexin	27	8	11	8	528	60.5	4.59	2391589.996	2316725.158
P14923	Junction	22	13	18	13	745	81.7	6.14	1722159.9	1611065.716
Q6FI03	G3BP prot	25	8	15	7	466	52.1	5.59	1600362.104	2105643.019
P17066	Heat shoc	15	7	15	1	643	71	6.14	882611.8906	
A0A804HL4	Leucine-r	11	13	15	13	1393	157.7	6.18	637244.9141	2568766.944
P08758	Annexin A	25	3	5	3	320	35.9	5.05	1027554.125	
O15451	Proline-,	16	11	15	8	1021	109.1	4.26	247453.9805	2486751.024
A6NLN1	Polypyrim	28	8	11	8	527	56.5	9.38	2373111.99	209447.7721
Q96F88	Processin	15	11	17	11	1024	114.6	9.2	1265015.338	4494494.125
P18669	Phosphogl	36	5	6	5	254	28.8	7.18	3271000.178	100998.3196
A0A7I2V64	Ras GTPas	30	7	13	1	355	40.2	4.97	65108.27344	46594.25191
Q6P0P7	THRAP3 pr	26	8	13	2	374	41.7	11.5	522414.0586	1533844.135
P00441	Superoxid	45	2	4	2	154	15.9	6.13	1929287.703	
E9PB61	THO compl	14	3	4	3	264	27.5	11.05	3444397.266	49048.377
P09211	Glutathio	45	4	5	1	210	23.3	5.64	776802.4063	253765.0171
P05388	60S acidi	36	7	8	7	317	34.3	5.97	1579853.25	1322210.084
P63244	Receptor	36	6	7	6	317	35.1	7.69	3048169.629	68286.48532
Q01780	Exosome c	19	13	15	13	885	100.8	8.46	774502.7168	3011649.519

F5H5D3	Tubulin a	30	13	1	519	57.7	5.07	97486746.72	188952694.9
Q13435	Splicing	28	21	21	895	100.2	5.67	82621749.57	126056011.1
B3KX19	116 kDa U	23	19	19	962	108.1	5	89974582.17	75484680.13
I6L9E8	Family wi	26	11	8	518	55.1	8.88	109745452.1	53573704.63
Q96N67	Dedicator	15	26	26	2140	242.4	6.8	33263929.17	67699215.69
Q02878	60S ribos	45	15	15	288	32.7	10.58	618302694.3	908777651.3
Q16195	Keratin (	55	12	1	244	27.6	4.83	1234183.25	2995802.25
Q8N6N5	Tubulin b	42	15	3	445	49.8	4.86	49461198.3	99283540.25
A0A024R15	HCG28765,	18	17	1	1337	147.1	5.01		626935.0625
O75533	Splicing	19	20	20	1304	145.7	7.09	62487565.92	76678490.63
A0A7I2V64	Ras GTPas	39	11	1	355	40.2	4.97		4136331.5
A0A384NYT	Tubulin b	42	15	3	442	49.4	4.89	13160846.46	23444366.88
H7BXY3	RNA helic	22	23	23	1166	130.5	8.56	37503720.85	81534837
A8K984	Structura	19	23	23	1197	135.5	8.43	59100067.49	156646924.3
P11021	Endoplasm	28	15	14	654	72.3	5.16	22369170.5	37434603.44
P39023	60S ribos	33	13	2	403	46.1	10.18	110964530.1	227176471
Q96F88	Processin	21	16	16	1024	114.6	9.2	26608550.52	79880998.13
B0QYK0	RNA-bindi	18	8	2	618	64.9	9.38	556495902.3	228214065.4
O15226	NF-kappa-	32	23	23	690	77.6	8.79	51036280.02	136454512
Q5HYL6	Uncharact	46	14	13	352	39.5	5.19	85338110.07	53190189.63
P46013	Prolifera	10	20	20	3256	358.5	9.45	7289256.195	42168879.56
Q58EY4	SWI/SNF r	14	15	8	1105	122.8	5.72	23236481.76	38550824.63
A0A5F9ZHW	Ataxin-2	18	16	15	1178	127.1	8.97	48986222.82	57657079.38
Q53HQ7	Elongatio	37	13	13	462	50.2	8.94	162365090.9	173038586.8
B7Z4V2	Stress-70	30	16	16	665	72.4	5.94	61647500.11	58775463.25
Q92621	Nuclear p	11	20	20	2012	227.8	6.19	17661789.45	44718944.19
Q92667	A-kinase	24	17	17	903	97.3	4.94	36553229.94	67102846.5
A4D1I0	HCG174180	14	18	17	1322	148.1	5.41	72690439.63	63640724.81
Q9BUP0	EF-hand d	46	9	7	239	26.9	5.39	22632459.31	64549989.5
B7Z7U2	cDNA FLJ5	24	19	2	938	103.5	5.35	3729676.517	7262196.25
P22626	Heterogen	42	14	12	353	37.4	8.95	338936867.1	202059295.1
Q13707	ACTA2 pro	28	7	2	330	36.8	5.35	65496910.31	117371847.1
Q9BVJ6	U3 small	28	19	19	771	87.9	7.87	52642033.81	67793991.88
B3KS36	cDNA FLJ3	35	12	1	354	40.1	10.3	2725757.539	7542867.5
Q9NTJ3	Structura	17	22	22	1288	147.1	6.79	27610878.38	80707940.88
M0QZM1	Heterogen	43	16	1	383	40	6.73	809017.1633	1922371.375
B2RNR6	Zinc fing	18	17	17	1074	116.9	9.04	30565100.39	89054742.88
Q9BZE4	GTP-bindi	30	19	19	634	73.9	9.5	32889842.13	94772209.75
Q8TDD1	ATP-depen	24	17	17	881	98.5	10.02	48019617.73	71983718.06
P49327	Fatty aci	10	20	19	2511	273.3	6.44	8651082.68	37071104.56
A0A1W2PQD	Heterogen	38	16	1	327	36.4	9.96	1693619.299	1004879.313
Q9GZR7	ATP-depen	20	15	15	859	96.3	9.06	22955514.29	59866735.69
P46087	Probable	27	18	18	812	89.2	9.23	25293071.79	96540972.31
A0A024R2K	Leucine r	19	13	12	742	84.1	6.98	26836114.08	37572873.44
Q0QEN7	ATP synth	41	13	13	445	48.1	5.07	38492122.54	52144358.31
A0A7G6J4N	Ubiquitin	22	13	13	802	87.5	5.27	36243378.78	55521243.63
A0A087X29	WD repeat	17	14	14	1151	124.9	6.92	8434901.207	35664782.25
P13639	Elongatio	23	19	19	858	95.3	6.83	72155434.61	58618015
A0A024RDF	Heterogen	40	13	11	306	32.8	8.16	344342476.1	196559607.1
O95373	Importin-	16	13	12	1038	119.4	4.82	35863082.93	55182144.56
P16615	Sarcoplas	16	14	14	1042	114.7	5.34	21110800.22	50307534.63
P18124	60S ribos	41	13	13	248	29.2	10.65	258030372.9	433312321.8
Q15058	Kinesin-l	13	18	18	1648	186.4	7.91	18011690.1	38853144.75
Q7Z6E9	E3 ubiqui	11	15	15	1792	201.4	9.64	16544935.31	39970030.69
P62241	40S ribos	51	10	10	208	24.2	10.32	117699433.2	277606985.4
Q92878	DNA repai	17	23	22	1312	153.8	6.89	49195080.34	72104149.81
Q96C19	EF-hand d	46	11	9	240	26.7	5.2	418158045.1	416356079.9
B4DZQ5	Serine/th	14	13	12	993	115.4	10.27	35086547.02	60859315.75
A0A7P0TAE	Calnexin	28	15	15	626	71.3	4.72	108940194.6	93047617.91
Q13823	Nucleolar	19	12	12	731	83.6	9.25	24324657.66	80960872.13
A0A1W6S96	GTF2I-BRA	16	13	1	955	107.6	7.94	580589.0173	1050441.875
A0A6Q8PFJ	Prelamin-	26	18	17	729	80.9	8.27	64017864.47	73921304.69
P61247	40S ribos	42	13	13	264	29.9	9.73	234359467.6	313189562.4
B7ZVZ4	KIAA1211	15	13	13	1226	136.4	5.88	33093203.56	40326770.88
O60841	Eukaryoti	16	13	13	1220	138.7	5.49	13120889.25	37303741
A8MXP9	Matrin-3	20	15	15	895	99.9	6.04	58797369.26	57011199.41
Q15393	Splicing	16	17	17	1217	135.5	5.26	23947107.52	69436600.81
A0A6Q8PGN	Inverted	14	13	13	1241	134.6	5.33	24580522	34405807.19
A0A2R8Y7Z	Catenin b	20	13	8	780	85.3	5.92	20760867.28	15691757.47
A8K6Q4	cDNA FLJ7	18	20	18	1123	128.5	6.32	62358051.21	38975207.88
P17987	T-complex	30	15	15	556	60.3	6.11	52890953.78	48836241.63
Q63HR1	Uncharact	34	11	11	387	42.4	8.44	87576432.69	99341038.75
A0A0G2JIW	Heat shoc	28	15	13	642	70.1	5.66	35678708.68	39355269.69
O14950	Myosin re	44	6	6	172	19.8	4.84	162822358.4	98446726.13
P62753	40S ribos	30	9	9	249	28.7	10.84	218358346.1	369792590.8
O75970	Multiple	10	17	16	2070	221.5	5.06	7765487.783	31225833.88
H0YEH2	Pumilio h	15	13	13	1125	120.2	6.8	33465527.66	65534190.75
Q15424	Scaffold	21	14	8	915	102.6	5.47	93196991.8	56297399.94
P05141	ADP/ATP t	42	14	4	298	32.8	9.69	489292546.7	710623242.1
Q9NW13	RNA-bindi	22	15	15	759	85.7	9.22	20945289.65	76177936.19
Q6KB66	Keratin,	38	15	15	452	50.5	5.67	45548626.81	18619947.31
Q9NZI8	Insulin-l	27	14	13	577	63.4	9.2	19138057.39	69710586.13
O76021	Ribosomal	26	14	14	490	54.9	10.13	63523265.58	74726065.56
A0A8I5KT5	Kinase D-	11	18	18	1764	195.9	6.79	17192240.51	40414006.13
E9PCY7	Heterogen	20	6	2	429	47.1	6.34	34074604.08	37873857.63
P08727	Keratin,	23	13	0	400	44.1	5.14	18005569.91	15501946.34
A0A7P0TAE	Endoplasm	24	17	16	789	90.7	5.24	45724600.61	34475870.44
O95833	Chloride	51	10	10	236	26.6	6.43	594972.3918	190388597.3
F5GZS6	4F2 cell-	25	13	13	599	64.8	5.1	115141235.3	441703561.5
Q9NVP1	ATP-depen	24	13	13	670	75.4	9.5	27917003.07	48791245.63
P16989	Y-box-bin	23	7	3	372	40.1	9.77	18734883.02	29652628
H7C463	MICOS com	31	15	11	613	68.1	6.06	38820960.5	51178684.31
P04406	Glycerald	31	7	7	335	36	8.46	81503450.03	47940941.94
A0A7I2V46	Exportin-	14	13	13	1056	121.5	5.97	22597560.96	31486061.25
P62906	60S ribos	43	10	10	217	24.8	9.94	99305809.54	188199780.6
F8W6I7	Heterogen	37	9	7	307	33.1	9.13	49621077.16	66742163.31
Q07666	KH domain	28	9	9	443	48.2	8.66	201852564.2	120843003
O15144	Actin-rel	41	13	13	300	34.3	7.36	67315324.54	66821823.44
Q9C0C2	182 kDa t	10	14	14	1729	181.7	4.86	4340278.221	32124529.13
P07355	Annexin A	40	15	15	339	38.6	7.75	76558634.39	45374876.13
J9R021	Eukaryoti	14	17	17	1382	166.4	6.79	51207395.73	125066253.9
A0A0A0MRJ	Neuron na	11	15	15	1830	197.3	8.22	6349921.257	21054936.06
P35251	Replicati	12	11	11	1148	128.2	9.36	10463651.37	28570749.19
Q14683	Structura	14	16	16	1233	143.1	7.64	16089566.41	56709418.81
Q14571	Inositol	5	14	8	2701	307.9	6.43	4046524.246	11751637.25
P12035	Keratin,	13	13	1	628	64.4	6.48	3398784.689	1174357.5
A0A2U3TZM	DNA helic	9	16	12	1902	216.7	6	1926538.562	36214636.44
Q92747	Actin-rel	37	12	12	370	41.5	8.18	68429344.94	69082853.5
Q8IWC1	MAP7 doma	21	17	17	876	98.4	9.32	21808468.15	37295734.75
A0A8B0RAT	Enhancer	12	15	15	1437	155.3	5.92	48606955.12	51133685.94
Q13576	Ras GTPas	12	17	15	1575	180.5	5.64	12384817.23	23949804.25
Q5C9Z4	Nucleolar	17	13	13	860	96.2	8.1	8548169.041	39873776.94
Q01780	Exosome c	19	13	13	885	100.8	8.46	18701505.76	58773317.31
A0A2R8Y5V	Tropomyos	41	14	6	248	28.6	4.7	20904256.91	37726292.44
Q8TB01	Similar t	27	13	13	560	62	5.68	21945954.24	33158896
Q99569	Plakophil	16	16	16	1192	131.8	8.94	17547762.7	26536061.69
Q14684	Ribosomal	18	10	10	758	84.4	9.76	8199988.819	24430894.31
B4DUQ1	Heterogen	36	12	12	439	48.5	5.92	29324616.09	33327198.13
K7ERE3	Keratin,	23	12	2	415	45.2	4.81	9520900.936	305248.3438
Q8TCJ2	Dolichyl-	14	11	11	826	93.6	8.91	658491310.6	408052656.5
H7BY36	RNA-bindi	25	7	1	308	32.2	9.82		
Q14692	Ribosome	11	13	13	1282	145.7	6.44	11527339.71	36270200.69
O00268	Transcrip	15	11	11	1085	110	9.94	27932017.57	25649392.13
Q14151	Scaffold	18	13	7	953	107.4	6.16	14809590.42	9387170.5
P51610	Host cell	9	12	12	2035	208.6	7.46	16253829.74	25702504.63
Q9C0J8	pre-mRNA	12	13	13	1336	145.8	9.17	28728080.08	40250994.38
O95793	Double-st	23	9	9	577	63.1	9.44	13545461.07	27767456.88
P52907	F-actin-c	49	10	8	286	32.9	5.69	44411927.02	45041060.81
B4DLG2	cDNA FLJ5	22	13	13	756	82.7	8.65	21612518.59	43234782.38
Q6P2Q9	Pre-mRNA-	8	17	17	2335	273.4	8.84	7469161.842	24981921.44
P78527	DNA-depen	4	17	17	4128	468.8	7.12	5958142.933	16519837.56
P23396	40S ribos	52	13	13	243	26.7	9.66	176039149.7	249859473.8
J3KS54	Protein f	21	13	1	700	78	6.32	947317.4948	758983.25
P25705	ATP synth	23	12	12	553	59.7	9.13	62289949.99	70601962.81
A0A024R1J	Neurofibr	23	15	15	595	69.6	6.47	43204146.18	59204774.13
Q68CP9	AT-rich i	9	13	13	1835	197.3	7.42	2528844.654	22709294.63
Q15323	Keratin,	36	14	2	416	47.2	4.88	79134565.14	17025722.75
Q6P4F7	Rho GTPas	14	12	12	1023	113.8	9.07	20497982.17	34945712.5
Q14203	Dynactin	11	13	13	1278	141.6	5.81	27600591	30177537.19
H7BXH2	Serine/th	15	11	11	827	92.4	4.63	14366871.66	40539780.63
A0A0S2Z5K	Spermatid	19	13	7	657	72.1	8.73	4129788.936	13435160
O95239	Chromosom	13	16	16	1232	139.8	6.27	18335795.6	20970743.13
P05388	60S acidi	34	8	8	317	34.3	5.97	30543007.45	71301484.13
C9JZR2	Catenin d	16	13	13	938	104.8	6.87	34410357.84	42304376.63
A6NLN1	Polypyrim	25	11	11	527	56.5	9.38	35681287.98	63229530.06
A0A6I8PS1	Guanine n	23	10	10	516	58	7.05	7047507.74	39789953.75
P06702	Protein S	45	4	4	114	13.2	6.13	125712585	38908848.63
O60237	Protein p	14	13	12	982	110.3	5.67	28584402.25	29830373.44
O76009	Keratin,	31	13	2	404	45.9	4.82	7454594.689	
P30050	60S ribos	59	7	7	165	17.8	9.42	188511788.5	130699070.4
B2R7T8	F-actin-c	36	10	10	272	30.6	6	79101902.15	80607245.69
Q12797	Aspartyl/	18	13	13	758	85.8	5.01	29322199.26	58191624.5
Q92615	La-relate	16	10	10	738	80.5	6.92	20665365.81	32215593
P22735	Protein-g	19	15	15	817	89.7	6.04	53602130.5	19249518.97
Q6P0P7	THRAP3 pr	28	8	1	374	41.7	11.5	24661702.02	7647718.5
B4DV79	Eukaryoti	18	11	11	738	85.1	5.26	17034146.26	13905291.5
Q08188	Protein-g	22	13	13	693	76.6	5.86	39446770.31	10974415.97
A0A0U1RRM	Protein e	17	9	9	802	87.3	7.77	28241819.44	33962264.38
Q86VP6	Cullin-as	13	14	14	1230	136.3	5.78	18354516.24	24080662.25
O76094	Signal re	19	11	11	671	74.6	9.26	46747592.98	49137116.56
O94887	FERM, ARH	10	8	6	1054	119.8	8.79	13247916.88	25687435.5
Q5VT25	Serine/th	8	13	8	1732	197.2	6.58	7915590.962	18775780.06
P61158	Actin-rel	30	10	10	418	47.3	5.88	35459613.24	33403873.06
P26358	DNA (cyto	9	13	13	1616	183.1	7.75	5093290.915	26177996.13
Q69YN4	Protein v	8	15	15	1812	201.9	5.01	18050200.31	28133229.5
P12236	ADP/ATP t	35	11	1	298	32.8	9.74	16084572.93	16030239.69
A0A8J9BZL	Golgin su	12	10	10	1029	116	5.05	20261225.75	27873267.69
P51532	Transcrip	7	10	10	1647	184.5	7.88	8091959.923	27108841.63
Q562R1	Beta-acti	16	5	1	376	42	5.59	417103842.5	434077728
B4DKS0	Monocarbo	11	7	7	480	51.8	8.5	24468274.06	72970636.39
P16403	Histone H	32	9	2	213	21.4	10.93	43122718.88	35012409.5
A8KA74	cDNA FLJ7	13	11	11	857	97.6	7.69	12639843.99	26873348.88
Q4VB24	Histone c	31	9	2	219	21.9	11.03	22821122.22	14198247.5
A0A7I2V59	60 kDa he	23	10	10	537	57.8	7.77	34822547.47	55843611.19

P55060	Exportin-	9	4	5	4	971	110.3	5.77	263510.4375	
B5BUB5	Autoantig	13	4	7	4	408	46.8	7.12	1741761.828	752924.4013
O95833	Chloride	31	5	9	5	236	26.6	6.43	8915.654297	2566060.847
Q16777	Histone H	43	3	8	2	129	14	10.9	9377561.723	
P23526	Adenosylh	15	3	5	3	432	47.7	6.34	1248219.586	
A0A2Z6ATB	Drebrin A	15	8	11	8	695	76.3	4.51	3290495.961	1426861.527
Q04695	Keratin,	31	13	18	7	432	48.1	5.02	3060433.371	2448586.883
Q92616	eIF-2-alp	5	5	6	5	2671	292.5	7.43	378424.957	
Q5T9A4	ATPase fa	17	10	20	4	648	72.5	9.2	1464538.133	4667188.927
P84243	Histone H	29	2	4	1	136	15.3	11.27	28585513	
Q2TAY7	WD40 repe	17	3	5	3	513	57.5	7.18	898678.0625	
H7BXY3	RNA helic	14	14	20	14	1166	130.5	8.56	1900527.629	2585494.564
A0A8B0RAT	Enhancer	10	12	15	12	1437	155.3	5.92	1973010.988	1258333.726
Q2NL82	Pre-rRNA-	17	9	13	9	804	91.8	7.42	793644.7754	2828909.997
O43598	2'-deoxyn	29	2	3	2	174	19.1	5.05	412428.7188	
A0A3B3IUA	Ribonuclo	13	2	4	2	272	30.1	9.55	895450.875	
M0QY97	Zinc fing	19	9	10	9	910	95.5	7.33	345463.2266	3129652.841
Q53HM6	Splicing	14	3	4	3	501	58.8	5.38	1168910.758	
E9PCY7	Heterogen	26	7	12	3	429	47.1	6.34	1866666.109	2024590.959
Q9Y4Y9	U6 snRNA-	36	1	4	1	91	9.9	4.54	192673.9531	
Q2L6I2	ABC50 pro	16	8	8	8	807	91.6	7.68	387290.3398	947708.4618
A0A7I2YQK	Inosine-5	19	4	4	4	513	55.5	7.02	638095.4609	
O15226	NF-kappa-	15	9	11	9	690	77.6	8.79	617513.6992	2012076.659
C7DJS2	Glutathio	57	4	5	1	151	16.7	5.1	40717.77344	
Q02790	Peptidyl-	12	3	4	3	459	51.8	5.43	2547151.938	456948.7421
B4DX78	RNA helic	13	3	5	2	470	53.7	5.9	295271.6797	
Q00341	Vigilin O	11	11	12	11	1268	141.4	6.87	781722.7266	1633221.048
Q6IQ30	Polyadeny	15	7	12	3	660	72.3	9.35	157210.8164	262098.9821
Q71UI9	Histone H	40	4	7	3	128	13.5	10.58	18214765.33	139361.4116
P54577	Tyrosine-	13	3	5	3	528	59.1	7.05	726133.5469	
Q53G71	Calreticu	16	2	3	2	406	46.9	4.45	1614554.484	
P51991	Heterogen	19	7	8	6	378	39.6	9.01	2256957.287	413325.7839
A0A8V8TM6	HCG181174	12	7	9	7	934	106.3	7.99	484577.0586	1222003.153
A0A286YFA	D-3-phosp	18	4	5	4	445	47.6	5.9	1466985.27	
Q8IY98	ACTR2 pro	16	2	4	2	339	38.7	6.09	164382.5469	
Q9NTJ3	Structura	13	11	12	11	1288	147.1	6.79	588954.5293	1321963.216
Q8TAQ2	SWI/SNF c	7	6	9	2	1214	132.8	5.69	15405.84473	142295.5266
P10599	Thioredox	58	4	7	4	105	11.7	4.92	12937135.41	399737.7708
P62805	Histone H	50	5	30	5	103	11.4	11.36	14089828.95	846179.1299
A8K9A4	cDNA FLJ7	19	3	4	1	306	33.6	5.14	420868.3125	
Q86UE4	Protein L	17	8	12	7	582	63.8	9.32	1766136.362	3425233.916
O43390	Heterogen	11	6	10	3	633	70.9	8.13	979090.1953	203018.4467
B4DKU3	cDNA FLJ5	19	10	10	10	726	82.5	6.38	420306.8633	1782645.655
Q86VP6	Cullin-as	5	3	5	3	1230	136.3	5.78	769313.0391	
B0QYK0	RNA-bindi	9	3	10	3	618	64.9	9.38	756040.1621	2771014.479
B2RBS8	cDNA, FLJ	14	9	18	9	609	69.3	6.28	4071199.914	7071093.482
Q16836	Hydroxyac	30	3	3	3	314	34.3	8.85	1041589.938	
Q01105	Protein S	19	3	4	3	290	33.5	4.32	2546016.391	237239.1766
Q96BS4	FBL prote	33	4	7	4	260	28.4	9.42	1830007.73	125189.083
Q9Y617	Phosphose	22	3	4	3	370	40.4	7.66	1832630.297	
D6RBZ0	Heterogen	14	4	6	3	327	35.7	6.95	1702006.313	164686.023
B4DN87	Serpin H1	15	3	3	3	397	44.2	8.95	478856.375	
P60842	Eukaryoti	26	5	7	5	406	46.1	5.48	4334007.648	102829.0146
P63104	14-3-3 pr	25	4	5	4	245	27.7	4.79	1244806.695	
Q9NR45	Sialic ac	16	2	3	2	359	40.3	6.74	704575.5625	
P51532	Transcrip	6	9	9	9	1647	184.5	7.88	310751.3086	1820444.893
A4QPB0	IQ motif	8	8	9	8	1657	189.2	6.48	219322.8125	502800.0883
Q6PKG0	La-relate	11	12	15	12	1096	123.4	8.82	1444259.713	3527509.036
P62424	60S ribos	26	7	10	7	266	30	10.61	1831422.789	4870215.666
E5RI56	Skp1_POZ	32	3	4	3	197	22.7	4.55	711925.2813	
B4DM97	cDNA FLJ5	9	2	3	2	334	38.3	6.96		
A0A7I2YQN	Heterogen	19	8	11	6	561	62.5	7.56	1555920.563	2053826.553
Q7Z406	Myosin-14	4	7	7	1	1995	227.7	5.6	44105.60547	
A8K4W0	40S ribos	28	7	10	7	264	29.9	9.73	2760883.922	5068356.359
P10768	S-formylg	31	4	5	4	282	31.4	7.02	1012290.535	
Q96IR1	RPS4X pro	36	7	12	7	243	27.2	9.94	700131.2012	1644438.048
F8W651	Coatomer	28	1	2	1	119	13.4	4.82		
P39687	Acidic le	14	2	3	2	249	28.6	4.09	6587895.25	
B3KMX0	DNA repli	5	2	3	2	863	96.5	6.74	1397797.25	
Q9BPX3	Condensin	8	4	4	4	1015	114.3	5.59	53150.18359	
Q9BXP5	Serrate R	10	7	9	7	876	100.6	5.96	1056029.123	464438.0125
P61313	60S ribos	39	7	12	7	204	24.1	11.62	3934393.609	10041157.94
Q9Y3I0	RNA-splic	21	7	9	7	505	55.2	7.23	722158.0547	659852.886
A0A024RCU	SFRS prot	21	8	9	7	548	61.9	5.69	1181669.875	3618376.532
Q92621	Nuclear p	4	3	3	3	2012	227.8	6.19	873985.457	
O95625	Zinc fing	11	9	10	9	1053	119.3	8.66	188514.1406	2237603.495
Q02878	60S ribos	30	8	10	8	288	32.7	10.58	3129797.963	12487364.81
B4DYY5	cDNA FLJ5	9	7	11	7	1078	113.5	6.68	434457.7031	2318800.672
B4DP06	Bifunctio	11	3	3	3	533	58.6	6.84	562931.7188	
A8K690	Stress-in	10	3	6	3	543	62.6	6.8	1111154.18	
P31946	14-3-3 pr	25	3	3	3	246	28.1	4.83	864116.9688	
A0A7I2V46	Exportin-	8	4	4	4	1056	121.5	5.97	492192.8008	95850.48074
A0A8I5QKQ	Protein d	6	2	3	2	456	51	5.94	371464.1484	
Q13151	Heterogen	26	5	6	4	305	30.8	9.29	1399906.359	557276.8258
Q53FS6	TNF recep	13	4	5	3	704	80	8.03	2895134.977	
U3KPZ7	RNA-bindi	8	7	10	6	1027	114.5	9.45	632103.7754	1398557.087
E9PPJ0	Splicing	14	9	10	9	878	98.1	5.62	842842.9883	3044391.761
P11387	DNA topoi	15	10	11	10	765	90.7	9.31	996635.8477	2325259.378
P46087	Probable	7	3	4	3	812	89.2	9.23	340803.2344	297037.4069
B3KU93	Aminopept	12	3	3	3	756	85.2	5.26	368180.4453	17432.9324
P04075	Fructose-	16	3	4	3	364	39.4	8.09	3573062.563	
A0A087X09	Farnesyl	44	3	3	3	248	28.6	5.77	458062.0313	
P18124	60S ribos	31	9	10	9	248	29.2	10.65	2121288.836	7244563.163
P55795	Heterogen	18	5	8	2	449	49.2	6.3	77683.96484	10685.74736
P49773	Adenosine	20	2	3	2	126	13.8	6.95	1833063.453	3060383.882
Q04760	Lactoylgl	20	2	3	2	184	20.8	5.31	816147.0313	
A0A7I2V4A	26S prote	18	2	2	2	358	40.4	4.94	577490.4375	
A8K6Q4	cDNA FLJ7	10	8	10	8	1123	128.5	6.32	534161.0352	1198733.586
Q9C063	LYST-inte	11	1	2	1	223	25.9	8.9	390731.375	
E9PQH6	Rho-relat	21	2	3	2	169	19.2	5.1	750241.3203	
Q71RC2	La-relate	15	8	10	8	724	80.5	6.61	781934.7969	2713479.445
P62241	40S ribos	39	7	10	7	208	24.2	10.32	1193939.582	2855102.693
P62306	Small nuc	41	1	2	1	86	9.7	4.67	738059.0625	
A0A3R5W30	UBA2/WTIP	11	3	3	3	788	87.4	6.46	2916492.25	
P00492	Hypoxanth	24	3	4	3	218	24.6	6.68	1486268.547	45441.10269
Q9NW13	RNA-bindi	12	8	8	8	759	85.7	9.22	2300483.496	1858375.47
P84077	ADP-ribos	46	4	5	2	181	20.7	6.8	1435428.281	215010.8522
A0A2U3TZM	DNA helic	4	3	3	3	1902	216.7	6	65098.83594	
Q96AG4	Leucine-r	25	5	6	5	307	34.9	9.57	500790.6602	1398924.281
H3BRG4	Cytochrom	14	2	2	2	412	44.6	9	143048.8594	
B4DRA0	cDNA FLJ5	16	5	8	5	502	56.4	10.02	421163.918	697449.6616
A0A6I8PL4	leucine--	6	3	3	3	1130	129.1	7.87	788962.6719	
Q9Y5B9	FACT comp	8	4	4	4	1047	119.8	5.66	474639.7148	10014.55719
B3KMB1	Structura	7	8	9	8	1197	135.5	8.43	1467935.73	2308679.231
Q4W4Y1	Dopamine	6	2	3	2	868	96	6.52	238696.3438	
P21796	Voltage-d	14	2	3	2	283	30.8	8.54	658521.5078	
A8CG34	Nuclear e	9	6	6	6	1229	125	10.37	89501.52344	1226833.675
Q9NVP1	ATP-depen	10	7	9	7	670	75.4	9.5	592113.9707	1473186.047
P52565	Rho GDP-d	29	2	3	2	204	23.2	5.11	977811.0625	
P30041	Peroxired	28	4	5	4	224	25	6.38	1351878.602	
Q9P035	Very-long	23	7	8	7	362	43.1	8.94	613209.4063	1483179.154
F5H018	GTP-bindi	16	2	4	2	198	22.4	8.73	1236191.125	33139.02518
A8K5W7	isoleucin	8	5	5	5	940	105.9	6.42	166734.293	407136.9355
Q9Y2X3	Nucleolar	6	1	2	1	529	59.5	8.92	218717.2031	
Q5VVC8	60S ribos	30	4	6	4	167	19	9.8	990406.5508	54388.39781
F5GY37	Prohibiti	7	2	3	2	267	29.7	9.88	2299719.25	
E9PK25	Cofilin-1	27	4	5	4	204	22.7	8.34	591806.4453	
H7C4C8	T-complex	14	2	3	2	323	35.5	5.96	1465770.063	
F4ZW63	NF45 OS=H	19	4	5	4	390	43	5.34	1084098.789	136500.4576
P53621	Coatomer	8	9	10	9	1224	138.3	7.66	681839.083	1363457.904
P62841	40S ribos	34	2	3	2	145	17	10.39	688002.4063	
O43290	U4/U6.U5	11	6	7	6	800	90.2	6.13	65032.61621	1120139.118
Q14692	Ribosome	6	7	8	7	1282	145.7	6.44	411762.5889	1354969.241
B4DY08	Heterogen	16	3	3	1	288	32	8.72	476561.5078	21786.85876
Q96P70	Importin-	5	2	3	2	1041	115.9	4.81	1233746.961	
H3BQZ9	Adenine p	27	2	3	2	153	16.6	5.59	858096.4844	
Q16778	Histone H	33	4	8	4	126	13.9	10.32	5016944.992	1190005.131
O75306	NADH dehy	10	2	2	2	463	52.5	7.55	696205.3438	
A0A6Q8PHH	glycine--	10	3	3	3	705	79.3	6.7	954215.1055	
B7Z2Z1	cDNA FLJ5	16	7	10	3	811	91	7.24	1190953.646	1441966.352
A0A024QZN	Vinculin	2	1	2	1	1066	116.6	6.09	218524.9219	
Q5JTH9	RRP12-lik	7	6	8	6	1297	143.6	8.75	206453.0449	1561071.753
P09936	Ubiquitin	27	2	2	2	223	24.8	5.48	367396.7813	
Q9H773	dCTP pyro	24	2	2	2	170	18.7	5.03	1036261.348	
P48556	26S prote	12	2	2	2	350	39.6	9.7	469733	
P32929	Cystathio	5	1	2	1	405	44.5	6.7	727737.5938	
A2A3R5	40S ribos	25	4	6	4	218	25	11.14	1043545.938	1074493.79
A0A494C12	Nucleolar	16	3	3	3	478	53	8.48	846891.25	
P05141	ADP/ATP t	23	6	10	3	298	32.8	9.69	4458663.172	10373769.1
A0A7P0T8N	Transcrip	8	7	8	7	1115	125.9	8.92	195867.0605	1310091.993
A0A0C4DGI	Citrate s	11	2	3	2	400	44.7	7.18	482800.3242	
P23381	Tryptopha	6	2	2	2	471	53.1	6.23	107083.7578	
Q6P2Q9	Pre-mRNA-	2	2	2	2	2335	273.4	8.84	771144.8906	
Q63HR1	Uncharact	8	2	3	2	387	42.4	8.44	924134.7188	
H0Y8G5	Heterogen	28	6	8	5	260	29.6	9.16	1511752.5	1590857.276
P84090	Enhancer	30	1	4	1	104	12.3	5.92	196354.5625	
A0A2R8Y6J	60S ribos	19	2	4	2	238	27	9.13	1017304.516	79753.79319
E9PKZ0	60S ribos	23	3	4	3	205	22.4	10.76	1660483.387	2161689.37
B4DKS0	Monocarbo	12	4	5	4	480	51.8	8.5	2638791.25	3034796.073
P35251	Replicati	8	6	6	6	1148	128.2	9.36	178807.459	779666.7812
Q6NVW9	SNRPA1 pr	34	2	2	2	175	20.1	8.65	60023.9375	
B5BUB1	RuvB-like	11	2	3	2	456	50.2	6.42	664962.5	
B4DUI5	Triosepho	27	4	4	4	213	22.9	6.92	992425.7109	50275.69034
J3KQ18	D-dopachr	29	1	2	1	132	14.2	7.3	399981.4063	
H0YI18	Protein c	25	1	2	1	80	9.1	4.74	1269738.625	
P50991	T-complex	11	3	3	3	539	57.9	7.83	285710.0547	
O15042	U2 snRNP-	8	5	7	5	1029	118.2	8.47	270343.168	591237.9237
A8K7N0	60S ribos	23	4	7	4	218	23.6	10.84	1301331.723	4587325.821
A0A5F9ZHJ	Acetyl-Co	15	2	2	2	337	35.5	7.37	1060086.125	
B4DLN1	cDNA FLJ6	6	2	2	2	442	48.1	9.52		
Q53FV0	Prohibiti	17	2	3	2	272	29.8	6.39	1413810.156	
I3L1P4	Proline-,	55	4	5	1	148	15.1	10.68		609845.219
B3KX19	116 kDa U	9	7	8	7	962	108.1	5	288336.1094	741923.6524
Q9BZE4	GTP-bindi	13	8	9	8	634	73.9	9.5	730792.0078	1248898.762

Q71RC2	La-relate	17	9	9	724	80.5	6.61	20821618.95	28615757.38
Q71DI3	Histone H	29	2	1	136	15.4	11.27		
P46781	40S ribos	48	13	13	194	22.6	10.65	541724695.1	670049064
A0A2R8Y59	Transcrip	18	11	11	638	73	8.28	8540576.268	27961487.56
Q5T8P6	RNA-bindi	14	13	12	1007	113.5	9.16	22431739.03	43750362.13
Q8TAQ2	SWI/SNF c	7	9	2	1214	132.8	5.69	1327406.159	2778119.375
A0A140TA6	Keratin,	28	12	3	436	49.4	5.06	8982536.716	367414.9688
P20700	Lamin-B1	23	14	11	586	66.4	5.16	26745590.87	36654801.75
P63244	Receptor	33	10	10	317	35.1	7.69	19636146.67	18924758.88
P78362	SRSF prot	19	10	8	688	77.5	4.97	29155363.2	47864968
F8W7C6	60S ribos	48	7	7	163	18.6	9.95	65203266.87	116060964.1
B4DYY5	cDNA FLJ5	12	9	9	1078	113.5	6.68	27586820.08	44415130.63
P61313	60S ribos	46	9	9	204	24.1	11.62	292186892	600493747.4
B2RWN5	HEAT repe	7	14	14	2144	242.1	6.55	7479372.726	28412895.38
Q2XSC7	Cellular	26	8	5	393	43.6	6.58	78788280.6	58881045.5
P46060	Ran GTPas	20	10	10	587	63.5	4.68	20952393.04	25968967.88
A8K5W7	isoleucin	11	9	9	940	105.9	6.42	11765419.52	13679950.69
Q14690	Protein R	7	12	12	1871	208.6	8.87	906631.389	15776146.63
P54652	Heat shoc	16	9	1	639	70	5.74	469740.5512	253639.9531
H0Y5B0	Band 4.1-	24	7	1	380	42.1	5.31	2148226.819	1331468.625
A0A804HL5	isoleucin	11	12	12	1237	141.5	6.2	16371977.95	27395311.81
B4DN72	cDNA FLJ5	25	13	6	486	53.5	5.66	66616100.1	4372232.781
Q8NI27	THO compl	8	13	13	1593	182.7	8.44	19060145.25	27122185.56
G3XAC6	RNA-bindi	22	8	8	423	48	10.56	38186063.8	65162402
O15042	U2 snRNP-	15	15	15	1029	118.2	8.47	70744892.64	72988994.88
A8K964	Pinin OS=	16	10	10	717	81.5	7.37	72821222.77	49970903.25
P62280	40S ribos	51	9	9	158	18.4	10.3	285652601.3	351492487.8
O15143	Actin-rel	30	10	10	372	40.9	8.35	53879106.36	53114422.88
P55795	Heterogen	14	4	1	449	49.2	6.3	882746.2588	
H7BYY1	Tropomyos	42	12	2	248	28.7	4.82	18764626.81	7085440
Q0ZGT2	Nexilin O	18	13	13	675	80.6	5.33	119906199.3	64198182.13
P11388	DNA topoi	8	11	11	1531	174.3	8.72	7355619.495	13195851.19
D9YZV5	Tropomyos	37	12	1	284	32.9	4.78	60690827.55	31803538.13
Q15459	Splicing	17	10	10	793	88.8	5.22	18706240.36	14780142.63
Q8IZL8	Proline-,	10	9	9	1130	119.6	4.34	6936886.887	21055951.44
P27816	Microtubu	16	14	14	1152	120.9	5.43	19816537.44	38524208.5
Q9NYV4	Cyclin-de	8	10	9	1490	164.1	9.44	12231571.38	25860443.13
P16402	Histone H	24	9	2	221	22.3	11.02	1555229878	1345535037
A0A7P0T8N	Transcrip	13	14	14	1115	125.9	8.92	15066777.73	32046460.88
P15880	40S ribos	36	10	6	293	31.3	10.24	191363821.9	262506549.5
O14578	Citron Rh	7	14	14	2027	231.3	6.57	6948711.825	47526764.75
Q9UK61	Protein T	7	13	13	1670	188.9	5.8	17479533.98	17421346.31
A0A3B3IRQ	Double-st	12	12	12	1158	128.3	8.62	9925827.024	20935694.63
Q9UNF1	Melanoma-	22	10	9	606	64.9	9.32	15892235.65	53576116.13
P54886	Delta-1-p	16	11	11	795	87.2	7.12	14738845.69	20758793.31
Q10570	Cleavage	9	12	12	1443	160.8	6.4	15484034.28	15365004.31
Q14C86	GTPase-ac	9	10	10	1478	164.9	5.22	8887056.987	19819211.88
B4DL07	cDNA FLJ5	16	10	10	683	78.4	9.11	9903665.497	33553530.63
P52597	Heterogen	25	7	5	415	45.6	5.58	30019152.61	32240275.31
P51991	Heterogen	21	7	6	378	39.6	9.01	45681940.19	46645456.75
A0A6Q8PHK	E3 ubiqui	6	9	9	2067	228.3	8.6		13748539.97
Q59F12	Protein 4	14	9	9	827	92.1	5.55	18015512.36	27085217.13
A0A8V8TMN	Coiled-co	20	7	7	608	67.2	7.81	7560983.423	15436864.75
P35658	Nuclear p	6	9	9	2090	213.5	7.47	4458060.147	18997803
D3YTB1	60S ribos	45	7	7	133	15.6	11.44	138141964.5	153232863.3
A8K6U2	cDNA FLJ7	13	9	9	941	105.1	8.87	1675541.521	12500612.13
A8KA33	cDNA FLJ7	20	11	11	696	77.1	5.83	31043322.97	32971862.25
B9EIS5	PCM1 prot	6	11	11	2016	227.5	5.03	6483380.045	16371269.94
A0A024R3R	Delta(14)	16	10	10	615	70.7	9.36	88855409.44	249399344.3
Q5JSZ5	Protein P	5	8	7	2229	242.8	8.34	3383359.992	10138697.63
A0A499FJY	RNA-bindi	13	10	10	925	101.3	9.96	23291796.81	26750181.25
B4DY08	Heterogen	20	9	9	288	32	8.72	37259334.29	38798311.13
Q9P206	Uncharact	16	11	11	1035	107	9.73	14202815.58	25934897.88
A0A6Q8PG3	Kinesin-l	17	13	13	717	81	6.1	25723801.01	49504581.88
Q4W4Y1	Dopamine	14	11	11	868	96	6.52	19870151.21	13287881.19
B7Z4B2	Aminopept	14	10	10	825	92.8	5.4	13688163.84	11907648.38
Q13151	Heterogen	31	7	6	305	30.8	9.29	35893946.91	27173605.25
Q9UL54	Serine/th	11	14	14	1235	138.2	7.27	3944299.623	23029146.25
O75694	Nuclear p	8	9	9	1391	155.1	6.16	7909461.653	14215520.41
A0A3B3ITJ	Heterogen	20	8	8	537	59.2	7.11	20036832.79	24955200.25
P49756	RNA-bindi	13	9	9	843	100.1	6.32	26913413.51	25122889.38
B3KXL8	cDNA FLJ4	21	8	8	529	57.4	4.34	15210998.78	27974338.25
O60264	SWI/SNF-r	11	11	11	1052	121.8	8.09	9053805.04	25505475.5
P12235	ADP/ATP t	31	10	1	298	33	9.76	477905.7853	1087101.75
Q9Y2X9	Zinc fing	11	8	8	895	96.9	8.48	5292675.564	22852315.38
Q6ZMY0	cDNA FLJ1	9	11	11	1340	150.9	8.22	4387302.42	14082120.25
A8K897	Nuclear p	15	12	12	819	93.3	5.77	23125072.22	19417891.19
Q9Y2X3	Nucleolar	17	7	7	529	59.5	8.92	8450682.046	17057040.06
O95625	Zinc fing	11	11	11	1053	119.3	8.66	7084384.79	39597072.38
Q68DI0	AP comple	13	12	5	951	105.6	5.38	14760808.9	18487798.75
A8CG34	Nuclear e	9	6	6	1229	125	10.37	7958305.292	25195057.13
P27708	CAD prote	5	9	9	2225	242.8	6.46	7983947.094	12421418.47
P07196	Neurofila	18	11	9	543	61.5	4.65	22380140.11	25098972.66
Q2NL82	Pre-rRNA-	12	9	9	804	91.8	7.42	12543574.66	37388305.97
Q5T750	Skin-spec	38	8	8	250	26.2	7.97	162804189.8	59299437.81
Q14974	Importin	12	10	10	876	97.1	4.78	32065564.07	31314431.25
Q9BQ39	ATP-depen	15	10	8	737	82.5	9.17	11065606.07	22423369.75
Q03701	CCAAT/enh	11	10	10	1054	120.9	5.94	6870010.361	18926622.25
B4DM74	60S ribos	40	7	7	154	18.1	10.48	254330023	330042746
M0QY97	Zinc fing	14	7	7	910	95.5	7.33	7047429.161	16641687.53
Q5T3I0	G patch d	23	9	9	446	50.4	9.63	10126729.74	21135188
A0A384P5Q	Catalase	19	9	9	527	59.7	7.39	34274059.67	10233375.75
V9HW25	Epididymi	31	10	3	284	33	4.67	1481196.433	4283652.25
Q9UIG0	Tyrosine-	7	10	10	1483	170.8	8.48	5286423.782	18283581.63
Q13835	Plakophil	16	10	10	747	82.8	9.13	44424501.65	15046861.56
O15397	Importin-	9	8	7	1037	119.9	5.16	3332971.35	11616205.69
Q5SSJ5	Heterochr	18	9	9	553	61.2	9.67	46188232.63	77594733.25
P40429	60S ribos	40	10	10	203	23.6	10.93	300415106	433870779.3
A0A7I2YQG	Ribosomal	25	6	6	216	25.7	11.27	134444582.2	241348705.8
A0A8Q3WKK	AP-3 comp	11	10	10	1049	116.5	6.3	9125406.525	18698803.75
A0A090N7Y	ATP-bindi	19	11	11	634	72.4	7.49	3074049.394	16520631.5
P05089	Arginase-	28	8	8	322	34.7	7.21	48934052.32	11264254.44
D3DPS3	Arginine/	12	9	9	840	92.6	10.11	12255492.2	30402917.5
B3KM97	very-long	24	8	8	308	36	9.5	38772199.31	90676892.38
F5GY05	Constitut	10	8	8	933	106	5.87	15285057.41	9642927.844
Q9ULW0	Targeting	16	10	10	747	85.6	9.23	16240940.74	16974933
B4E0S6	RNA helic	15	11	11	784	89.5	7.46	23538867.74	29821010.88
H0YFD6	enoyl-CoA	12	7	7	792	86.3	9.1	4057066.841	11585465.63
A0A2R8YFE	Actin, cy	51	3	1	79	8.8	9.2	5222154.314	5965694.5
Q9P2N5	RNA-bindi	10	7	6	1060	118.6	9.19	6405017.781	10282647.13
P49411	Elongatio	29	12	12	455	49.8	7.61	23345650.33	35133220.41
P12273	Prolactin	55	6	6	146	16.6	8.05	74841065.07	31827085.25
Q9HAU5	Regulator	9	10	10	1272	147.7	5.69	15085881.05	12019691.13
A0A068F7M	FH1/FH2 d	9	8	8	1190	129.2	6.37	6085177.59	12429864.56
A0A2R8Y84	40S ribos	30	4	4	131	15.2	10.9	21175057.38	15751027.75
Q9HBO0	Desmocoll	12	8	8	840	93.8	5.53	60403711.63	21419678.56
A4ZU86	Nucleopho	34	8	8	274	30	4.75	142229321.4	293814836
Q96QZ7	Membrane-	7	8	8	1491	164.5	7.58	7033988.235	9208595.25
P55060	Exportin-	9	9	9	971	110.3	5.77	13735952.59	14153511.56
A8K3C5	cDNA FLJ7	19	8	8	432	51.4	9.79	41071340.95	43971523.88
D3DR32	M-phase p	6	9	9	1780	206	5.8	3666815.823	11964023.75
Q02978	Mitochond	28	8	8	314	34	9.91	10633766.03	19959716.5
P05783	Keratin,	23	11	9	430	48	5.45	59165539.82	54627053.38
Q4KMP7	TBC1 doma	13	7	7	808	87.1	9.19	12812121.62	18798924.41
A8K8B2	cDNA FLJ7	15	9	7	655	74.2	6.1	22118543.23	45745928.5
P42167	Lamina-as	20	6	6	454	50.6	9.38	11395328.88	19680602.38
H3BQZ7	HCG204479	14	11	11	746	84.6	4.93	26366205.5	20785273.44
P31151	Protein S	49	7	7	101	11.5	6.77	23487402.57	6600758.727
P25311	Zinc-alph	27	8	8	298	34.2	6.05	42659176.56	15817909.31
B4E386	cDNA FLJ5	22	6	1	300	34.1	4.84	267323.5155	526370.625
Q8IWR0	Zinc fing	10	8	8	971	110.5	7.3	6530948.271	17452342.25
Q96BS4	FBL prote	31	6	6	260	28.4	9.42	13141704.94	17073293
Q86VX4	Structura	8	8	8	1217	141.4	7.18	5167247.925	10613876.88
Q9Y383	Putative	21	8	8	392	46.5	10.01	25094574.89	22594195
Q96QE3	ATPase fa	5	8	8	1844	207.4	9.19	1332521.009	12408681.63
Q96AG4	Leucine-r	25	7	7	307	34.9	9.57	21574135.62	42156377.13
P38159	RNA-bindi	21	8	8	391	42.3	10.05	42133386.9	24099222.94
A0A0A0MRM	Nucleolar	13	9	9	708	74.6	9.47	70180785.9	68852289.25
B4DKU3	cDNA FLJ5	16	9	9	726	82.5	6.38	11383657.82	23758835.38
P26373	60S ribos	28	7	7	211	24.2	11.65	363310827.6	529280786.1
K7EMS3	Keratin,	22	6	1	181	20.2	4.6	3914251.21	1391771.875
P83731	60S ribos	36	7	7	157	17.8	11.25	214577227.7	292561441
P22492	Histone H	25	6	3	207	22	11.71	9185740.537	10499610.63
P62917	60S ribos	26	5	5	257	28	11.03	171356527	309032203.8
B3KQ33	cDNA FLJ3	8	8	8	1081	118.7	4.96	11304904.99	16594744.19
P46977	Dolichyl-	12	9	9	705	80.5	8.07	22866035.31	56969519.75
B3KN06	Coronin C	19	8	8	474	53.2	6.93	17183760.87	17459478.38
A0A1U9X8D	PPP1R18 C	15	7	7	613	67.9	5.4	38097470.24	34416363.25
K7EMD9	Keratin,	38	8	1	165	19.2	4.74	1862751.266	
Q52LJ0	Protein F	17	6	3	433	45.5	8.69	4899503.007	2587172.375
Q9NQW6	Anillin C	8	9	9	1124	124.1	8.07	11872076.57	19912625.88
P62277	40S ribos	36	6	6	151	17.2	10.54	142727624.8	114796385.6
A8K651	Complemen	17	4	4	282	31.4	4.84	7319928.136	13070785.75
A8K8N3	cDNA FLJ7	8	7	7	1193	134.3	5	7181005.696	12775488.75
J3KRX5	60S ribos	39	7	7	174	20.2	10.1	114741967.5	150682739.2
Q96P63	Serpin B1	17	6	6	405	46.2	5.53	75544155.46	39583840.75
A0A0S2Z53	E3 ubiqui	9	9	9	1001	113.6	6.23	16719579.83	17661270.06
G3V203	60S ribos	34	5	5	164	18.7	11.59	379337330.1	664234787.6
P78386	Keratin,	18	11	4	507	55.8	6.55	11533825.44	
P05386	60S acidi	52	2	2	114	11.5	4.32	7187920.099	2401817.5
Q9BUQ8	Probable	12	8	8	820	95.5	9.55	9098465.724	18859631.94
B7Z592	Zinc fing	20	7	7	392	44.9	7.87	2165897.297	17389691.5
B2RAN0	DNA helic	6	8	8	1417	158.9	7.56	1395341.658	8556673
Q5VZL5	Zinc fing	6	8	7	1548	172.7	6.84	6263251.218	13540418.63
B3KPD3	cDNA FLJ3	12	8	8	752	86.7	9.52	6712479.192	16129063.06
A0A2R8Y6J	60S ribos	34	7	7	238	27	9.13	37134778.39	38517812
B4DTA2	cDNA FLJ6	25	6	4	271	30.2	8.72	24585318.7	16168625
Q9NQX4	Unconvent	5	10	7	1742	202.7	7.71	4404003.631	6857967.563
A0A8V8TQW	DNA misma	10	8	8	951	108.1	7.44	1644414.821	9061613.375
Q8WUM0	Nuclear p	8	8	8	1156	128.9	5.1	7566719.172	13733562.13
P46778	60S ribos	38	6	6	160	18.6	10.49	76424843.2	129035285.8
Q59E91	DNA-direc	8	8	8	1072	121.2	8.69	7695863.139	19398055.44
A8K7N0	60S ribos	28	5	5	218	23.6	10.84	116864780.4	174037387.8
Q5JR04	RNA helic	9	8	8	947	107.1	8.79	7997646.104	11986050.88

Q5U043	S-(hydrox	18	2	2	2	374	39.7	7.49	433095.1875	405662.7811
B4DF70	cDNA FLJ6	23	3	3	3	183	20.1	8.78	118125.7969	26913.87847
P45974	Ubiquitin	9	3	3	3	858	95.7	5.03	608295.2734	
O76021	Ribosomal	13	6	9	6	490	54.9	10.13	1482295.914	2737410.58
O00231	26S prote	12	2	3	2	422	47.4	6.48	100607.5547	
O75131	Copine-3	8	2	2	2	537	60.1	5.85	58885.23828	
Q5C9Z4	Nucleolar	11	7	7	7	860	96.2	8.1	179831.3027	848031.0306
A0A0A0MSK	Torsin-1A	5	1	2	1	462	52.4	7.06	216295.7813	
Q99436	Proteasom	14	2	2	2	277	29.9	7.68	214588.9844	
Q4VB24	Histone c	19	5	9	5	219	21.9	11.03	32056400.4	17293259.76
F5GZQ3	Trifuncti	9	2	3	2	459	49.6	9.42	538369.5781	
H0Y8C6	Importin-	4	2	3	2	1099	123.8	5.06	171913.9453	
P78362	SRSF prot	11	6	7	5	688	77.5	4.97	368672.1787	1069398.092
Q9BZZ5	Apoptosis	15	4	4	4	524	59	7.34	464291.3242	65056.33826
A0A087X26	TAR DNA-b	15	3	3	3	301	34.2	6.44	813890.6016	
B4E0S6	RNA helic	13	7	8	7	784	89.5	7.46	390879.9492	616304.651
B4DZC3	5'-3' exo	8	5	6	5	896	102.4	7.64	274836.8457	475479.2868
B2RE88	Phosphate	19	4	5	4	361	39.9	9.36	905168.5078	622062.0997
Q96ST3	Paired am	8	7	7	7	1273	145.1	7.25	132396.0146	1269820.807
I6L9E8	Family wi	13	4	6	4	518	55.1	8.88	324211.2715	316077.7373
A8KA74	cDNA FLJ7	8	5	5	5	857	97.6	7.69	358088.6699	480374.4013
P49756	RNA-bindi	7	3	4	3	843	100.1	6.32	161606.4844	275853.9769
A0A1W6S96	GTF2I-BRA	6	4	4	4	955	107.6	7.94	493677.168	367836.6422
Q5T749	Keratinoc	16	5	7	5	579	64.1	8.27	560274.0723	1482452.231
H3BRE2	IST1 homo	20	1	2	1	105	12.1	8.69	79162.64063	
B2RTS4	Centrosom	6	6	6	6	1486	164.5	7.24	213897.4805	838522.0183
Q59E91	DNA-direc	9	7	8	7	1072	121.2	8.69	333706.2715	1088502.089
A0A7D5Q2L	NAD(P) de	11	2	3	2	373	41.9	8.06	261544.8711	
P62879	Guanine n	21	2	2	2	340	37.3	6	281731.4688	
E9PC52	Histone-b	16	2	3	2	416	46.9	5.07	340480.7344	
P23396	40S ribos	27	6	8	6	243	26.7	9.66	1125241.844	2332549.13
Q4ZG57	DNA repli	6	2	2	2	785	88.9	5.6	2412240.813	
P56537	Eukaryoti	14	1	2	1	245	26.6	4.68	569914.75	
P51610	Host cell	3	2	3	2	2035	208.6	7.46	78728.35938	
P42166	Lamina-as	9	2	2	2	694	75.4	7.66	189556.7344	
Q9UBB4	Ataxin-10	9	2	2	2	475	53.5	5.25	109043.9375	
P49721	Proteasom	26	2	2	2	201	22.8	7.02	98470.49219	
Q5JSD2	Voltage-d	21	1	2	1	194	21.2	7.93	293705.2813	
P26373	60S ribos	20	4	8	4	211	24.2	11.65	1729257.32	4388982.411
Q12769	Nuclear p	4	2	2	2	1436	162	5.5	336810.793	
Q13867	Bleomycin	11	3	3	3	455	52.5	6.27	187757.0234	
F8WCU9	AT-rich i	4	5	6	5	1762	188.9	7.46	174564.8828	462289.4317
P12236	ADP/ATP t	18	5	8	2	298	32.8	9.74	1062281.188	1395914.683
D3DPS3	Arginine/	11	6	6	6	840	92.6	10.11	53375.52051	502018.7838
O95373	Importin-	8	5	5	5	1038	119.4	4.82	510114.0313	211362.4386
O60684	Importin	13	2	2	2	536	60	4.98		
P60900	Proteasom	24	2	2	2	246	27.4	6.76	490272.7813	37781.21926
B4DI38	Adenylyl	4	1	2	1	452	49	8.21	638832.625	
Q32Q75	Eukaryoti	13	1	2	1	217	25.1	6.15	120133.7109	
A0A590UJ5	Cullin-1	8	2	2	2	759	87.6	7.78	998104.3125	48227.61306
P15121	Aldo-keto	17	2	3	2	316	35.8	6.98	317226.8594	
A8K4K9	ER membra	22	2	2	2	297	34.8	6.81	138569.7813	
Q53HG0	Eukaryoti	14	2	2	2	352	39.8	6.54	560350.5625	
P53618	Coatomer	4	2	3	2	953	107.1	6.05	174706.4219	25029.63995
A8K3H8	cDNA FLJ7	8	2	2	2	589	65.3	5.11	269579.2813	
X6RJP6	Transgeli	20	2	2	2	187	21.1	7.81	105398.1328	
H7C144	Alpha-act	8	4	4	4	906	104.3	5.5	374313.1133	
Q53F35	Acidic (L	12	1	2	1	250	28.7	4.06	512494.2188	
Q86Y46	Keratin,	11	5	10	2	540	58.9	7.23	249130.4961	348175.1547
P25205	DNA repli	6	4	4	4	808	90.9	5.77	346715.0625	287952.0037
P81605	Dermcidin	23	3	6	3	110	11.3	6.54	3948274.094	5463364.96
P61026	Ras-relat	10	1	2	1	200	22.5	8.38	206825.3203	
B2R7R5	cDNA, FLJ	6	1	2	1	369	40.9	7.44	298944.4277	
Q8IWX8	Calcium b	7	6	7	6	916	103.6	9.04	295421.0605	612594.2476
Q5T8P6	RNA-bindi	5	4	5	3	1007	113.5	9.16	218279.3516	467373.4677
A0A7P0Z3Z	DNA damag	5	2	2	2	1069	119	5.24	180285.3594	
P08865	40S ribos	18	2	3	2	295	32.8	4.87	975530.2188	
E7ETY2	Treacle p	5	7	8	7	1488	152.2	8.85	463726.4766	1388882.431
K7EJV9	60S ribos	16	3	3	3	170	19.4	10.48	541032.793	
P31150	Rab GDP d	11	3	3	1	447	50.6	5.14	286165.0938	
O00268	Transcrip	8	5	5	5	1085	110	9.94	52570.04297	406766.9038
A8K9T9	phosphori	4	2	2	2	1338	144.6	5.8	112011.5156	
A0A8I5KVU	La-relate	4	6	7	6	1735	188.7	5.14	524707.9082	949786.9122
A0A087WYT	Prostagla	23	3	8	3	164	19.1	4.55	2968503.641	
Q9Y394	Dehydroge	9	1	1	1	339	38.3	8.32		
Q92896	Golgi app	5	5	6	5	1179	134.5	6.9	215427.4219	734729.1366
P52597	Heterogen	14	3	5	1	415	45.6	5.58	193249.7656	
Q09028	Histone-b	7	1	2	1	425	47.6	4.89	1072873.313	
I3L352	ATP-depen	8	1	2	1	396	44.9	8.07	877376.125	
P31040	Succinate	11	2	2	2	664	72.6	7.39	72166.57813	
P13489	Ribonucle	11	2	2	2	461	49.9	4.82	135224.4375	
H0YEH2	Pumilio h	7	6	7	6	1125	120.2	6.8	1119132.4	910575.0735
Q5QPM7	Proteasom	16	1	1	1	263	28.9	6.44		
Q96CN7	Isochoris	15	2	2	2	298	32.2	7.39	234789.0156	
A4UCS5	Peroxired	19	1	2	1	154	16.6	7.47	988585.625	56963.90042
A8KA19	Exportin-	6	2	2	2	962	109.8	5.39	702672.5781	
B3KXS5	Lon prote	6	2	2	2	923	103	6.67	315316.1875	
B2RDW0	cDNA, FLJ	26	2	3	2	149	16.8	4.22	358901.8438	16468.11428
Q9Y678	Coatomer	5	2	2	2	874	97.7	5.47	197450.2891	
P39656	Dolichyl-	8	2	2	2	456	50.8	6.55	427099.8906	
P62266	40S ribos	27	2	2	2	143	15.8	10.49	1550661.375	163303.8763
Q9UNX4	WD repeat	5	2	2	2	943	106	6.64	7261692.156	
P49411	Elongatio	11	3	3	3	455	49.8	7.61	428211.7969	578196.7417
A0A7I2V3I	Eukaryoti	11	2	2	2	396	46.7	5.54	298408.3438	
Q7Z417	FMR1-inte	8	4	5	4	695	76.1	8.7	455186.8711	1210506.846
O00264	Membrane-	16	1	2	1	195	21.7	4.7	826958.1094	
P42285	Exosome R	7	6	6	6	1042	117.7	6.52	193945.2588	603501.3282
B4DSK9	histone d	19	2	3	2	237	27.3	8.07	597958.0039	
P01040	Cystatin-	59	4	5	4	98	11	5.5	494307.2539	797260.5296
Q9Y383	Putative	6	1	2	1	392	46.5	10.01	726892.4219	
B4DN77	Calpain-2	7	2	2	2	622	71.5	4.91	802002.0625	
Q5VT52	Regulatio	5	5	6	5	1461	155.9	7.42	70423.6709	296563.0314
Q8TAS0	ATP synth	11	1	2	1	291	32.2	9.11	3662145.125	
Q2VIK8	RcNSEP1 (	13	2	2	1	319	35.4	10.01	100859.9453	243657.2316
P06702	Protein S	25	2	3	2	114	13.2	6.13	228015.7344	1640335.522
G3V4W4	Glucosami	17	1	2	1	163	18	6.9	31376.60547	
Q9Y2X9	Zinc fing	6	3	3	3	895	96.9	8.48	45660.45703	508249.0093
P58546	Myotrophi	18	1	2	1	118	12.9	5.52	95219.82031	
A0A2R8Y59	Transcrip	6	4	5	4	638	73	8.28	149808.4102	686550.0797
P11172	Uridine 5	7	1	2	1	480	52.2	7.24		
Q75MU2	Eukaryoti	17	1	2	1	156	17.1	5.31	1070448	
H0Y449	Y-box-bin	11	2	3	1	374	42	10.43	397343.4219	593984.3581
Q7RU04	Aminopept	3	2	2	2	658	73.5	6.11	405109.9688	
Q59FS7	RNA helic	9	5	5	5	676	75	8.46	183325.2637	1711424.6
X1WI28	60S ribos	26	4	5	4	200	23	10.01	399004.3936	913079.8605
A0A024R3R	Delta(14)	11	4	4	4	615	70.7	9.36	268856.2266	589342.8563
G3V241	glycine h	27	2	2	2	142	15.8	4.88		
H0YMD0	Annexin (	24	5	6	5	227	25.3	5.97	376417.1172	581578.8667
A0A024QZ4	HCG198558	14	1	2	1	121	14.4	5.29	216713.6406	
Q9UK59	Lariat de	5	1	1	1	544	61.5	5.47	217476.9844	
B4E2U0	6-phospho	13	2	3	2	461	50.8	8.18	23129.89648	
A0A0A0MSA	Band 4.1-	7	5	5	4	1046	116.1	5.22	101831.3867	440075.531
P12532	Creatine	5	1	1	1	417	47	8.34		
Q01085	Nucleolys	5	1	2	1	375	41.6	7.74	112028.1406	
P12273	Prolactin	36	5	5	5	146	16.6	8.05	576953.4297	1392669.561
J3QTR3	Ubiquitin	32	3	6	3	106	12.2	10.14	1926310.371	2035467.666
A0A804HL5	isoleucin	3	2	2	2	1237	141.5	6.2	355984.5	193484.349
P30050	60S ribos	38	4	4	4	165	17.8	9.42	248905.0801	126986.2568
P54819	Adenylate	18	2	2	2	239	26.5	7.81	200628.7969	
K9MS24	Spectrin	1	1	2	1	2364	274.2	5.55	105079.4766	
A0A7I2YQG	Ribosomal	17	3	5	3	216	25.7	11.27	1141818.623	3444192.626
Q02952	A-kinase	2	1	1	1	1782	191.4	4.41		
Q5BJH1	PSAP prot	12	1	2	1	240	26.3	6.04		
A0A7P0T9A	Adenylosu	10	2	2	2	513	57.9	7.43	19267.14844	
Q92804	TATA-bind	15	5	7	3	592	61.8	8.02	490962.0625	1889551.891
B4DLV7	Rab GDP d	11	3	3	1	449	51.1	8.18	830105.9844	10089.87997
B4DV79	Eukaryoti	11	5	5	5	738	85.1	5.26	282450.2148	167463.0264
H0YJG7	Activator	27	2	2	2	216	23.8	5.34	84103.9375	
C9JFN4	eIF5-mimi	24	2	2	2	216	24.3	8.34	424404.4063	
Q15084	Protein d	13	2	2	2	440	48.1	5.08	350141.4688	
B4DRH6	enoyl-CoA	6	2	2	2	717	78.3	8.92	290218.5859	
Q5D862	Filaggrin	2	3	6	3	2391	247.9	8.31	690483.6328	910912.0568
Q7Z4W8	Heparin-b	20	1	2	1	128	14.8	9.32	819076.7031	
Q9NZI8	Insulin-l	12	4	5	4	577	63.4	9.2	275271.6484	261147.4878
P46781	40S ribos	22	4	5	4	194	22.6	10.65	712064.4648	1512906.36
B4DN60	cDNA FLJ5	11	1	1	1	299	34.9	5.02	82757.42188	
B4DE40	cDNA FLJ5	19	2	2	2	275	31.1	4.93	225832.9609	
B4DTA2	cDNA FLJ6	17	3	3	2	271	30.2	8.72	575002.875	
A0A8V8TQJ	DNA misma	3	2	2	2	1362	152.9	6.92	26394.31836	
B2RCT6	cDNA, FLJ	10	2	2	2	499	55.7	5.62	127593.6797	
B4DNL5	Protein d	11	2	2	2	492	55.3	4.79	1132811.469	
E9PBS1	Bifunctio	7	1	1	1	413	45.6	6.68	444748.0313	
Q5SZ64	Splicing	32	2	2	2	190	20.7	6.2		
A0A0A0MTB	WD repeat	2	1	1	1	895	99.3	7.36		
A0PJ47	SAFB2 pro	17	5	7	1	526	56.9	4.79	122791.3281	
Q96EP5	DAZ-assoc	10	2	3	2	407	43.4	8.56	920301	
P18085	ADP-ribos	37	3	3	1	180	20.5	7.14	31661.51563	
H7C3T4	Peroxired	20	2	2	1	161	18.3	6.19	345255.875	
Q13451	Peptidyl-	6	1	1	1	457	51.2	5.9	418605.8125	
P62081	40S ribos	27	2	2	2	194	22.1	10.1	566342.3125	
Q15691	Microtubu	21	3	3	3	268	30	5.14	409953.5898	17211.53205
B4DZQ5	Serine/th	5	4	4	4	993	115.4	10.27		327324.7734
Q52LJ0	Protein F	9	3	4	3	433	45.5	8.69	180910.4688	133847.5775
Q5QTS3	60S ribos	19	4	6	4	203	23.6	11.02	3702013.773	4431630.325
P38919	Eukaryoti	7	1	1	1	411	46.8	6.73	242758.7031	
B3KUZ8	Aspartate	12	2	2	2	371	41.3	8.84	1118541.574	
Q6ZTI3	cDNA FLJ4	14	2	2	2	423	48.4	5.38	225741.3438	
Q13155	Aminoacyl	18	2	3	2	320	35.3	8.22		
Q6ZMY0	cDNA FLJ1	4	5	8	5	1340	150.9	8.22	254004.7539	442591.9489
Q5U0A0	Proteasom	27	3	3	3	241	26.4	4.79	649441.4609	
Q96K97	cDNA FLJ1	14	2	2	2	335	38.2	6.3	219357.3281	40779.79283
M0R0F0	40S ribos	15	2	3	2	200	22.4	9.55	78976.32813	234460.9427
Q9UBV8	Peflin OS	6	1	2	1	284	30.4	6.54	47674.96484	
P46977	Dolichyl-	4	1	2	1	705	80.5	8.07		

P31944	Caspase-1	21	6	6	242	27.7	5.58	57325030.2	19842176.56
Q6PCB5	Lysine-sp	9	7	6	846	94.8	8.78	24854046.16	19883709.25
K7EJV9	60S ribos	26	5	5	170	19.4	10.48	93256941.92	101453436.5
Q53EP4	Dolichyl-	14	8	8	607	68.5	6.38	6196139.062	12677168.63
Q92973	Transport	8	6	6	898	102.3	4.98	10160846.65	10471937.31
D3DVC4	Nestin, i	6	9	9	1621	177.3	4.36	4488989.719	10367080.56
E7ENY0	Alpha-add	15	7	7	663	73.4	6.55	12566703.03	9420883.875
D6RBZ0	Heterogen	20	6	5	327	35.7	6.95	43046007.23	38097229.13
Q10567	AP-1 comp	9	8	1	949	104.5	5.06	1002731.052	1134505.75
B5MDF5	GTP-bindi	23	5	5	233	26.2	7.01	31366549.21	30588003.5
A8K7F6	RNA helic	20	8	8	406	46.1	5.48	18665039.09	21236725.38
Q96P70	Importin-	7	6	6	1041	115.9	4.81	9600547.309	11338001.38
A0A0B4J1V	HCG203999	8	6	6	794	87.9	9.51	4518592.933	12054027.5
A0A8Q3SIC	Myosin li	35	7	5	217	23.8	6.57	22112748.92	28910503.75
M0R2B7	DNA polym	8	9	8	1133	126.3	7.21	5627770.49	6422698
H0YDF9	SCRIB ove	64	6	1	140	14.9	4.83	417770.3942	1116530.125
A0A024RDQ	Heat shoc	10	7	7	814	92.1	5.55	10124107.16	8612187.531
B7Z2N4	cDNA FLJ5	8	7	7	981	109.6	6.14	12118176.34	13260262.75
Q96PY5	Formin-li	7	8	5	1086	123.2	7.4	2704901.473	9343221.75
O00410	Importin-	8	8	8	1097	123.6	4.94	9874454.998	23844623.44
Q8IWX8	Calcium h	8	7	7	916	103.6	9.04	15205549.35	18177151.88
Q9BY44	Eukaryoti	18	9	9	585	64.9	8.87	3863386.443	11082723.94
P42285	Exosome R	9	8	8	1042	117.7	6.52	11727744.75	11939070.56
Q14008	Cytoskele	5	8	8	2032	225.4	7.8	800973.5574	7409012.469
Q96GQ7	Probable	12	9	9	796	89.8	9.28	3607067.241	13166145.56
P29508	Serpin B3	21	7	2	390	44.5	6.81	40132130.97	19375678.75
Q7Z478	ATP-depen	7	8	8	1369	155.1	8.09	5585214.737	12063435.75
A0A2R8Y7W	Cyclin-de	7	5	4	1099	121.2	8.9	2298437.75	7624412.375
A8K946	mRNA cap	14	7	7	504	57.7	7.69	17811835.31	7187270.375
M0R0R2	40S ribos	20	4	4	225	25.3	9.76	49307628.83	46145449.88
Q9H501	ESF1 homo	10	8	8	851	98.7	5.11	5550065.487	19209025.06
Q53FN0	Serine/ar	21	5	5	221	25.5	11.85	579012067.4	83463029
A0A494C05	NF-kappa-	22	6	6	414	47	10.14	27273789.41	22612529.25
Q9NZW5	Protein F	16	8	8	540	61.1	6.18	12079079.54	9080298.563
Q9H8H2	Probable	10	7	7	851	94	9.99	1785699.869	10303881.66
O15511	Actin-rel	39	5	4	151	16.3	5.67	41191418.57	36370020.5
Q08J23	RNA cytos	11	6	6	767	86.4	6.77	880017.3166	4512805
P62805	Histone H	50	5	5	103	11.4	11.36	32786780.95	16830418.56
Q9Y678	Coatomer	9	6	5	874	97.7	5.47	6390828.801	8374195.188
O94906	Pre-mRNA-	8	7	7	941	106.9	8.25	3144488.487	3716869.25
H0Y938	Coatomer	9	7	7	898	101.5	5.82	8506538.765	14155602.25
P68431	Histone H	29	2	1	136	15.4	11.12	12581260.91	6494423.5
Q86Y46	Keratin,	9	6	2	540	58.9	7.23	48705780.07	17874928.75
Q6UWP8	Suprabasi	12	3	3	590	60.5	7.01	6463549.19	523338.1875
A0A6I8PL4	leucine--	8	7	7	1130	129.1	7.87	3424168.496	7693610.938
Q96EY7	Pentatric	10	7	7	689	78.5	6.42	4652205.677	15117790.81
Q86YP4	Transcrip	14	6	6	633	68	9.94	3831311.224	11689914.75
H3BRG4	Cytochrom	15	5	5	412	44.6	9	7503307.836	9605803.563
P62269	40S ribos	39	8	8	152	17.7	10.99	167586570	91657842.88
O14617	AP-3 comp	7	7	7	1153	130.1	8.48	5762037.022	11725213.56
Q13200	26S prote	7	5	5	908	100.1	5.2	6458861.089	6794776.813
Q93009	Ubiquitin	8	8	8	1102	128.2	5.55	10957301.21	13833630.31
Q53F48	Heterogen	18	4	4	346	36.9	7.34	9973744.688	3371423.5
E7ENQ1	Mitogen-a	7	7	7	1154	132.1	7.53	9555967.952	17103942.44
O60763	General v	6	6	6	962	107.8	4.91	8695485.778	9943189.375
Q06787	Fragile X	13	9	7	632	71.1	7.42	28002764.11	23602435.44
Q9P258	Protein R	19	8	8	522	56	8.78	25691723.04	33718754
Q86U86	Protein p	4	7	7	1689	192.8	6.89	1064430.503	11092492.06
A0A494C10	Protein K	10	6	6	709	83.2	5.17	6230858.392	12762453.88
Q01650	Large neu	11	5	5	507	55	7.72	10224116.59	14634206.5
Q9NZR1	Tropomodu	27	8	7	351	39.6	5.27	13049929.59	14867321.84
J3QTR3	Ubiquitin	41	4	4	106	12.2	10.14	127584739.6	98941695.75
O15145	Actin-rel	31	5	5	178	20.5	8.59	44677818.18	27264186
Q7Z3Y8	Keratin,	12	7	4	459	49.8	5.05	38052472.23	15970310.94
Q9UP60	SNC73 pro	15	5	5	384	40.9	6.15	14832304.08	3394485.938
P26196	Probable	16	6	6	483	54.4	8.66	12075140.81	11715948.25
H0YN88	40S ribos	23	4	4	136	15.9	9.91	34156124.33	53606665.13
B4DKZ9	threonine	12	7	7	602	70.3	8.56	6605136.73	4595895.531
Q13136	Liprin-al	6	6	6	1202	135.7	6.29	5780089.16	3661134.438
Q6ZN55	Zinc fing	6	5	5	896	98.8	8.07	1211911.162	12243646.63
Q96T88	E3 ubiqui	9	6	6	793	89.8	7.56	1722215.602	6916730.875
Q9BVI4	Nucleolar	12	6	6	516	58.4	7.49	4946558.205	15476936.44
P46779	60S ribos	45	8	8	137	15.7	12.02	165510523.3	201838497
O75475	PC4 and S	12	6	6	530	60.1	9.13	11112747.06	23069061.5
Q9NWK9	Box C/D s	21	7	7	470	53.9	5.8	4458658.972	10309075.19
A0A7I2V36	GRB10-int	7	7	7	1081	125.8	5.45	7691422.357	15814242.5
P08621	U1 small	17	8	8	437	51.5	9.94	35314995.25	32491050.88
Q7L7L0	Histone H	49	4	1	130	14.1	11.05	15308904.83	9175284
Q53GY0	Plastin 3	11	7	7	630	70.7	5.68	8231381.832	13500939.31
A8K644	Splicing	11	5	2	494	56.7	11.52	11855426.05	23060344.88
Q16527	Cysteine	42	6	6	193	20.9	8.62	20265052.92	14049667.19
Q12769	Nuclear p	5	7	7	1436	162	5.5	10514004.77	12998419.31
Q16531	DNA damag	5	7	7	1140	126.9	5.26	5910249.867	9615658.313
A0A3F2YNW	Inositol	2	5	1	2750	312.8	6.04		1327003.75
B1Q2B0	URCC5 OS=	7	5	5	911	101.2	8.43	3429294.7	11952589.75
B4DVQ5	Eukaryoti	8	6	6	898	103.6	5.53	7095591.712	7477030.938
Q8IW35	Centrosom	8	6	6	865	96.9	5.02	8951411.179	9744935.625
A0A0S2Z4X	Epididymi	8	6	4	852	94.3	6.73	4175451.366	7567468.875
P32969	60S ribos	21	4	4	192	21.9	9.95	18679868.76	35745510.25
Q9NRL2	Bromodoma	4	6	6	1556	178.6	6.6	1888137.972	10279149.44
Q92945	Far upstr	13	7	7	711	73.1	7.3	10622424.44	9447912.5
Q96CS0	Ribosome	15	6	6	476	54.2	10.36	13400643.85	23530131.25
O96028	Histone-l	6	6	6	1365	152.2	8.69	1091283.06	7617852.5
B4DTK7	cDNA FLJ6	7	5	5	977	108.9	8.44	17487015.8	30637687
Q96JN8	Neuralize	4	5	5	1562	166.8	5.86	2131677.647	8666521.25
O00425	Insulin-l	9	4	3	579	63.7	8.87	1381953.22	4564224.844
P01040	Cystatin-	46	4	4	98	11	5.5	96039244.89	24728098.19
P61160	Actin-rel	17	6	6	394	44.7	6.74	13433899.83	14193381.59
Q96GA3	Protein L	16	6	6	475	54.8	4.91	5849404.064	17232863.63
P46783	40S ribos	33	6	6	165	18.9	10.15	48068499.76	43501724.25
A0A8Q3WKT	RNA bindi	14	5	5	447	46.9	6.62	1570015.736	9085405.625
Q9NTI5	Sister ch	5	7	6	1447	164.6	8.47	1808580.083	10447838.88
Q2L6I2	ABC50 pro	7	4	4	807	91.6	7.68	2685372.19	5013795.813
Q8WYL5	Protein p	8	6	6	1049	115.4	6.25	3359172.989	2471610.25
Q9P035	Very-long	14	5	5	362	43.1	8.94	23326708.39	128344036.8
Q9BZ95	Histone-l	4	3	3	1437	161.5	8.21	502318.1373	3275686.938
B7Z6Z4	Myosin li	20	5	3	238	26.7	5.08	2511433.982	3894170.875
B4DM21	cDNA FLJ5	13	5	3	490	55.1	7.72	12074197.87	11422860.75
Q13625	Apoptosis	9	7	7	1128	125.5	6.07	6323027.206	11178061.19
Q9BXP5	Serrate R	7	7	7	876	100.6	5.96	12835542.19	7144659.219
Q92522	Histone H	23	4	4	213	22.5	10.76	26369584.76	19074840.75
Q5VUJ6	Leucine-r	9	6	6	765	84.5	6.55	9391972.095	8459310.188
Q6UN15	Pre-mRNA	10	4	4	594	66.5	5.59	5661651.967	4500784.563
O75128	Protein c	5	5	5	1261	135.5	7.75	2395516.57	6908011.813
B5BU25	Splicing	14	4	4	471	53.1	9.09	8191010.102	18277320.88
Q16777	Histone H	50	4	1	129	14	10.9		
Q53FG5	DNA repli	10	6	6	734	82.2	8.28	4975807.994	10944752.38
Q8WUM5	Gem (Nucl	7	7	7	1058	120	6.09	4616113.208	5486997.344
A0A8I5KV9	Eukaryoti	4	7	2	1895	207.8	6.54		1481980
Q9UEG4	Zinc fing	8	5	5	869	96.6	7.93	1941605.538	8351724.875
Q8NI62	Ribosomal	68	5	1	79	8.8	8	1987779.124	2200183.25
Q53FI7	Four and	21	5	5	280	31.9	8.22	6755081.603	9964617.063
A8K2U0	Alpha-2-m	4	5	5	1454	161	5.73	4554371.659	909481.2813
Q5TGY3	Transcrip	4	4	4	1603	168.2	9.04	329436.4868	3936223.188
Q5K634	SCCA2/SCC	15	6	1	390	44.6	6.39	2460330.549	11777782
A0A0S2Z4Z	Serine/ar	7	5	5	918	103.9	11.84	20407003.22	21402446
O75909	Cyclin-K	12	5	5	580	64.2	8.41	3893959.601	9226594.625
W0S1J7	PJA2/FER	6	3	3	858	96.2	4.67		2510965.438
B4DY09	Interleuk	18	5	5	352	38.9	4.94	2575017.575	8754191.688
Q06830	Peroxired	32	7	7	199	22.1	8.13	44158791.51	43532984
A0A494C0R	Clustered	5	6	5	1348	150.4	6.11	4024196.592	4924735.563
P61254	60S ribos	28	6	6	145	17.2	10.55	174498464.8	188928168
Q9Y224	RNA trans	20	6	6	244	28.1	6.65	50689045.83	17913679
Q15758	Neutral a	10	4	4	541	56.6	5.48	5983560.609	9035023.375
Q96IZ0	PRKC apop	11	3	3	340	36.5	5.41	6568729.462	5942888.656
Q5VVC8	60S ribos	27	5	5	167	19	9.8	76182061.74	72983419.75
Q9Y5Q9	General t	6	5	5	886	101.2	5.07	2380774.079	8045201.813
P42356	Phosphati	3	5	5	2102	236.7	7.06	1469294.456	5648048.875
A0A8I5KQH	Golgin su	5	6	6	1276	143.6	5.62	4015104.295	709845.5313
Q9NZT1	Calmoduli	21	2	2	146	15.9	4.44	31728355.42	5938547
Q9Y5A9	YTH domai	8	4	4	579	62.3	8.79	4788200.553	22883719.25
B4DRP9	cDNA FLJ5	9	6	6	731	76.4	8.32	5817261.772	7619330.313
Q8ND56	Protein L	10	5	5	463	50.5	9.52	7827945.723	6221545.266
P51116	RNA-bindi	8	4	1	673	74.2	6.23	446344.9539	922726.1875
B2RTX8	WAPAL pro	4	4	4	1184	132.1	5.48	6586480.24	7167570.5
Q53GR7	Solute ca	8	4	4	675	74.1	8.38	894663.1997	6377136.625
Q14244	Ensconsin	9	6	6	749	84	9.61	9635729.567	8800367.438
Q8TAS0	ATP synth	18	5	5	291	32.2	9.11	2705248.012	10125242.88
O95202	Mitochond	7	5	5	739	83.3	6.7	5268405.723	7030513.875
Q96FS4	Signal-in	10	7	7	1042	112.1	6.6	10853127.88	7747996.625
Q9NX58	Cell grow	13	3	3	379	43.6	9.57	3932749.635	8599325.188
B4E2B5	MICOS com	19	5	1	404	43.2	9.2	1719029.833	1867965
J3KTL2	Serine/ar	25	6	6	253	28.3	10.08	29228557.47	20203046.38
A0A7P0Z4N	Protein A	12	3	3	474	53.3	4.68		7461741.375
Q9BPX5	Actin-rel	29	4	3	153	16.9	6.6	22029723.23	31482666.56
Q8N201	Integrato	3	6	6	2190	244.1	6.13	1011632.111	4532802.563
Q7L0Y3	tRNA meth	12	3	3	403	47.3	9.36	1134660.933	4496273.5
Q9H0H5	Rac GTPas	11	6	6	632	71	8.88	3053727.149	9528701.125
J3KSD8	Bleomycin	22	5	5	262	30.2	7.46	24493657.96	9404741.406
Q9Y490	Talin-1 O	2	5	5	2541	269.6	6.07	643199.7056	5343425
Q9HCY8	Protein S	44	4	4	104	11.7	5.24	16292585.89	3270285.563
Q641R2	Oxysterol	9	5	5	728	82.5	7.08	5787931.29	8861875.813
A0A8I5KV6	Transcrip	5	5	5	1251	142.6	8.16	7977111.813	8995130.188
P62851	40S ribos	30	5	5	125	13.7	10.11	97044036.33	89306612.75
I0B0K8	Truncated	2	6	6	4021	430.2	9.29	38642734.06	15197760.31
P47755	F-actin-c	28	5	3	286	32.9	5.85	1323704.78	1747683.625
P05387	60S acidi	53	3	3	115	11.7	4.54	10876298.1	6787679.25
A0A8V8TM6	HCG181174	7	6	6	934	106.3	7.99	30549589.48	21778950.13
Q9Y4W2	Ribosomal	6	4	4	734	83	4.73	5022610.362	3466054.25
B4DT66	Protein S	8	5	5	650	75.5	9.22	1900060.742	11417419.19
B4E228	cDNA FLJ5	12	5	5	470	53.9	9.76	1383298.363	5016353.438
A8K9K6	cDNA FLJ7	10	6	6	594	65.9	9.23	9133073.196	14380606.75
Q58FF6	Putative	9	5	1	505	58.2	4.73		971819.625
G3V1N3	GAS2-like	13	5	5	590	63.6	9.91	4417416.607	6989432.125

B2R4P2	cDNA, FLJ	21	3	3	2	199	22.2	8.38	557509.7813	101389.9515
P26196	Probable	14	3	4	3	483	54.4	8.66	82952.48438	36356.98485
A8MQ02	Afadin OS	3	4	4	4	1781	201.7	6.51	550564.2402	142120.6134
Q9NSD9	Phenylala	11	2	2	2	589	66.1	6.84	380936.8906	
O75663	TIP41-lik	8	1	2	1	272	31.4	5.91	332656.7422	
H0YCI4	Nucleosom	13	1	1	1	204	23.4	4.92	220927.8906	
C9J177	Protein p	18	2	2	2	289	33.7	5.22	249363.5313	
P62937	Peptidyl-	28	3	3	3	165	18	7.81	769016.168	
B3KMS0	Condensin	2	1	2	1	1401	157.1	6.61	79162.64063	
Q0VDF9	Heat shoc	6	1	1	1	509	54.8	5.59		
Q53S41	Uncharact	20	1	1	1	147	16.6	4.44	553864.875	
Q92945	Far upstr	8	5	7	5	711	73.1	7.3	306505.8867	174711.4829
H0YHA7	60S ribos	22	3	5	3	167	19	11.78	1412381.531	5712054.107
Q8N516	Elongator	2	1	1	1	1332	150.1	6.01	110364.8828	
H7C4X9	Protein k	4	4	4	4	1095	121.1	6.38	38711.03906	485602.1152
Q5TGY3	Transcrip	4	4	4	4	1603	168.2	9.04	42455.42188	307147.6247
B4DZX4	cDNA FLJ5	20	1	1	1	168	18.2	7.39		
Q14166	Tubulin--	7	2	2	2	644	74.4	5.53		
H3BS86	E3 ubiqui	11	1	1	1	213	24.4	6.06		
P61758	Prefoldin	25	2	2	2	197	22.6	7.11	400933.7734	
A0A8I5QKM	Lysine-sp	3	1	1	1	794	85.4	6.46		
P28070	Proteasom	8	1	2	1	264	29.2	5.97	227467.9219	
P11388	DNA topoi	4	3	4	3	1531	174.3	8.72	515664.3672	357935.7027
A8K6N9	calcium/c	6	1	1	1	518	58.3	7.18		
Q6MZS5	Uncharact	9	4	6	1	613	68.3	8.91	103350.1953	25554.99094
P42126	Enoyl-CoA	7	1	1	1	302	32.8	8.54		
P28482	Mitogen-a	13	2	2	2	360	41.4	6.98	32001.02344	96435.9353
Q15269	Periodic	2	1	2	1	919	102.4	6.15		
K7EKQ2	Hsp90 co-	8	1	1	1	353	41.6	6.64	231541.2813	
Q6FHH6	LANCL1 pr	7	1	1	1	399	45.2	7.75	68672.78906	
B4DY59	cDNA FLJ5	14	4	4	4	318	35.4	8.95	47920.61914	411129.5455
A0A024RDL	phosphose	17	2	2	2	252	28.1	7.37	314072.3047	
P10620	Microsoma	16	1	1	1	155	17.6	9.39	26053.77734	
Q6KB66	Keratin,	9	4	4	3	452	50.5	5.67	163049.8359	226155.8147
E9PK21	Peptidyl-	13	1	1	1	208	22.8	5.2		
A0A2R8Y7H	Ribose-ph	13	1	2	1	321	35.3	7.61		
O43583	Density-r	15	1	1	1	198	22.1	5.3	181207.125	
Q9Y5S9	RNA-bindi	18	2	2	2	174	19.9	5.72	514834.4375	
Q15323	Keratin,	10	4	5	3	416	47.2	4.88		222602.5202
A0A8I5KT5	Kinase D-	3	4	4	4	1764	195.9	6.79	83743.49023	340395.469
A0A024R1S	GTP-bindi	5	3	4	3	660	71.4	8.22	366863.4375	492080.8694
Q9UP60	SNC73 pro	7	3	4	3	384	40.9	6.15	400233.2188	280428.2507
Q0KKI6	Immunoblo	14	2	3	1	219	24	8.06	520437.1504	21003592.49
O75909	Cyclin-K	9	3	4	3	580	64.2	8.41	143153.8672	491186.3384
O95749	Geranylge	9	1	1	1	300	34.8	6.14	105914.4531	
B4DWI8	Paraspeck	7	3	4	2	463	52.5	7.25	48169.55859	209374.7888
B0QYA8	Eukaryoti	24	1	1	1	123	14.5	9.61		
B4E0D5	cDNA FLJ5	39	3	3	3	163	19.4	10.11	316241.2188	
Q9UMS4	Pre-mRNA-	8	3	4	3	504	55.1	6.61	169372.9258	273283.2342
B8ZZK2	gamma-glu	22	2	2	2	166	18.4	5.9	74181.02344	52834.22204
P31944	Caspase-1	19	4	4	4	242	27.7	5.58	247098.8652	565091.1612
O00743	Serine/th	22	2	2	2	305	35.1	5.69	80971.84375	
P24534	Elongatio	32	2	2	2	225	24.7	4.67		
A0A0A7M1X	Lamin B2,	10	1	1	1	483	53.5	7.12		
Q9UL54	Serine/th	4	4	4	4	1235	138.2	7.27	100965.2188	592493.7449
A0A8I5KPP	AP-1 comp	9	5	5	2	655	73	7.49	245427.5859	541525.573
B4DKZ9	threonine	4	2	2	2	602	70.3	8.56	420847.9063	
P61201	COP9 sign	6	1	1	1	443	51.6	5.53	278851.5	
B2R7B5	cDNA, FLJ	17	3	5	3	443	48.2	8.66	306570.084	512621.8294
Q5TA01	Glutathio	19	1	2	1	180	20.9	6.55		
P30049	ATP synth	21	1	1	1	168	17.5	5.49	263133.75	
F8VPA7	Elongatio	14	1	1	1	200	22.1	9.66	128630.1016	
F8VV59	Nucleosom	9	1	1	1	327	38	4.68	405012.875	
Q99590	Protein S	3	3	4	3	1463	164.6	8.41	54926.66211	204754.5739
A0A0C4DGG	Cell divi	3	2	2	2	1043	120.4	5.92		75842.28608
Q15631	Translin	10	1	1	1	228	26.2	6.44	1005859	
P27694	Replicati	8	2	2	2	616	68.1	7.21	98756.86719	20654.93885
P62906	60S ribos	13	2	3	2	217	24.8	9.94	1367343.102	230734.0935
A0A8I5QKX	cytochrom	12	1	2	1	273	31.3	6.6		
P27708	CAD prote	3	2	2	2	2225	242.8	6.46	72314.9375	
O95782	AP-2 comp	4	4	4	1	977	107.5	7.03	35485.86328	66032.11326
Q5U8W9	Protein a	7	2	3	2	327	37.5	5.72	1092127.734	
P51398	28S ribos	9	3	3	3	398	45.5	8.88	143994.832	219513.8462
C9JV50	Phosphati	19	1	1	1	187	21.4	5.88		
O60888	Protein C	15	1	1	1	179	19.1	5.5		
Q92878	DNA repai	1	1	1	1	1312	153.8	6.89	126958.1172	
P56192	Methionin	4	1	1	1	900	101.1	6.16	364381.75	
A0A6Q8PEX	Gangliosi	13	1	1	1	274	31.3	5.02		
A0A0U1RQT	Glutamine	9	2	2	1	342	38.5	6.54	358342.5	
Q59FA0	DNA polym	2	1	1	1	1011	111.8	7.58	251631.9844	
A0A024R4H	DNAation	22	2	2	2	268	29.4	4.61	228038.9375	
J3KS05	Chromobox	27	2	2	2	173	20	5.02	45238.05859	
J3KNF4	Superoxid	16	1	1	1	255	27.1	5.9	49421.08594	
A8K5Y7	cDNA FLJ7	2	1	1	1	1204	136.2	5.9		
A0A1U9X8Y	Chloride	15	1	1	1	240	26.8	5.26	857405.0625	
P00558	Phosphogl	9	2	2	2	417	44.6	8.1	156639.0625	
Q8WXX5	DnaJ homo	7	1	2	1	260	29.9	5.73	48879.22656	
A6NHK2	Small nuc	48	1	1	1	52	5.9	5.95	1467566	
B4DEE5	cDNA FLJ6	9	1	1	1	320	35.8	4.84	290504.2188	
B4E162	cDNA FLJ5	6	1	1	1	607	66.7	8.48	127035.8984	
A0A7P0NMY	Filamin-A	2	1	1	1	1574	167.8	6.87	234330.375	
P53396	ATP-citra	2	1	1	1	1101	120.8	7.33	276256.1563	
Q49AN9	Small nuc	48	1	1	1	64	7.1	7.18	626155.0625	
Q8IVF7	Formin-li	3	3	3	3	1028	117.1	6.65	5322291.053	8863749.567
E9PK47	Alpha-1,4	8	2	2	2	819	94	7.31	98840.66406	
Q6IAX9	SRPR prot	5	2	2	2	638	69.9	9	23702.91211	45444.61392
Q562R1	Beta-acti	9	2	3	1	376	42	5.59	652071	379723.0814
A0A024R3R	Nucleopor	2	1	1	1	981	110.4	4.91	84122.99219	
Q86VX4	Structura	4	3	3	3	1217	141.4	7.18	186820.8379	132255.549
D6W5Y5	Cold-indu	7	1	2	1	297	31.9	9.61	142896.3906	
A0A024R5G	Nuclear m	2	1	1	1	1536	174.4	5.19		
Q6P453	Ubiquitin	3	1	1	1	923	105.3	5.35	162418.6875	
Q96DI7	U5 small	10	1	1	1	357	39.3	8.1	130978.4531	
P62310	U6 snRNA-	33	1	1	1	102	11.8	4.7	696865.25	
B3KX51	cDNA FLJ4	5	1	2	1	303	34.9	5.38	395016.8438	
Q9H8H2	Probable	4	3	3	3	851	94	9.99	253402.7383	398831.4626
P20618	Proteasom	11	1	1	1	241	26.5	8.13	391261.0625	
D3DQS4	Formin bi	4	3	4	3	1017	110.2	4.75	66025.06543	578325.8419
Q14126	Desmoglei	3	3	4	3	1118	122.2	5.24	164002.6289	137690.4694
O75691	Small sub	2	2	2	2	2785	318.2	7.39		
P00966	Argininos	6	1	2	1	412	46.5	8.02	603184.1641	42064.5502
Q9UHY7	Enolase-p	28	2	2	2	261	28.9	4.78	102837.0078	
C9JYQ9	60S ribos	20	1	1	1	121	14.5	9.38	268502.3125	
Q5M7Z5	GRHPR pro	7	1	1	1	341	36.8	6.35	522884.6563	
K7ESL0	Spermidin	32	1	1	1	119	13	6.76	225917.9531	
H3BND8	Ubiquitin	3	2	2	2	909	105.5	5.58		386310.1244
O75489	NADH dehy	11	1	1	1	264	30.2	7.5	268215.9375	
H0YAC6	Lipopolys	2	1	1	1	1505	166.8	5.64		
A0A0B4J2D	Putative	10	1	1	1	268	28.1	8.27	373018.4375	
B4DT29	Actin-rel	10	1	1	1	356	40.7	5.82	502193.8125	
A0A087WVM	formate--	2	1	1	1	913	99.2	7.27	210257.8125	
A0A5C2GK1	IG c1122_	34	2	3	1	111	12.1	7.12		2902392.58
B4DW97	RNA helic	6	4	5	3	711	80	8.85	119691.8047	268050.115
A0A8V8TLR	Xaa-Pro d	6	1	1	1	507	55.9	6.16	309679.3125	
A0A7I2V3Z	Importin	7	1	1	1	399	43.5	8.87		
B4DMM1	E3 ubiqui	8	4	4	4	693	79	8.43	56777.14844	309113.2797
Q8IWR0	Zinc fing	6	5	5	5	971	110.5	7.3	308991.5547	812455.2144
Q8TCW2	DNA-direc	8	1	1	1	299	33.6	6.54		
E9PK52	Band 4.1-	6	2	2	1	811	90.9	5.86		
A0A024R32	60S ribos	15	2	3	2	157	17.5	11.66	1455183.734	1665252.076
Q96B13	Serine/th	6	1	1	1	384	44.8	6.48		
A0A024R3X	Heat shoc	37	2	2	2	71	7.5	6.68	290617.2422	
Q9GZS3	SKI8 subu	12	1	1	1	305	33.6	5.47	475664.0313	576371.8988
Q8N1N4	Keratin,	6	3	4	2	520	56.8	6.02	206119.875	206358.9465
P53597	Succinate	13	1	1	1	346	36.2	8.79		
P61009	Signal pe	13	1	1	1	180	20.3	8.62		
B2R823	RNA-bindi	6	1	1	1	252	27.9	9.73		
H0YL69	Proteasom	14	1	1	1	236	26.4	7.71	535293.4375	
P07737	Profilin-	10	1	2	1	140	15	8.27	437872.9531	
Q13185	Chromobox	16	1	1	1	183	20.8	5.33	380349.9688	
A6NDG6	Glycerol-	21	2	2	2	321	34	6.14		
B7Z3V1	Sodium/po	6	4	4	4	1020	112.4	5.35	278010.3516	117873.2472
P14174	Macrophag	26	2	3	2	115	12.5	7.88	5743058.813	65400.08799
N0E638	Casein ki	30	1	1	1	101	11.9	4.56		
A8KAK1	cDNA FLJ7	2	1	1	1	1531	174.9	5.52	106643.2969	
H3BPE3	Eukaryoti	14	1	1	1	132	15.3	5.03	425283.4063	
Q9Y3A6	Transmemb	12	1	1	1	229	26	4.84	145904.1563	
B4DPZ3	cDNA FLJ5	6	2	2	2	630	70.4	5.3	172176.25	165480.5625
Q59EC0	Adenosine	4	4	4	4	1244	137.7	8.48	396188.5195	583840.0078
Q92734	Protein T	6	1	1	1	400	43.4	5.1	1159900	
Q5T5C7	serine--t	9	2	2	2	536	61.3	7.06	512025.5156	
K7ENV7	Isochoris	13	1	1	1	174	18.9	7.75	267021.1563	
P33240	Cleavage	5	1	1	1	577	60.9	6.83	146118.0781	
O43684	Mitotic c	12	1	1	1	328	37.1	6.84	110714.4375	
H7C463	MICOS com	8	3	3	3	613	68.1	6.06	69270.04883	435596.8413
P23258	Tubulin g	10	1	1	1	451	51.1	6.14		
C9JAZ1	Metaxin-2	12	1	1	1	229	25.7	5.27	227063.3125	
P22612	cAMP-depe	7	1	1	1	351	40.4	8.59	85159.67188	295699.6312
F8WJN3	Cleavage	12	2	2	2	478	52.2	6.43	107934.4844	
Q0IIN5	VCP prote	7	1	1	1	475	52.4	5.02	80013.78125	
Q92547	DNA topoi	2	3	3	3	1522	170.6	6.96	70373.31445	188137.3319
P35998	26S prote	4	1	1	1	433	48.6	5.95	131501.8438	
B3KT66	26S prote	7	1	1	1	343	40.3	5.85		
Q69YN4	Protein v	1	2	2	2	1812	201.9	5.01	177279.7656	91222.78352
Q16658	Fascin OS	5	1	1	1	493	54.5	7.24	393311.3438	
Q8TCS8	Polyribon	4	1	1	1	783	85.9	7.77	121078.0078	
P49419	Alpha-ami	7	1	1	1	539	58.5	7.99		
Q59G24	Activated	18	1	1	1	134	15.1	9.38	362417.3438	
P98179	RNA-bindi	20	1	1	1	157	17.2	8.91		
Q9HB00	Desmocoll	6	4	4	4	840	93.8	5.53	689587.4766	765681.6237
P08133	Annexin A	8	2	2	2	673	75.8	5.6	190114.2344	
B3KM97	very-long	10	3	3	3	308	36	9.5	62288.49512	364640.4683
B4DNB9	AP-2 comp	6	1	1	1	460	52.3	9.5	39505.125	
A8K489	Pre-mRNA-	8	1	1	1	396	44.2	5.25		

A0A7P0T9U	Splicing	9	5	5	764	80.3	9.2	17087156.06	11751734
Q9H6F5	Coiled-cc	15	4	4	360	40.2	10.33	10721621.33	17961150
B4DZ54	cDNA FLJ6	5	5	5	1028	107.6	7.34	5703500.238	9018981.125
X6R700	Chromatin	22	4	4	223	23.6	12.26	13043264.63	8512918
P05109	Protein S	47	5	5	93	10.8	7.03	66478867.38	27780096.5
Q7Z682	Uncharact	7	4	4	641	69.5	9.48	68690545.79	66812770.63
A0A024R32	60S ribos	15	2	2	157	17.5	11.66	297299730.6	320276954
B7ZM24	SLC12A2 p	4	5	5	1196	129.6	6.46	3338183.592	6555274.313
Q9Y520	Protein P	2	5	3	2896	316.7	9.13	2549486.575	5435387.375
Q02040	A-kinase	8	6	6	695	80.7	9.73	8142496.996	10152791.25
P59998	Actin-rel	28	5	5	168	19.7	8.43	77700512.81	67589402
Q5TZA2	Rootletin	2	4	4	2017	228.3	5.49	3534800.493	4354336.063
A0A087X2D	Serine/ar	45	4	3	95	10.3	5.14	19812874.28	19191284.5
Q9BY77	Polymeras	15	5	5	421	46.1	9.99	8725787.232	9530925.438
P81605	Dermcidin	23	3	3	110	11.3	6.54	445281647	130621057.7
A0A024RAC	Elongin-A	6	4	4	772	87.2	9.57	6618790.984	9127327
B9ZVT1	RNA-bindi	7	6	6	881	102.6	7.53	11868082.21	3339173.719
K7ELC7	60S ribos	28	3	3	144	16.5	10.58	16410985.33	22218576.13
B7Z809	C-1-tetra	5	5	5	1020	110.4	8.56	5457155.972	4693701.188
Q9HCM4	Band 4.1-	6	4	4	733	81.8	6.58	2561187.5	5124642.125
A0A024R4Z	HCG203944	8	3	3	569	63.3	7.36	2232820.164	10059329.25
P26641	Elongatio	10	5	5	437	50.1	6.67	7249532.636	7210875.438
O75342	Arachidon	8	5	5	701	80.3	7.64	15257129.45	7739151.938
A0A8V8TPX	Nuclear p	4	5	5	1661	179.6	6.6	5758357.099	5526479.844
P25205	DNA repli	8	6	6	808	90.9	5.77	9881407.905	10015309
A0A8Q3WLD	H/ACA rib	13	5	5	485	54.3	9.48	7997032.995	11137938.88
Q9BTX1	Nucleopor	11	6	6	674	76.3	9.09	2464530.777	9541346.875
A0A7I2YQK	Inosine-5	11	4	4	513	55.5	7.02	4588704.679	4226675.625
P31689	DnaJ homo	14	4	4	397	44.8	7.08	3570436.769	9662625.438
L0ERK8	Tumor sup	39	3	1	99	11.4	7.85	2815444.563	
L0EP99	Tumor sup	39	3	1	99	11.4	7.85	2876841.958	2549116.25
Q6IS14	Eukaryoti	28	3	3	154	16.8	5	1938586.47	1238545.531
B3KMS0	Condensin	4	6	6	1401	157.1	6.61	3164900.349	5956181.688
E9PB61	THO compl	23	4	4	264	27.5	11.05	4294949.103	2381486.5
P08729	Keratin,	8	5	1	469	51.4	5.48	2215209.42	442205.9688
P68871	Hemoglobi	35	4	2	147	16	7.28	13131522.38	1894830
F8WEF5	PHD and F	4	4	4	1647	178.3	8.98	3234200.033	6501948.5
B2RE88	Phosphate	15	6	6	361	39.9	9.36	43584708.24	94320404.88
Q6N096	Uncharact	12	5	3	466	50.9	8.06	29008801.09	10506216.44
H3BS42	Zinc fing	9	4	4	509	56.6	8.27	1170782.061	6084508.25
Q0KKI6	Immunoblo	21	3	2	219	24	8.06	102501506.4	1029345321
Q71UI9	Histone H	30	2	1	128	13.5	10.58		
Q5T200	Zinc fing	3	5	5	1668	196.5	9.42	3587344.681	10475375.44
B4DDU6	T-complex	10	5	4	503	55.4	5.43	8029991.879	5983963.375
O43175	D-3-phosp	9	4	4	533	56.6	6.71	4982273.03	5409512.25
P48047	ATP synth	21	4	4	213	23.3	9.96	4407353.233	4639514.75
Q9Y5B9	FACT comp	5	5	5	1047	119.8	5.66	6545753.214	8386224.375
Q96PE2	Rho guani	3	6	6	2063	221.5	6.29	396686.5037	5203330.313
Q86VR6	RNA helic	5	3	3	745	85.8	9.22	323398.3805	4009864.75
Q03252	Lamin-B2	9	6	3	620	69.9	5.59	12516162.27	10781474.88
B2RAM6	cDNA, FLJ	4	4	4	1056	119.1	5.72	3805164.79	3265073.375
P50402	Emerin OS	25	5	5	254	29	5.5	8763656.546	18452281
O75223	Gamma-glu	22	4	4	188	21	5.14	17875939.9	950133.125
B4DK64	protein-s	4	5	5	1450	161.1	5.31	4967208.824	7908197.313
A0A590UK8	Serine/ar	3	4	1	1090	121	6.84	1264515.809	3249663
O95071	E3 ubiqui	2	4	4	2799	309.2	5.85		3072428.781
P28331	NADH-ubiq	6	3	3	727	79.4	6.23	3584286.323	11528418.88
B4DY85	cDNA FLJ6	9	3	3	516	55.4	6.06	2471779.519	3804250.438
A0A7P0TB9	RNA helic	10	4	4	423	46.9	7.47	5669860.719	7463856.875
P69905	Hemoglobi	30	4	4	142	15.2	8.68	19215821.92	4343823.813
Q92896	Golgi app	3	4	4	1179	134.5	6.9	5792389.815	6372325.063
A8K0D2	cDNA FLJ7	10	4	4	475	54.4	8.78	4889330.99	8344858.5
Q9UQ88	Cyclin-de	6	5	5	783	91.3	5.36	4482054.207	6941597.875
A0A2R8Y81	40S ribos	29	3	3	150	16.1	10.05	5238415.263	7944455.5
H7C3M7	FERM, ARH	10	4	2	482	51.6	4.91	4428161.734	7291656.25
Q9Y4B6	DDB1- and	4	6	5	1507	168.9	5.06	2381346.751	8307696.625
O60293	Zinc fing	2	4	4	1989	226.2	8.13	127701.6113	5314795
A0A1U9X9D	Valine--t	4	4	4	1264	140.4	7.59	2397807.137	4469065.688
Q9H9B4	Siderofle	14	4	4	322	35.6	9.07	6856008.194	14215160.5
Q14966	Zinc fing	3	5	5	1978	220.5	6.38	893848.5698	2865875.125
P08865	40S ribos	15	5	5	295	32.8	4.87	28120029.33	41476815.47
P55084	Trifuncti	11	5	5	474	51.3	9.41	5618301.697	6589557.625
Q16629	Serine/ar	18	4	3	238	27.4	11.82	19303134.9	20122615.13
Q155Q3	Dixin OS=	8	5	5	683	77.4	6.24	3427808.254	6516513.063
A0A2U3TZI	Centrosom	7	4	4	711	79	8.31	2837635.474	5338042.25
Q8IVF7	Formin-li	4	4	1	1028	117.1	6.65	204135.9727	390316.9375
M0QXA7	Protein W	5	4	4	968	103.2	9.52	473521.2493	5054026.656
A0A8F5XQU	Endoribon	3	5	5	1708	193.3	5.86	751179.104	5381734.063
V9GYM8	Rho guani	4	4	2	1031	116	7.37	3662232.952	4075614.25
Q8WVV4	Protein F	9	5	5	589	68	6.32	11058076.86	3858411.375
A6QKW0	SHINC3 OS	19	5	5	247	27.9	9.7	21491116.15	38165931.5
P22531	Small pro	61	3	3	72	7.9	8.31	42718645.3	12221388.22
P22528	Cornifin-	43	3	1	89	9.9	8.48	82676731.22	15909797
P35052	Glypican-	12	5	5	558	61.6	7.3	2333204.131	10135253.5
Q12986	Transcrip	5	5	5	1120	124.3	8.24	3107897.818	7434953.25
Q8NE02	AKAP8 pro	7	5	5	692	76.1	5.12	5999040.238	8078666.438
P51398	28S ribos	10	3	3	398	45.5	8.88	1307391.435	3039170.125
A0A8V8TMM	Pyruvate	13	4	4	344	37.4	6.39	9359907.959	7902265.188
A0A024R55	MAP/micro	9	5	3	551	61.6	9.51	4303112.857	5054278
A8K818	cDNA FLJ7	10	3	3	510	54.9	8.19	3676965.024	8822769.25
Q99590	Protein S	2	3	3	1463	164.6	8.41	2856101.526	5365690.5
P50991	T-complex	9	4	3	539	57.9	7.83	2017800.902	3376796.875
Q9BZI7	Regulator	10	5	5	483	57.7	9.48	8834172.541	4847951.25
Q7L2J0	7SK snRNA	8	5	5	689	74.3	9.57	4778561.858	6764347.75
A0A2R8Y6G	phosphopy	10	4	4	434	47.3	6.99	7501448.759	2981783.75
Q9H7N4	Splicing	4	4	4	1312	139.2	9.25	4156865.18	5397234
Q92576	PHD finge	3	5	5	2039	229.3	6.96	1717444	9087231.625
B5MDQ0	DNA excis	4	5	5	1127	126.9	5.4	20667090.41	11632574.88
Q7L590	Protein M	4	3	3	875	98.1	8.75	1462238.825	4099530.438
Q9BRJ6	Uncharact	17	2	2	194	22.1	9.64	614736.914	4230494.063
A0A0G2JIC	28S ribos	15	2	2	215	24.3	8.56		2994280.5
B8QGS9	Truncated	4	3	3	824	91.2	9.16	996740.3289	3512674.813
A0A286YEY	Immunoglo	10	4	2	395	43.8	6.52	4167261.64	1793563.5
B2R5Y4	cDNA, FLJ	8	5	5	557	65.6	6.64	6543350.185	13379058.5
B4DY59	cDNA FLJ5	10	3	3	318	35.4	8.95	2313575.823	4570095.375
Q9P2I0	Cleavage	5	3	3	782	88.4	5.11	3108243.602	2119794.25
B4DLB0	Upstream	7	3	3	505	58.3	5	2336395.789	4823793.625
A0A7P0T99	Acetyltra	7	5	5	636	67.8	7.84	13617808.39	6926569.625
A0A5C2G7I	IGH c572_	16	1	1	118	12.7	5.4	10547890.29	443613.4688
A0A161I20	Lactotran	8	5	5	711	78.3	8.18	5027000.636	1909152.25
Q6NUR7	Ezrin OS=	7	4	4	586	69.2	6.27	4093253.411	6241391.75
Q96QA5	Gasdermin	10	3	3	445	49.3	5.29	7651242.152	2215656.688
Q00341	Vigilin O	4	4	4	1268	141.4	6.87	3356239.415	4369120.063
Q96JQ2	Calmin OS	5	3	3	1002	111.6	4.94	1259648.686	3500431.125
B2RDD7	Protein a	6	4	4	637	72.7	6.29	6934848.545	7577742.125
P07996	Thrombosp	4	4	4	1170	129.3	4.94	9342353.749	639870.625
Q9BXS6	Nucleolar	11	4	4	441	49.4	9.91	14627139.17	14912250
Q9UHK0	FMR1-inte	7	3	3	495	56.3	9.13	2842518.724	9359479.875
Q9H444	Charged m	19	4	4	224	24.9	4.82	5914797.329	1227948.563
B4E0B9	5'-3' exo	6	4	4	874	99.9	8.02	3070718.985	8259882.25
H0YLY0	non-speci	3	6	1	1685	191.5	6.3	936172.3308	2067580.625
B2R5U1	Staphyloc	6	5	5	885	99.6	6.96	5616658.425	5502470.313
Q8IW75	Serpin A1	10	4	4	414	47.1	9.29	6668961.255	
F5H7W8	Protein C	20	4	4	293	31.7	9.32		4894081.563
Q9UBF2	Coatomer	3	2	1	871	97.6	5.81	1037376.032	1127564
P07305	Histone H	16	3	3	194	20.9	10.84	21957801.27	24345232
P29692	Elongatio	13	2	2	281	31.1	5.01	3170705.226	2185097.188
M1V490	KIF5B-RET	3	3	3	1173	134.1	6.04	2528432.915	2367233.063
B5MCF9	Pescadill	6	4	4	571	66	7.5	2489428.797	8050708.75
F2Z2E2	Ras GTPas	3	5	3	1588	179.4	7.66	513620.9726	1479511.625
E7EX17	Eukaryoti	6	3	3	616	69.7	5.67	7792903.198	4416765.375
Q12792	Twinfilin	11	4	4	350	40.3	6.96	5699735.289	9247319.563
D9HTE9	Plasma me	16	5	5	318	35	9.99	11953167.14	10346790
A0A5C2GJ5	IG c360_l	17	1	1	108	11.7	6.51	3501355.035	
B2R791	U4/U6 sma	6	4	4	683	77.5	9.5	5074251.104	9605844.5
A0A140VKH	Testis ti	5	3	3	646	71.6	5.07	5586087.108	11316102
M0R210	40S ribos	28	4	4	129	14.4	10.23	8751283.533	11042286.88
Q13144	Translati	5	4	4	721	80.3	5.08	1941700.153	5440965.563
E9PDP5	Ankyrin r	3	5	2	1565	166.6	5.01	6612253.319	7353804.25
H0YBE0	Protein L	37	3	2	60	6.4	8.66	14266954.06	23145879.75
B7ZLF8	Cornifin-	34	3	1	89	9.9	8.76	48900527.08	18186748.94
Q9H0S4	Probable	7	3	3	455	50.6	9.1	1469750.724	9163756.125
Q9NY12	H/ACA rib	28	5	4	217	22.3	10.92	3368889.719	17559950.31
M0R0P8	Unconvent	2	4	4	2157	243.2	8.78	2233741.247	4989209.375
P53618	Coatomer	5	3	3	953	107.1	6.05	3844863.465	5334861
A0A0U1RQZ	Zymogen g	26	3	3	178	19.6	5.95	5619447.343	2899540.688
O75179	Ankyrin r	2	4	1	2603	274.1	6.52	2126726.026	1187641.5
P47929	Galectin-	24	3	3	136	15.1	7.62	12576544	3007762
Q9Y5K6	CD2-assoc	8	4	4	639	71.4	6.4	50465164.08	339787512.5
A8KAH1	Phosphati	5	2	2	473	55.4	8.38	1015753.293	7508379.5
Q9C0C9	(E3-indep	4	4	4	1292	141.2	5.12		2644332.438
Q15717	ELAV-like	13	4	4	326	36.1	9.17	6825741.651	5986266.063
Q6P158	Putative	2	3	3	1386	155.5	7.71	5232545.639	6126402.25
J3KNR0	non-speci	6	5	3	776	86.9	9.52	5887056.666	9263205.5
P56192	Methionin	7	4	4	900	101.1	6.16	2164327.953	4659516.5
Q9H792	Inactive	3	4	4	1746	193	6.89		5440067.5
Q9BQQ5	Ribosomal	27	3	3	106	12	11.46	95728110.44	93218307
B5BU01	Eukaryoti	11	4	4	333	38.3	5.94	4166828.761	11186485.38
Q96EY1	DnaJ homo	10	4	4	480	52.5	9.26	3226507.45	8727478.625
H7BZ37	BCL-6 cor	3	3	3	1659	181	6.64	83862252.08	38455406.63
A0A0B4J25	Lysozyme	25	4	4	137	15.3	8.69	22680538.51	5803207.5
D3DSM4	Collagen,	3	4	4	1336	135.4	6.47	3288982.482	6513989.875
Q9P266	Junctiona	4	5	5	1359	148.3	7.09	7633395.007	6578219.75
P62081	40S ribos	19	4	4	194	22.1	10.1	36918981.4	102666317
Q86SQ0	Pleckstri	2	3	3	1253	142.1	7.43	2363356.883	5523183.5
Q5VWQ0	Lysine-sp	5	4	3	802	90	8.6	14848260.86	8169540.75
Q9NUL7	Probable	6	3	3	540	59.5	10.42	1598700.645	3368066.188
B7Z6F7	cDNA FLJ6	3	4	4	1274	141	6.13	3476027.006	5016196.25
Q12873	Chromodom	2	5	1	2000	226.5	7.3		973767.0625
A0A024R6F	Zinc fing	10	3	3	424	48.1	7.94	1106981.998	3118058.125
Q01469	Fatty aci	27	3	3	135	15.2	7.01	29057594.93	7190158.313
E7EU96	Casein ki	9	3	3	385	45.3	7.94	3607823.828	3391550.75
E9PC52	Histone-b	7	3	3	416	46.9	5.07	3875547.003	7752723.688
B4DZG2	cDNA FLJ5	3	2	2	905	102.9	9.38	433335.4577	1826236.75

B3KPV5	cDNA FLJ3	7	3	3	3	661	76.3	4.77	16573.37695	341780.2414
B4DP31	Phosphori	8	1	1	1	282	31.2	6.95	845348.25	
Q15365	Poly(rC)-	8	1	1	1	356	37.5	7.09	444379.9688	
C9J2N0	Profilin	20	1	1	1	125	13.6	6.06	377572.0625	
H7BZW6	Histone c	11	1	1	1	143	16	9.13	98278.02344	
Q5SSJ5	Heterochr	5	3	3	3	553	61.2	9.67	849733.7188	1431359.199
B7Z6I3	DNA ligas	3	1	1	1	738	82.3	8.57	110757.2344	
E9PMD4	FACT comp	12	1	1	1	173	19.8	5.57	112847.3281	
B4DKJ4	cDNA FLJ5	33	1	1	1	142	16.1	4.68		
B5BU25	Splicing	9	2	2	2	471	53.1	9.09	41383.51563	38142.25862
Q8WYA6	Beta-cate	7	1	1	1	563	65.1	5.05	89888.94531	
F8WEJ5	Asparagin	5	1	1	1	401	45.6	7.09	87964.92969	
Q7Z6E5	P-type Ca	2	1	1	1	1054	115.6	5.41	137369.8438	
Q9BV37	PWP1 homo	8	3	3	3	501	55.8	4.79	149441.1895	398831.311
P26885	Peptidyl-	11	1	1	1	142	15.6	9.13	111451.8203	
Q9Y3C8	Ubiquitin	17	1	1	1	167	19.4	7.4	108909.6094	
J3KT29	60S ribos	15	1	2	1	122	13.2	10.93	322329.0625	
B3KUV5	ubiquitin	10	1	1	1	315	35.3	8.51	240615.875	
Q9HCM4	Band 4.1-	8	3	3	3	733	81.8	6.58	39161.11719	170113.8296
O94874	E3 UFM1-p	3	1	1	1	794	89.5	6.79	75162.07813	
Q8NE01	Metal tra	4	1	1	1	707	76.1	6.09		
Q9UBE0	SUMO-acti	13	2	2	2	346	38.4	5.3	275575.9375	
K7ESA3	Synaptic	18	1	1	1	125	13.6	9.38	327952.3438	
A0A6Q8PFW	Apoptosis	7	1	1	1	293	32	9.74		
B4DRK5	cDNA FLJ5	7	1	1	1	257	28.2	6.76		
Q13098	COP9 sign	6	1	1	1	491	55.5	6.74		
A0A7I2V3Q	Ribosomal	8	3	3	3	634	72.1	4.75	13738.45605	187774.7877
O60547	GDP-manno	6	1	1	1	372	41.9	7.31	193670.7969	
H3BQZ7	HCG204479	6	3	3	3	746	84.6	4.93		303734.6459
A0A8V8TKT	STE20/SPS	6	1	1	1	524	57.2	6.49	80682.26563	
I3L4C3	Sperm-ass	12	1	1	1	194	22.1	7.93	209084.4375	
Q9H4N9	DNA helic	7	1	1	1	351	39.2	7.69	204553.6406	
B4DWW8	U4/U6 sma	5	1	1	1	450	50.1	5.25	150764.6563	
P35520	Cystathio	4	1	1	1	551	60.5	6.65		
A0A8V8TQB	Nuclear p	3	1	1	1	712	80.3	5.86	134449.7813	
Q9Y6M1	Insulin-l	7	1	1	1	599	66.1	8.46	76230.20313	
Q9UIG0	Tyrosine-	2	3	3	3	1483	170.8	8.48	50503.69189	234245.9483
Q15388	Mitochond	14	1	1	1	145	16.3	8.6	1138076.875	
P37108	Signal re	10	1	1	1	136	14.6	10.04	91325.14063	19205.83982
H0Y579	UV excisi	27	1	1	1	114	12.2	5.52		
Q5T6V5	Queuosine	8	1	1	1	341	39	5.88		
A0A8V8TL3	Purine nu	10	1	1	1	210	23.4	6.68		
Q12996	Cleavage	3	1	1	1	717	82.9	8.12	312061.0313	
B4DWC4	Chloride	7	1	1	1	245	27.8	6.38	821093.9375	
A0A1U9X8J	SKIV2L OS	3	1	1	1	1246	137.6	6.1		
E7EN95	Filamin-B	1	1	1	1	2409	256.1	5.73	151000.5625	
Q6FHZ0	Malate de	7	2	2	2	338	35.5	8.68	237155.3711	
Q9UKV3	Apoptotic	3	3	3	3	1341	151.8	6.43	150947.4766	23145.98141
A0A5F9ZHW	Ataxin-2	4	3	4	3	1178	127.1	8.97	157346.4355	288637.9148
B4DE91	cDNA FLJ5	6	1	1	1	484	52.7	6.06	136511.8906	
B4DZ87	cDNA FLJ5	4	1	1	1	501	56.9	5.91	175462.4531	
B4DU42	cDNA FLJ5	4	1	1	1	642	71.8	7.3		
A0A0S2Z4B	Serine/th	5	1	1	1	511	57.6	6.3	151592.375	164738.4197
Q7L2H7	Eukaryoti	14	2	2	2	374	42.5	5.63	146703.6563	
J3QRD0	Cytosolic	22	1	1	1	134	14.9	5.64		
B3KWE1	histidine	8	1	1	1	440	49.6	5.41		
Q7Z478	ATP-depen	3	3	4	2	1369	155.1	8.09	78929.23828	242435.8565
G3V1L9	Tight jun	2	3	3	3	1861	208.2	7.25	252941.8047	149068.2202
B2R5H0	Protein S	15	1	1	1	105	11.7	7.18	61708.51953	
G3V5T3	Cleavage	32	1	1	1	62	7.2	4.77		
Q15369	Elongin-C	27	1	2	1	112	12.5	4.78	104672.4063	
B4DUL5	cDNA FLJ5	7	1	1	1	365	40.3	5.88		
Q12974	Protein t	17	1	1	1	167	19.1	8.37		
B4DQI7	cDNA FLJ5	11	1	1	1	264	31.4	9.22		
Q15459	Splicing	6	2	2	2	793	88.8	5.22	570119.2031	
Q16595	Frataxin,	18	1	1	1	210	23.1	8.69		
A0A024RDQ	Heat shoc	3	1	1	1	814	92.1	5.55		
Q96P63	Serpin B1	6	2	4	2	405	46.2	5.53	233822.9531	161148.1424
Q8TC62	Septin OS	4	1	1	1	417	48.6	8.63	87378.11719	
Q15126	Phosphome	7	1	1	1	192	22	5.73	58343.01563	
Q9NZ90	Carboxype	18	1	1	1	233	26.9	4.78	277205.875	
Q5H924	HECT, UBA	1	1	1	1	3407	374	5.1		
O00505	Importin	8	1	1	1	521	57.8	4.94		
Q15061	WD repeat	4	1	1	1	677	74.8	5.57		
A8KAH1	Phosphati	5	2	2	2	473	55.4	8.38	6299.387695	221974.8552
O95292	Vesicle-a	16	2	2	2	243	27.2	7.3	2299062.578	1688382.547
Q5URX0	beta-N-ac	7	1	1	1	331	38.2	6.44	32718.87695	
Q6ICN0	GRB2 prot	13	1	1	1	217	25.1	6.55		
A0A0K0K1L	Carbonic	12	1	1	1	260	29.2	7.74		
B7Z8Z6	DNA repli	1	1	1	1	954	106.8	5.36	222654.0469	
Q13257	Mitotic s	10	1	1	1	205	23.5	5.08	290852.8125	
B3KTB3	cDNA FLJ3	4	1	1	1	945	103.2	4.81	64856	
Q16540	39S ribos	27	1	1	1	153	17.8	9.69		
B3KV72	cDNA FLJ1	3	1	1	1	707	78.3	8.6		
A0A0S2Z43	Hydroxyst	7	1	1	1	192	19.6	7.21	89068.41406	
O60763	General v	3	1	1	1	962	107.8	4.91	253216.5469	
Q59ES1	Leukotrie	7	1	1	1	219	24.2	6.89	128140.0781	
B2RWN5	HEAT repe	1	1	1	1	2144	242.1	6.55		
Q9H0E9	Bromodoma	4	3	3	3	1235	135.3	4.6	55150.38672	111180.4155
B4DTK7	cDNA FLJ6	5	3	3	3	977	108.9	8.44	40369.50391	563943.8954
O43818	U3 small	9	1	1	1	475	51.8	7.85	165853.3438	
Q9NZ32	Actin-rel	8	1	1	1	417	46.3	7.37		
A0A384DVU	Patatin-l	4	2	2	2	1365	149.8	7.81		228899.8769
Q2TAA2	Isoamyl a	10	1	1	1	248	27.6	5.3	60253.92188	
A0A7I2V5B	NADH dehy	9	1	1	1	361	41.5	7.96		
Q68E01	Integrato	2	1	1	1	1043	118	5.8		
Q9Y5Q9	General t	4	3	3	3	886	101.2	5.07	71872.79443	178752.1006
F8WBC0	Ras-relat	47	1	1	1	32	3.5	9.42		
X6RLR1	Dynactin	19	1	1	1	176	19.9	6.8	70974.97656	
Q6ZR44	thioredox	4	1	1	1	581	63.9	7.99	623605.0625	978396.3845
E9PC15	Acylglyce	7	1	1	1	394	43.8	6.34		
A0A494C10	Protein K	4	2	2	2	709	83.2	5.17	242119.2441	557894.4979
A0A182DWF	thioredox	4	1	1	1	494	53.5	6.52	87660.23438	
B0QY90	Eukaryoti	8	1	1	1	466	55.1	8.95	187336.9688	
Q9UM02	Prolyl en	4	1	1	1	710	80.7	5.86	37879.25	
Q9BQA1	Methyloso	8	1	1	1	342	36.7	5.17	152609.7656	
A0A087WUI	Mannose-1	7	1	1	1	399	43.6	7.17	334338.9375	
D6REC9	THO compl	14	1	1	1	166	18.4	4.97	205425.5781	
P05109	Protein S	24	2	4	2	93	10.8	7.03	225588.8828	323289.6473
B4DN54	cDNA FLJ5	10	1	1	1	259	28.5	8.54	89585.88281	
Q08188	Protein-g	3	2	2	2	693	76.6	5.86	137732.0469	490751.2812
A0A087X2I	Proteasom	18	1	1	1	213	23.1	4.75	151220.25	
H0YJX6	Protein a	16	2	3	2	279	32.3	5.91	33283.70703	52087.30873
A0A8Q3WL2	Ubiquitin	21	1	1	1	121	13.9	8.43	445724.4063	
Q9BWJ5	Splicing	28	1	1	1	86	10.1	6.35		
P61081	NEDD8-con	9	1	1	1	183	20.9	7.69	237760.7813	
Q14683	Structura	2	3	3	3	1233	143.1	7.64	72886.22168	230993.6053
H9KV45	Ubiquitin	17	1	1	1	141	15.9	6.29	253479.7969	
Q53F48	Heterogen	10	2	2	2	346	36.9	7.34	100994.2813	53216.54297
P62314	Small nuc	17	1	1	1	119	13.3	11.56		
Q86VR6	RNA helic	3	2	2	2	745	85.8	9.22	81404.24414	151282.3752
B4DT22	cDNA FLJ5	4	1	1	1	616	70.3	8.35	489580.0625	
P83731	60S ribos	11	2	2	2	157	17.8	11.25	135499.6055	305183.788
Q13835	Plakophil	3	2	3	2	747	82.8	9.13	190262.4531	177150.9465
C9JZR2	Catenin d	4	3	3	3	938	104.8	6.87	46615.66992	209687.3242
B1Q2B0	URCC5 OS=	4	3	3	3	911	101.2	8.43	85110.59961	237937.748
G3V4T2	Polyadeny	16	1	1	1	178	20.2	9.48	73612.64844	
C9JIZ0	U6 snRNA-	32	1	1	1	75	8.1	4.35		
B4DFM5	Vacuolar	5	1	1	1	385	43.2	5.33	69590.22656	
Q7Z7K6	Centromer	7	1	1	1	275	29.9	9.73	177012.3281	
P30044	Peroxired	10	1	1	1	214	22.1	8.7	339691.4375	
A0A0S2Z4Z	Serine/ar	2	2	2	2	918	103.9	11.84		265003.9492
B4DM21	cDNA FLJ5	5	2	2	2	490	55.1	7.72	158815.375	127693.9876
A0A8I5KVV	AFG3-like	4	1	1	1	772	85.7	8.48	380716.125	
P08621	U1 small	4	2	2	2	437	51.5	9.94	784357.8125	621363.9726
Q59FP7	DNA (cyto	2	3	4	3	1606	181.9	7.65	99092.60938	358335.0024
A0A384P5S	Epididymi	1	1	1	1	2452	282.1	5.34	126683.1797	
B4DHH8	cDNA FLJ5	3	1	1	1	858	98.1	6.71		
Q5SRE5	Nucleopor	2	1	1	1	1749	195.9	6.73		
D6R953	Soluble 1	10	1	1	1	222	25.2	4.86		
Q13011	Delta(3,5	7	1	1	1	328	35.8	8	465935.5625	
Q9UGR2	Zinc fing	2	2	2	2	977	109.8	7.25	16314.25977	11339303.41
P31025	Lipocalin	13	2	3	2	176	19.2	5.58	224377.2734	158653.5233
Q05CR9	C6orf130	49	1	1	1	41	4.7	4.08		
B2RDG4	cDNA, FLJ	5	1	1	1	508	57.4	8.76		
B4DXT2	cDNA FLJ5	2	1	1	1	813	91.9	6.09	96742.64063	
Q1LZN2	NOMO3 pro	3	1	1	1	807	89.8	5.67	192814.7031	
U3KQM0	Membrane-	34	1	1	1	91	10.5	4.56	424457.9063	
P46013	Prolifera	1	2	2	2	3256	358.5	9.45	22120.92969	80008.08512
Q05DF2	SF3A2 pro	5	1	1	1	481	51.4	10.11	85280.0625	
Q3B768	KIAA0406	2	1	1	1	803	90.3	6.43		
Q96IN7	PPM1G pro	10	1	1	1	333	36.3	4.28		
B4DP80	NAD(P)H-h	13	1	1	1	307	33.6	8.73		
Q8TCX0	Delta 2-i	27	1	2	1	48	5.5	9.54	455939.1875	658202.0262
P45954	Short/bra	5	1	1	1	432	47.5	6.99		
F5H4Z7	Corrinoid	16	1	1	1	173	18.8	8.16		
Q9NWK9	Box C/D s	8	3	3	3	470	53.9	5.8	19070.95703	298699.0251
Q9BVJ6	U3 small	4	2	2	2	771	87.9	7.87	179920.3398	123907.7925
D6RER5	Septin OS	9	1	1	1	432	49.8	6.68	497854.9063	
Q99961	Endophili	10	1	1	1	368	41.5	5.43	52169.90234	
A0A7P0T9L	Cullin-4B	3	1	1	1	780	90.3	7.99		
B1AMT4	Cohesin s	8	1	1	1	548	62.5	5.88		
A8K5K5	Eukaryoti	8	1	1	1	320	35.6	6.13		
Q8NDV7	Trinucleo	2	2	3	2	1962	210.2	7.01	97218.94922	176375.4732
P68402	Platelet-	9	1	1	1	229	25.6	5.92	147925.9219	
Q8WUF5	RelA-asso	3	1	1	1	828	89	6.81		
B4DNJ5	Dolichyl-	5	1	1	1	378	43.3	6.48	221192.4063	
A0A7S5BY4	IGH c312_	11	1	1	1	123	13.9	8.81	93681.20313	
A0A0S2Z4X	Epididymi	3	2	2	2	852	94.3	6.73		98296.28757
B2R6K4	cDNA, FLJ	5	1	1	1	340	37.3	6.18		
B4DE78	cDNA FLJ5	13	1	1	1	207	23.5	4.82		
B3KM43	cDNA FLJ1	4	1	1	1	621	68.8	6.13		
O96028	Histone-l	2	2	2	2	1365	152.2	8.69	48818.46289	132326.6329
A0A087WYF	Branched-	9	1	1	1	225	25.3	6.04	144060.8125	

A8K9F5	cDNA FLJ7	5	2	2	567	63.6	9.98	876501.6789	3658087.063
P23490	Loricrin	7	3	3	312	25.7	8.09	49472939.12	14589613.75
Q5QJE6	Deoxynucl	5	4	4	756	84.4	6.16	602015.3996	5181400.875
Q5SRE5	Nucleopor	3	5	5	1749	195.9	6.73	1253610.368	4358684.438
K7EQJ5	40S ribos	9	2	2	141	16.6	10.46	13452591.36	16190237.25
Q5QNW6	Histone H	28	3	3	126	13.9	10.32	14864635.78	6823907.281
P08047	Transcrip	4	1	1	785	80.6	7.34	1364624.66	2636355.5
Q9H4L7	SWI/SNF-r	3	3	3	1026	117.3	5.55	676147.0537	2655598.625
P33993	DNA repli	5	3	3	719	81.3	6.46	1928111.432	3180606.688
B3KVY9	cDNA FLJ4	8	3	3	412	45	8.98	2045660.701	9463301.25
Q92616	eIF-2-alp	2	5	5	2671	292.5	7.43	1282065.429	3549690.656
Q12872	Splicing	4	4	4	951	104.8	8.05	3303360.104	4751647.938
Q96HS1	Serine/th	11	3	3	289	32	8.68	8606091.749	9025173.625
O60885	Bromodoma	3	4	4	1362	152.1	9.19	4542266.697	8996712.25
Q6ZVX7	F-box onl	15	2	2	275	30.8	6.62		
B4DWS6	cDNA FLJ6	12	3	3	304	33.5	9.39	3051483.149	5954599.75
P54136	Arginine-	7	4	4	660	75.3	6.68	886300.4461	3621243.656
H7C2W9	60S ribos	29	3	3	108	12.8	11	19532520.8	13789830.5
C9JH19	Cathepsin	8	4	4	451	48.6	7.84	13788153.77	4837541.313
Q6P432	Peptidyl-	17	4	4	247	27.5	9.5	8335850.002	7921507
C9J384	Protein C	19	4	4	225	26.2	9.88	5669353.73	8183465.75
Q15365	Poly(rC)-	10	3	3	356	37.5	7.09	9580107.422	5007479.75
Q9BU76	Multiple	13	3	3	263	29.4	10.02	6227004.763	6790914.375
A0A8V8TLC	SKI2 subu	4	2	2	1033	114	6.11	693738.6959	1251213.688
Q8N684	Cleavage	8	4	4	471	52	8	3385988.28	4638787.563
Q9NY93	Probable	6	3	3	547	61.6	9.26		3565873.875
Q92820	Gamma-glu	9	3	3	318	35.9	7.11	3414253.748	770227.625
Q9HC07	Transmemb	11	2	2	324	34.9	7.02	1286933.186	4346404.313
Q9BXW9	Fanconi a	3	4	4	1451	164	5.88	2712930.206	5070284.25
B3KNC3	Nucleolar	4	3	3	749	84.9	5.62	1110810.547	8012866.5
P04792	Heat shoc	18	4	4	205	22.8	6.4	11759707.84	2850639.219
Q0P607	Splicing	2	3	3	1146	125.7	9.55	1405196.521	5919968.75
Q9Y295	Developme	6	2	2	367	40.5	8.9	718078.977	3691109.5
B2R8F3	cDNA, FLJ	7	2	2	319	35.2	9.11	2273347.934	5977747.25
D3DQS4	Formin bi	3	3	3	1017	110.2	4.75	948775.252	2725943.125
Q8N516	Elongator	2	3	3	1332	150.1	6.01	543400.8221	1223814.156
A0A8Q3SHW	Nucleolar	9	5	5	598	70	8.68	392306.3467	6204194
A0A494C1P	Actin-bin	5	2	2	426	48	7.97	5056996.593	4718155
O14647	Chromodom	2	4	4	1828	211.2	8.1	10723323.07	8541682
Q5VYS8	Terminal	2	3	3	1495	171.1	6.83	301941.5873	2297756.25
Q9NWQ8	Phosphopr	8	2	2	432	47	4.65	755322.7995	1851541.5
B7ZKK7	eIF2AK2 p	6	3	3	546	61.5	8.29	390070.5613	3359592
Q9C075	Keratin,	11	5	3	422	48.1	6.54	3449448.176	555146.2031
Q58F28	PPP1R10 p	11	3	3	312	34.7	9.88	3319574.999	3299857.938
Q96SN8	CDK5 regu	2	4	4	1893	214.9	5.58	1650950.72	4245814.5
A0A087WWH	BRD4-inte	2	2	2	1508	152.7	7.46	230225.5865	1561592.063
Q9BZ93	Prosomal	43	2	2	58	6.2	8.97	2094975.904	1052940.344
Q5VWG9	Transcrip	4	3	3	929	103.5	9.06	3372170.163	2858994.875
Q9BRK4	Leucine z	5	4	3	669	72.7	6.51	11573977.65	6109557.875
Q86UK7	E3 ubiqui	6	5	5	904	98.6	8.4	4181878.532	8284977.688
P24928	DNA-direc	2	4	4	1970	217	7.37	1244871.647	6380207.125
Q8WYP5	Protein E	2	3	3	2266	252.3	6.6		2918750.188
Q14146	Unhealthy	2	3	3	1524	170.4	7.31		2465319.5
A0A087X12	Probable	28	2	2	115	13	9.94	363465.3373	3219190.625
H0YJQ1	Neuroguid	11	3	3	316	35.8	9.09	623814.3826	4923828.75
Q9HC36	rRNA meth	12	4	4	420	47	8.73	12706081.06	9875252.375
Q9BQA1	Methyloso	6	2	2	342	36.7	5.17	1484708.326	1270561.438
A0A590UJS	Disks lar	4	3	3	839	93.5	5.92	6352042.98	5237307
O95782	AP-2 comp	4	4	4	977	107.5	7.03	10479483.73	15823882.75
A2VCQ4	PRKCSH pr	17	3	3	181	20.3	4.72	1475885.137	268964.3438
A0A5C2G36	IGL c2395	21	2	2	113	12.4	4.83	21970082.42	536239.0625
Q9Y3U8	60S ribos	29	3	3	105	12.2	11.59	8017266.56	8384716.25
E9PLX0	Calpain-1	23	2	2	122	13.6	7.77	1678570.615	510158.25
P42766	60S ribos	21	3	3	123	14.5	11.05	128208527.4	69278218
P00441	Superoxid	17	2	2	154	15.9	6.13	4126551.707	772202.25
K7ER00	Phenylala	7	4	4	548	62.4	7.84	4314582.001	12984008.13
P35249	Replicati	9	3	3	363	39.7	8.02	331743.7241	2341158.938
A0A8I5KVV	AFG3-like	4	4	4	772	85.7	8.48	3056204.56	4710819.313
Q9H2G2	STE20-lik	2	3	2	1235	142.6	5.15	1119349.273	1642827.625
B4DTV0	cDNA FLJ5	4	2	2	1079	114.1	6.43	3193246.45	2484852.188
Q5VTL8	Pre-mRNA-	6	3	3	546	64.4	10.54	6428241.574	6269420
P37198	Nuclear p	7	2	2	522	53.2	5.31	735851.529	1773810.438
P49207	60S ribos	28	4	4	117	13.3	11.47	38958641.96	43398207.5
B7Z284	DNA-direc	4	4	4	1207	136.9	6.86	314959.6904	3230010.625
Q8N163	Cell cycl	3	3	3	923	102.8	5.22	1491302.555	2010592.875
A0A5C2G1B	IGL c2340	18	2	1	113	12.3	5.05	36863344.85	1172245.625
Q96FQ6	Protein S	22	2	2	103	11.8	6.79	10045435.87	4214181.625
Q5TB53	Transmemb	11	3	3	253	29.9	5.82	963312.1453	4505954.125
A0A5C2G37	IGL c3728	27	3	1	113	12.5	6.57	87554963.31	
B4DF70	cDNA FLJ6	23	4	4	183	20.1	8.78	16068088.2	6107098.375
A8KA19	Exportin-	4	3	3	962	109.8	5.39	4380192.883	2852635.563
O94776	Metastasi	4	3	3	668	75	9.66	9846838.787	16849272.94
P35610	Sterol O-	6	3	3	550	64.7	8.94	1237909.891	5678526
A0A0A0MT0	Gelsolin	4	3	3	767	84.7	5.83	2654209.991	4709659.875
Q01081	Splicing	9	2	2	240	27.9	8.81	4101970.932	6546341.125
Q9NX20	39S ribos	11	2	2	251	28.4	10.13	2291699.953	2682142.625
P10599	Thioredox	21	2	2	105	11.7	4.92	33665945.98	11820380.5
P82914	28S ribos	11	3	3	257	29.8	10.48	4401592.025	8182265.375
P56270	Myc-assoc	9	4	3	477	48.6	8.95	10764374.62	10706340
H0Y3N9	Histone 1	2	2	2	1049	116.8	8.5	2830134.205	6800842.75
Q13242	Serine/ar	15	3	3	221	25.5	8.65	2523716.366	4469465.25
Q9UMS4	Pre-mRNA-	4	2	2	504	55.1	6.61	1019258.421	3805107.313
Q9UI42	Carboxype	7	3	3	421	47.3	6.7	6620931.883	1187308.313
Q86W92	Liprin-be	2	3	3	1011	114	5.55	4252336.031	4768978.375
F8VZX2	Poly(rC)-	12	3	3	321	33.8	8.24	4015389.015	6739912
Q96JM3	Chromosom	3	2	2	812	89	8.44	303648.5503	2004039.25
P04075	Fructose-	11	4	4	364	39.4	8.09	6653932.141	2516170.625
A0A024R3H	Chromosom	8	2	2	293	34.2	9.73	2686120.17	1402125.75
Q9BQ67	Glutamate	5	2	2	446	49.4	4.92	629042.4494	3200307.375
Q53FV0	Prohibiti	12	3	3	272	29.8	6.39	3746699.398	8940193.25
P18583	Protein S	1	2	2	2426	263.7	5.64	1611655.389	4414018.625
Q5VWU8	Partition	3	3	3	1001	109.9	6.6	447315.8961	2879434.75
J3QLR8	28S ribos	18	3	3	152	17.5	9.63	3271845.991	5687929.125
P61026	Ras-relat	15	3	3	200	22.5	8.38	12054311.71	5956284
Q4JLR8	Hemoglobi	35	3	1	105	11.4	6.68		933790.75
A4D2N6	Ubiquitin	2	3	3	1350	148.1	8.57	902607.6155	2230628.188
Q9NQV6	PR domain	2	2	2	1147	130.1	6.74	662846.4952	2918441.25
D3DQA6	RNA helic	3	3	3	883	100.3	9.33	2894040.177	4244593
B2R9Y2	Nuclear s	8	4	4	558	66.3	8.97	5356249.071	2645561.813
A8KAK1	cDNA FLJ7	2	2	2	1531	174.9	5.52	1255120.28	2823541.375
Q701L7	Type II h	5	3	2	513	56.6	6.74	2651313.261	311787.1875
A0A087WUV	Basigin C	19	3	3	189	20.5	6.68	2703208.825	3376411.25
E9PQB7	Cofilin-1	25	3	3	122	13.9	8.72	7007489.676	6834440.125
P62266	40S ribos	15	2	2	143	15.8	10.49	13457091.07	13989760.5
Q16513	Serine/th	4	4	4	984	112	6.3	2964776.931	3037162.438
Q7Z2K8	G protein	4	3	3	1008	102.3	8.06	3430761.85	3079579.5
F8VV59	Nucleosom	5	1	1	327	38	4.68		
O60382	KIAA0324	2	2	2	1791	191.2	11.93	2342442.321	4889158
Q9NXF1	Testis-ex	3	2	2	929	105.6	9.36	176438.7671	1087218.75
Q53GD7	FUS inter	8	2	2	262	31.3	11.27	1049678.318	952870.0625
O75175	CCR4-NOT	5	3	3	753	81.8	6.2	1757861.965	2343610.625
A8K5U9	cDNA FLJ7	6	4	4	652	70.7	7.62	5275731.081	5815627.875
Q15746	Myosin li	2	3	3	1914	210.6	6.15	708020.2983	2743243.313
Q8IY67	Ribonucle	7	2	2	606	63.8	8.48	1043991.37	1672983.563
P25490	Transcrip	9	3	3	414	44.7	6.25	1721409.579	6149110.75
J3KQN4	60S ribos	18	3	3	142	16.4	10.43	35434391.4	23074310
D3DU86	TNF recep	5	3	3	570	63.9	6.95	2672127.259	5494632.563
Q7Z5L9	Interfero	5	1	1	587	61	8.69	286192.73	478668.0625
A0A5C2G3N	IGL c913_	17	1	1	107	11.6	8.53	1757392.726	
Q5CZ94	Uncharact	10	2	2	234	25	7.27	14971034.93	415232.1563
Q9H2H9	Sodium-cc	5	3	3	487	54	7.02	5077634.287	6698349.625
P40222	Alpha-tax	5	3	3	546	61.9	6.52	2029132.124	5528573.25
B4E063	Kinesin-1	4	3	3	665	73	8.98	1883749.058	3212974
F5GY37	Prohibiti	9	2	2	267	29.7	9.88	1365814.155	988667.1563
Q16610	Extracell	4	2	2	540	60.6	6.71	3629837.831	1022643.375
A0A8I5QL0	Histone-1	1	2	2	1738	184.6	9.33		710382.4375
P23258	Tubulin g	6	3	3	451	51.1	6.14	1136941.443	4120635.688
A0A8V8TQT	Fatty acy	6	2	2	390	43.7	7.23		2726987.375
A8K3H8	cDNA FLJ7	5	2	2	589	65.3	5.11	1661655.969	4392721
Q8NC60	Nitric ox	4	2	2	698	78.4	8.66		2961360.875
B3KSG9	Transmemb	5	2	2	573	65.4	7.28	1175765.135	2373463.875
P82675	28S ribos	5	2	2	430	48	9.92	801424.7823	3485697.5
B3KNK4	Phosphati	4	1	1	445	51.3	7.09		1091049.625
P02765	Alpha-2-H	5	2	2	367	39.3	5.72	25422441.64	9787107.5
P28289	Tropomodu	6	2	2	359	40.5	5.1	4508315.278	2907845.625
B4DJL0	Dolichyl-	8	2	2	283	31.3	5.78	1730149.387	2029854.875
E9PB90	hexokinas	3	2	2	889	98.9	6.04	1756328.799	1472781.188
D3DUE6	Cytokine-	4	2	2	558	60.9	9.42	282861.1579	1859959.688
A8K883	cDNA FLJ7	8	3	1	524	59.2	5.02		
P36873	Serine/th	9	3	3	323	37	6.54	3825729.401	4403648.313
B4DJ49	cDNA FLJ6	6	2	1	443	50.4	8.92	3504031.431	6269270
Q9UGM3	Deleted i	7	1	1	2413	260.6	5.44	1955634.356	1043947.313
A0A5C2G6P	IGL c84_1	17	1	1	107	11.8	8.44	1406605.023	
Q9H0E9	Bromodoma	2	2	2	1235	135.3	4.6	1483109.628	2494732.875
Q5VT52	Regulatio	3	2	2	1461	155.9	7.42		
E9PHA2	Condensin	4	2	2	730	81.5	5.06	599270.9953	1530473.938
Q59EK7	CSODF038Y	11	3	2	326	37.2	10.96	679749.9073	1312827.125
O60610	Protein d	2	3	3	1272	141.3	5.41	8027899.179	10919146.53
A0A024R25	Melanoma	3	2	1	778	86.1	5.83	344657.8807	626718.9375
B4DR61	Protein t	7	3	3	482	52.9	8.24	5601137.482	10433858
A0A024R1S	GTP-bindi	4	2	2	660	71.4	8.22	1134924.664	2433446.75
O75487	Glypican-	4	3	3	556	62.4	6.68	1085717.001	3253189.375
Q53G19	Mitochond	10	2	2	192	20.6	9.91	2128510.511	3378453.125
Q2TTR7	Receptor	2	2	2	1210	134.2	6.68	1862088.63	2691767
Q53HQ0	Flotillin	7	3	3	427	47.3	8.09	3012175.557	3109651.969
A0A3B3IRR	UPF0488 p	7	1	1	226	24.5	9.88	857680.8439	1792899.375
B3KPV5	cDNA FLJ3	3	2	2	661	76.3	4.77	3497209.015	5468266.5
A0A8V8TP0	Erbin OS=	2	3	3	1343	150.4	5.31	2281077.269	3416782.938
A8K594	cDNA FLJ7	4	3	3	963	107.4	7.59	2508167.61	3264290.563
A0A087WVZ	Protein k	2	2	2	1136	125.7	6.8	2608743.314	3236383.75
F8WAM2	T-complex	22	2	2	98	10.5	7.42	3052661.411	2323349.875
P42357	Histidine	3	2	2	657	72.7	6.95	5126823.498	589756.625
Q04721	Neurogeni	1	3	2	2471	265.2	5.14	1682369.222	2365757.313
O14976	Cyclin-G-	2	2	2	1311	143.1	5.73	1050659.437	2045358.938

Q59H77	T-complex	4	1	1	1	577	63.5	6.43		
F8WCX2	Zinc fing	3	2	2	2	785	80.8	6.84	33688.46484	184564.4801
Q9NXG9	cDNA FLJ2	5	1	1	1	416	47.5	6.14		
O75607	Nucleopla	22	1	1	1	178	19.3	4.63	53684.19922	
Q99757	Thioredox	15	1	1	1	166	18.4	8.29	162103.9063	
A0A024R7I	GIPC PDZ	9	1	1	1	236	26.1	5.57		
Q86YP4	Transcrip	5	2	2	2	633	68	9.94	54323.92383	98884.59499
P50748	Kinetocho	1	1	1	1	2209	250.6	5.97		
Q9H2G2	STE20-lik	2	1	1	1	1235	142.6	5.15		
Q2F834	Myosin re	14	1	1	1	142	16.4	4.55	157138.2188	
M0R2K3	BRISC and	12	1	1	1	200	21.8	4.53	183375.5469	
Q9NQZ2	Something	5	2	2	2	479	54.5	5.62		77224.12457
Q5JC46	Transform	7	1	1	1	439	48.7	5.35		
Q6PD74	Alpha- an	7	1	1	1	315	34.6	4.64	185530.4688	
Q9NX24	H/ACA rib	21	1	1	1	153	17.2	8.22	319453.5625	
A0JLU5	ESF1 prot	5	2	2	2	552	63.4	4.73		149874.0354
Q9Y520	Protein F	1	2	2	2	2896	316.7	9.13	69020.0625	224603.9118
E9PFH4	Transport	3	1	1	1	857	96.6	5.66	58216.14844	
A0A6Q8PG3	Kinesin-1	3	2	3	2	717	81	6.1	54219.86426	177762.1239
Q9UQ88	Cyclin-de	2	2	2	2	783	91.3	5.36		125809.3912
O00232	26S prote	5	1	1	1	456	52.9	7.65	278990.9063	
Q8N1G2	Cap-speci	3	1	1	1	835	95.3	7.05		
B7ZB80	cDNA, FLJ	11	1	1	1	322	35.5	5.39	58718.66406	
Q13423	NAD(P) tr	1	1	1	1	1086	113.8	8.09	286648.4688	336716.8338
A0A1W2PP7	glycine c	5	1	3	1	420	46.2	7.59	766899.9277	1493155.859
P43487	Ran-speci	11	1	1	1	201	23.3	5.29	155447.7656	
Q8IWC1	MAP7 doma	2	2	2	2	876	98.4	9.32	22528.75977	293471.447
P61326	Protein m	12	1	1	1	146	17.2	6.11	46017.73438	87756.3291
Q6P158	Putative	1	2	2	1	1386	155.5	7.71	48529.51563	54185.38436
A0A8V8TL8	Rho-assoc	2	1	1	1	1445	167.1	5.88		
Q5UGI6	Serine/cy	5	1	1	1	333	37.3	8		
I3L4P8	Heme oxyg	15	1	1	1	130	15.1	4.88	72130.1875	
Q05D78	Double-st	5	1	1	1	517	59.7	6.92	365824.5313	
Q0VDC5	peptidylp	81	1	1	1	42	4.5	6.51	460548.0938	
B5MCH7	eIF5-mimi	7	1	1	1	343	39.4	6.35		
Q5JR04	RNA helic	2	2	3	2	947	107.1	8.79	107884.8438	78703.61371
E9PDW2	Succinyl-	10	1	1	1	334	36.7	6.74	116676.4531	
Q71UP1	Cytochrom	81	1	1	1	37	4.2	4.81		
F5GZS6	4F2 cell-	3	1	1	1	599	64.8	5.1	213205.0781	
G3V547	Ribosome	18	1	1	1	121	14	9.5	117694.4688	
H0YBE0	Protein L	37	2	2	1	60	6.4	8.66	99655.85938	176338.4852
J3KT04	RNA helic	45	1	1	1	85	9.3	4.72	128605.625	
P13798	Acylamino	3	1	1	1	732	81.2	5.48	129709.9844	
A0A679KTH	beta-gala	2	2	3	2	1279	145.4	5.81	142840.0938	1316789.315
P27695	DNA-(apur	7	1	1	1	318	35.5	8.12	154018.1094	
Q9Y490	Talin-1 O	1	1	1	1	2541	269.6	6.07		
M1V490	KIF5B-RET	2	1	1	1	1173	134.1	6.04		
Q8NDD9	Uncharact	11	1	1	1	332	37.4	5.9		
Q15050	Ribosome	6	1	1	1	365	41.2	10.7	74281.46875	
A0A0B4J25	Lysozyme	9	1	1	1	137	15.3	8.69	252320.25	309029.3186
P31151	Protein S	11	1	2	1	101	11.5	6.77	165328.3125	230498.6744
A4UJ43	Glutathio	8	1	1	1	210	24.8	5.24	169461.75	
J3QKW7	Developme	13	1	1	1	244	26.6	9.5	159633.6406	
Q5JWE9	Guanine n	2	1	1	1	1022	109.5	5.07	151096.3281	
I3L2R6	Thioredox	19	1	1	1	98	11.3	5.21	231989.4531	
P25787	Proteasom	9	1	1	1	234	25.9	7.43		
Q9NRG9	Aladin OS	4	1	1	1	546	59.5	7.5		
Q7L0Y3	tRNA meth	5	2	2	2	403	47.3	9.36	25060.1123	92193.49024
A0A2R3Z0D	Hemoglobi	19	1	1	1	68	7.4	8.54	38784.44141	118729.0841
Q68E00	Uncharact	6	1	1	1	315	32.6	6.77	130288.1406	
A0A5C2G7I	IGH c572	16	1	1	1	118	12.7	5.4	299584.6563	
H7BXF3	Transform	11	1	1	1	127	15.2	10.2	209934.7188	468000.1896
O60264	SWI/SNF-r	2	2	3	2	1052	121.8	8.09	43287.71094	105843.326
I6L8B7	Fatty aci	20	2	2	2	101	11.3	6.07	242677.7813	283320.9468
A0A7S5EUY	IGH c347_	11	1	2	1	126	14.1	8.4	205927.0156	717041.6301
B1AHD3	Chronophi	42	1	1	1	79	8.8	4.41	74309.72656	
Q3B726	DNA-direc	10	1	1	1	338	37.4	6.98		
J3QRM1	CDK5 regu	6	1	1	1	272	30.2	4.87		
Q9NUG6	p53 and D	13	1	1	1	133	15.5	6.06	62783.36328	
P48594	Serpin B4	4	2	2	2	390	44.8	6.21	123673.6758	115005.8454
M0R2A0	ER membra	7	1	1	1	371	39	6.9	87759.75781	
U6FSN9	Tyrosine-	2	2	2	2	1331	150.4	6.29	72669.5918	32173.28355
A0A140T94	Pre-mRNA-	4	1	1	1	669	76.9	6.09	364571.5313	
O94887	FERM, ARH	2	1	1	1	1054	119.8	8.79		67464.14864
B4DPT6	Sugar pho	7	1	1	1	322	37.3	5.85		
A8K8N3	cDNA FLJ7	2	2	2	2	1193	134.3	5		131920.6374
J3KP30	Deoxynucl	2	1	1	1	601	66.1	5.11	8818.727539	60333.98955
Q9NRL2	Bromodoma	1	1	1	1	1556	178.6	6.6		137140.2734
Q59EF0	Delta isc	4	1	1	1	503	59.3	7.4		
A0A024R75	Calumenin	9	1	1	1	315	37.1	4.59		
P22392	Nucleosid	10	1	1	1	152	17.3	8.41	774957.5625	289532.963
Q92522	Histone H	6	1	2	1	213	22.5	10.76	44154.28516	180162.3319
Q9H857	5'-nucleo	6	1	1	1	520	60.7	6.77		
O14556	Glycerald	3	1	2	1	408	44.5	8.19	1154915.875	1910281.276
B3KNE7	(human) h	6	1	1	1	418	47.9	8.9	79349.70313	
B4DSB9	DNA misma	5	1	1	1	720	81.3	6.57		
F5GY05	Constitut	2	2	2	2	933	106	5.87	93680.24609	185398.9963
A0A8V8TPX	Nuclear p	1	1	1	1	1661	179.6	6.6	300709.75	
Q5JSZ5	Protein F	1	1	1	1	2229	242.8	8.34		150305.132
P49207	60S ribos	8	1	1	1	117	13.3	11.47	141098.4531	313321.3074
P09012	U1 small	6	1	1	1	282	31.3	9.83	46366.625	
Q59FZ5	PTPRF int	2	1	1	1	696	77.7	8.92	10403.80078	50815.15937
Q7L2M7	PFKL prot	2	1	1	1	568	62.6	8	80498.59375	
P62277	40S ribos	10	1	1	1	151	17.2	10.54	154851.2813	
P05089	Arginase-	7	2	3	2	322	34.7	7.21	115484.3984	107042.5025
B5MCF9	Pescadill	3	2	2	2	571	66	7.5	915029.4902	1486270.33
Q9HAU5	Regulator	2	2	3	2	1272	147.7	5.69	38342.08984	74184.40386
Q71MF7	Uncharact	5	2	2	2	545	60.7	10.04	31243.9375	33077.54744
P36405	ADP-ribos	16	1	1	1	182	20.4	7.24	48857.08203	
Q9H2G4	Testis-sp	2	1	1	1	693	79.4	4.58		48131.31047
B4DN99	cDNA FLJ5	7	1	1	1	370	42.6	6.64		
A0A8Q3SI9	Myosin li	21	1	1	1	105	11.2	8.09	67023.47656	
O94906	Pre-mRNA-	1	1	1	1	941	106.9	8.25	54789.96094	117491.4409
Q59GK9	60S ribos	9	1	1	1	163	18.9	10.54	35915.24219	100237.4084
O75475	PC4 and S	3	1	1	1	530	60.1	9.13	72112.96875	
H0Y938	Coatomer	3	1	1	1	898	101.5	5.82		
E9PCX4	Slit homo	9	1	1	1	146	16.7	9.25	13425.04004	40855.21358
E5RI99	60S ribos	11	1	2	1	114	12.6	9.55	176727.7656	80071.19307
J3KQN4	60S ribos	12	2	2	2	142	16.4	10.43	194260.375	604938.8916
A0A494C05	NF-kappa-	6	2	2	2	414	47	10.14	132488.5625	132061.0393
B7Z977	cDNA FLJ5	6	2	2	2	515	58.1	9.01	17535.43164	59985.04614
P25208	Nuclear t	10	1	1	1	207	22.8	4.59	226777.0938	
P57772	Selenocys	3	1	1	1	596	65.3	8.35	23819.27344	
B8ZZQ4	Pentatric	9	1	1	1	187	21.1	9.72	71161.33594	150312.8232
A0A087X2D	Serine/ar	22	1	1	1	95	10.3	5.14	241771.4531	
B2R791	U4/U6 sma	3	2	2	2	683	77.5	9.5	103487.2109	52540.94091
Q9NR28	Diablo IA	12	1	1	1	239	27.1	5.9		
Q5TB53	Transmemb	10	1	1	1	253	29.9	5.82	152537.125	
M0QX76	40S ribos	20	1	2	1	50	5.6	9.63	183411.7344	271827.0592
Q5H909	Melanoma-	4	2	2	2	588	63.1	9.35	16130.7998	106396.6544
Q9Y6A4	Cilia- an	16	1	1	1	193	22.8	9.76	134715.9219	
B7ZKN7	DNA helic	2	2	2	2	1042	117	8.63	37514.57031	182264.2375
M0QZR9	ELAV-like	8	1	1	1	153	17.1	8.66	40263.54297	55948.31589
Q9P206	Uncharact	3	2	2	2	1035	107	9.73	16674.49609	260233.6034
A0A8I5KW5	Endoplasm	8	1	1	1	381	44.1	5.35	383081.9063	
Q7Z4Q2	HEAT repe	3	1	1	1	680	74.5	5.11	19804.27344	
Q9Y266	Nuclear m	11	1	1	1	331	38.2	5.38		
I3L443	rRNA meth	6	1	1	1	177	19.7	9.66		82952.53257
Q03701	CCAAT/enh	1	1	1	1	1054	120.9	5.94		112277.265
C9JLV6	Malate de	23	1	1	1	114	12.4	5.01		
Q9UFN0	Protein N	13	1	1	1	247	28.4	9.16	82040.96875	
A0A7P0TAE	A-kinase	2	1	1	1	612	68.2	5.06		75138.10832
B5BU01	Eukaryoti	3	1	1	1	333	38.3	5.94	34942.74609	70599.75395
V9GZ54	Moesin (F	17	1	1	1	103	12.1	5.25		
X5D299	Succinate	2	1	2	1	548	58.6	8.09	111295.3047	215737.9757
D9HTE9	Plasma me	4	1	1	1	318	35	9.99	58362.40625	193118.8475
Q00688	Peptidyl-	17	2	2	2	224	25.2	9.28	1839249.621	
A0A7S5EUS	IGH c1305	11	1	1	1	124	13.4	5.4	44115.68359	
O14992	Heat shoc	4	1	1	1	474	52.3	8.21	142987.3594	
A0A5C2FX8	IGL c1591	12	1	1	1	111	12.2	8.88	108837.9609	
C9JLU1	DNA-direc	14	1	1	1	148	16.9	4.68	49132.22266	
P41091	Eukaryoti	5	1	1	1	472	51.1	8.4		
Q59FA2	Serine/ar	10	1	1	1	233	25.6	9.01	196014.1563	
P09417	Dihydropt	11	1	1	1	244	25.8	7.37		
Q9NQX3	Gephyrin	5	1	1	1	736	79.7	5.43	88950.66406	
Q9Y5M8	Signal re	6	1	1	1	271	29.7	9.04	14785.68652	
F8VRN8	LIM domai	3	1	1	1	388	43.2	8.51		
A0A5C2GJ5	IG c360_l	17	1	1	1	108	11.7	6.51	21902.63086	
J3KRX5	60S ribos	6	1	1	1	174	20.2	10.1	83392.04688	368739.7266
A0A8I5KYH	Magnesium	3	1	1	1	305	34.6	9.58	57037.67188	218903.301
O76003	Glutaredo	7	1	1	1	335	37.4	5.39	73136.99219	
H7C4A9	cAMP-depe	17	1	1	1	99	11.6	9.79	86323.11719	
A0A669KBF	Carbamoyl	2	1	2	1	660	73.3	5.41	631327.5625	2843721.055
Q8IVT2	Mitotic i	2	1	1	1	679	75.3	6.83	40219.68359	123818.6534
P18754	Regulator	7	1	1	1	421	44.9	7.52	547322.125	
B7Z8F4	RNA helic	1	1	1	1	1181	133.9	7.88		60873.14509
A0A7I2V5Y	Kinesin-1	2	1	1	1	974	111.6	8.5		43109.71626
P07305	Histone H	5	1	1	1	194	20.9	10.84	248190.6875	216075.6383
A6QKW0	SHINC3 OS	5	1	1	1	247	27.9	9.7	47077.625	96917.28628
B4E392	cDNA FLJ5	4	1	1	1	437	49.1	5.5	508555.1875	135148.4175
B4DR61	Protein t	2	1	1	1	482	52.9	8.24	55090.91406	147096.0523
A0A2R8Y84	40S ribos	9	1	1	1	131	15.2	10.9	74021.13281	
Q6NUR7	Ezrin OS=	3	1	1	1	586	69.2	6.27	72014.4375	
Q641R2	Oxysterol	2	1	1	1	728	82.5	7.08		79319.6347
A0A1U9X8X	Corneodes	3	1	1	1	528	51.5	8.35	37674.47656	66767.50538
I0B0K8	Truncated	0	1	1	1	4021	430.2	9.29	73923.24219	109188.7164
A0A0J9YXT	MICOS com	14	1	2	1	49	5.2	10.21	202500.6094	209742.2498
P60866	40S ribos	17	1	1	1	119	13.4	9.94	280606.2813	
B7Z8S9	mRNA (gua	9	1	1	1	129	15	7.85	37858.16406	37408.61951
H0YN88	40S ribos	16	1	1	1	136	15.9	9.91	122261.3438	112940.0919
P13473	Lysosome-	4	1	1	1	410	44.9	5.63	112739.0234	39943.53145
Q8N5C6	S1 RNA-bi	1	1	1	1	995	111.7	8.72	21563.32031	40976.29067
Q9NUJ1	Palmitoyl	7	1	1	1	306	33.9	8.57		
Q86W92	Liprin-be	1	1	1	1	1011	114	5.55		47801.93481

J3KPH2	Arachidon	3	3	3	867	97.7	8.18	5090045.773	2718038.875
A0A087X0L	Mitochond	6	3	3	435	48.5	9.45	708785.0991	5139679.375
P63104	14-3-3 pr	11	2	2	245	27.7	4.79	2872381.32	692025.6875
Q9BQ04	RNA-bindi	7	3	3	359	40.1	6.74	1740574.114	4155823.813
B7Z2A9	V-type pr	2	2	2	794	91.1	6.58	1498495.035	1224613.875
O15260	Surfeit l	8	2	2	269	30.4	7.78	378938.9871	1680246.125
E9PLD3	Ribonucle	13	1	1	99	11.6	12.23	2728718.776	3045105.75
A0A1W2PNX	Protein u	2	2	2	1084	118.3	6.71	1264980.065	1089327.094
Q9Y2R9	28S ribos	10	2	2	242	28.1	9.99	1623233.704	2182480.5
Q6IPN0	Reticulon	6	2	2	343	36.9	4.81	1809199.322	5502558.25
Q5VY60	HAUS augm	2	2	2	802	91.1	6.43	1163248.81	1546948.25
Q6NXF8	PRDM15 pr	2	2	2	1141	129.5	7.96		2404472.125
A8K7J3	cDNA FLJ7	3	2	2	807	92.6	5.29	1775717.565	1174113.125
A0A087WYT	Prostagla	13	2	2	164	19.1	4.55	1856030.018	4085562.25
P59665	Neutrophi	19	2	2	94	10.2	6.99	10173745.8	4494140.5
B7ZLC9	GEMIN5 pr	2	3	3	1507	168.3	6.62	1975696.811	3970028.125
B2RBE0	cDNA, FLJ	3	2	2	720	80.3	8.46	851983.5986	2712064.625
O75635	Serpin B7	6	2	2	380	42.9	6.8	1848426.342	
B4DMV0	Brain-spe	4	3	3	500	54.8	9.17	2110282.974	2335843.688
O75683	Surfeit l	7	1	1	361	41.4	10.64		1515094.25
Q1RMG2	Adenosylh	7	2	2	306	33.8	6.61	2277505.551	1194087.844
E5RG18	Double-st	19	1	1	69	7.8	9.31		1211041.625
A0A3B3ISG	RICIN dom	4	2	2	567	64.2	9.28	866715.7918	2294244.375
A0A087WX5	Hepatoma-	22	1	1	68	7.2	4.22	1825403.617	1104997.375
P49848	Transcrip	3	2	2	677	72.6	8.6	1426134.095	1317626.063
F8WCX2	Zinc fing	3	2	2	785	80.8	6.84	1286107.201	2417238.75
Q4ZG57	DNA repli	4	3	3	785	88.9	5.6	3674506.125	1681086.188
Q7Z589	BRCA2-int	2	2	2	1322	141.4	9.33	202565.5263	1606087.938
Q96N66	Lysophosp	5	2	2	472	52.7	8.97	259415.8826	2803672.25
P17900	Gangliosi	9	2	2	193	20.8	5.31	3477840.422	1236967.25
B2R5H0	Protein S	24	3	3	105	11.7	7.18	9406781.719	2932255.438
A0A5C2FWS	IGL c365_	17	1	1	107	11.8	8.47	923590.3379	
A0A024RC4	Cadherin-	3	2	2	906	99.7	4.81	2105301.516	3606347.875
Q6AHZ7	Uncharact	2	3	3	2109	238.7	7.36		3139170.625
Q14202	Zinc fing	2	2	1	1370	152.3	6.35		
A4UCR9	Mitochond	13	2	2	176	20.8	10.93	1575378.342	4653224.625
Q86XZ4	Spermatog	6	2	2	545	59.5	8.9	286658.949	1168190.25
F5H423	ADP-ribos	10	2	2	210	23.3	8.6	5473287.305	4339291.625
H7C0P8	DNA repai	16	2	1	158	18.5	8.73	1299693.205	1108651.25
Q53H03	Nuclear a	3	3	3	788	85.2	4.3	3168341.757	5244318
Q9GZZ8	Extracell	14	2	2	138	14.2	5.5	8739646.36	2880231.25
P11047	Laminin s	1	2	2	1609	177.5	5.12		3062391.875
P13010	X-ray rep	3	2	2	732	82.7	5.81	5314220.207	22487382
A0A7P0T8Y	Dystrobre	3	2	2	617	69.2	6.49	1749915.042	1527312.688
Q6P1A2	Lysophosp	4	2	2	487	56	8.69	813187.5249	3127898.875
F5GX80	Zinc fing	10	2	2	211	23.5	5.5	4174408.217	1064578.25
B2R8G6	peptidylp	4	1	1	356	38.5	7.69		572819.4375
P84243	Histone H	29	2	1	136	15.3	11.27		
R4GMU7	60S ribos	9	2	2	198	23	10.64	1279932.926	1851180.125
A0A2U3TZV	Phosphoin	2	2	2	1685	188.6	7.84		1898151.438
A0MZ66	Shootin-1	3	2	2	631	71.6	5.33	8825162.72	4856862.375
H3BRS3	Telomere	7	3	3	426	47.4	8.38	2983609.967	1908579.938
K7ELG9	Protein L	6	1	1	230	24.9	7.42	1846398.269	1788906.75
Q5U0A0	Proteasom	8	1	1	241	26.4	4.79		
A0A494C1T	Protein F	2	2	2	1380	149	6.71	1343159.35	1758784.625
A0A7S5EUY	IGH c347	11	1	1	126	14.1	8.4	22712170.61	28228686.5
E7ETZ8	Microtubu	3	2	2	623	71.4	8.56	1518165.441	1817487.813
B7ZB05	cDNA, FLJ	2	1	1	776	87.9	9.11	229297.4067	796711.8125
Q59EK1	Adducin 3	4	3	3	698	78.2	6.15	5064167.51	1584469.813
B3KWP5	cDNA FLJ4	16	2	2	111	13.1	10.26	8404606.759	3201320.875
A0A1U9X8X	Corneodes	8	2	2	528	51.5	8.35	5528715.056	2065236.75
Q6PJJ2	RRP1 prot	5	2	2	460	52.7	9.33	3680573.465	7163388.75
X5D2F4	Cytoplasm	2	2	2	1253	145.1	6.9	3307958.422	2267088.5
G9BAV9	Lens epit	2	2	2	1121	131.2	6.58	1867839.267	1755222
Q9ULX3	RNA-bindi	4	2	2	412	46.6	7.18	2253880.529	4884998
P50748	Kinetocho	1	3	3	2209	250.6	5.97	1150926.049	2673720.938
Q9UGL9	Cysteine-	21	1	1	99	9.7	8.66	1210719.734	
Q9P1Y5	Calmoduli	2	1	1	1249	134.7	8.35	570235.9243	963406.1875
A0A024R68	Numb homo	4	2	2	603	65.9	8.66	968243.4421	1882551.938
Q5SZE1	Ceramide	5	1	1	304	36.4	9.25	607458.2448	1244021.125
A0A7P0TB2	Neurogeni	1	2	1	2517	268.3	5.14	1055527.949	
Q8N5C6	S1 RNA-bi	2	2	2	995	111.7	8.72	536375.4055	1898059.438
A0A5C2G56	IGL c3566	17	2	1	113	12.6	6.57	3922962.419	
B4DI06	2',3'-cyc	6	2	2	298	32.9	9.54	1474325.124	2322378.594
E9PC15	Acylglyce	6	2	2	394	43.8	6.34	1431250.031	3130259.875
P31025	Lipocalin	11	2	2	176	19.2	5.58	9046174.789	2617522.25
C9JXA5	Guanine n	7	2	1	251	27.5	6.76		2759547.25
O75477	Erlin-1 (	3	1	1	348	39.1	7.87		613965.4375
Q5UGI6	Serine/cy	5	1	1	333	37.3	8	4682435.347	
Q5T760	Serine/ar	4	2	2	491	54.3	10.45	2058413.019	4277255
B4DDI8	cDNA FLJ5	8	1	1	169	18.3	3.92	671741.3781	
P39019	40S ribos	13	2	2	145	16.1	10.32	1843998.077	1237392.5
Q75MH1	40S ribos	13	1	1	115	13	10.98	1410432.862	395219.8438
Q86WV6	Stimulato	7	2	2	379	42.2	7.05	7151636.317	1214391.25
V9GYF0	Rho guani	7	2	1	263	29.5	5.06	2670730.293	3731332.5
J3QSH4	Vascular	4	2	1	512	55.8	9.57	168088.5765	957183.75
Q9H176	ZNF143 pr	6	2	2	531	57.2	6.54	2326670.301	3295390.25
Q59H77	T-complex	4	2	2	577	63.5	6.43	910408.584	2716937.375
B4DYP4	Poly [ADP	2	2	2	1090	118.7	6.95	1350602.274	1464307.563
E9PKN0	Pre-mRNA	3	2	2	784	88.9	9.85	240695.3702	3035617.063
P22314	Ubiquitin	3	2	2	1058	117.8	5.76	621529.6138	1222617.438
I3L1U8	Active br	10	2	1	165	18.9	6.42	495564.2136	676333.3125
B4DUI5	Triosepho	12	2	2	213	22.9	6.92	1177723.946	644615.8125
P17302	Gap junct	4	1	1	382	43	8.76	2186619.006	1990740.75
Q569I8	DNA polym	4	1	1	267	29.4	9.61		1060620.625
A0A0D9SGE	PHD finge	7	2	2	366	41.3	8.68	1628993.945	5392187.375
A4FVA6	MTCH1 pro	6	2	2	370	39.7	9.48	1432796.031	635860.3125
A0A0S2Z47	creatine	5	2	2	405	44.9	6.11	3080204.508	3457517.75
B3KW34	cDNA FLJ4	4	1	1	257	28	4.36	2757088.04	5750394
M0QXT1	FERM, ARH	5	1	1	248	28.2	6.39		728949.75
B3KUJ0	cDNA FLJ3	3	1	1	424	44.5	8.24	1694023.85	1911375.375
J3QRU1	Tyrosine-	3	2	1	548	61.3	6.57	779695.4392	748388.5625
A8K6H6	Tyrosine-	1	1	1	1187	135.2	8.25		1398839.25
B4DLE7	cDNA FLJ5	7	2	2	329	38.7	8.16	347163.1357	1690630.188
Q0VF96	Cingulin-	2	2	2	1302	149	5.67	398013.0746	425098.4375
B2RCT6	cDNA, FLJ	4	2	2	499	55.7	5.62	3030763.796	1205608.375
P11279	Lysosome-	4	2	2	417	44.9	8.75	2631516.199	5331984.406
Q15629	Transloca	5	2	2	374	43	9.63	2931060.343	5491897
P09012	U1 small	6	2	2	282	31.3	9.83	5257508.557	1784442.875
F1T0L5	ATP-depen	3	2	2	666	72.4	8.16	377745.8676	1294760.625
M1VE83	Tyrosine-	3	2	1	643	71.6	4.81	1321951.944	2165872.063
H7C4H9	Polyhomeo	10	1	1	223	24.2	4.98		
Q15648	Mediator	1	2	2	1581	168.4	8.73		1341048.969
Q9NUQ3	Gamma-tax	3	2	2	528	60.5	7.52	1045907.283	1924550.125
B4E324	cDNA FLJ6	3	1	1	480	54.2	6.98	793927.8316	531828.0625
Q6PJG2	Mitotic d	3	1	1	1045	114.9	9.19		1384028.75
Q96I25	Splicing	5	2	1	401	44.9	5.97	697061.5555	872063.8125
H7C224	Interleuk	4	2	1	392	42.2	6.61	772577.3103	875047.625
U3KQS2	Glucose-6	3	1	1	451	48.9	7.99		742644.875
Q59HE6	RNA bindi	2	1	1	505	56.1	5.49	209601.9506	416794.125
Q9NWH9	SAFB-like	2	2	2	1034	117.1	7.87	4751871.109	5705251.5
G3V5V3	Ribosome	1	1	1	998	113.9	5.85	250798.6534	898490.625
Q86UL3	Glycerol-	5	2	2	456	52	9.19	267126.0716	1896051.125
Q53F85	Death dom	3	2	2	740	81.2	4.88	1631947.813	4807495.25
Q59GX7	Stearoyl-	4	1	1	366	42.3	9.25	2704223.647	4182470
P60866	40S ribos	9	1	1	119	13.4	9.94	1168047.199	1295159.625
Q8NEX9	Short-cha	6	2	2	313	35.2	9.16	1905457.986	1391405.719
H0YL69	Proteasom	7	2	2	236	26.4	7.71	2700482.42	1535418.531
C9JJU7	Ribosome	10	2	2	203	22.2	10.89	457241.0329	1401089.625
Q8N5W9	Refilin-B	6	1	1	214	22.9	5.44	960546.9637	2136634.5
Q9UMY1	Nucleolar	7	2	2	257	29.4	9.67	3664922.937	3672512.25
O75330	Hyalurona	3	2	2	724	84	5.83	806992.4116	1398774.813
H0Y8X0	Protein D	8	2	2	285	32.7	6.81	1076396.518	873067.5
Q05C82	MARCKS pr	40	1	1	47	4.6	7.25	2054284.816	1333405.75
A0A8I5QKX	cytochrom	4	1	1	273	31.3	6.6	1387428.741	1095961.5
Q8NDT2	Putative	3	2	2	890	97.1	9.85	2165993.392	2881994.375
B4DEB0	cDNA FLJ5	5	2	2	509	58.5	7.68		
Q9ULE4	Protein F	1	2	2	1060	121	6.18	111946823.4	337382932
A0A5C2GMT	IG c1332_	11	1	1	123	13.7	8.81	1440698.621	
A0A8Q3SII	RANBP2-li	1	2	2	1643	184.7	6.37	455086.5444	769792.6875
H0Y3V8	Sphingosi	11	1	1	135	15	7.77	420821.7457	1091065.375
Q9ULA6	SNW domai	10	2	2	287	33.4	9.31	3174268.035	3104805.25
Q8WVC0	RNA polym	2	1	1	666	75.4	4.51	615357.9353	913142.625
A0A6Q8PHK	Ribose-ph	16	2	2	121	13.1	7.77	2764186.69	4710978.625
A0A804HIC	Transcrip	2	2	2	1907	216.4	5.02		2189595.5
A0A075B6R	Probable	2	1	1	567	64.2	6.43		904684.3125
A0A5C2G2X	IGL c693_	25	2	2	108	11.7	7.96	21321862.29	645376
H0Y3M2	BUB3-inte	7	1	1	198	21.3	5.02	4415692.627	6709755.5
Q1ED39	Lysine-ri	2	1	1	458	51.6	9.86	878397.781	1050930
E7ERK3	Voltage-d	5	1	1	309	34.6	4.74		
A0A7S5EUX	IGH c2806	10	1	1	122	13.6	7.85	1751915.695	
Q5T093	Protein F	9	1	1	117	13.4	10.4		1854109.125
Q9H2G4	Testis-sp	2	2	2	693	79.4	4.58	507969.6002	1751554.813
A0A024RC6	Protein r	3	2	1	620	71.6	6.57	1539933.379	1892491.688
K7EL20	Eukaryoti	5	1	1	262	29.3	5.41	1392125.358	1412012
F5GX64	DNA-bindi	8	2	2	240	26.6	8.25		1252901.875
A0A0S2Z5H	Clathrin	2	1	1	643	70.3	6.58	2162844.073	2378809.25
A0A0U1RQV	non-speci	2	2	2	1137	133	5.35	608120.0353	699859.5
O60299	Leucine z	3	2	2	673	71.7	7.64	1894381.254	2228876.313
Q68DW4	MICOS com	4	1	1	268	29.2	9.69	960705.3296	1297931
P27482	Calmoduli	8	1	1	149	16.9	4.42		
B7Z8Z6	DNA repli	1	1	1	954	106.8	5.36	1019309.277	392003.5
D6W5Y5	Cold-indu	4	1	1	297	31.9	9.61	2526761.345	2055906.125
P07199	Major cen	2	1	1	599	65.1	4.55		1828342.75
B7Z6K5	cDNA FLJ5	6	1	1	155	17.5	6.58	2342680.534	1663034.625
Q9HAU0	Pleckstri	1	1	1	1116	127.4	7.53	626886.3301	1120650.75
Q8WUA4	General t	2	2	2	911	100.6	7.31	283904.4278	2720211.438
P11498	Pyruvate	1	1	1	1178	129.6	6.84	10707902.87	
Q29RF7	Sister ch	1	2	1	1337	150.7	7.91		
A0A5C2G3V	IGL c983_	25	2	2	107	11.8	8.44	1364818.057	
E9PM05	Etoposide	10	2	2	187	21.6	9.73	1094496.462	2664977.75
Q59FC9	Enigma ho	5	2	2	436	48.9	7.87	1543330.212	1888390.5
Q71US4	eIF-2B-de	2	1	1	520	57.5	9.32	431738.9039	1106751
A0A5C2G1W	IGL c2904	14	2	1	113	12.4	7.94	99780722.2	

B0AZV0	cDNA, FLJ	12	1	1	1	256	28.5	4.91	60650.33203	
Q9C0J8	pre-mRNA	1	1	2	1	1336	145.8	9.17	126299.6641	279335.6831
Q71UM5	40S ribos	10	1	1	1	84	9.5	9.45		193098.5952
B7ZLW8	Methionin	1	1	1	1	1265	140.5	5.55	1579539.5	2992865.414
Q5T4U5	Medium-ch	4	1	1	1	454	50.2	7.8	54042.69922	
Q53F85	Death dom	2	1	2	1	740	81.2	4.88	31286.53516	131915.543
M0R1H5	40S ribos	23	1	2	1	79	9.5	10.65	47044.51563	30145.36575
Q9BQQ5	Ribosomal	9	1	1	1	106	12	11.46	189640.7813	402274.2424
P61513	60S ribos	20	1	1	1	92	10.3	10.43	39396.17188	22269.87392
E5RHD2	Poly [ADP	3	1	2	1	243	23.6	8.43	47123.78125	139241.1549
Q1ZYL5	Tropomyos	4	1	1	1	245	28.5	4.78	48287.21875	136149.4085
Q6R327	Rapamycin	1	1	1	1	1708	192.1	7.47	21145.25977	
J3QRN6	Unconvent	1	1	1	1	961	111.2	9.39	104937.5547	25124.92105
Q01081	Splicing	5	1	1	1	240	27.9	8.81	30125.58008	81013.77344
A8K503	cDNA FLJ7	22	1	1	1	158	18	8.87	66879.72656	
F5H116	Piwi-like	3	1	1	1	203	23.2	8.82	102292.6953	180254.2089
B4DX42	Splicing	2	1	1	1	433	46.4	9.35	106339.2578	136841.8195
P47929	Galectin-	8	1	1	1	136	15.1	7.62	64757.48438	43398.1355
S4R3N9	Siderofle	19	1	1	1	177	19.6	9.38		
Q96EY1	DnaJ homo	3	1	1	1	480	52.5	9.26	24457.57031	119079.1718
D6RBQ6	Ubiquitin	2	1	1	1	530	59.6	7.99	2124658.25	1378959.909
Q14203	Dynactin	2	1	1	1	1278	141.6	5.81	67815.96875	
Q5T7C4	High mobi	9	1	1	1	158	18.3	9.7	208323.1719	
P18583	Protein S	1	1	1	1	2426	263.7	5.64	26270.90234	44679.83621
Q5EBM5	PB1 prote	2	1	1	1	425	46.8	6.8		100727.0881
H0YKD8	60S ribos	6	1	2	1	170	19.1	11.46	64573.58203	160788.7228
O76094	Signal re	3	1	1	1	671	74.6	9.26	13774.5625	73902.25205
Q6PJJ2	RRP1 prot	2	1	1	1	460	52.7	9.33		222718.5787
H0YM74	Zinc fing	2	1	1	1	858	91.9	7.97		82722.89757
B4DL07	cDNA FLJ5	2	1	1	1	683	78.4	9.11	14057.55762	53158.38312
Q502W6	von Wille	1	1	1	1	1294	145.7	7.33	1185943.625	730113.9115
Q16094	Calnexin	4	1	2	1	272	31.2	4.79	1015144.125	285300.247
Q59GX7	Stearoyl-	4	1	1	1	366	42.3	9.25		55923.92543
P78413	Iroquois-	2	1	1	1	519	54.4	6.18	470253.8438	509994.6198
P15531	Nucleosid	25	1	1	1	152	17.1	6.19	1563379.375	
Q9HCD5	Nuclear r	4	1	1	1	579	65.5	9.6		
Q9H072	Uncharact	2	1	2	1	533	58.1	8.62	115046.4531	124588.05
Q53G98	Activatin	2	1	1	1	481	54.6	5	16967.02344	42606.67093
A0A8V8TMN	Coiled-co	2	1	1	1	608	67.2	7.81	29454.26953	108189.7736
F8WEF5	PHD and F	1	1	1	1	1647	178.3	8.98	21094.32422	48787.75233
Q9Y547	Intraflag	19	1	1	1	144	16.3	5.03	280267.5938	
F5H303	Nucleolar	3	1	1	1	270	31.1	9.55		51216.43672
O76071	Probable	10	1	1	1	339	37.8	4.97	106885.7969	
B4DM74	60S ribos	8	1	1	1	154	18.1	10.48	90389.77344	139978.9923
Q6PJM8	RRP1B pro	3	1	1	1	408	46.9	8.94	59132.79688	
O60362	Uncharact	6	1	1	1	242	27.3	8.88	77769.28125	80592.83952
O60271	C-Jun-ami	2	1	1	1	1321	146.1	5.15	81447.42188	135320.1225
A0A7S5BZ5	IGH c139	6	1	1	1	126	14	8.88	67502.60156	87488.27443
Q53HB9	RNA helic	4	1	1	1	547	61.5	9.26		
Q9H089	Large sub	1	1	1	1	658	75.2	6.38	80576.57813	78063.27017
B4DT66	Protein S	2	1	1	1	650	75.5	9.22	30308.11328	85855.73361
B7Z357	cDNA FLJ5	4	1	1	1	233	25.9	8.09	46568.75	
F6T1Q0	2',5'-pho	4	1	1	1	472	52.1	7.84	76159.09375	76381.27442
A0A087X29	WD repeat	1	1	1	1	1151	124.9	6.92	22816.29102	79702.67146
P40222	Alpha-tax	2	1	1	1	546	61.9	6.52	30271.91211	80146.67913
Q8TF01	Arginine/	2	1	1	1	805	92.5	10.02	54537.51953	
D3DX49	MAGUK p55	1	1	2	1	576	64.5	6.73	1480835.875	1226635.218
H7C1P0	Replicati	12	1	1	1	111	11.8	5.22	28433.40234	38857.39328
Q9UF51	Uncharact	1	1	2	1	877	94.9	8.65	672167.1875	809339.3806
A0A8I5KXL	Cold shoc	1	1	1	1	599	67.2	5.87	34252.91797	41372.43965
Q9Y3U8	60S ribos	10	1	1	1	105	12.2	11.59	58151.60938	172641.1679
A0A8I5KYP	Electron	6	1	1	1	327	34.5	8.47	21823.47656	53793.21947
A8K594	cDNA FLJ7	3	1	1	1	963	107.4	7.59	30105.50586	
Q8IY67	Ribonucle	4	1	1	1	606	63.8	8.48	38672.63281	34367.31561
Q9Y6B5	non-speci	4	1	1	1	540	60.5	5.57		
A0A7I2YQF	Eukaryoti	4	1	1	1	362	40.5	5.64	198113.7188	
B4DHZ9	cDNA FLJ5	11	1	1	1	295	32.4	6.98	223105.7656	
P11498	Pyruvate	1	1	1	1	1178	129.6	6.84	486867.5	591040.616
B4DQZ0	cDNA FLJ5	12	1	1	1	214	24.2	5.44		
B4DKK9	cDNA FLJ5	8	1	1	1	180	20	7.43	58333.44922	
P57077	MAP3K7 C-	6	1	1	1	142	16.4	4.89	622069.5625	595691.2303
E5RI60	A disinte	2	1	1	1	705	77.4	7.68		37391.58067
H0Y3N9	Histone l	1	1	1	1	1049	116.8	8.5	11811.54199	56179.32507
A0A7P0TBI	Anoctamin	1	1	1	1	771	89.9	8.62	76392.05469	32737.08426
E5RFK8	Leucine z	4	1	1	1	273	32.1	5.44	60396.08594	68358.4288
S4STV3	NADH-ubiq	5	1	2	1	603	67	9.1	38061.92188	
Q15182	Small nuc	3	1	1	1	285	29.7	10.07	32299.87305	65687.7731
Q8NAR1	cDNA FLJ3	19	1	1	1	161	16.6	7.37	46020.17969	251006.1367
Q5T750	Skin-spec	5	1	1	1	250	26.2	7.97	83416.74219	95420.27238
A0A024R4X	Protein k	2	1	1	1	435	50	6.43	29732.92969	38900.92304
A0A7S5C3L	IGH c2479	7	1	1	1	123	13.6	8.84	186938.6406	349233.8614
A0A3B3ITF	Male-spec	8	1	1	1	102	11.5	10.08	48429.13281	114307.7653
A0A6I8PS1	Guanine n	3	1	1	1	516	58	7.05	77932.76563	262392.676
A0A804HIF	Potassium	1	1	1	1	540	59.6	8.73		
Q9UK61	Protein T	1	1	1	1	1670	188.9	5.8		29852.66721
Q58F28	PPP1R10 p	3	1	1	1	312	34.7	9.88	32946.8125	117579.002
B7Z3A4	cDNA FLJ5	1	1	1	1	1349	148.8	9.06		90523.22469
A0A7T0PYL	Immunglob	7	1	1	1	122	12.9	5.29	997308.5	1674156.746
P23490	Loricrin	3	1	1	1	312	25.7	8.09	216597.9688	
A0A2R8Y88	Girdin (F	1	1	2	1	1113	127.2	8.21	1991151.375	1781892.422
H0YB97	Acyl-CoA	14	1	2	1	175	19.8	7.68		
K7EIH0	Ubiquitin	9	1	1	1	101	12	9.88	118375.8359	107721.3807
A0A8V8TKI	Integrin	1	1	1	1	1021	113.2	5.68	1023215.188	2120241.682
L8E8M5	Alternati	14	1	1	1	49	5.3	10.29	215012.7656	306117.1776
Q08J23	RNA cytos	1	1	1	1	767	86.4	6.77		159661.4845
A0A8V8TN5	Non-recep	3	1	1	1	210	24.2	6.14	145561.3906	298554.2551
A0A0J9YXY	T cell re	25	1	1	1	114	12.5	8.16		90730.96157
Q8TCJ2	Dolichyl-	1	1	1	1	826	93.6	8.91		
Q9UGM3	Deleted i	7	1	1	1	2413	260.6	5.44	310606.9688	
Q9NVF9	Ethanolam	2	1	1	1	386	44.8	7.33	197825.9688	704826.0034
E9PDF6	Unconvent	2	1	1	1	1107	128.4	9.31		
Q9Y230	RuvB-like	6	1	1	1	463	51.1	5.64	82650.61719	
F5GXG4	Serine/th	7	1	1	1	140	15.9	7.02	16165.39844	57432.05297
F4MH47	Ubiquitou	1	1	1	1	1377	152.6	7.85	401605.9375	636329.0746
J3KT73	60S ribos	20	1	1	1	64	7.6	9.99	38137.61328	
Q6IMI6	Sulfotran	3	1	1	1	304	35.9	6.92	265441.25	236822.1772
C9J8D0	HMG box t	5	1	1	1	448	50.6	5.74		49011.08593
Q9H6F5	Coiled-co	2	1	1	1	360	40.2	10.33	59487.47656	66837.27525
K7ELQ6	Uncharact	23	1	1	1	150	15.1	11.96		

A0A0S2Z3I	Solute ca	7	2	2	259	28.2	9.58	3051343.024	3906207.25
O60362	Uncharact	7	2	2	242	27.3	8.88		4056274.375
B2RTU6	HCG198101	1	1	1	1609	180.4	6.55		597474.875
Q9NR12	PDZ and L	2	1	1	457	49.8	8.41	679718.3187	1063682.25
Q6Y1H2	Very-long	5	1	1	254	28.4	9.55	975921.4989	1530080.625
D3DWB6	ubiquitin	0	1	1	2379	271.1	5.97		2255363
P0DMU9	Cancer/te	9	2	2	189	21.1	9.55		1778927.5
A0A024R1X	Protein (	2	1	1	741	80.4	7.17	2137333.277	
Q9NXE8	Pre-mRNA-	3	1	1	425	49.6	10.18	1185606.845	
P14174	Macrophag	18	1	1	115	12.5	7.88	1804832.604	696134.625
A0A1B0GUE	Acid cera	3	1	1	330	37.4	6.79	1202206.511	503768.3438
B3KXB8	cDNA FLJ4	2	1	1	929	99.4	8.82		
A0A2R8Y69	IQ motif	3	2	2	779	87.8	7.01	998657.0874	2661250.375
A0A024QZN	Vinculin	2	2	2	1066	116.6	6.09	1873624.574	429018.8125
Q59GX2	Solute ca	4	2	2	517	57	9.47	1413220.996	3234338.25
A4D1M6	Elongatio	6	1	1	225	24.9	4.82	669235.6701	
F5H5T2	dolichyl-	19	1	1	77	9	5.14		1017436.313
Q9NW36	cDNA FLJ1	4	1	1	310	35.1	6.39		1636886
Q96QD8	Sodium-cc	4	1	1	506	56	8		
O60725	Protein-S	6	1	1	284	31.9	7.96		1476459
Q12899	Tripartit	4	1	1	539	62.1	5.03		1235197.625
Q8TBT6	Cytochrom	4	1	1	315	34.5	8.87	373149.2711	1403284.25
H0Y368	Dolichol-	7	2	2	295	33.3	9.14	2230270.244	3024653.75
B4DY51	cDNA FLJ5	3	1	1	402	46.5	9.38	498797.6979	2155093.25
P41743	Protein k	2	1	1	596	68.2	5.85	427487.185	659242.3125
A0A8I5KXL	Cold shoc	3	2	2	599	67.2	5.87	581024.916	1493965.625
A0A059QB8	ATP synth	4	2	2	226	24.7	10.1	1430201.99	2458071.875
Q15043	Metal cat	3	1	1	492	54.2	5.33	1079411.116	1047675.813
B3KNE4	cDNA FLJ1	1	1	1	818	91.2	5.38	1115216.298	2808326.5
B4E1S3	cDNA FLJ5	5	1	1	234	25	9.54	2361741.696	1223104
F8W084	Cleavage	12	1	1	107	11.6	4.21	2937896.771	3541341.75
P20618	Proteasom	10	2	2	241	26.5	8.13	1786880.969	473569.4375
H0Y9X1	Translati	6	2	2	242	27.7	9.57	1519805.633	2649899.375
D6RIY6	Exosome c	3	1	1	423	46.9	5.16		1024257.75
Q9UQR0	Sex comb	4	2	2	700	77.2	8.54	468007.2845	1914189.75
L0EQ92	Tumor sup	19	2	1	107	11.9	8.62	7221595.133	9884929
A0A5C2G8J	IGL c3955	13	1	1	110	11.6	8.97	2333913.033	1346540
B3KY94	CDP-diacy	10	2	2	237	25.9	8.22	8406853.598	3085846.5
Q9H1I8	Activatin	3	2	2	757	86.3	5.16	879809.0612	1065149.125
H3BMM9	RNA-bindi	5	1	1	284	31.7	11.62	444370.5447	476681.1875
Q13585	Melatonin	2	1	1	617	67.3	7.72	2943743.866	4081123.5
A0A8I5KYH	Magnesium	6	2	2	305	34.6	9.58	4055376.699	9480913.25
B7Z361	Reticulon	5	1	1	205	22.9	8.84	2173794.391	
A0A7S5BY4	IGH c312_	11	1	1	123	13.9	8.81	3730492.204	
Q8N0Z3	Spindle a	1	1	1	855	96.2	7.44	473378.3456	566456.125
Q05DF2	SF3A2 pro	4	1	1	481	51.4	10.11	827679.3304	1033499.938
Q659F8	Uncharact	6	1	1	225	23.9	9.03	897395.5243	2634078.5
Q8NCN4	E3 ubiqui	3	2	2	708	77.1	9.1	532226.6063	754408.1875
A0A2R8Y6L	dual-spec	3	2	2	605	68.4	8.95	1351505.177	687838.5
A0A8Q3WKT	Complemen	4	2	2	391	42.8	7.84	2608187.976	1650968.75
F5H1T9	Keratin-a	8	1	1	124	13	7.81	2093071.286	
A0A1B1PEN	Cytochrom	5	1	1	261	30	7.28	4462812.935	4676355.5
Q99460	26S prote	2	1	1	953	105.8	5.39		596758.9375
Q15182	Small nuc	5	2	2	285	29.7	10.07	4662876.256	9422756.5
Q9UNX4	WD repeat	2	2	2	943	106	6.64	893217.6431	1876957.188
A0A5C2GFT	IG c260 h	9	1	1	127	13.8	7.94	1515473.886	
Q02224	Centromer	0	1	1	2701	316.2	5.64	1052111.788	713620.6875
Q6DD87	Zinc fing	3	1	1	382	40.4	7.96	1607465.368	1197738.625
Q8IZP0	Abl inter	2	1	1	508	55	7.06	710229.2364	982037.9375
Q96ST3	Paired am	2	2	2	1273	145.1	7.25	1100512.794	1551691.188
Q9BV37	PWP1 homo	3	2	2	501	55.8	4.79	406804.1619	7179626.25
E9PFH4	Transport	1	1	1	857	96.6	5.66	545596.0195	744306.3125
F5H5N1	NADH dehy	5	1	1	182	19.8	9.5	1199973.806	1144526.625
D3DUR9	Zinc fing	2	1	1	576	63.1	8.95		1216505
O00264	Membrane-	7	1	1	195	21.7	4.7		
Q59FD4	hexokinas	3	2	2	949	105.7	6.84	378407.7487	739448.5
A0A087WUD	Oligosacc	14	1	1	83	9.4	7.43		1021033.188
C9J8R7	GTP-bindi	5	1	1	210	22.5	9.83		1164090.625
B3KRJ9	cDNA FLJ3	2	1	1	514	58.2	9.92	1033426.67	1007554.375
B2RDX0	cDNA, FLJ	2	1	1	672	75.6	4.75	618783.2162	465507.6563
B4DH43	cDNA FLJ5	2	1	1	830	82.7	8.56		938017.75
A0A5C2G07	IGL c2621	14	1	1	108	11.9	9.2	2954115.804	
A0A024R75	Calumenin	3	1	1	315	37.1	4.59	1110264.073	
E7EN89	Toll inte	4	1	1	246	27	5.64	1870461.908	2085699.25
A0A5C2GMY	IG c604_l	12	1	1	112	12.4	8.47	27853086.14	703017.875
P15531	Nucleosid	13	2	2	152	17.1	6.19	2340339.594	1103222.094
Q5JTC6	APC membr	1	1	1	1135	124	4.84		561226.6875
Q7KZN9	Cytochrom	3	1	1	410	46	9.82	472359.809	1354601.625
I3L2C6	Mitochond	4	1	1	214	23.7	4.98	1341802.097	1558554.5
Q9UEK9	Keratin (	70	1	1	23	2.5	4.45	1482253.897	
Q53R85	Aspartyl-	5	1	1	188	21.3	8.41	235413.3521	452810.3438
B4E1E0	CTP synth	2	1	1	435	49.7	7.52	1111285.539	1915863.875
A0A6Q8PGU	vesicle-f	2	2	2	916	100.3	5.86	935628.0161	
A0A3S8NFS	ATIII-T1	2	1	1	414	47.1	6.04	1685615.239	
P38919	Eukaryoti	3	1	1	411	46.8	6.73	635893.6153	754725.9375
O76031	ATP-depen	2	1	1	633	69.2	7.58	307901.3865	568560.9375
Q92734	Protein T	3	1	1	400	43.4	5.1	4267580.558	1393882.625
Q9UPN7	Serine/th	2	2	2	881	96.7	4.55		4781124.875
Q9NZH8	Interleuk	7	1	1	169	18.7	5.14	1137210.551	
A0A8V8TL1	Phosphati	2	1	1	699	80.8	6.25		
P62861	FAU ubiqu	8	1	1	133	14.4	10.17	19150078.95	15706575
A0A7P0T9L	Cullin-4E	1	1	1	780	90.3	7.99	436219.7157	
I3L374	TLC domai	24	1	1	38	3.7	6.39	913815.8057	1755091.25
A0A7T0M69	NADH-ubiq	3	1	1	347	39	9.83		1051877.625
A0A0J9YYL	Poly(U)-b	5	2	2	505	54.6	5.34	694373.1409	879201.4375
O75044	SLIT-ROBO	1	1	1	1071	120.8	6.7	1201526.42	
A8K201	RRP15-lik	4	1	1	259	28.9	5.38	170488.5562	950751.8125
A0A024R7K	Splicing	1	1	1	1082	120.2	7.28		533428.625
Q9Y5I4	Protocadh	1	2	1	1007	109.4	5.41	26922624.46	8682139
K7EMH1	60S ribos	15	1	1	89	10.4	9.66	3046238.593	6836553.5
A0A8I5QJG	Rab3 GTPa	2	2	2	885	99	6.19	418280.2816	818803.8906
O60287	Nucleolar	0	1	1	2271	254.2	6.47		585611.375
Q8WTT2	Nucleolar	2	1	1	800	92.5	9.17		1441570.375
E5RK27	Voltage-c	12	1	1	108	11.8	7.94	1200848.503	1731694.75
K7EL56	RNA helic	9	1	1	108	11.9	4.5	977736.3041	937054.625
Q6NTF9	Rhomboid	2	1	1	364	39.2	9.32	1082025.18	1766595
P49755	Transmemb	4	1	1	219	25	7.44	1010430.581	1066885.75
A8K9U1	cDNA FLJ7	1	1	1	1698	191	5.96		352441.3438
P78371	T-complex	2	1	1	535	57.5	6.46	1211271.659	1779410.5
Q96JB2	Conserved	1	1	1	828	94	5.57	621184.1926	830451.1875
J3KPG5	Bromodoma	1	1	1	1875	207.9	6.64		2058507.625
Q5JSD2	Voltage-c	7	1	1	194	21.2	7.93	613225.9756	
Q5VUR7	Putative	2	2	2	823	94	7.94	3934166.895	4119412.125
Q8NDV7	Trinucleo	1	1	1	1962	210.2	7.01	435041.6657	1594493.75
E9PPH5	Acidic le	11	1	1	114	13.1	4.22	581540.1789	670734.9375
A0A499FIX	Extended	2	1	1	845	93.9	8.21		651368.6875
Q00577	Transcrip	3	1	1	322	34.9	6.44	594315.9918	621233.0625
A0A8Q3SIU	Ras GTPas	1	2	1	1416	159.6	8.7	5359638.837	6373301.625
Q9H0E3	Histone c	1	1	1	1048	110.3	9.83	572455.1	1618055.625
A8K4T9	cDNA FLJ7	3	1	1	291	30.4	9.47	2031723.659	1679860.625
Q4R9M9	Kinesin f	1	1	1	1809	203.5	5.55	980739.5177	17092996
B7Z8F4	RNA helic	1	1	1	1181	133.9	7.88	334048.3038	626288.4375
A0A8V8TNA	Disabled	3	2	1	518	58.1	8.19		547987
Q9Y6X9	ATPase MC	1	1	1	1032	117.8	8.38	185291.1291	898465.75
H0YA24	Pleiotrop	5	1	1	156	17.4	9.54	938879.9522	1754929.75
O00488	Zinc fing	7	1	1	134	15.2	9.82	1356636.183	1228170.125
Q8N7H5	RNA polym	2	1	1	531	59.9	4.63	493834.3595	686219.8125
B7Z9L3	Chloride	2	2	2	747	82.6	7.74		2589578.5
O75131	Copine-3	2	1	1	537	60.1	5.85	2168832.019	781287.625
Q15334	Lethal(2)	1	1	1	1064	115.3	6.29		991401
B3KMW2	cDNA FLJ1	1	1	1	726	78.7	9.85	322780.8321	696863.25
O00257	E3 SUMO-p	3	2	2	560	61.3	9.36	2888373.796	3926760.75
Q4G0R1	PIBF1 pro	1	1	1	698	83.1	5.53	480479.5326	614964.75
A0A5C2GAB	IGH + IGL	9	1	1	106	11.6	5.34	860648.1805	
J3KN16	Proteasom	1	2	2	2017	223.6	8.75		819229.875
Q6ZNW0	cDNA FLJ2	6	1	1	181	19.7	7.28	1792738.211	1060423.25
G9K389	YWHAE/FAM	3	1	1	384	41.3	4.97	618797.7724	340619.8438
B2RMQ4	Cytoskele	2	1	1	682	76.8	9.41	806464.767	1570793.875
E9PQ59	Nuclear f	1	1	1	987	108.2	8.29	3498655.205	8733625
P25788	Proteasom	5	2	2	255	28.4	5.33	3270485.506	1393216.25
Q9Y3R4	Sialidase	3	1	1	380	42.2	6.87	1202732.948	
P17010	Zinc fing	1	1	1	805	90.5	6.14	768819.3523	1649966.875
Q9UHB7	AF4/FMR2	1	1	1	1163	127.4	9.31	529510.4082	683692.5
F8WBC0	Ras-relat	34	1	1	32	3.5	9.42	2496495.949	1943468
O60909	Beta-1,4-	2	1	1	372	41.9	9.38	615545.8966	2177435
P49862	Kallikrei	6	1	1	253	27.5	8.47	1067702.068	602248.875
M0QYQ9	Splicing	16	1	1	51	5.5	4.82	921465.928	1765398.375
Q9BW60	Elongatio	4	1	1	279	32.6	9.6		9305509
B3KXC7	cDNA FLJ4	2	1	1	581	64	7.78	22589243.43	70224312
O14732	Inositol	3	1	1	288	31.3	6.61	682984.9922	180814.75
C9J3L8	Transloco	3	1	1	265	29.6	4.3	1569045.398	3059591
Q9BRT6	Protein L	12	1	1	129	15.2	10.37		681975.4375
Q96CP7	TLC domai	3	1	1	247	28.5	9.47		1241410.625
Q0VGM7	RBMX2 pro	6	1	1	173	19.8	9.22	816415.2411	1304184.625
Q2TAL8	Transcrip	1	1	1	776	86.4	5.87	700657.4029	635028.25
B4DHE8	RNA-bindi	2	1	1	324	34.8	8.88	356859.6023	1243634
B4DHV8	Alpha-1,3	6	2	2	254	29.5	8.82	3253039.652	6636057.875
A0A087WUE	Leukocyte	2	1	1	874	95.5	9.32		1685891.875
Q8IZH2	5’-3’ exo	1	1	1	1706	194	7.21	288187.5541	1107102.875
Q9BU23	Lipase ma	1	1	1	707	79.6	10.1		2610561.5
P33947	ER lumen	5	1	1	212	24.4	8.72	1816287.131	3308374
A0A1B0GVC	GTP-bindi	10	1	1	112	12.5	6.52	12224898.81	6703826.5
B7Z9T7	creatine	7	1	1	146	16.7	7.9	580340.4155	298002.7813
A0A5C2GF3	IGH + IGL	10	1	1	116	12.7	5.96	1946465.685	
H7BXZ6	Mitochond	2	1	1	597	68.4	6	252308.5802	393267.5313
A0A805TBS	Rho GTPas	1	2	2	1948	216.2	7.8	25206064.25	24832970.5
Q96EK4	THAP doma	3	1	1	314	34.4	8.98	707078.3475	2507256
B4DM82	Peptidyl-	7	1	1	129	14.1	8.47	1301519.667	628252.25
D6RF35	Vitamin D	2	1	1	476	53	5.52	2304679.733	1151642
O95639	Cleavage	4	1	1	269	30.2	8.31	399693.7942	591686.5
P0C7P4	Putative	3	1	1	283	30.8	8.87	544906.3851	1036446.375
B2RAQ8	carnitine	1	1	1	773	88.3	8.59	389556.0232	1094087.375
Q5BJH1	PSAP prot	4	1	1	240	26.3	6.04	939107.5352	385820.9375
E9PDS0	Nonsense-	1	1	1	1170	130.8	7.88		1020299.563

Q8WUY1	Protein T	4	1	1	208	23.9	9.55	952153.3916	1132121.125
Q96FX8	p53 apopt	4	1	1	193	21.4	7.03	8389099.226	2805268
O43167	Zinc fing	2	1	1	697	78.2	7.61		4591393
Q6FHZ0	Malate de	3	1	1	338	35.5	8.68	1063642.418	1007981.813
I3L1P4	Proline-,	21	1	1	148	15.1	10.68		
A0A590UJ2	Paralemmi	2	1	1	648	68.3	4.45		555134.3125
B4DPK9	cDNA FLJ5	1	1	1	835	91.9	4.6	486512.9883	589176.5625
O15031	Plexin-B2	1	1	1	1838	205	6.24	800166.7944	1102255
A0A024R7S	Clathrin	5	1	1	211	23.2	4.69	899863.3622	460733.1563
Q08AD1	Calmoduli	1	1	1	1489	168	6.8	272916.0594	720507.875
A0A5C2GGK	IGL c559	13	1	1	110	11.7	6.54	4400455.197	2215982.75
H3BP09	Mothers a	11	1	1	114	12.7	4.56	540491.5892	1020800.813
O75691	Small sub	0	1	1	2785	318.2	7.39		955209.8125
P12270	Nucleopro	0	1	1	2363	267.1	5.02		
B7Z6B6	Zinc fing	10	1	1	131	14.6	7.77	402574.8511	1013904.813
B3KMK7	cDNA FLJ1	3	1	1	528	59.6	6.23	567051.4537	921444.5
B4DGW2	cDNA FLJ5	1	1	1	1078	121.5	6.37	341501.7975	664910.75
Q2M1P5	Kinesin-l	1	1	1	1343	150.5	6.79	475009.5382	3543216
H3BSL4	Transcrip	2	1	1	607	64	5.39	507527.1482	1806216.25
Q9NPI1	Bromodoma	3	1	1	651	74.1	6.39		889314.8125
G3V4T2	Polyadeny	4	1	1	178	20.2	9.48	3586568.918	3960860.75
O60306	RNA helic	1	1	1	1485	171.2	6.37	508487.5508	793674.125
K7EKU0	G patch d	6	1	1	221	25.6	6.46	913495.6312	1337049.25
B4DTZ1	cDNA FLJ6	1	1	1	1012	113.4	8.65		
B3KRS8	cDNA FLJ3	2	1	1	422	46.8	8.34		925163.875
Q05DH1	Proteasom	5	1	1	238	26.7	8.87	1521253.938	
Q13185	Chromobox	8	1	1	183	20.8	5.33	368308.3008	658501.125
Q59FK3	Annexin (	5	1	1	225	25.4	6.38	840622.7503	1070072
A0A3G2LMK	CD59 bloc	17	1	1	71	8.2	5.77	2204681.654	2089977.125
P62633	CCHC-type	5	1	1	177	19.5	7.71	1753090.211	1492628.75
Q16658	Fascin OS	2	1	1	493	54.5	7.24	147508.6543	488847.0938
X6RLX0	ELKS/Rab6	1	1	1	1120	128.4	5.97	651636.2213	547901.4375
Q8TCG1	Protein (	1	1	1	905	102.1	6.23	794717.245	653938.125
A0A8V8TRF	Choline/e	2	1	1	416	46.6	8.16	8543630.488	4370090.5
A8K5H6	Exonuclea	1	1	1	845	93.8	8.4	986685.6063	1530395.125
A8K2T9	cDNA FLJ7	1	1	1	1011	116.5	6.05	764655.0306	1266589.75
E7ETM0	Casein ki	3	1	1	264	30.7	9.48	778306.4462	610263.6875
Q92830	Histone a	1	1	1	837	93.9	9.04	181543.6504	445629.3438
A0A7I2V45	Serine/th	1	1	1	861	94.1	6.07	1036609.208	827976.75
O95905	Protein e	2	1	1	644	72.7	4.87	920370.4735	1104334.125
Q9UKV8	Protein a	1	1	1	859	97.1	9.19		1272994.875
Q96GC5	39S ribos	5	1	1	212	23.9	8.98		
X6RGP5	Protein t	1	1	1	1059	115.2	6.14	659908.2105	1843251.5
Q9H2P0	Activity-	2	1	1	1102	123.5	7.34	677768.0388	960372.5625
A8K5C5	Protein F	2	1	1	675	77.3	6.07	1161475.801	762899.375
Q71UM5	40S ribos	10	1	1	84	9.5	9.45	1466497.762	3171258.5
B4DYL7	cDNA FLJ5	1	1	1	1227	139.6	7.02		1460675.375
B4DXV3	cDNA FLJ6	2	1	1	578	67.9	6.33	354621.3707	1169988.125
Q9Y5Z9	UbiA pren	3	1	1	338	36.8	8.15		1113357
B4DPW5	cDNA FLJ5	2	1	1	497	57.4	8.22	980616.1832	1050242
P21796	Voltage-d	4	1	1	283	30.8	8.54	1603635.837	802798.9375
B4DJI1	L-lactate	3	1	1	305	33.6	8.46	3074887.957	
Q5H9F3	BCL-6 cor	1	1	1	1785	190.4	7.85		700154.25
A0A4D5RAA	Annexin (	2	1	1	282	31.5	8.09	755110.4974	353874.5
P36021	Monocarbo	2	1	1	539	59.5	5.59	1627903.021	3431316.5
A0A7I2V3J	Laminin s	1	1	1	1203	132.8	4.96		516440.4375
A0A7P0Z44	Alpha-1,3	2	1	1	527	60.1	9.14		1917032
Q15050	Ribosome	2	1	1	365	41.2	10.7	2255052.146	4082154.5
O00151	PDZ and L	3	1	1	329	36	7.02	964403.7343	1774364
B9A6K8	TBC1 doma	1	1	1	1250	140.4	5.24	329108.7021	1097213.875
Q8NBQ5	Estradiol	3	1	1	300	32.9	9.07	2002472.602	1329729.25
P57740	Nuclear p	1	1	1	925	106.3	5.43	762978.7201	762690.4375
Q71SV8	Crn-relat	1	1	1	852	99.6	6.23	1057516.522	3248872.5
A0A1B0GVD	Protein l	4	1	1	258	27.9	9.13	851346.33	1695143.875
A0A6I8PIK	Polyadeny	14	1	1	73	8.4	10.51	1197639.994	
P38432	Coilin OS	2	1	1	576	62.6	9.07		589740.5625
H3BV16	NADH dehy	10	1	1	142	17.4	6.55		655070.8125
A0A7S5C2R	IGH c2757	13	1	1	122	13.4	8.47	3555262.003	
Q96EP5	DAZ-assoc	4	1	1	407	43.4	8.56	1316021.68	579909.4375
A2N0U5	VH6DJ pro	6	1	1	109	12.2	5.36	5693300.015	188698.2656
A0A024RCA	Solute ca	3	1	1	249	26.6	9.44	768332.1145	1506994.5
Q9BQS8	FYVE and	1	1	1	1478	166.9	4.92	2771637.496	1529378.125
Q8IWV8	E3 ubiqui	0	1	1	1755	200.4	6.24	7477276.16	2869821.5
O60318	Germinal-	1	1	1	1980	218.3	6.39		1490502
B4DIW9	Serine/th	1	1	1	722	77.7	6.99	598439.7859	586482.75
O14641	Segment p	1	1	1	736	78.9	6.02	736562.1218	920393.4375
B2R7L2	Annexin (	2	1	1	466	50.3	6.61	1867097.448	
O14992	Heat shoc	2	1	1	474	52.3	8.21	665804.9532	603883.5625
B4DTN6	cDNA FLJ6	1	1	1	843	93.6	8.87	431974.0367	977649.5625
Q15828	Cystatin-	7	1	1	149	16.5	8.09	1568829.653	
A0A7I2V4D	Nodal mod	1	1	1	800	87.4	6.05	745928.7803	1007648.75
Q9NVI1	Fanconi a	1	1	1	1328	149.2	6.74		1063781.75
B5MDE0	Protein F	2	1	1	502	55.8	8.72	578573.265	
Q8IXG2	UG0656C10	2	1	1	392	43.8	5.53	2206081.941	4468607
Q5JTZ9	Alanine--	1	1	1	985	107.3	6.27	667545.045	1067477.5
K7ERZ5	DNA ligas	3	1	1	545	61.7	9.2	1212021.692	469351.6563
Q68DW7	Cohesin s	1	1	1	998	114.9	5.44	777042.9016	1361515
B3KNK2	cDNA FLJ1	1	1	1	674	73.6	5.08	1250898.761	1934791
F5GXT0	Double-st	5	1	1	221	25	5.44	601922.3853	543369.9375
D6REJ4	Dachshund	6	1	1	175	18.7	8.5		982946
Q8N1F8	Serine/th	1	1	1	1088	120.2	5.36	1284633.71	2370446.25
P55081	Microfibr	3	1	1	439	51.9	4.98	518251.2716	345298.5
Q9H1A4	Anaphase-	0	1	1	1944	216.4	6.3	171452.5526	417932.0625
H0Y3U6	Glypican-	5	1	1	310	35.1	6.24		858067.25
Q8WXE0	Caskin-2	1	1	1	1202	126.7	7.09	2137697.362	1300837
B4DM79	cDNA FLJ5	1	1	1	543	60.7	5.35	550224.9897	280471.7813
A0A1B0GVA	Ryanodine	21	1	1	33	3.9	4.55	416337.4609	1433756.875
Q9H8F6	cDNA FLJ1	4	1	1	176	19.9	5.34	82427143.71	63360580
Q8N5G2	Macoilin	2	1	1	664	76.1	9.07		1284501.25
A0A0C4DGB	rRNA aden	4	1	1	275	30.8	10.24	497236.9606	931705.0625
Q8WXE9	Stonin-2	1	1	1	905	101.1	5.39	1271703.397	966158.125
B4DSC2	Elongatio	3	1	1	289	34.1	9.38	635876.2204	2654249.5
K7EQE8	Derlin-2	13	1	1	82	9.6	8.92		
P49720	Proteasom	3	1	1	205	22.9	6.55	921046.337	409947.6563
A0A384DVU	Patatin-l	1	1	1	1365	149.8	7.81	1193262.887	985153.25
A0A087WVM	formate--	1	1	1	913	99.2	7.27	719137.1654	657119.0625
G3V1R7	Long-chai	2	1	1	385	44	8.32	1604896.362	2493007.25
B3KMV5	cDNA FLJ1	1	1	1	1104	122.8	5.85	518969.7163	971174.25
Q6P1N1	PPM2C pro	1	1	1	606	68.4	7.9	7634216.13	6230802.5
A0A6Q8PGB	Centrosom	0	1	1	2766	323	5.96		765620.3125
P82933	28S ribos	3	1	1	396	45.8	9.51	657501.315	1434909
A0A2P1M8C	Kelch-lik	2	1	1	444	49.5	6.87	896418.814	
Q75MU2	Eukaryoti	8	1	1	156	17.1	5.31	2568981.613	1260539.875
O95969	Secretogl	10	1	1	90	9.9	8.25	4227122.971	714211.25
Q8TAG3	Anion exc	1	1	1	1159	127.7	6.49	420999.2882	974431.6875
Q8WVS4	Cytoplasm	1	1	1	1066	122.5	7.31	497570.1511	787450.875
A0A0G2JHN	Bromodoma	2	1	1	328	35.8	6.62	909342.3626	1055529
A0A024R0F	Tripartit	1	1	1	1127	122.4	6.67		2064241.75
Q5QPA5	39S ribos	3	1	1	263	29.6	10.7	2372800.973	848378.1875
Q71SM9	Dyad symm	9	1	1	255	28.5	9.31	77261220.63	
B3KN91	Putative	3	1	1	327	35.9	6.09	350727.1529	475590.6875
F5H2X7	Cip1-inte	1	1	1	954	106.2	6.74	1082827.639	1235643.375
B4DE91	cDNA FLJ5	2	1	1	484	52.7	6.06	409886.649	
J3QQT0	[F-actin]	3	1	1	269	31.1	9.47	10548316.71	4383245
M0QYV5	ATP-depen	5	1	1	143	16.1	9.83	975213.1408	696047.75
F8WCT1	ADP-ribos	5	1	1	229	25.6	10.95	680719.4302	332329.5938
Q9HD33	39S ribos	4	1	1	250	29.4	10.37	487454.2142	535769.75
A0A6E1XQM	Protein f	2	1	1	1233	139.9	7.3	1717202.283	6987818.5
A6NKM8	UDP-galac	7	1	1	218	23.5	10.56	2100051.705	2915830.5
A0A0A0MT2	PI-PLC X	8	1	1	88	9.9	8.54	854493.7761	594095.75
A0A1B0GWD	UBAP1-MVE	11	1	1	71	8.2	7.25	923198.5303	315193.625
E9PE17	28S ribos	7	1	1	129	14.4	9.85	2914243.845	2582521
Q13111	Chromatin	1	1	1	956	106.8	5.94	2729753.771	627077.25
D3DSU2	Homeobox	2	1	1	406	46.1	6.19	1753201.828	7378939
B4DFG8	PRA1 fami	2	1	1	292	31.5	8.82	3994904.252	2310409
A0A8Q3SHF	La-relate	3	1	1	581	66.8	9.55	365560.9671	760112.4375
B7ZM68	FGD5 prot	1	1	1	1178	129.4	5.4	293777.8933	374076
B4DJ30	cDNA FLJ6	1	1	1	995	112.9	6.06	1159018.05	883549.625
Q3LIE7	Uncharact	2	1	1	427	49.4	7.55	745000.9327	1587218.625
A0A590UJQ	E3 ubiqui	1	1	1	900	103.1	6.48		1108576.25
F8WC81	WD repeat	7	1	1	97	11.1	7.93	9037066.154	5135184
A0A024RBC	Calcium-t	1	1	1	1220	134.6	5.91	438371.1238	605411.5
Q6P1M0	Long-chai	2	1	1	643	72	8.47		1561128.125
A0A0U1RQT	Synaptoph	8	1	1	135	15	5.77	812433.8676	595984.25
C9K0I9	Unconvent	10	1	1	83	9.7	5.39	2943083.103	5459206
E5RI99	60S ribos	11	1	1	114	12.6	9.55	7789376.024	3974494.75
Q86SX2	Full-leng	5	1	1	139	15.6	9.23	3385517.471	1683084.5
Q6P1J9	Parafibro	1	1	1	531	60.5	9.61	393904.9239	599732.0625
A2A3S3	Musclebli	2	1	1	361	39.3	8.43	1330846.706	1411303.125
A0A024RCR	Large pro	1	1	1	1126	118.6	5.6	575403.8337	751748.8125
F8WF02	Pyruvate	3	1	1	251	27.6	7.9		1475708.125
F2FB34	SOGA OS=H	1	1	1	1351	151.2	6.44		429949.75
Q7Z3C6	Autophagy	1	1	1	839	94.4	6.67		
Q13601	KRR1 smal	2	1	1	381	43.6	9.77	1310575.634	2492553.75
K7ELS8	Synaptogy	7	1	1	135	14.7	8.09	1069063.822	
F8WBJ6	RNA-bindi	7	1	1	136	15.2	9.82		
Q9BTC0	Death-ind	0	1	1	2240	243.7	7.88	548503.38	1867408
A0A5C2G7H	IGL c424	17	1	1	107	11.6	8.94	506495.1427	779238.9375
P35754	Glutaredo	10	1	1	106	11.8	8.09	747450.2281	265717.8438
Q86UH7	Envelope	1	1	1	682	77.1	8.69	20138426.91	7780094
Q9HC84	Mucin-5B	0	1	1	5762	596	6.64	1185459.593	4518075
E9PLA7	Nuclear F	13	1	1	115	13.2	9.76	372816.0505	833356.0625
E7EUJ7	Cancer-as	1	1	1	817	95	5.49	9679256.051	3250711.5
P25786	Proteasom	3	1	1	263	29.5	6.61	1756247.744	924119.8125
C9JJV6	Myeloid-a	7	1	1	146	15.9	8.65	2985462.098	4921068
Q99848	Probable	2	1	1	306	34.8	10.1	660470.5844	837430.0625
A0A5C2FUQ	IGL c748_	11	1	1	113	12.6	7.97	7965396.725	766482.5
A4VCI6	HIP1 prot	2	1	1	517	57.7	4.77	7516713.73	17351240
A4FU69	EF-hand c	1	1	1	1503	173.3	5.82	1199502.695	1360296.625
H3BR99	Synaptoso	14	1	1	90	10.3	6.52	2670576.155	1282152.875
B4DT57	cDNA FLJ6	2	1	1	450	50	5.6		887582.0625
E7EUI6	Integrin	1	1	1	801	88.7	5.44	1090613.176	665254.6875
Q9NPL8	Complex I	4	1	1	285	32.2	8.5	1180519.448	1561542.75
A0A0A0MTL	Rho famil	1	1	1	1238	134	6.43		

Q9UQL6	Histone d	1	1	1	1122	121.9	6.24	13338673.51	2834681
B7WP74	Pre-mRNA-	1	1	1	745	85.5	5.71	2247880.142	
B3KT27	cDNA FLJ3	6	1	1	130	14.8	10.36	755686.6424	1448492
Q2VY69	Zinc fing	3	1	1	593	69	8.4	981038.7941	812278.625
H0Y9I3	Exportin-	9	1	1	107	12.1	8.68	384101.974	711728.8125
Q96JM4	Leucine-r	0	1	1	1722	199.2	6.19		1386414.375
Q5T1S8	Noncompac	19	1	1	102	11.1	9.86	2188645.751	962905.1875
H0YD93	RUN and F	1	1	1	620	71.4	5.99	2710534.122	1556446.5
A4FS09	Minichrom	11	1	1	85	9.4	8.44	989300.2739	
A0A8V8TQK	WD repeat	1	1	1	572	62.3	7.24	856806.8117	1498199.125
Q17R88	Sortilin-	1	1	1	1222	135.7	6.6	1849770.821	5042329.5
P23470	Receptor-	1	1	1	1445	161.9	6.42		326296.9375
H0YKX0	RBR-type	1	1	1	917	102.3	6.73	3885624.556	10418513
J3QLU9	Receptor	1	1	1	1055	117	6.54	960614.1877	945818.1875
P10745	Retinol-b	1	1	1	1247	135.3	5.11	2481375.935	3064197
Q0IIN5	VCP prote	3	1	1	475	52.4	5.02		
Q6ZU65	Ubinuclei	1	1	1	1347	146	9.19	893923.0114	1285110.625
A0A5C2G8Z	IGL c4177	10	1	1	107	11.4	8.47	690441.9589	
A0A7S5BYD	IGH c487_	6	1	1	125	13.5	6.6	107625989.9	15104349
B7Z671	cDNA FLJ5	2	1	1	388	42.8	4.58		1537981.75
A0A5C2G2D	IGL c2606	6	1	1	112	12.4	7.99	11444672.83	201849.9375
Q6IAX9	SRPR prot	1	1	1	638	69.9	9	527100.6137	1274224.75
Q96N64	PWWP doma	2	1	1	755	81.9	9		345034.0625
P62829	60S ribos	7	1	1	140	14.9	10.51	1569916.954	1645168.25
Q6TGM0	Mutant ad	4	1	1	240	26.7	5.67	2277148.231	7717702.5
Q00587	Cdc42 eff	2	1	1	391	40.3	7.15	1682206.688	2468944
V9GYZ0	Pantothen	4	1	1	171	18.7	9.98	1583992.792	4676319
D3DRR9	Chromosom	5	1	1	239	24.8	11.71		2584111.75
Q9BTD8	RNA-bindi	2	1	1	480	50.4	9.63	527415.1106	1387979.625
Q9H6S7	cDNA: FLJ	1	1	1	724	81.2	8.85	440332.6077	1089269.5
B3KVZ3	Centromer	3	1	1	228	26.2	4.93	1298408.037	312459.5625
A0A059RIK	Cytochrom	3	1	1	227	25.5	4.89	1128641.64	380097.9063
B4DEU4	Calpain-3	9	1	1	266	30.6	5.47	660948.8228	1282806.75
H0YIW7	Probable	6	1	1	189	21.2	8.41	2423457.756	9151728
Q7LAX7	Growth ar	8	1	1	124	14.5	9.61	175098.682	497007.9375
Q96DP0	NADH dehy	2	1	1	456	49.2	8.98	606606.9226	938595.125
A0A0S2Z6F	Histocomp	2	1	1	345	38	6.86	2500784.995	3714570
Q96QD9	UAP56-int	3	1	1	318	35.8	11.78	3104816.328	2039812.75
J3QR07	YTH domai	1	1	1	735	85.5	6.23	1044186.247	1008490.25
A0A0D9SFB	dynamin (	1	1	1	835	94	6.64	459114.4854	485752.0938
Q6UXP6	LKHP9428	3	1	1	218	23.9	6.28	3490452.796	4763731.5
Q6WKZ4	Rab11 fam	1	1	1	1283	137.1	5.43	1899847.171	4111903.5
S6AWD3	IgG L cha	8	1	1	183	19.9	8.91	2860621.96	
Q96M86	Dynein he	0	1	1	4753	533.3	6.71	1865556.673	
P57771	Regulator	4	1	1	180	20.9	9.26	11544720.43	8639556
Q9Y6D5	Brefeldin	0	1	1	1785	201.9	6.33	2119390.944	2805468.5
A0A8V8TQT	N-alpha-a	2	1	1	428	50.4	8.68	1268479.184	718639.875
B4DL80	cDNA FLJ5	1	1	1	763	84.8	7.31		640822.875
Q96DR8	Mucin-lik	10	1	1	90	9	4.64	3299164.224	
Q9P242	Neuronal	2	1	1	653	70.5	8.48		6749676
F8WF16	Periphili	2	1	1	303	34.8	8.02	1891106.245	1031658.563
F8WDV0	Importin-	1	1	1	863	99.6	5.58	7389415.599	4390684.5
Q6UWH6	Protein T	4	1	1	196	22.5	7.11	923373.4455	1503965.875
Q5VVY2	cDNA FLJ4	6	1	1	115	13.6	5.94	1177084.926	1911699
H7C0C7	CCR4-NOT	6	1	1	127	14.3	5.82	57370657.2	91610208
A0A3B3IS2	Complemen	8	1	1	146	16.4	7.09	6036008.099	2816378.5
F5H1Y3	Flap endo	7	1	1	165	18.3	7.37	301862.7366	843055.125
A8K1K0	cDNA FLJ7	1	1	1	612	69.8	5.35	24871725.85	37065972
M0QYV2	Acid phos	3	1	1	227	26.8	7.94	15541808.23	27441632
Q96CN4	EVI5-like	1	1	1	794	91.3	5.34		1494249.125
H0YED3	Nuclear p	6	1	1	113	12.1	7.84	237775.4165	719966.125
E7ENL6	Collagen	1	1	1	638	69.7	9.32	10981957.48	6340875.5
Q53EU2	GATA bind	2	1	1	595	60	8.35	1849581.048	2188435
A0A0J9YW1	RNA-bindi	8	1	1	106	12.3	8.72	1283599.198	397301.2188
A0A5C2GHD	IG c462_1	15	1	1	107	11.8	8.46	2031901.232	
Q9UJV9	Probable	2	1	1	622	69.8	6.84	4141558.534	3004112.75
Q92547	DNA topoi	1	1	1	1522	170.6	6.96	360384.4206	634421.375
B3KR68	cDNA FLJ3	11	1	1	394	43.6	7.84		
Q8NGY6	Olfactory	2	1	1	317	35.7	8.82	1276169.29	1836473
B4DMD3	cDNA FLJ5	1	1	1	589	64.9	7.01	2628417.011	2424561.25
E9PF06	39S ribos	4	1	1	176	19.6	9.2	871765.6818	1615335.25
A0JLQ5	Ribosome	2	1	1	335	39.1	9.91	406329.8495	1603084.75
A0A5C2GAC	IGH + IGL	14	1	1	118	12.8	6.58	13169316.01	
Q9NWB7	Intraflag	2	1	1	429	49.1	4.98	2701926.315	2043442.75
A0A5C2G5G	IGH c236_	5	1	1	129	14.1	7.28	10935888.12	6529816
H7BZ55	Ciliary r	0	1	1	1653	185.5	5.5	46534889.17	186270464
A0A024R8D	Mitochond	3	1	1	319	35.5	8.94	880986.779	1566094.25
Q15170	Transcrip	6	1	1	159	18.6	5.02	905355.3116	
A0A8I5KP8	KN motif	1	1	1	1128	122.9	5.22	971435.1908	1582571
Q8IY18	Structura	1	1	1	1101	128.7	8.38		
E9PBS1	Bifunctio	2	1	1	413	45.6	6.68	796015.4585	503528.75
A4UCS5	Peroxired	5	1	1	154	16.6	7.47	391512.8866	373512.4375
O14981	TATA-bind	1	1	1	1849	206.8	6.52	265133.6031	762586.875
Q6P0N0	Mis18-bin	1	1	1	1132	129	9.25	1152559.477	3937672.75
A0A8V8TNC	F-BAR dom	1	1	1	797	87.7	7.42	4678752.948	1592178.875
Q53ZX8	Protease	24	1	1	84	9.1	9.47	15405348.31	9198792
B4DH29	DNA-direc	1	1	1	1099	125.1	8.29	392768.6699	536669.9375
Q9BXT2	Voltage-d	3	1	1	260	28.1	9.17	1273832.941	1409322.375
Q6ZV73	FYVE, Rho	1	1	1	1430	160.7	7.03	3740554.657	1359329
A0A6I8PU7	DnaJ homo	1	1	1	497	56.7	7.14	1245805.202	2903895.25
H7C114	Protein p	12	1	1	60	6.2	4.94	346349.7439	1168540.875
P36222	Chitinase	2	1	1	383	42.6	8.46	108792954.3	29148982
Q7Z3R8	DNA-direc	1	1	1	1133	127.7	8.5		255483.7344
Q14257	Reticuloc	3	1	1	317	36.9	4.4	994589.3435	1374039.25
A0A7S5C21	IGH c2422	13	1	1	125	13.8	8.44		10105505
H7C3G1	Protein E	5	1	1	209	24	7.91	437593.9412	1058427
U3KQH5	Nuclear p	26	1	1	42	4.7	11.05		3623576.5
Q99735	Microsoma	10	1	1	147	16.6	9.55		
D6RA20	Protocadh	1	1	1	901	97.2	5	1622437.043	987480.75
K7ELW0	Parkinson	4	1	1	169	17.9	7.87	1141999.336	665874.625
A8K6X9	cDNA FLJ7	1	1	1	1173	133.4	6.77	1245146.009	928847.8125
C9JNQ3	Phosphori	7	1	1	104	11.5	9.72	9776805.559	45743460
A0A142CHG	GO2-q chi	3	1	1	354	40.2	6.05	1370741.738	1948148.25
A6NGG3	Putative	10	1	1	77	8.5	9.45	6053863.457	7512154.5
Q5CZC0	Fibrous s	0	1	1	6907	780.1	6.71	3372135.736	6712026.5
Q8NHP8	Putative	1	1	1	589	65.4	6.8	269326.4934	1464431.375
H7BXS9	Trinucleo	6	1	1	407	42.6	5.43		925252.9375
E9PR46	RNA helic	3	1	1	407	46.5	6.98	589207.3337	1056877.5
A0A5C2FTW	IGL c162_	12	1	1	112	12.5	8.48		2452725.25
C9JC24	WD repeat	6	1	1	162	17.7	8.34	44648352.83	25128108
A0A5C2G1N	IGL c1855	8	1	1	108	11.6	7.12	4182892.629	294879.5938
Q9UBW7	Zinc fing	1	1	1	1377	154.8	6.34	252232.7041	501307.2813
H0YK45	Probable	5	1	1	165	18	5.57		4850387.5
Q8N5Z0	Kynurenin	2	1	1	425	47.3	6.96	6394965.898	
K7ERQ8	PCAF_N do	4	1	1	282	31.4	7.42	1667668.315	
B4DEP3	cDNA FLJ5	1	1	1	1213	133.3	5.94	4111802.662	
A0A5C2GL7	IG c322_1	8	1	1	108	11.9	5.99	2158128.37	
B5BUA4	Shugoshin	1	1	1	527	60.1	9.2	2480209.512	1439003.375
C0JYZ2	Titin OS=	0	1	1	33423	3711.4	6.52	5474537.199	3668603.75
Q5FWF5	N-acetylt	1	1	1	840	94.9	9.16	2089815.545	2071879.625
Q9H078	Caseinoly	1	1	1	707	78.7	9.01	1298370.953	8478560
