# Supplementary material for: CLIC3 interacts with NAT10 to inhibit N4-acetylcytidine modification of p21 mRNA and promote bladder cancer progression
Source: Cell Death Dis. 2024 Jan 5;15(1):9. doi: 10.1038/s41419-023-06373-z (PMC10770081; doi:10.1038/s41419-023-06373-z)

Figure 2F

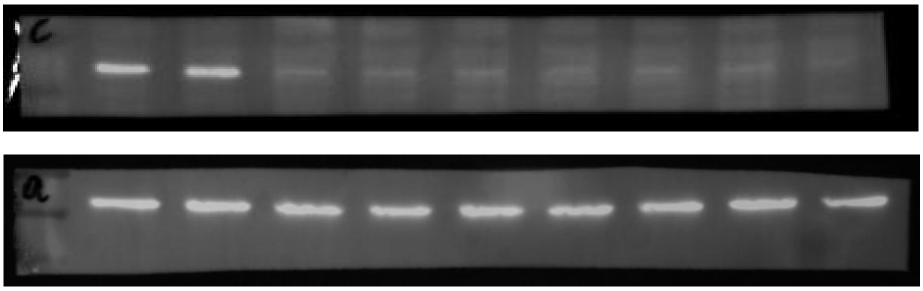

Figure 2I

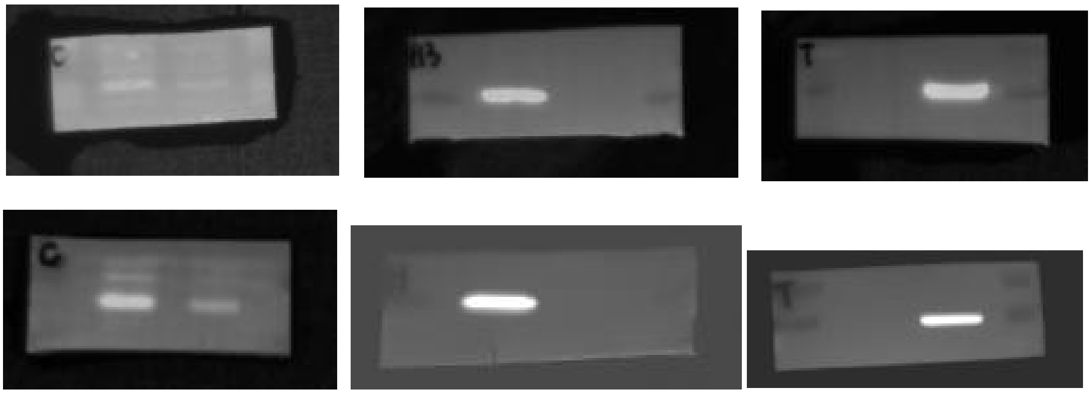

Figure 3A

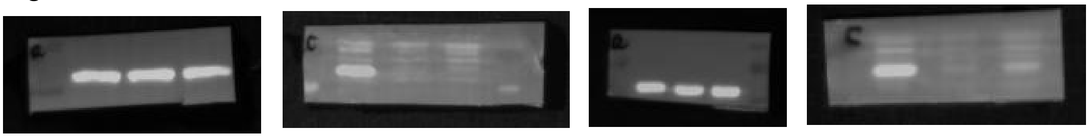

Figure 4E

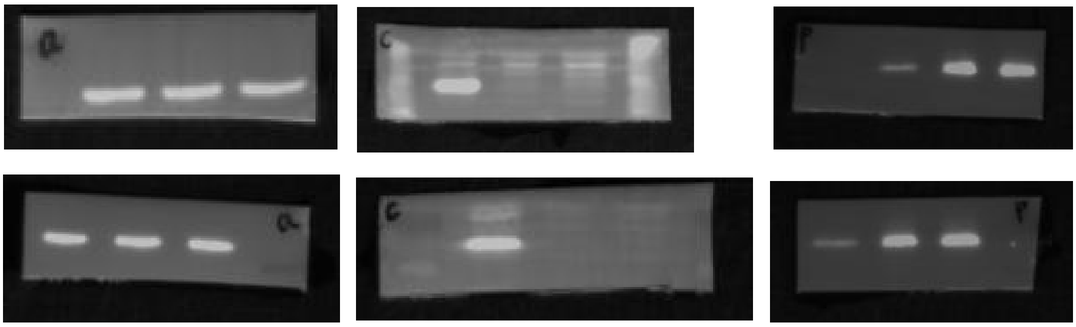

Figure 5A

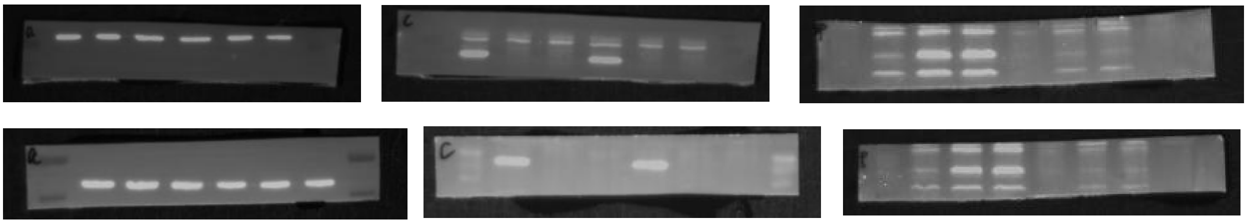

Figure 6E

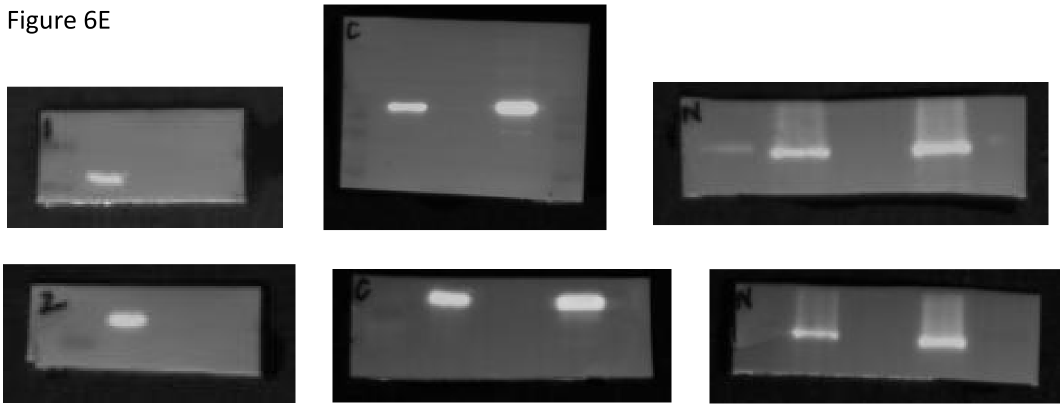

Figure 6F

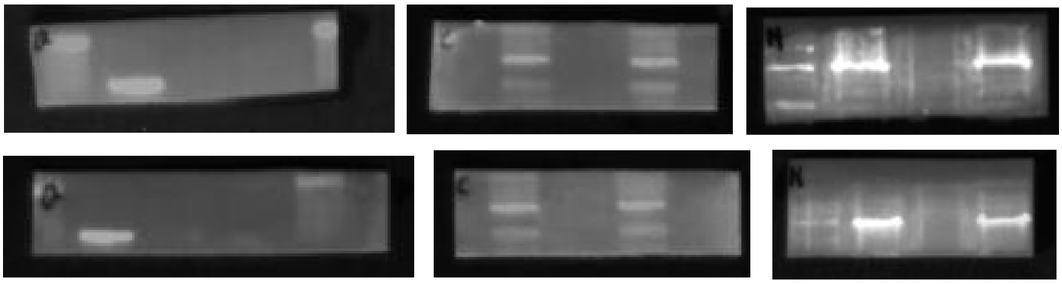

Figure 6I

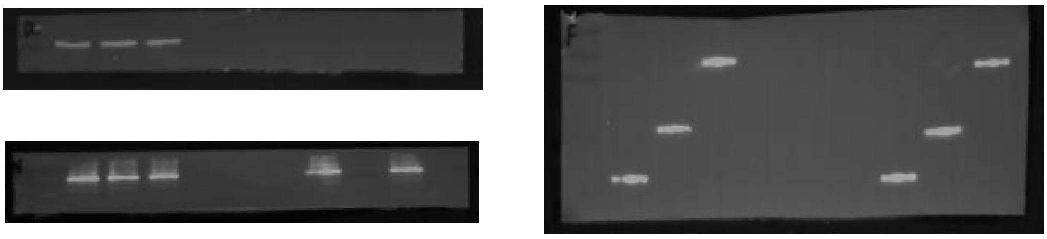

Figure 6J

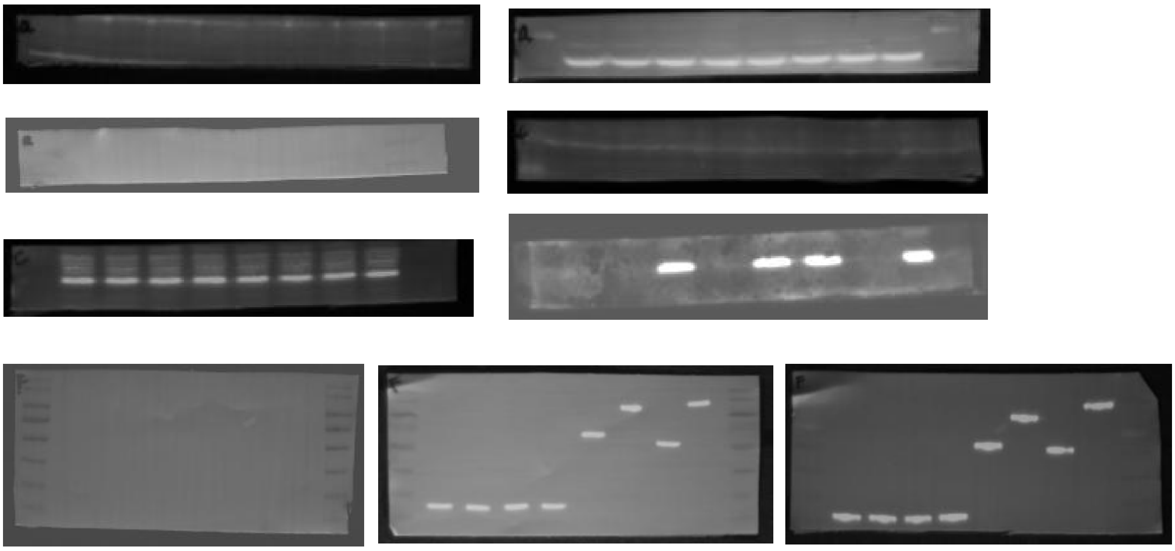

Figure 7C

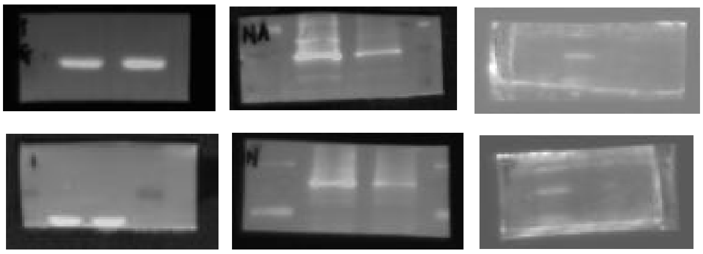

Figure 7D

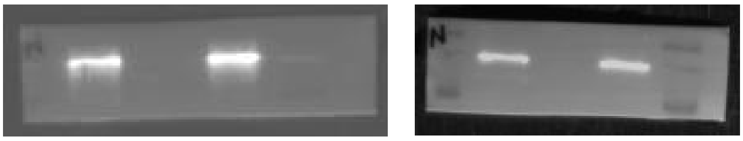

Figure 7G

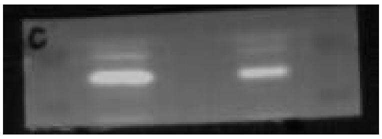

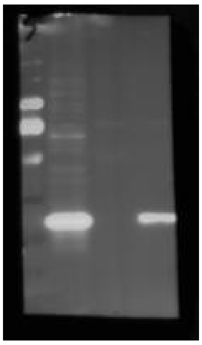

Figure S3C

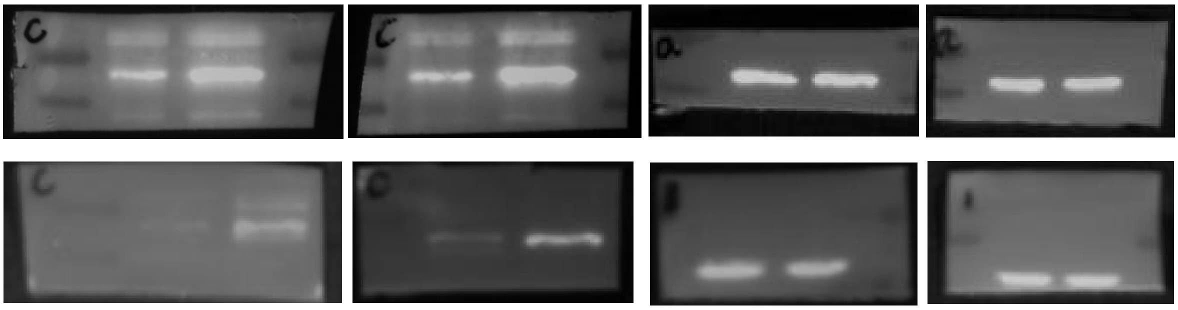

Figure S4A

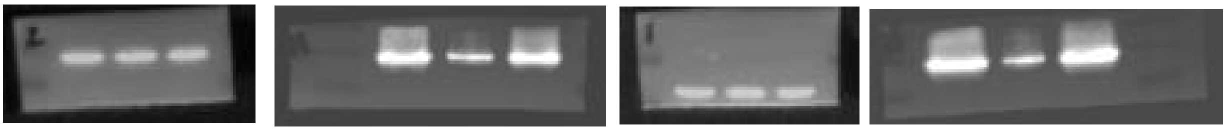

Figure S4B

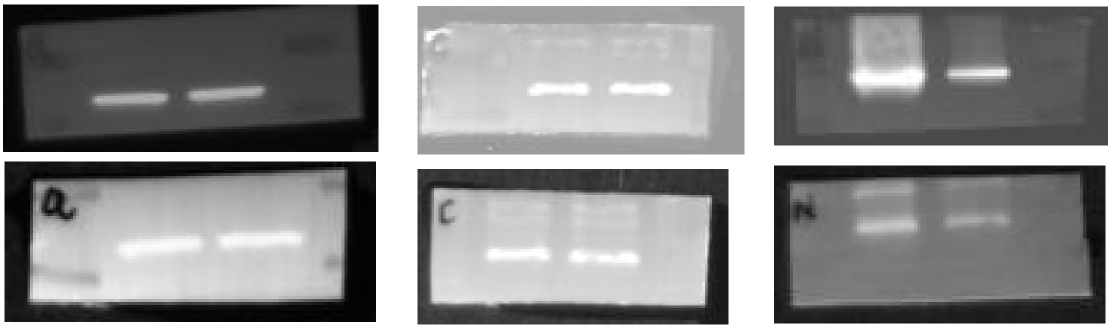

Figure S4C

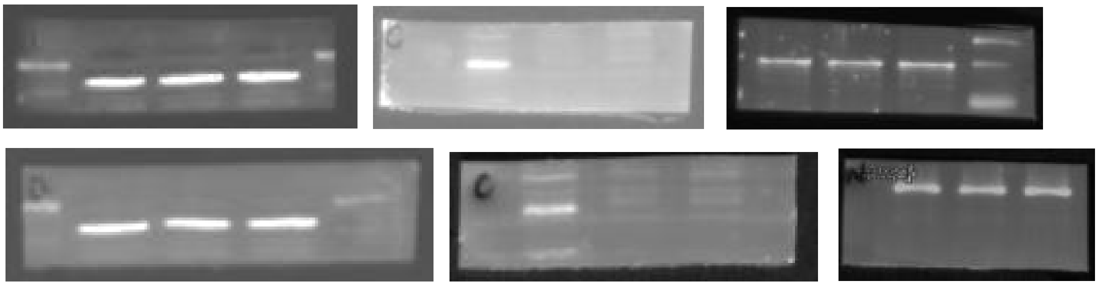

Figure S4H

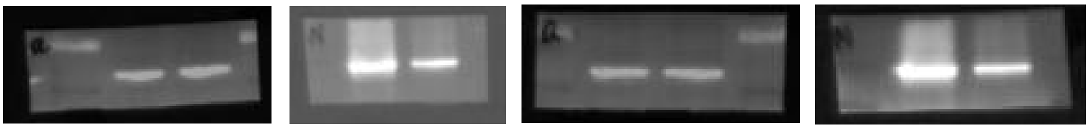

Figure S5B

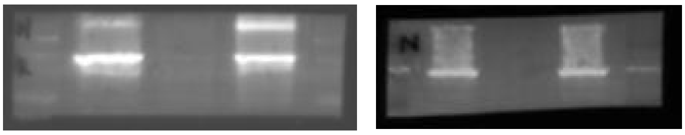

Supplement: Supplementary file 3 — Supplementary Figures [file 41419_2023_6373_MOESM3_ESM.pdf]
